# Highly Stereoselective Glycosylation Reactions of Furanoside Derivatives via Rhenium (V) Catalysis

**DOI:** 10.1021/acs.joc.1c00706

**Published:** 2021-05-25

**Authors:** Emanuele Casali, Sirwan T. Othman, Ahmed A. Dezaye, Debora Chiodi, Alessio Porta, Giuseppe Zanoni

**Affiliations:** † Department of Chemistry, University of Pavia, Viale Taramelli, 12, Pavia 27100, Italy; ‡ Department of Chemistry, College of Science, Salahaddin University-Erbil, Erbil 44002, Iraq; § International University of Erbil, Newroz Street, Erbil-Kurdistan 44001, Iraq

## Abstract

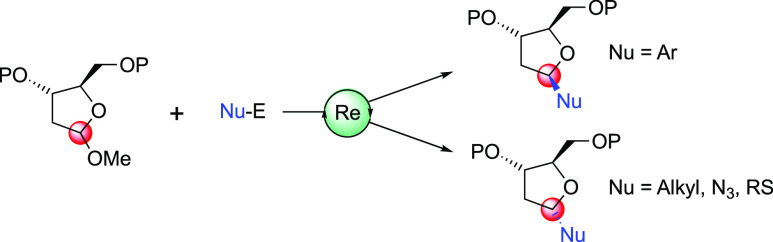

A novel approach for the formation of anomeric carbon-functionalized furanoside systems was accomplished through the employment of an oxo-rhenium catalyst. The transformation boasts a broad range of nucleophiles including allylsilanes, enol ethers, and aromatics in addition to sulfur, nitrogen, and hydride donors, able to react with an oxocarbenium ion intermediate derived from furanosidic structures. The excellent stereoselectivities observed followed the Woerpel model, ultimately providing 1,3-*cis*-1,4-*trans* systems. In the case of electron-rich aromatic nucleophiles, an equilibration occurs at the anomeric center with the selective formation of 1,3-*trans*-1,4-*cis* systems. This anomalous result was rationalized through density functional theory calculations. Different oxocarbenium ions such as those derived from dihydroisobenzofuran, pyrrolidine, and oxazolidine heterocycles can also be used as a substrate for the oxo-Re-mediated allylation reaction.

## Introduction

**Figure 1:**
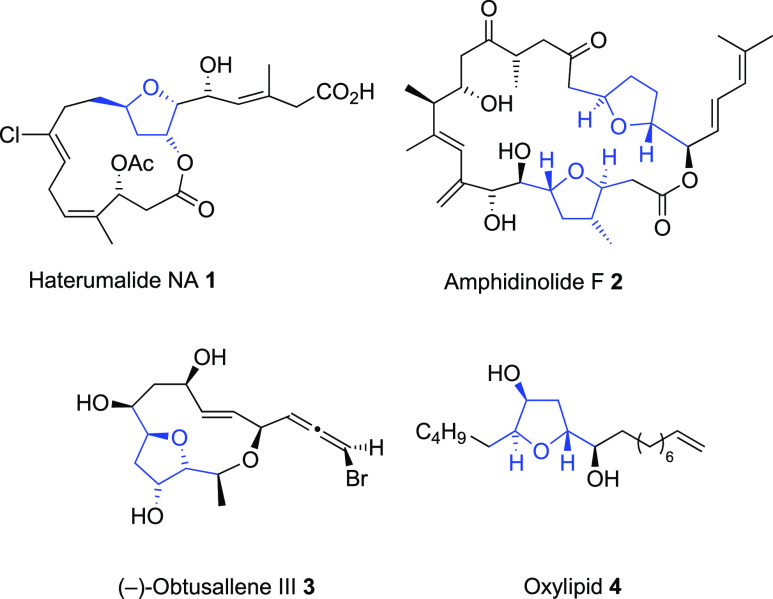
Representative members of the *trans*-2,5-dialkyl-substituted THF family.

Decorated tetrahydrofurans (THFs) are prevalent motifs in the structures of a myriad of natural products. In particular, the *trans*-1,4-dialkyl-substituted THF ring is a common functionality in biologically active compounds, polyether antibiotics, acetogenins, *C*-glycosides, and amphidinolides.^1^Figure 1 illustrates a few examples of natural products that contain this THF ring systems, such as haterumalides NA **1**, amphidinolide F **2**, (−)-obtusallene III **3**, and oxylipid **4**.

**Scheme 1:**
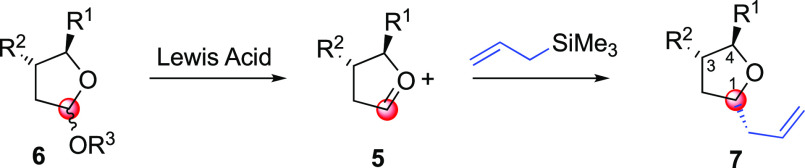
General Approach to the Trisubstituted THF Core via the Sakurai–Hosomi Reaction

Among the different synthetic strategies to assembly-substituted THFs,^1^ one of the classical approaches is the venerable Sakurai–Hosomi reaction (Scheme 1), which operates through the exposure of a suitable THF acetal, such as **6**, to a strong Lewis acid (e.g., TMSOTf, SnCl_4_, BF_3_·Et_2_O, or TiCl_4_) and soft, nucleophilic allylsilanes to afford the corresponding allylated THF product **7**.^2^

**Scheme 2:**
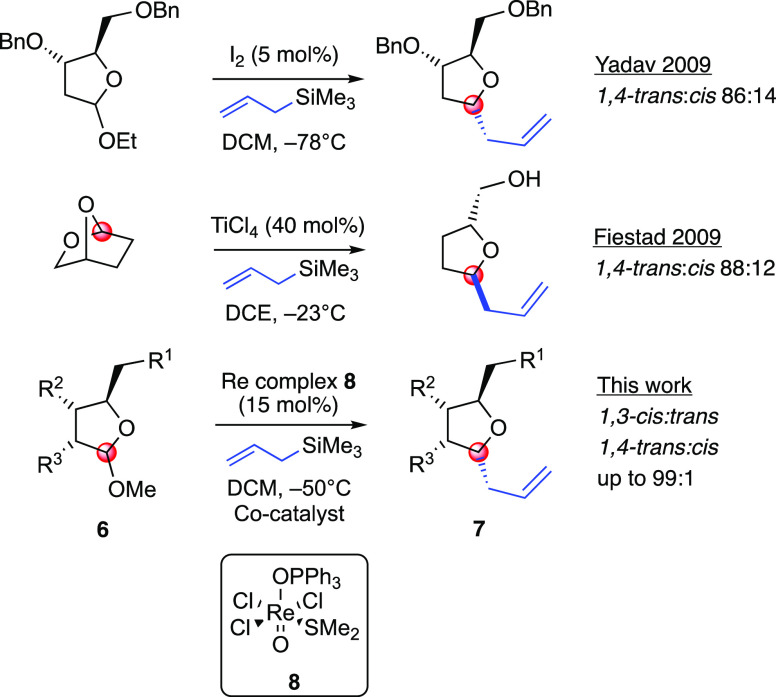
Catalytic Approaches to the Allylation Reaction via a Five-Membered Ring-Oxocarbenium Intermediate DCE = dichloroethane and DCM = dichloromethane.

However, this reaction is limited by the highly corrosive and moisture-sensitive Lewis acids that are required in stoichiometric amounts to provide the desired products. Therefore, an affordable and reliable catalytic system that could promote an efficient and stereoselective reaction would be advantageous considering both the commercial availability and the low synthetic manipulation of THF acetals. To the best of our knowledge, only two catalytic approaches have been developed for the allylation of THF acetals. In 2009, Yadav reported a protocol using molecular iodine in CH_2_Cl_2_ at cryogenic temperatures.^3^ In the same year, Fiestad delivered an alternative approach to solve the innate stereochemical problems of cyclic, five-membered oxocarbenium ions, developing an allylation reaction on a 2,7-dioxabicyclo[2.2.1]heptane ring system which uses a catalytic amount of TiCl_4_ as a Lewis acid.^4^ As a part of our program aimed to explore the chemistry of rhenium (V),^5^ herein, we report a new talent of oxo–rhenium complex **8**, namely, its ability to promote the formation of an oxocarbenium species in a catalytic fashion (Scheme 2).

## Results and Discussion

In the course of our studies on rhenium (V) chemistry, we discovered that Lewis acidic oxo-rhenium complex **8** was catalytically competent in the promotion of Meyer Schuster rearrangements and one-pot Meyer Schuster/Diels–Alder reaction sequences. Indeed, the 14e^–^ complex **8** exhibited an efficiency even higher than that of hallmark Lewis acids (e.g. Cu(OTf)_2_, BF_3_·Et_2_O) usually employed in the Meyer Schuster rearrangement.^5a−5c^

Therefore, we reasoned that substoichiometric metal-oxo complex **8** could promote the Sakurai–Hosomi reaction between suitable THF acetals and allyltrimethylsilane.^5d^

In the event, the reaction of simple ethoxyl-acetal **9** (Table 1)^6^ with allyltrimethylsilane (250 mol %) in the presence of 15 mol % catalyst **8** in CH_2_Cl_2_ was initially carried out in a range of temperatures from −50 to −30 °C, but no significant trace of product was observed. Only after 30 min at room temperature, the expected products **10** (Table 1) were obtained, albeit in moderate 45% isolated yields and as a 55:45 *trans*–*cis* epimeric mixture at C-1 (Table 1, entry 1).^7^

**Table 1:**
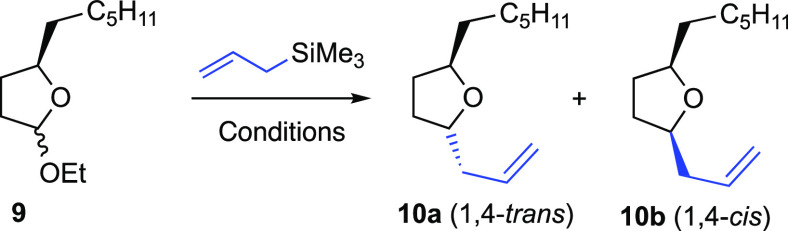
Optimization of Reaction Conditions: General Reaction Conditions: **9** (1 mmol), Allyltrimethylsilane (2.5 mmol), Oxo-Re Catalyst **8** (15 mol %), and Cocatalyst (5 mol %) Were Reacted in CH_2_Cl_2_ (10 mL) at −50 °C^a^

entry	oxo-rhenium catalyst	cocatalyst	time (h)	yield (%)
**1**^b^	Re(O)Cl_3_(OPPh_3_)(Me_2_S) **8**	none	0.5	45
**2**	Re(O)Cl_3_(OPPh_3_)(Me_2_S) **8**	Cu(OTf)_2_	12	92
**3**^c^	Re(O)Cl_3_(OPPh_3_)(Me_2_S) **8**	Cu(OTf)_2_	16	n.d.
**4**^d^	Re(O)Cl_3_(OPPh_3_)(Me_2_S) **8**	CuI	26	80
**5**	Re(O)Cl_3_(OPPh_3_)(Me_2_S) **8**	CoCl_2_	26	85
**6**^e^	none	CoCl_2_–Me_2_S	12	n.d.
**7**^e^	none	Co(OTf)_2_–Me_2_S	12	n.d.
**8**	(dppe)ReOCl_3_	none	12	n.d.
9	(dppp)ReOCl_3_	none	12	n.d.
10	(dppm)ReOCl_3_	none	12	n.d.

a Temperature was then increased to −30 °C for a given time indicated in the table.

b Without the cocatalyst and the reaction mixture was warmed to rt for 0.5 h.

c 15 mol % Cu cocatalyst.

d 30 mol % of cocatalyst.

e 5 mol % of Me_2_S, without catalyst **8**, has been used.

Substrate **9** has been used as a 1:1 mixture at C1 by virtue of the control experiment and the literature precedent that revealed that the stereoselectivity was not influenced by the anomeric composition of the starting glycoside.^8^

We speculate that this low reactivity could be attributed to the inability of acetal **9** to displace the dimethylsulfide ligand from the complex **8**, a pre-equilibrium necessary to initiate the catalytic cycle (*vide infra*). We hypothesized that a thiophilic cocatalyst would facilitate the displacement of the sulfide ligand to form the active, oxophilic 12e^–^ Re complex and by consequence improve the reaction kinetics. A brief screening of copper salts was performed, as they maintain a high affinity for sulfur.^9^ We were delighted to find that catalyst **8** (15 mol %) in the presence of Cu(OTf)_2_ (5 mol %) afforded the desired product **10** even at low temperature (−50 to −30 °C) with 92% isolated yield (Table 1, entry 2). Increasing the catalyst loading of Cu(OTf)_2_ to 15 mol %, no reaction was observed (Table 1, entry 3). Switching to a softer copper (I) salt, such as CuI,^10^ the reaction proceeded promptly but only when the catalyst loading was increased to 35 mol % of both Re catalyst **8** and CuI cocatalyst (Table 1, entry 4). This behavior could be ascribed to the stronger electrophilicity of Cu(II) compared to that of Cu(I), which causes Cu(II) to bind Me_2_S molecules more strongly. The essential role of the Me_2_S scavenging in the catalytic cycle was further demonstrated by employing CoCl_2_ instead of Cu(OTf)_2_.^11^

Indeed, allylated products **10** have been obtained in a slightly lower isolated yield (85%) using CoCl_2_ in the presence of **8** (15 mol %) (Table 1, entry 5). A background reaction promoted by the Lewis acidity of the cocatalyst was ruled out by carrying out the allylation reaction with the sole Cu(OTf)_2_ and CoCl_2_ in the presence of an equimolecular amount, with respect to the cocatalyst, of Me_2_S (Table 1, entries 6 and 7). CoCl_2_ was inactive to promote the allylation reaction, while Cu(OTf)_2_ induced an extensive decomposition of acetal **9**.

Not surprisingly, when alternative 14e^–^ Re-complexes, such as (dppm)ReOCl_3_, (dppe)ReOCl_3_, and (dppp)ReOCl_3_, were employed, no reactions were observed, even upon warming the reaction mixture at room temperature (Table 1, entries 8–10).

**Scheme 3:**
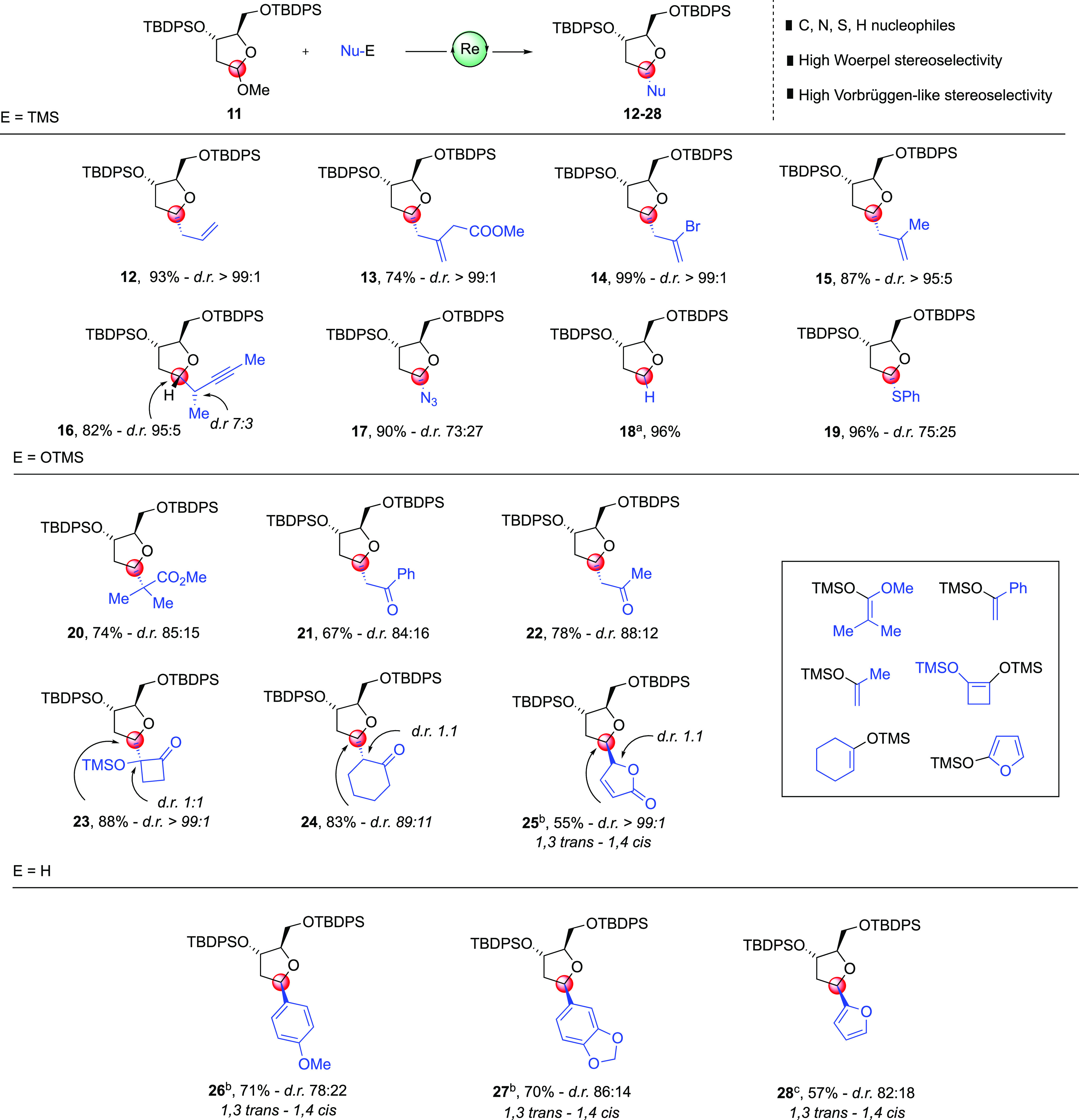
Oxo-Rhenium-Mediated Allylation of O-Methyl bis-TBDSO-Protected Furanoside Acetals with Various Nucleophiles Conditions: 1.0 equiv of furanoside **11**, 2.5 equiv of nucleophile, 15 mol % oxo-Re complex **8**, 5 mol % Cu(OTf)_2_, CH_2_Cl_2_ (0.05 M), from −50 °C to −30 °C; (a) reaction temperature was increased to 0 °C; (b) 30 mol % oxo-Re complex **8** and 15 mol % Cu(OTf)_2_ were used; and (c) 1.5 equiv of furan nucleophile was used. Unless otherwise noted, alkylated furanosides displayed a 1,3-*cis* - 1,4-*trans* stereochemistry. Diastereoselectivities, except for compound **16**, were determined from the ^1^H NMR spectrum.

With an allylation protocol in hands, we tested the versatility of our synthetic method by reacting oxo-Re catalyst **8** with different nucleophiles using (3*S*,4*R*)-bis-TBDPS-protected methoxy-acetal **11** (Scheme 3).

Allyltrimethylsilane was added to an oxocarbenium ion intermediate with an exquisite 99:1, 1,3-*syn* (1,4-*anti*) stereoselectivity with a 93% isolated yield. Relative stereochemistry of **12** has been established by chemical correlation with the known Borhan allylated diol, previously reported as diol **4**.^12^ This complete selectivity is remarkable in light of the fact that other catalytic approaches^3^ afford the corresponding TBS-protected **12** in 90:10 selectivity. Indeed, in order to achieve the same level of stereoselectivity, the reaction requires an equimolar amount of Lewis acids such as BF_3_ etherate^13^ or SnBr_4_.^14^ We could anticipate that the stereochemical outcome, for products from **13** to **24**, is in good agreement with the inside attack model proposed by Woerpel and co-workers for ribose-derived analogues.^8a,15^

The same levels of both stereoselectivities and yields have been observed using substituted allylsilanes such as 2-bromoallyltrimethylsilane, ethyl 3-(trimethylsilyl)-4-pentenoate, and methallyltrimethylsilane, delivering alkylated products **13**, **14**, and **15**, respectively. The latter was obtained with a 1,3-*syn* stereoselectivity of 95:5, a remarkable result in light of the poor selectivity obtained by Yadav for an analogous compound.^3^ The use of 2-(trimethylsilyl)-2,3-pentadiene afforded product **16**, with a pendant alkyne, in good yield and high 1,3-*syn* stereoselectivity and 7:3 (UPLC analysis) at a C-4′ (*S*)-stereocenter.^16^ Noncarbon-based nucleophiles such as nitrogen (Me_3_SiN_3_), sulfur (Me_3_SiSPh), and hydride (Et_3_SiH)^17^ afforded the corresponding azido- (**17**) and thio- (**19**) glycosides and 1,4-anhydro-2-deoxy-glycitol (**18**) in high yield and good selectivity. Other TMS-enol ether nucleophiles proved to be excellent partners in the oxocarbenium coupling; glycosylation with acetal **11** proceeded smoothly under the previous C-allylation conditions. All the reactions provided the desired *C*-glycosides (**20**–**24**) in high yields and stereoselectivity. An NOE enhancement observed by NMR analysis of **23** (see the Supporting Information for details) supported its 1,3-*cis*, 1,4-*trans* relative configurations, thus establishing the expected stereochemical Woerpel outcome. Of note, 1,3-*syn* stereoselectivity has been imposed without aid of either a directing group^18^ or by *ortho*-alkynylbenzoate glycosyl derivatives.^19^ This showcases the high atom economy of our glycosidation protocol. When vinylogous heterocyclic TMS-ether 2-(trimethylsilyloxy)furan was used,^20^ an interesting stereochemical inversion was observed. Indeed, glycoside **25** was obtained in 57% isolated yield higher than a 99:1 mixture of diastereoisomers, in which the 1,3-*trans* stereoisomer was formed rather than the 1,3-*cis* stereoisomer.^21^ The synthesis of aryl-*C*-glycosides via Lewis acid catalysis has been reported in the literature to afford primarily the β-stereoisomer (1,4-*cis* in our scenario) over the α-stereoisomer (1,4-*trans*).^22^ Albeit the origin of observed stereoselectivity has been attributed to a Lewis/Brønsted acid thermodynamic isomerization, a clear mechanistic picture is not described in the literature.^22a−22c,22e,23^ The formation of **25** therefore could be explained by an initial Friedel–Crafts reaction on the electron-rich 2-(trimethylsilyloxy)furan and then epimerization followed by furan ring dearomatization. Not surprisingly, a deviation from the Woerpel stereochemical model was observed when electron-rich, aromatic *C*-nucleophiles were subjected to our glycosidation protocol. In the event, anisole (para regioisomer only), 1,3-benzodioxole, and furan afforded the corresponding aryl-*C*-glycosides **26**, **27**, and **28**, respectively, in high yields and stereoselectivity. Vorbüggen stereoselectivity, that is, 1,3-*anti* and 1,4-*syn*, was inferred by NOE experiments (see the Supporting Information) and tentatively explained using quantum chemical calculations (*vide infra*).

**Scheme 4:**
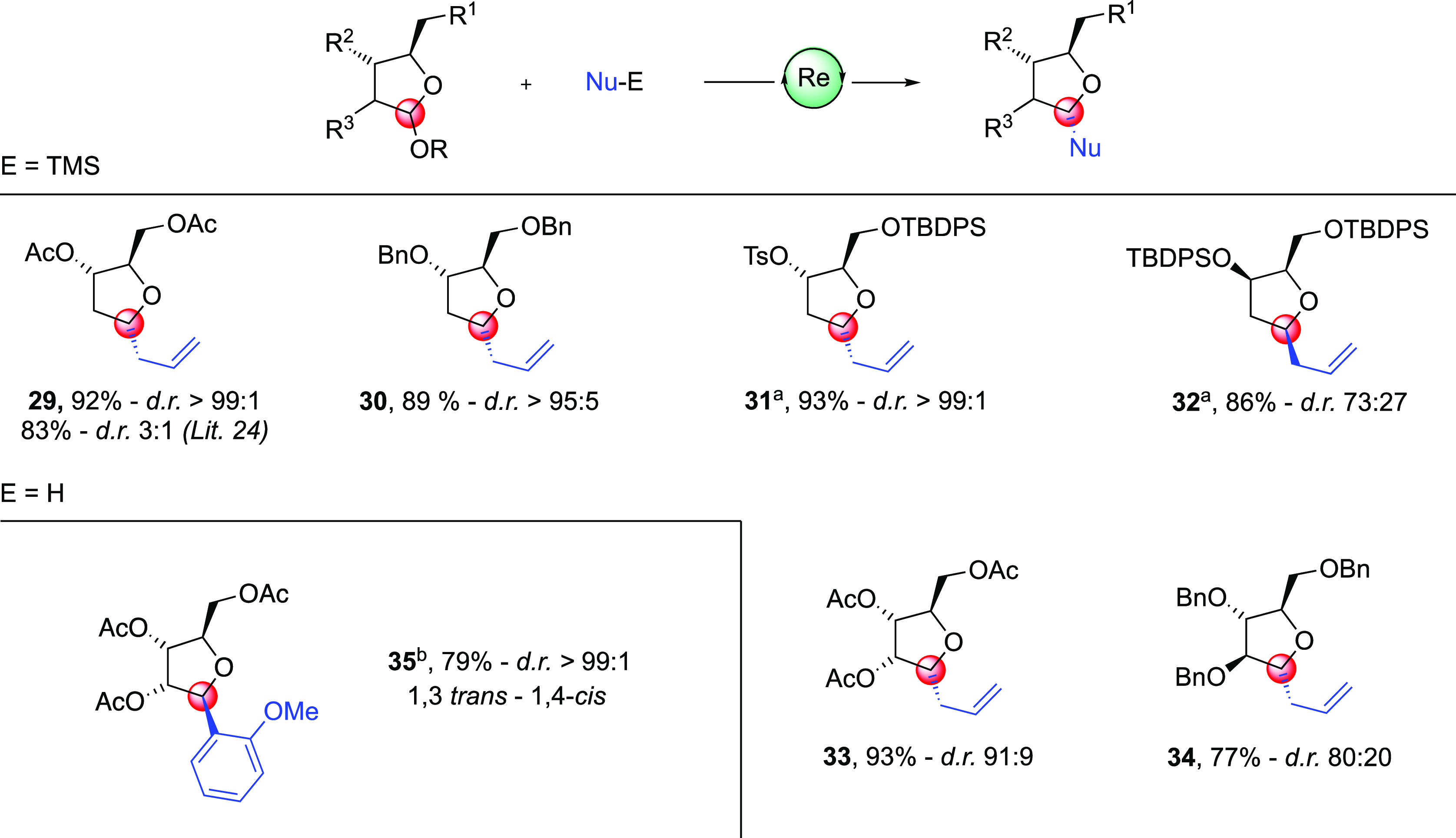
Oxo-Rhenium-Mediated Allylation Reaction on Decorated O-Alkyl Furanoside Acetals Conditions: 1 equiv of furanoside substrate, 2.5 equiv of allyltrimethyl silane, 15 mol % oxo–Re complex **8**, 5 mol % Cu(OTf)_2_, CH_2_Cl_2_ (0.05 M), from −50 to −30 °C. Unless otherwise noted, reactions were carried out on the corresponding O-methyl furanoside. (a) Reaction was carried out on the corresponding O-allyl acetal; (b) 30 mol % oxo-Re complex **8** and 15 mol % Cu(OTf)_2_ were used. Unless otherwise noted, alkylated furanosides displayed a 1,3-*cis*/1,4-*trans* stereochemistry. Diastereoselectivities were determined from the ^1^H NMR spectrum.

Next, the rhenium oxo-complex-mediated allylation procedure was next extended to more decorated and synthetically useful THF acetal substrates (Scheme 4). Acetates and benzyl ethers on carbons C-4 and C-5 and tosylate on C-4 (ribose numbering system)^1^ are well-tolerated, affording the corresponding allylated diacetate **29**, bis-benzyl ethers **30**, and tosylate **31**, in high yields and selectivities. The potential of this catalytic Re-mediated process compares favorably with those reported in the literature that typically require a stoichiometric amount of the Lewis acid trimethylsilyltrifluoromethanesulfonate (TMSOTf).^24^ The stereochemical behavior predicted by Woerpel was demonstrated by reacting the corresponding C-3 epimer of **11** (not shown) under our Re-mediated allylation reaction. Glycoside **32** was thus obtained in 86% isolated yield as a 73:27 1,3-*cis*/1,3-*trans* mixture of C-1 isomers, respectively. The stereochemical inversion could be ascribed to the pseudo-equatorial orientation—instead of the preferred pseudo-axial one—of the C-3 alkoxy group in the five-membered-ring oxocarbenium systems, as observed by Woerpel.^8a,15b^

Fully decorated THF afforded the corresponding allylated products **33** and **34** in 93% and 77% isolated yields, respectively. The 91:9 stereoselectivity in favor of the 1,3-*cis* stereoisomer of **33**, confirmed by two-dimensional NOESY experiments, is of particular importance in light of the conflicting results found in the literature and which prompted Vogel to publish an in-depth examination of the synthesis of **33** using alternative approaches.^25^ Our robust and reliable allylation protocol could therefore be used to prepare a stereochemically defined allyl-*C*-glycoside such as **33** without any doubt about the C-1 allyl stereochemistry. Finally, aryl-*C*-glycoside could be prepared on a decorated THF ring as demonstrated by the high yield and stereoselective preparation of compound **35**. This complete stereoselectivity is due to an anchimeric assistance of the acetate group at the C-2 position of the ribose, which shields the α-face of the oxocarbenium intermediate.^26a^ The regioselectivity of the Friedel–Craft reaction is in line with the data in the literature, as only the ortho product has been obtained. This is intriguing, in light of the fact that the stereoselectivity in our case is complete.^26b^

Another key feature of **31** and **32** formation lies in the nature of substituent at C-1, namely, the allyloxy group. This group could be easily ionized under our Re catalysis, to form the corresponding oxocarbenium ion. Of note, the direct use of allyloxy-protected glycosides in glycosidation reactions, for example, allows us to save the deprotection-activation sequence procedure which usually involves the use of Pd, Ir, or even Hg salts.^27^

**Scheme 5:**
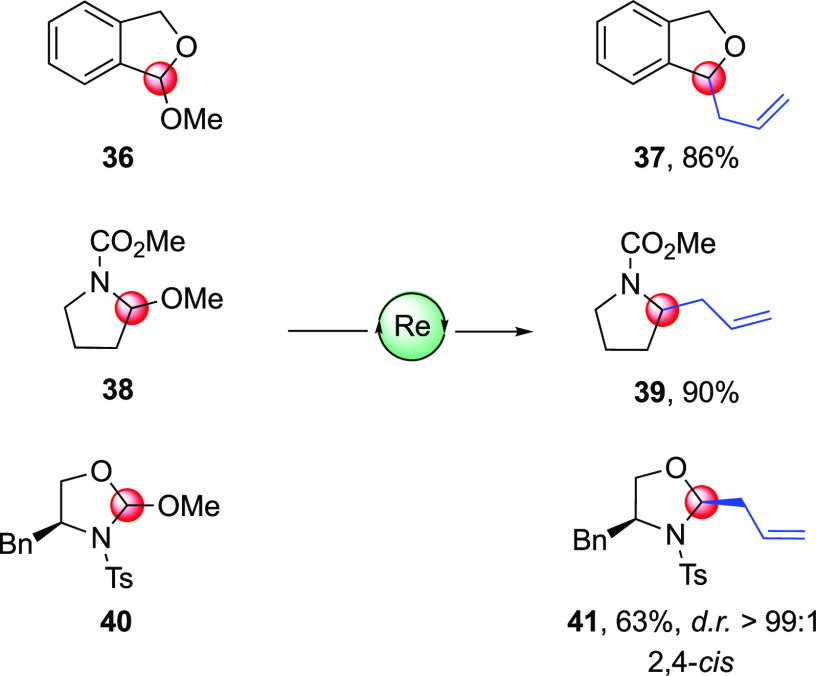
Oxo-Rhenium-Mediated Allylation Reaction on Different Oxocarbenium Ions Conditions: 1 equiv of furanoside substrate, 2.5 equiv of allyltrimethyl silane, 15 mol % oxoRe complex **8**, 5 mol % Cu(OTf)_2_, CH_2_Cl_2_ (0.05 M), from −50 to −30 °C. Diastereoselectivities were determined from the ^1^H NMR spectrum.

Dihydroisobenzofuran, pyrrolidine, and oxazolidine heterocycles could also be employed as substrates for this oxo-Re-mediated allylation (Scheme 5). In the event, 1-methoxy-1,3-dihydroisobenzofuran **36** and 2-methoxy-1-methoxycarbonylpyrrolidine **38**(28) afforded the corresponding allylated product **37** and **39** in 86 and 90% isolated yields, respectively. Interestingly, when chiral^29a^ (4*S*)-4-benzyl-2-methoxy-3-tosyl-1,3-oxazolidine **40** was subjected to the allylation reaction, the corresponding 2,4-*cis* diastereoisomer **41** was obtained in more than a 99:1 ratio in 63% isolated yield. According to the data available in the literature, stereoisomer **41** corresponds to the thermodynamic product.^29b^

## Computational Studies

To shed some light on the peculiar stereochemical behavior of aryl-*C*-glycoside formation, some computational density functional theory (DFT) calculations have been carried out.

**Scheme 6:**
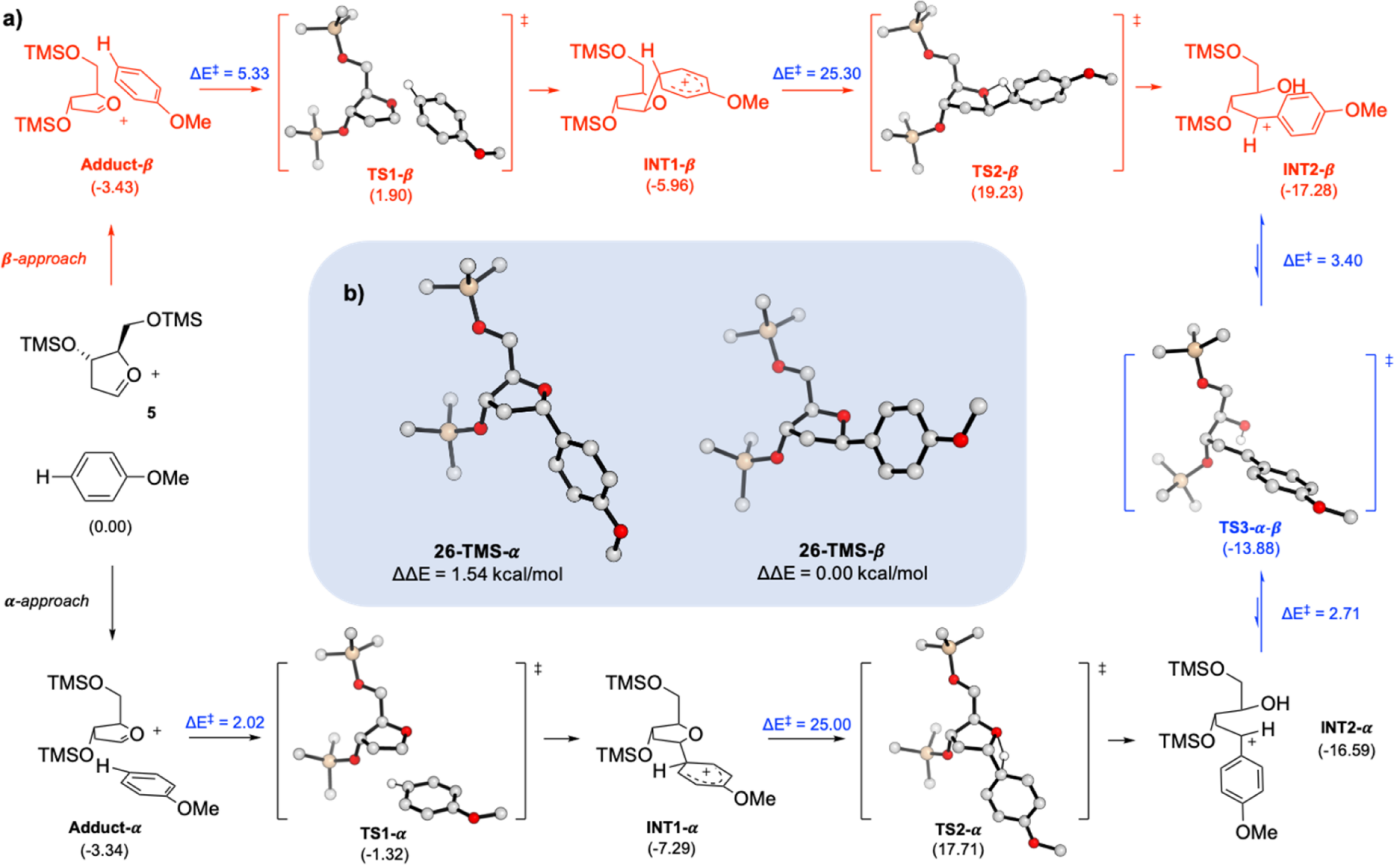
(a) Proposed Mechanism for the Aryl-*C*-glycoside Formation Based on DFT Calculations (Δ*E* in kcal/mol); (b) Relative Energy between Products **26-TMS-β** and **26-TMS-α** [B3LYP/6-31G(d,p)&6-31+G(d,p)/CH_2_Cl_2_(PCM)//B3LYP/6-31G(d,p)&6-31+G(d,p)]

The reaction starts with the initial approach of the anisole reactant toward the α- or β-face of the oxocarbenium species **5**, by forming two quasi-isoenergetic adducts, **adduct-α** and **adduct-β**, respectively (Scheme 6a). The system undergoes the Friedel–Crafts reaction passing through the attachment to the **α** side, a largely favored (ΔΔ*E* = 3.22 kcal/mol) approach over the **β** one, as indicated by the energy of the **TS1-α** and **TS1-β** transition states (TSs), −1.32 and 1.90 kcal/mol, respectively.

After the first TS, the two next Wheland intermediates are reported as **INT1-α** and **INT1-β**, which maintain the same distribution in selectivity as in the previous TSs.

The results obtained so far, seemed to be in disagreement with the experimental data: literature methods for C-1 epimerization of the furanoside derivative require harsh conditions in order to be accomplished. Strong acids, such as benzenesulfonic acid or TFA, in refluxing toluene^22g^ or in over stoichiometric concentrations are usually required.^23b^ Under these conditions, the formation of a carbocation intermediate has been demonstrated by NMR studies.^23b^ However, in our case, the equilibration occurred at −30 °C in the presence of a catalytic amount of a Lewis acid; therefore, a different scenario should be operative.

Specifically, **INT1** species could undergo an intramolecular hydrogen transfer of the two corresponding Wheland cationic intermediates to the furan oxygen atom and the concomitant opening of the THF ring. The TSs obtained for this transformation appear to be the rate-determining step in both the cases, with an activation energy of 25.00 kcal/mol to overcome **TS2-α** and 25.30 kcal/mol for **TS2-β**. The resulting intermediates **INT2** correspond to the opened-ring cationic structures, where the alcohol obtained from the protonation of the furane oxygen is opposed to the trigonal planar cationic counterpart (see Scheme 6a). Also, in this step, the α-approach is favored over the β-one. These two transition states are fundamental to explain the reactivity of this reaction: indeed, the population of **INT2-α** as a main intermediate occurs under a kinetic-dominated scenario. Furthermore, **INT2-α** can be easily interconverted in the more stable **INT2-β** through **TS3-α-β** with a very low energy barrier (2.71 kcal/mol) under thermodynamic equilibration. This negligible C–C bond energy rotation is the key step for the equilibration between the two C-1 epimers, thus resulting in the formation of the β-arylated product as a main isomer. By applying a Boltzmann population analysis of the direct (**α** to **β**, Δ*E* = 2.71 kcal/mol) and reverse (**β** to **α**, Δ*E* = 3.40 kcal/mol) reactions at the equilibrium, we found that theoretical values of 76:24 (**α**/**β**) are fully consistent with the selectivity trend observed in our experiments, 78:22 (**α**/**β**) (Scheme 6a).

In Scheme 6b, the structures of the final deprotonated products **26-TMS-α** and **26-TMS-β** are reported together with the relative energy, showing the higher stability of the **β** product over the **α** one (ΔΔ*E* = 1.54 kcal/mol).

## Conclusions

In summary, a new methodology for the formation of oxocarbenium ions and subsequent addition of a wide range of nucleophiles was developed, exploiting the advantage of using commercially available THF *O*-methoxy-acetals as starting materials in combination with a Re (V) complex catalyst. The peculiarity of this method is the use of catalytic amounts of a stable and easy to handle Re (V) catalyst as a Lewis acid, together with the Cu(OTf)_2_ cocatalyst, achieving high yields and stereoselectivities. The wide scope for both the nucleophile and the oxocarbenium species paves the way for further investigation in this research field, also opening to a fast and reliable approach for other heterocyclic substrates. Moreover, the computational explanation of the diastereoselectivity observed in the case of electron-rich aromatic nucleophiles uncovered the presence of an equilibration step, which is ultimately responsible for the regulation of the observed *anti*-Woerpel selectivity.

## Experimental Section

### General Methods

All the reactions were performed in glassware which has been dried in an oven at 140 °C for at least 3 h prior to use and allowed to cool in a desiccator over self-indicating silica gel pellets. Anhydrous solvents were distilled from appropriate drying agents. Allyltrimethylsilane, anisole, methyl trimethylsilyl dimethylketene acetal, γ-decalactone, 1-*O*-methyl-2-deoxy-*d*-ribose, 2-deoxy-d-ribose, methyl d-ribofuranoside, methyl d-arabinofuranoside, methyl 3,5-di-*O*-acetyl-2-deoxy-*d*-ribofuranoside **S29**, 1-methoxy-1,3-dihydro-2-benzofuran **36**, 2-methoxy-pyrrolidine-1-carboxylic acid methylester **38**, and complex ReOCl_3_(OPPh_3_)(SMe_2_) **8** were purchased and used as received. The reactions were carried out under a slightly positive static pressure of argon. Routine monitoring of reactions was performed using GF- 254 Merck (0.25 mm) aluminum-supported TLC plates. Compounds were visualized by UV irradiation at a wavelength of 254 nm or stained by exposure to a 0.5% solution of vanillin in H_2_SO_4_–EtOH followed by charring. Flash column chromatography was performed using Kieselgel 60 Merck (40–63 μm). Yields are reported for chromatographically and spectroscopically pure isolated compounds. NMR spectra were recorded on 200, 300, and 400 MHz spectrometers. Chemical shifts are reported in δ units relative to the employed solvent; the main abbreviations used have the following meaning: s = singlet, d = doublet, t = triplet, q = quartet, qu = quintuplet, m = multiplet, and br = broad. Coupling constants (*J*) are given in Hz. The multiplicity (in parentheses) of each carbon atom was determined by DEPT experiments, while * indicates the presence of anomers. A relative configuration of the anomeric center of compounds **Ba-*trans*** and **Bb-*cis*** was determined with NOE experiments.

### Synthesis of Acetals

#### (5*R*)-2-Ethoxy-5-hexyltetrahydrofuran (**9**)

DIBAL-H (2.50 mL, 1 M solution in hexane, 2.50 mmol, 1.2 equiv) was added dropwise to a solution of γ-decalactone (0.35 g, 2.10 mmol, 1.0 equiv) in dry CH_2_Cl_2_ (13 mL) at −78 °C under an argon atmosphere. After been stirred for 30 min, the mixture was quenched by adding a saturated solution of NH_4_Cl (50 mL). CH_2_Cl_2_ (80 mL) was then added, and the resulting mixture was warmed to rt before the addition of saturated solution of Rochelle’s salt. The layers were separated, and the aqueous phase was extracted with CH_2_Cl_2_ (4 × 10 mL). The combined organic phases were washed with brine, dried with MgSO_4_, filtered, and concentrated under reduced pressure. The resulting residue was dissolved in CH_2_Cl_2_/EtOH (2:1, 24 mL) and cooled to 4 °C, and then, PTSA (cat.) was added. After 12 h, excess solid NaHCO_3_ was added, and the resulting mixture was stirred for 15′, filtered, and concentrated under reduced pressure. The residue was dissolved in CH_2_Cl_2_ (50 mL), and a saturated solution of NaHCO_3_ (50 mL) was added. The layers were separated, and the aqueous phase was extracted with CH_2_Cl_2_ (3 × 20 mL). The combined organic phases were dried with Na_2_SO_4_, filtered, and concentrated under reduced pressure. The resulting residue was purified by flash chromatography on silica gel. Elution with hexane/EtOAc (98:2) gave compound **9** (386 mg, 92%), colorless oil as a 1:1 mixture of unseparable anomers; ^1^H NMR (300 MHz, CD_2_Cl_2_): δ 5.10 (dd, *J* = 4.9, 2.0 Hz, 1H), 5.03 (dd, *J* = 3.7, 1.5 Hz, 1 H), 4.01 (m, 1H), 3.71 (m, 1H), 3.43 (dq, *J* = 16.1, 8.6 Hz, 1H), 2.04–1.33 (m, 14H), 1.12 (t, *J* = 7.1 Hz, 3H), 0.92 (t, *J* = 6.9 Hz, 3H) ppm; ^13^C{^1^H} NMR (75 MHz, CD_2_Cl_2_): δ 104.4 (CH), 104.1* (CH), 81.2 (CH), 78.6 (CH), 63.2 (CH_2_), 62.7* (CH_2_), 38.5 (CH_2_), 36.4* (CH_2_), 34.0 (CH_2_), 33.1 (CH_2_), 32.7* (CH_2_), 30.3 (CH_2_), 30.2* (CH_2_), 27.1 (CH_2_), 26.9 (CH_2_), 23.4 (CH_2_), 15.9 (CH_3_), 14.7 (CH_3_) ppm; IR (film, cm^–1^): 2980, 1464, 1369, 1107, 739; HRMS (ESI) *m*/*z*: [M + H]^+^ calcd. for C_12_H_25_O_2_, 201.1849; found, 201.1852.

#### *tert*-Butyl(((2R,3S)-3-((tert-butyldiphenylsilyl)oxy)-5-methoxytetrahydrofuran-2-yl)methoxy)diphenylsilane (**11**)


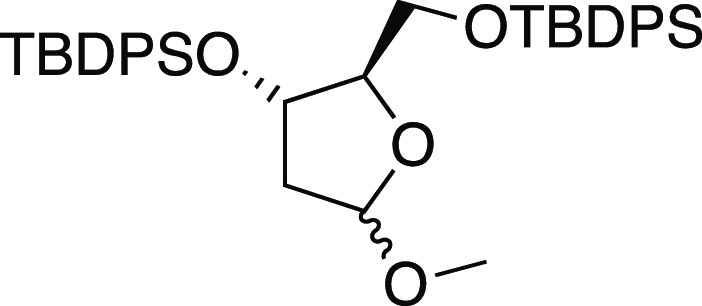
The commercially available 1-*O*-methyl-2-deoxy-d-ribose (1000 mg, 6.75 mmol) was dissolved in dry CH_2_Cl_2_ (67.5 mL) at room temperature under an argon atmosphere; then, imidazole (1379 mg, 20.25 mmol, 3 equiv) and TBDPSCl (3.80 mL, 14.85 mmol, 2.2 equiv) were added under magnetic stirring. The resulting solution has been stirred for 10 h and then was quenched by adding a saturated solution of NaHCO_3_ (50 mL). The layers were separated, and the aqueous phase was extracted with CH_2_Cl_2_ (3 × 50 mL). The combined organic phases were dried with Na_2_SO_4_, filtered, and concentrated under reduced pressure. The resulting residue was purified by flash chromatography on silica gel. Elution with hexane/EtOAc (99:1) gave compound **11** (3922 mg, 95%), colorless oil as a 1:1 mixture of unseparable anomers. The spectroscopic data correspond with those reported in the literature (See the Supporting Information for ^1^H NMR spectra).^30^

#### (2*R*,3*S*,5*S*)-5-(Allyloxy)-2-(((*tert*-butyldiphenylsilyl)oxy)methyl)tetrahydrofuran-3-ol (**Ba**) and (2*R*,3*S*,5*R*)-5-(allyloxy)-2-(((*tert*-butyldiphenylsilyl)oxy)methyl)tetrahydrofuran-3-ol (**Bb**)


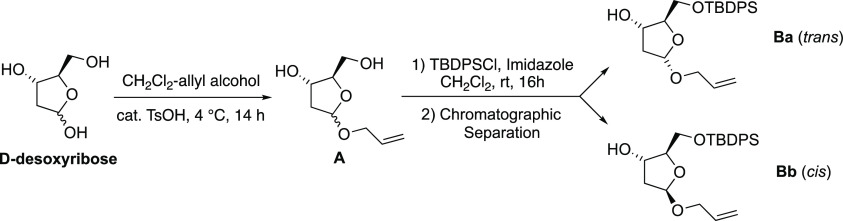
2-Deoxy-d-ribose (134.1 mg, 1.0 mmol) was dissolved in CH_2_Cl_2_/2-propen-1-ol (15:1, 16 mL) and cooled to 4 °C, and then, PTSA (cat.) was added. After 14 h, excess solid NaHCO_3_ was added, and the resulting mixture was stirred for 15′ and concentrated under reduced pressure. The residue was dissolved in CH_2_Cl_2_ (50 mL), and a saturated solution of NaHCO_3_ (50 mL) was added. The layers were separated, and the aqueous phase was extracted with CH_2_Cl_2_ (3 × 20 mL). The combined organic phases were dried with Na_2_SO_4_, filtered, and concentrated under reduced pressure. The resulting residue was purified by flash chromatography on silica gel. Elution with hexane–EtOAc (2:8) gave a desired allyl acetal **A** as a 1:1 mixture of unseparable anomers. Allyl acetal **A** (120.1 mg, 0.7 mmol) was dissolved in dry CH_2_Cl_2_ (6 mL) at room temperature under an argon atmosphere; then, imidazole (120.1 mg, 1.7 mmol, 3.0 equiv) and TBDPSCl (0.16 mL, 0.61 mmol, 1.1 equiv) were added under magnetic stirring. The resulting solution has been stirred for 16 h and then was quenched by adding a saturated solution of NaHCO_3_ (4 mL). The layers were separated, and the aqueous phase was extracted with CH_2_Cl_2_ (3 × 8 mL). The combined organic phases were dried with Na_2_SO_4_, filtered, and concentrated under reduced pressure. The resulting residue was purified by flash chromatography on silica gel. Elution with a gradient from hexane/MTBE (99:1) to hexane/MTBE (9:1) gave compound **Ba-*trans*** (139 mg) and compound **Bb-*cis*** (88.1 mg), colorless oil, overall yield = 96%, *trans*/*cis* ratio = 1.58/1;

##### (**Ba**)-***trans***

^1^H NMR (300 MHz, CD_2_Cl_2_): δ 7.70 (m, 4H), 7.40 (m, 6H), 5.85 (m, 1H), 5.35–5.15 (m, 3H), 4.28 (dd, *J* = 13.0, 5.1 Hz, 2H), 4.18 (t, *J* = 4.9 Hz, 1H), 4.02 (dd, *J* = 13.0, 5.9 Hz, 1H), 3.80 (dd, *J* = 10.9, 3.8 Hz, 1H), 3.68 (dd, *J* = 10.9, 4.8 Hz, 1H), 2.80 (dd, *J* = 10.6, 1.9 Hz, 1H), 2.25 (ddd, *J* = 13.6, 6.1, 4.7 Hz, 1H), 1.05 (s, 9H); ^13^C{^1^H} NMR (75 MHz, CD_2_Cl_2_): δ 136.5 (CH), 135.5 (CH), 134.2 (C), 130.6 (CH), 134.6 (CH), 128.6 (CH), 117.3 (), 104.8 (CH), 88.8 (CH), 73.9 (CH), 68.9 (CH_2_), 65.4 (CH_2_), 42.1 (CH_2_), 27.5 (C), 20.0 (C); IR (film, cm^–1^): 2933, 1564, 1472, 1432, 1115; HRMS (ESI) *m*/*z*: [M + H]^+^ calcd. for C_24_H_33_O_4_Si, 413.2143; found, 413.2139; [α]_D_^20^ + 58.0, *c* = 0.96 in CH_2_Cl_2_.

##### (Bb)-*cis*

^1^H NMR (300 MHz, CD_2_Cl_2_): δ 7.75 (m, 4H), 7.40 (m, 6H), 5.85 (m, 1H), 5.25–5.05 (m, 3H), 4.50 (dt, *J* = 6.3, 4.2 Hz, 1H), 4.15 (dd, *J* = 13.0, 5.0 Hz, 1H), 3.90 (m,1H), 3.80 (dd, *J* = 10.3, 5.4 Hz, 1H), 3.70 (dd, *J* = 10.3, 7.3 Hz, 1H), 2.24 (ddd, *J* = 13.4, 6.7, 2.3 Hz, 1H), 2.08 (m, 1H), 1.05 (s, 9H); ^13^C{^1^H} NMR (75 MHz, CD_2_Cl_2_): δ 136.3 (CH), 135.5 (CH), 134.2 (CH), 130.5 (C), 130.4 (C), 128.5 (CH), 128.4 (CH), 116.9 (CH_2_), 104.1 (CH), 86.9 (CH), 73.9 (CH), 69.0 (CH_2_), 66.3 (CH_2_), 42.1 (CH_2_), 27.4 (CH_3_), 19.9 (C); IR (film, cm^–1^): 2931, 1568, 1475, 1431, 1118; HRMS (ESI) *m*/*z*: [M + H]^+^ calcd. for C_24_H_33_O_4_Si, 413.2143; found, 413.2139; [α]_D_^20^ = −41.7, *c* = 0.94 CH_2_Cl_2_.

#### (2*R*,3*S*)-3-(benzyloxy)-2-((benzyloxy)methyl)-5-methoxytetrahydrofuran (**S30**)


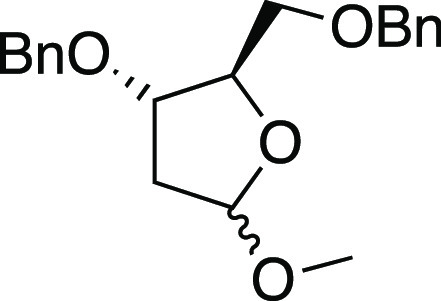
The commercially available 1-*O*-methyl-2-deoxy-d-ribose (200 mg, 1.35 mmol) was dissolved in dry DMF (6.75 mL) at room temperature under an argon atmosphere. Then, the solution was cooled down to 0 °C, and NaH (71 mg, 2.97 mmol, 2.2 equiv) was added under magnetic stirring. The resulting solution was stirred for 30 min, and then, benzyl-bromide (481 μL, 4.05 mmol, 3 equiv) was added dropwise. The so-obtained mixture was then warmed to room temperature and left under stirring overnight. The quench was operated by the addition of a saturated solution of NH_4_Cl (10 mL). The layers were separated, and the aqueous phase was extracted with Et_2_O (3 × 10 mL). The combined organic phases were dried with Na_2_SO_4_, filtered, and concentrated under reduced pressure. The resulting residue was purified by flash chromatography on silica gel. Elution with hexane/EtOAc (9:1) gave compound **S30** (353 mg, 80%), colorless oil as a 1:1 mixture of unseparable anomers. The spectroscopic data correspond with those reported in the literature (see the Supporting Information for ^1^H NMR spectra).^31^

#### (2*R*,3*S*,5*S*)-5-(Allyloxy)-2-(((tert-butyldiphenylsilyl)oxy)methyl)tetrahydrofuran-3-yl 4-methylbenzenesulfonate (**S31**)


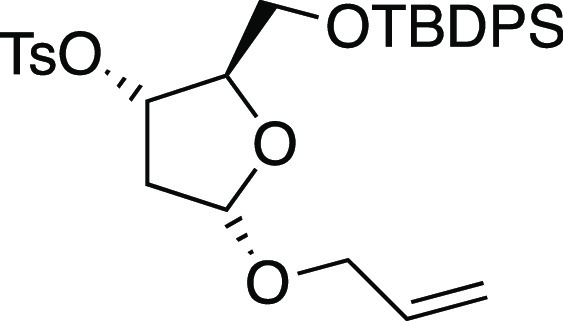
Free alcohol **Ba** (100.1 mg, 0.24 mmol) was dissolved in dry pyridine (4.0 mL) at room temperature under an argon atmosphere; then, tosyl chloride (185.0 mg, 0.96 mmol, 4.0 equiv) and 4-dimethylaminopyridine (2.9 mg, 0.02 mmol, 0.1 equiv) were added under magnetic stirring. The resulting solution has been stirred for 15 h and then was quenched by adding a saturated solution of NaHCO_3_ (5 mL). The layers were separated, and the aqueous phase was extracted with Et_2_O (3 × 5 mL). The combined organic phases were dried with Na_2_SO_4_, filtered, and concentrated under reduced pressure. The resulting residue was purified by flash chromatography on silica gel. Elution with hexane/AcOEt (9:1) gave compound **S31** (122.3 mg, 90%) as colorless oil; ^1^H NMR (300 MHz, CD_3_CN): δ 7.75 (d, *J* = 8.3 Hz, 2H), 7.61 (m, 4H), 7.50–7.40 (m, 8H), 6.05–5.85 (m, 1H), 5.32–5.12 (m, 3H), 5.02 (dt, *J* = 7.6, 1.5 Hz, 1H), 4.25–4.15 (m, 2H), 4.05–3.91 (m, 1H), 3.60–3.48 (dd, *J* = 11.3, 3.0 Hz, 2H), 2.45 (s, 3H), 2.25-2.37-1.90 (m, 2H), 1.08 (s, 9H) ppm; ^13^C{^1^H} NMR (75 MHz, CD_3_CN): δ 145.5 (C), 135.4 (CH), 135.3 (CH), 134.8 (CH), 133.0 (C), 132.9 (C), 130.1 (CH), 129.9 (CH), 129.9 (CH), 127.8 (CH), 127.7 (CH), 115.9 (CH_2_), 103.2 (CH), 83.5 (CH), 80.6 (CH), 67.9 (CH_2_), 63.2 (CH_2_), 39.2 (CH_2_), 26.1 (CH_3_), 20.7 (CH), 18.7 (C) ppm; IR (film, cm^–1^): 3068, 2855, 1471, 1424, 1261, 1102, 1004, 940, 713; HRMS (ESI) *m*/*z*: [M + H]^+^ calcd. for C_31_H_39_O_6_SSi, 567.2231; found, 567.2233; [α]_D_^20^ = +65.3, *c* = 2.72 CH_2_Cl_2_.

#### Synthetical Sequence for the Preparation of Inverted Acetal **S32**

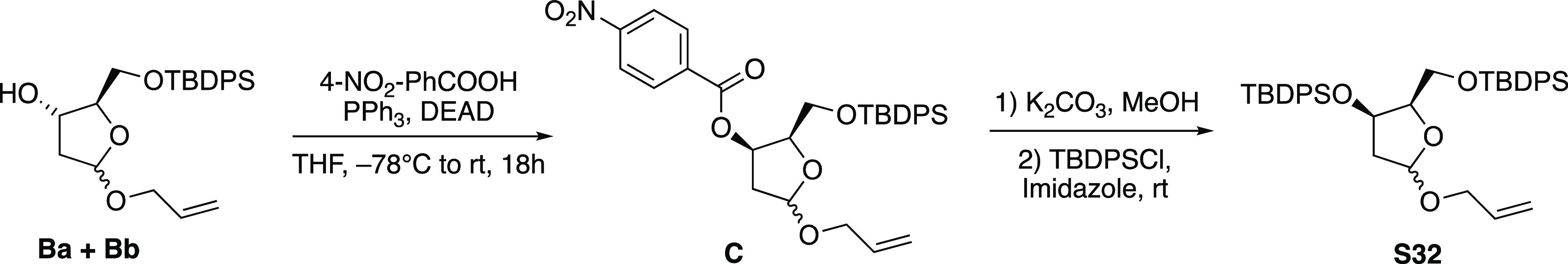



##### (2*R*,3*R*)-5-(Allyloxy)-2-(((tert-butyldiphenylsilyl)oxy)methyl)tetrahydrofuran-3-yl 4-nitrobenzoate (**C**)


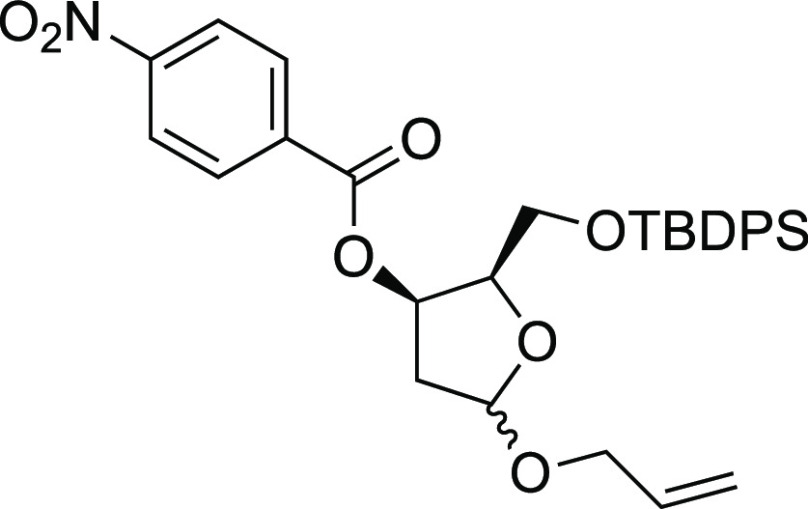
To a magnetically stirred solution of anomeric mixture of **Ba** and **Bb** (80.9 mg, 0.196 mmol) in dry toluene (2 mL) under an Ar atmosphere at −78 °C were added PPh_3_ (66.8 mg, 0.255 mmol, 1.3 equiv), 4-NO_2_-C_6_H_4_-CO_2_H (36.1 mg, 0.216 mmol, 1.1 equiv), and DEAD (41 mL, 0.262 mmol). The reaction mixture was allowed to warm to room temperature, and the reaction was completed after 16 h. The reaction mixture was thus concentrated under vacuum. The residue was purified by column chromatography on silica gel using hexane/AcOEt 92:8 as an eluent to afford the ester **C** as white foam (97.9 mg, yield = 89%); ^1^H NMR (300 MHz, CDCl_3_): δ 8.40–8.09 (m, 4H), 7.75–7.66 (m, 3H), 7.65–7.57 (m, 2H), 7.51–7.25 (m, 8H), 5.96 (dddd, *J* = 17.3, 10.6, 5.8, 5.0 Hz, 1H), 5.82 (dt, *J* = 5.3, 3.7 Hz, 1H), 5.41 (dd, *J* = 5.0, 3.7 Hz, 1H), 5.29 (dq, *J* = 17.3, 1.8 Hz, 1H), 5.15 (dq, *J* = 10.5, 1.6 Hz, 1H), 4.46 (td, *J* = 6.2, 4.0 Hz, 1H), 4.23 (ddt, *J* = 13.2, 5.0, 1.6 Hz, 1H), 4.10–3.91 (m, 4H), 2.50–2.40 (m, 2H), 1.00 (s, 9H); ^13^C{^1^H} NMR (75 MHz, CDCl_3_): δ 164.9 (C), 151.9 (C), 136.7 (C), 136.6 (C), 136.3 (C), 134.4 (C), 131.9 (CH), 131.1 (CH), 131.0 (CH), 129.0 (CH), 128.9 (CH), 124.8 (C), 116.9 (CH_2_), 103.7 (CH), 80.6 (CH), 76.3 (CH), 69.6 (CH_2_), 63.0 (CH_2_), 41.4 (CH_2_), 27.41 (CH_3_), 20.01 (C); IR (film, cm^–1^): 3071, 2932, 2857, 1719, 1529, 1479, 1429, 1281, 1103, 1003, 935; HRMS (ESI) *m*/*z*: [M + H]^+^ calcd. for C_31_H_36_NO_7_Si_2_, 562.2256; found, 562.2253.

##### (2*R*,3*R*)-5-(Allyloxy)-2-(((*tert*-butyldiphenylsilyl)oxy)methyl)tetrahydrofuran-3-ol (**D**)


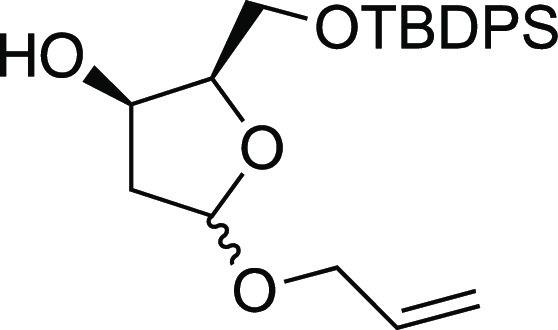
To a magnetically stirred solution of **C** (236 mg, 0.42 mmol) in dry MeOH (8 mL) were added few crystals of K_2_CO_3_. The reaction mixture was stirred at room temperature, and the reaction was completed after 16 h. Methanol was removed in vacuo, and the residue was purified on silica gel using hexane/AcOEt 80:20 as an eluent to afford the desired alcohol **D** as colorless oil (164.6 mg, yield = 95%); ^1^H NMR (300 MHz, (CD_3_)_2_CO): δ 7.86–7.67 (m, 4H), 7.56–7.36 (m, 6H), 5.95 (ddt, *J* = 17.1, 10.6, 5.4 Hz, 1H), 5.39–5.02 (m, 3H), 4.48 (p, *J* = 4.4 Hz, 1H), 4.28–3.78 (m, 5H), 2.14 (t, *J* = 4.4 Hz, 2H), 1.07 (s, 9H); ^13^C{^1^H} NMR (75 MHz, (CD_3_)_2_CO): δ 136.9 (CH), 136.8 (CH), 136.7 (CH), 136.7 (CH), 134.9 (C), 134.8 (C), 130.9 (CH), 129.0 (CH), 128.9 (CH), 116.5 (CH_2_), 103.9 (CH), 82.80 (CH), 72.21 (CH), 69.3 (CH_2_), 64.3 (CH_2_), 44.1 (CH_2_), 27.7 (CH_3_), 27.5 (CH_3_), 20.1 (C); IR (film, cm^–1^): 2934, 1566, 1475, 1431, 1117; HRMS (ESI) *m*/*z*: [M + H]^+^ calcd. for C_24_H_33_O_4_Si, 413.2143; found, 413.2145.

#### (((2*R*,3*R*)-5-(Allyloxy)-2-(((*tert*-butyldiphenylsilyl)oxy)methyl)tetrahydrofuran-3-yl)oxy) (*tert*-butyl)diphenylsilane (**S32**)


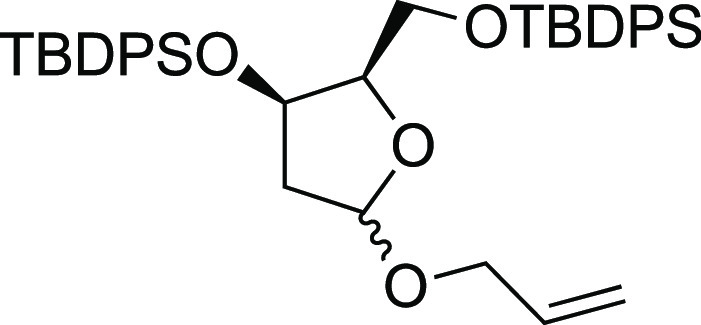
The epimeric alcohol **D** (42 mg, 0.1 mmol) was dissolved in dry CH_2_Cl_2_ (2 mL) under Ar, and at room temperature, imidazole (28 mg, 0.41 mmol, 4.1 equiv) and TBDPSCl (32 mL, 0.11 mmol, 1.1 equiv) were added. The reaction mixture was stirred at room temperature for 16 h and was quenched with H_2_O (2 mL). The layers were separated, and the aqueous layer was extracted with CH_2_Cl_2_ (3 × 5 mL). The combined organic layers were dried over Na_2_SO_4_, filtered, and concentrated under vacuum. The residue was purified by column chromatography on silica gel using hexane/MTBE 95:5 as an eluent to afford the product **S32** as colorless oil (59.9 mg, yield = 92%); ^1^H NMR (300 MHz, (CD_3_)_2_CO): δ 7.77 (ddt, *J* = 12.8, 6.3, 1.8 Hz, 4H), 7.64 (ddt, *J* = 6.6, 3.7, 1.9 Hz, 4H), 7.52–7.32 (m, 12H), 6.00–5.76 (m, 1H), 5.28–5.02 (m, 3H), 4.65 (dt, *J* = 6.0, 3.9 Hz, 1H), 4.20–3.88 (m, 5H), 2.15–2.01 (m, H, overlapped with solvent), 1.85 (ddd, *J* = 13.8, 6.2, 2.8 Hz, 1H), 1.10 (s, 9H), 1.00 (s, 9H) ppm; ^13^C{^1^H} NMR (75 MHz, (CD_3_)_2_CO): δ 136.9 (CH), 136.8 (CH), 136.8 (CH), 136.7 (CH), 136.7 (CH), 136.5 (CH), 135.1 (C), 135.0 (C), 134.9 (C), 134.4 (C), 131.1 (CH), 131.1 (CH), 130.9 (CH), 129.1 (CH), 128.9 (CH), 128.9 (CH), 116.6 (CH_2_), 103.5 (CH), 82.9 (CH), 74.6 (CH), 69.2 (CH_2_), 65.0 (CH_2_), 43.7 (CH_2_), 27.7 (CH_3_), 27.6 (CH_3_), 20.2 (C), 20.1 (C) ppm; IR (film, cm^–1^): 3065, 2858, 1470, 1421, 1257, 1106, 1002, 941, 751, 713, 601; HRMS (ESI) *m*/*z*: [M + H]^+^ calcd. for C_40_H_51_O_4_Si_2_, 651.3320; found, 651.3322.

#### (2*R*,3*R*,4*R*)-2-(Acetoxymethyl)-5-methoxytetrahydrofuran-3,4-diyl diacetate (**S33**)


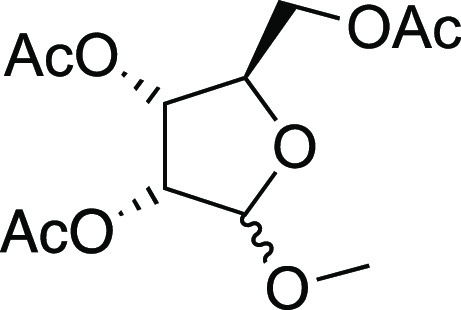
The commercially available methyl d-ribofuranoside (90.7 mg, 0.55 mmol) was dissolved in dry CH_2_Cl_2_ (8 mL) at room temperature under an argon atmosphere; then, pyridine (0.35 μL, 3.92 mmol, 7.1 equiv) and Ac_2_O (0.20 μL, 1.93 mmol, 3.5 equiv) were added under magnetic stirring. The resulting solution has been stirred for 15 h and then was quenched by adding a saturated solution of NaHCO_3_ (5 mL). The layers were separated, and the aqueous phase was extracted with CH_2_Cl_2_ (3 × 5 mL). The combined organic phases were dried with Na_2_SO_4_, filtered, and concentrated under reduced pressure. The resulting residue was purified by flash chromatography on silica gel. Elution with hexane/MTBE (9:1) gave compound **S33** (145.3 mg, 91%) as colorless oil as a mixture of unseparable anomers. The spectroscopic data correspond with those reported in the literature (See the Supporting Information for ^1^H NMR spectra).^32^

#### (2*R*,3*R*,4*S*)-3,4-bis(benzyloxy)-2-((benzyloxy)methyl)-5-methoxytetrahydrofuran (**S34**)


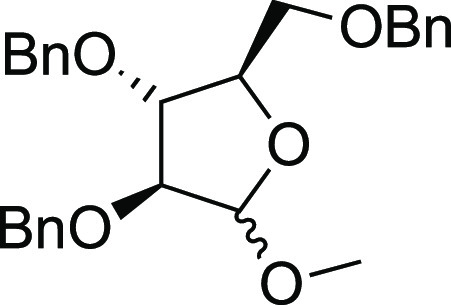
The commercially available methyl d-arabinofuranoside was involved in the same procedure abovereported for the synthesis of compound **S30**, by giving compound **S34** in 85% of isolated yield as colorless oil as a mixture of unseparable anomers. The spectroscopic data correspond with those reported in the literature (see the Supporting Information for ^1^H NMR spectra).^33^

### General Procedure for Allylation with Allyl-TMS

A suitable acetal (0.08 mmol) was dissolved in dry CH_2_Cl_2_ (30 mM) at room temperature under an argon atmosphere; then, allyltrimethylsilane (0.40 mmol, 5.0 equiv or less, see manuscript for details) was added under magnetic stirring. The resulting solution was cooled at −50 °C, and complex **8** (15 mol %) followed by Cu(OTf)_2_ (5 mol %) were added in one portion. The reaction mixture was allowed to warm at −30 °C and stirred at this temperature until the starting material was completely consumed. The reaction was then quenched with a saturated solution of NaHCO_3_ (2 mL). The layers were separated, and the aqueous phase was extracted with CH_2_Cl_2_ (3 × 4 mL). The combined organic phases were dried with Na_2_SO_4_, filtered, and concentrated under reduced pressure. The resulting residue was purified by flash chromatography on silica gel. Elution with a suitable solvent mixture (see conditions for each compound) gave the final product (see yield for each compound).

#### (2*S*,5*R*)-2-Allyl-5-hexyltetrahydrofuran (**10a**) and (2*R*,5*R*)-2-allyl-5-hexyltetrahydrofuran (**10b**)

See Table 1 for yields associated to each reaction conditions; for entry 2, purification with hexane/MTBE (99:1) gave 186.2 mg, 95%, as colorless oil; ^1^H NMR (300 MHz, CDCl_3_): δ 5.82 (ddt, *J* = 10.1, 2.7, 0.9 Hz, 1H), 5.12 (dd, *J* = 2.1, 0.9 Hz, 1H), 5.04 (dd, *J* = 2.7, 0.9 Hz, 1H), 3.95 (dt, *J* = 32.0, 7.4 Hz, 1H), 3.89 (dt, *J* = 43.3, 6.2 Hz, 1H), 2.37 (m, 1H), 2.24 (m, 1H), 1.99 (m, 2H), 1.59–1.29 (m, 12H), 0.89 (t, *J* = 6.9 Hz, 3H) ppm; ^13^C{^1^H} NMR (75 MHz, CDCl_3_): δ 134.6 (CH), 116.1 (CH_2_), 79.1 (CH), 78.5 (CH), 77.9 (CH), 77.3 (CH), 34.0 (CH_2_), 39.9 (CH_2_), 35.6 (CH_2_), 35.5 (CH_2_), 31.4 (CH_2_), 31.3 (CH_2_), 30.9 (CH_2_), 30.4 (CH_2_), 29.9 (CH_2_), 28.9 (CH_2_), 25.6 (CH_2_), 22.1 (CH_2_), 13.6 (C) ppm; IR (film, cm^–1^): 3070, 2974, 2869, 1641; HRMS (ESI) *m*/*z*: [M + H]^+^ calcd. for C_13_H_25_O, 197.1900; found, 197.1903.

#### (((2*R*,3*S*,5*R*)-5-Allyl-2-(((tert-butyldiphenylsilyl)oxy)methyl)tetrahydrofuran-3-yl)oxy)(tert-butyl)diphenylsilane (**12**)

Purification with hexane/MTBE (98:2) gave 59.1 mg, yield 93%, as colorless oil; ^1^H NMR (300 MHz, CDCl_3_): δ 7.75 (m, 8H), 7.45 (m, 12H), 6.00 (dddd, *J* = 17.2, 10.2, 6.9, 6.9 Hz, 1H), 5.27 (dd, *J* = 16.5, 0.8 Hz, 1H), 5.20 (dd, *J* = 9.6, 0.8 Hz, 1H), 4.62 (ddd, *J* = 6.8, 3.5, 3.5 Hz, 1H), 4.20 (m, 2H), 3.62 (dd, *J* = 10.9, 3.5 Hz, 1H), 3.40 (dd, *J* = 10.9, 3.8 Hz, 1H), 2.65 (ddd, *J* = 13.9, 6.9, 6.9 Hz, 1H), 2.52 (ddd, *J* = 13.9, 6.9, 6.9 Hz, 1H), 2.19 (ddd, *J* = 8.4, 6.0, 1.8 Hz, 1H), 1.89 (ddd, *J* = 12.5, 5.6, 4.9 Hz, 1H), 1.20 (s, 9H), 1.05 (s, 9H) ppm; ^13^C{^1^H} NMR (75 MHz, CDCl_3_): δ 135.7 (CH), 135.5 (CH), 135.4 (CH), 135.3 (CH), 133.7 (C), 133.6 (C), 133.3 (C), 129.6 (CH), 129.4 (CH), 129.4 (CH), 127.6 (CH), 127.5 (CH), 116.5 (CH_2_), 86.6 (CH), 78.6 (CH), 74.8 (CH), 64.4 (CH_2_), 40.8 (CH_2_), 40.3 (CH_2_), 26.8 (CH_3_), 26.6 (CH_3_), 19.0 (C) ppm; IR (film, cm^–1^): 3069, 2933, 2855, 1475, 1431, 1261, 1113, 1002, 935, 777, 741, 702, 614; HRMS (ESI) *m*/*z*: [M + H]^+^ calcd. for C_40_H_51_O_3_Si_2_, 635.3371; found, 635.3370; [α]_D_^20^ + 38.0, *c* = 1.9, CH_2_Cl_2_.

#### ((2*R*,3*S*,5*R*)-3-Acetoxy-5-allyltetrahydrofuran-2-yl)methyl acetate (**29**)

Purification with hexane/AcOEt (9:1) gave 22.3 mg, yield 92%, as colorless oil; ^1^H NMR (300 MHz, CDCl_3_): δ 5.82 (m, 1H), 5.07 (m, 3H), 4.18 (m, 4H), 2.25–2.45 (m, 3H), 2.08 (s, 3H), 2.06 (s, 3H), 1.75 (dt, *J* = 13.1, 5.3 Hz, 1H) ppm; ^13^C{^1^H} NMR (75 MHz, CDCl_3_): δ 170.6 (C), 170.5 (C), 134.0 (CH), 117.4 (CH_2_), 80.8 (CH), 78.1 (CH), 75.6 (CH), 63.9 (CH_2_), 39.9 (CH_2_), 36.7 (CH_2_), 20.9 (CH_3_), 20.7 (CH_3_) ppm; IR (film, cm^–1^): 2953, 1749, 1494, 1451, 1367, 1243, 1171, 1125, 1085, 1026, 992, 896, 768, 721; HRMS (ESI) *m*/*z*: [M + H]^+^ calcd. for C_12_H_19_O_5_, 243.1227; found, 243.1230; [α]_D_^20^ + 34.7, *c* = 1.16, CH_2_Cl_2_.

#### (2*R*,3*S*,5*R*)-5-Allyl-3-(benzyloxy)-2-((benzyloxy)methyl)tetrahydrofuran (**30**)

Purification with hexane/MTBE (97:3) gave 30.1 mg, yield 89%, as colorless oil; ^1^H NMR (300 MHz, CDCl_3_): δ 7.57–7.10 (m, 10H), 5.86 (ddt, *J* = 17.2, 10.2, 7.0 Hz, 1H), 5.23–5.02 (m, 2H), 4.67–4.46 (m, 4H), 4.29–4.07 (m, 3H), 3.65–3.47 (m, 2H), 2.54 (dtt, *J* = 13.2, 6.5, 1.4 Hz, 1H), 2.45–2.23 (m, 2H), 1.82 (ddd, *J* = 12.5, 7.0, 5.2 Hz, 1H) ppm; ^13^C{^1^H} NMR (75 MHz, CDCl_3_): δ 138.2 (C), 138.1 (C), 134.7 (CH), 128.3 (CH), 128.2 (CH), 127.5 (CH), 127.5 (CH), 127.5 (CH), 116.9 (CH_2_), 82.1 (CH), 80.7 (CH), 78.1 (CH), 73.3 (CH_2_), 71.5 (CH_2_), 70.7 (CH_2_), 40.3 (CH_2_), 37.2 (CH_2_) ppm; IR (film, cm^–1^): 3061, 2927, 1445, 1367, 1219, 1081, 1017, 957, 737, 703; HRMS (ESI) *m*/*z*: [M + H]^+^ calcd. for C_22_H_27_O_3_, 339.1955; found, 339.1953; [α]_D_^20^ + 39.5, *c* = 1.5, CH_2_Cl_2_.

#### (2*R*,3*S*,5*R*)-5-Allyl-2-(((*tert*-butyldiphenylsilyl)oxy)methyl)tetrahydrofuran-3-yl 4-methylbenzenesulfonate (**31**)

Purification with hexane/MTBE (92:8) gave 51.1 mg, yield 93%, as colorless oil; ^1^H NMR (300 MHz, CDCl_3_): δ 7.75 (d, *J* = 8.3 Hz, 2H), 7.62 (m, 4H), 7.40 (m, 6H), 7.25 (d, *J* = 8.3 Hz, 2H), 5.85 (m, 1H), 5.25 (dt, *J* = 3.9, 0.8 Hz, 1H), 5.16–5.07 (m, 2H), 4.24 (quintet, *J* = 6.7 Hz, 1H), 4.16 (m, 1H), 3.60 (dd, *J* = 11.0, 4.0 Hz, 2H), 2.50 (s, 3H), 2.25–2.37 (m, 3H), 1.90 (m, 1H), 1.08 (s, 9H) ppm; ^13^C{^1^H} NMR (75 MHz, CDCl_3_): δ 144.7 (C), 135.4 (CH), 134.2 (CH), 133.6 (C), 132.9 (C), 132.8 (C), 129.8 (CH), 129.7 (CH), 129.6 (CH), 127.7 (CH), 127.6 (CH), 127.6 (CH), 117.2 (CH_2_), 83.35 (CH), 82.3 (CH), 78.6 (CH), 63.9 (CH_2_), 40.1 (CH_2_), 37.6 (CH_2_), 26.6 (CH_3_), 21.5 (CH_3_), 19.0 (C) ppm; IR (film, cm^–1^): 2856, 1473, 1429, 1268, 1098, 1009, 951; HRMS (ESI) *m*/*z*: [M + H]^+^ calcd. for C_31_H_39_O_5_SSi, 551.2282; found, 551.2285; [α]_D_^20^ + 34.7, *c* = 0.6 CH_2_Cl_2_.

#### (((2*R*,3*R*,5*S*)-5-Allyl-2-(((*tert*-butyldiphenylsilyl)oxy)methyl)tetrahydrofuran-3-yl)oxy)(tert-butyl)diphenylsilane (**32**)

Purification with hexane/MTBE (98:2) gave 54.2 mg, yield 86%, as colorless oil; ^1^H NMR (300 MHz, CD_2_Cl_2_): δ 7.81–7.27 (m, 20H), 6.01–5.74 (m, 1H), 5.29–4.97 (m, 2H), 4.64–4.39 (m, 1H), 4.25–3.75 (m, 4H), 2.51 (dtt, *J* = 14.5, 6.5, 1.4 Hz, 1H), 2.41–2.25 (m, 1H), 2.14–1.59 (m, 2H), 1.11 (s, 9H), 1.01 (s, 9H) ppm; ^13^C{^1^H} NMR (75 MHz, CD_2_Cl_2_): δ 136.7 (CH), 136.6 (CH), 136.5 (CH), 136.5 (CH), 136.4 (CH), 136.1 (CH), 135.8 (CH), 134.8 (CH), 134.7 (CH), 134.6 (C), 134.1 (C), 134.0 (C), 130.5 (CH), 130.5 (CH), 130.4 (CH), 130.3 (CH), 130.3 (CH), 128.5 (CH), 128.4 (CH), 128.4 (CH), 128.3 (CH), 117.1 (CH_2_), 116.9 (CH_2_), 83.9 (CH), 82.5 (CH), 77.7 (CH), 74.8 (CH), 64.6 (CH_2_), 43.3 (CH_2_), 41.4 (CH_2_), 41.15 (CH_2_), 27.5 (CH_3_), 27.5 (CH_3_), 27.5 (CH_3_), 27.4 (CH_3_), 19.9 (C), 19.8 (C) ppm; IR (film, cm^–1^): 3068, 2934, 2853, 1473, 1435, 1262, 1112, 1000, 936, 774, 742, 701, 618; HRMS (ESI) *m*/*z*: [M + H]^+^ calcd. for C_40_H_51_O_3_Si_2_, 635.3371; found, 635.3375.

#### (2*R*,3*R*,4*S*,5*R*)-2-(Acetoxymethyl)-5-Allyltetrahydrofuran-3,4-diyl diacetate (**33**)

Purification with hexane/AcOEt (85:15) gave 27.9 mg, yield 93%, as pale-yellow oil; ^1^H NMR (300 MHz, CDCl_3_): δ 5.74 (ddt, *J* = 17.1, 10.2, 7.0 Hz, 1H), 5.46 (dd, *J* = 4.6, 3.4 Hz, 1H), 5.34–5.25 (m, 1H), 5.18–5.04 (m, 2H), 4.39–4.02 (m, 4H), 2.56–2.29 (m, 2H), 2.20–2.01 (m, 9H) ppm; ^13^C{^1^H} NMR (75 MHz, CDCl_3_): δ 170.6 (C), 169.7 (C), 169.6 (C), 132.9 (CH), 117.7 (CH_2_), 78.8 (CH), 76.5 (CH), 72.2 (CH), 72.0 (CH), 63.6 (CH_2_), 33.8 (CH_2_), 20.7 (CH_3_), 20.4 (CH_3_), 20.4 (CH_3_) ppm; IR (film, cm^–1^): 2957, 1745, 1494, 1447, 1367, 1243, 1101, 1083, 1047, 891, 771, 723; HRMS (ESI) *m*/*z*: [M + H]^+^ calcd. for C_14_H_21_O_7_, 301.1282; found, 301.1283; NOESY correlations (see the Supporting Information).

#### (2*R*,3*R*,4*R*,5*R*)-2-Allyl-3,4-bis(benzyloxy)-5-((benzyloxy)methyl)tetrahydrofuran (**34**)

Purification with hexane/MTBE (9:1) gave 34.2 mg, yield 77%, as colorless oil; ^1^H NMR (300 MHz, CDCl_3_): δ 7.46–7.19 (m, 15H), 5.94–5.72 (m, 1H), 5.24–4.97 (m, 2H), 4.69–4.32 (m, 4H), 4.17–3.99 (m, 2H), 3.99–3.79 (m, 2H), 3.73–3.46 (m, 2H), 2.52 (tq, *J* = 7.0, 1.4 Hz, 2H) ppm; ^13^C{^1^H} NMR (75 MHz, CDCl_3_): δ 138.2 (C), 137.8 (C), 137.7 (C), 134.8 (CH), 134.1 (C), 128.3 (CH), 128.3 (CH), 128.3 (CH), 128.2 (CH), 128.2 (CH), 127.7 (CH), 127.6 (CH), 127.6 (CH), 127.5 (CH), 127.5 (CH), 127.4 (CH), 116.8 (CH_2_), 83.7 (CH), 82.7 (CH), 82.5 (CH), 80.9 (CH), 73.2 (CH_2_), 71.3 (CH_2_), 71.2 (CH_2_), 70.5 (CH_2_), 33.07 (CH_2_) ppm; IR (film, cm^–1^): 2957, 1445, 1367, 1209, 1081, 1017, 737, 703; HRMS (ESI) *m*/*z*: [M + H]^+^ calcd. for C_29_H_33_O_4_, 445.2373; found, 445.2378; NOESY correlations (see the Supporting Information).

### General Procedures for the Glycosylation Scope

#### Procedure A

The acetal for the specific reaction (0.2 mmol) was dissolved in dry CH_2_Cl_2_ (4 mL) at room temperature under an argon atmosphere; then, the trimethylsilyl-substituted alkyl-based reagent (0.5 mmol, 2.5 equiv) was added under magnetic stirring. The resulting solution has been cooled at −50 °C, and complex **5** (15 mol %) followed by Cu(OTf)_2_ (5 mol %) were added in one portion. The reaction mixture was allowed to warm at −30 °C and stirred at this temperature for 12 h (except for compound **16** which was warmed up to 0 °C). The reaction was then quenched with a saturated solution of NaHCO_3_ (5 mL). The layers were separated, and the aqueous phase was extracted with CH_2_Cl_2_ (3 × 7 mL). The combined organic phases were dried with Na_2_SO_4_, filtered, and concentrated under reduced pressure. The resulting residue was purified by flash chromatography on silica gel.

#### Procedure B

The acetal for the specific reaction (0.2 mmol) was dissolved in dry CH_2_Cl_2_ (4 mL) at room temperature under an argon atmosphere; then, the trimethylsilyl-substituted aromatic reagent (0.5 mmol, 2.5 equiv) was added under magnetic stirring (except for compound **28** where furan was used in 1.5 equiv). The resulting solution has been cooled at −50 °C, and complex **5** (30 mol %) followed by Cu(OTf)_2_ (15 mol %) were added in one portion. The reaction mixture was allowed to warm at −30 °C and stirred at this temperature for 12 h. The reaction was then quenched with a saturated solution of NaHCO_3_ (5 mL). The layers were separated, and the aqueous phase was extracted with CH_2_Cl_2_ (3 × 7 mL). The combined organic phases were dried with Na_2_SO_4_, filtered, and concentrated under reduced pressure. The resulting residue was purified by flash chromatography on silica gel.

##### Methyl 3-(((2*R*,4*S*,5*R*)-4-((*tert*-butyldiphenylsilyl)oxy)-5-(((*tert*-butyldiphenylsilyl)oxy)methyl)tetrahydrofuran-2-yl)methyl)but-3-enoate (**13**-Procedure A)

Purification with hexane/MTBE (85:15) gave 104.5 mg, yield 74%, as colorless oil; ^1^H NMR (400 MHz, (CD_3_)_2_CO): δ 7.67–7.47 (m, 8H), 7.43–7.25 (m, 12H), 5.62–5.46 (m, 2H), 4.49 (ddd, *J* = 6.6, 4.6, 3.4 Hz, 1H), 4.07–3.89 (m, 2H), 3.54 (s, 3H), 3.44 (dd, *J* = 11.0, 3.4 Hz, 1H), 3.32 (dd, *J* = 11.0, 4.0 Hz, 1H), 2.97 (dt, *J* = 4.2, 1.1 Hz, 2H), 2.45–2.22 (m, 2H), 2.04 (dt, *J* = 13.4, 6.7 Hz, 1H), 1.72 (ddd, *J* = 12.6, 6.4, 4.6 Hz, 1H), 1.00 (s, 9H), 0.85 (s, 9H) ppm; ^13^C{^1^H} NMR (101 MHz, (CD_3_)_2_CO: δ 171.5 (C), 135.7 (CH), 135.6 (CH), 135.5 (CH), 135.4 (CH), 133.6 (C), 133.6 (C), 133.3 (C), 130.6 (CH), 129.9 (CH), 129.9 (CH), 129.7 (CH), 129.6 (CH), 127.8 (CH), 127.7 (CH), 127.6 (CH), 124.3 (CH), 86.4 (CH), 78.5 (CH), 74.9 (CH), 64.4 (CH_2_), 50.9 (CH_3_), 40.3 (CH_2_), 39.3 (CH_2_), 37.4 (CH_2_), 26.5 (CH_3_), 26.2 (CH_3_), 18.8 (C), 18.7 (C) ppm; IR (film, cm^–1^): 2937, 1746, 1651, 1562, 1475, 1429, 1113, 1001, 826, 729; HRMS (ESI) *m*/*z*: [M + H]^+^ calcd. for C_43_H_55_O_5_Si_2_, 707.3583; found, 707.3581; [α]_D_^20^ + 33.6, *c* = 0.72, CH_2_Cl_2_.

##### (((2*R*,3*S*,5*S*)-5-(2-bromoallyl)-2-(((*tert*-butyldiphenylsilyl)oxy)methyl)tetrahydrofuran-3-yl)oxy)(*tert*-butyl)diphenylsilane (**14**-Procedure A)

Purification with hexane/MTBE (98:2) gave 140.9 mg, yield 99%, as colorless oil; ^1^H NMR (400 MHz, CDCl_3_): δ 7.60–7.39 (m, 8H), 7.38–7.16 (m, 12H), 5.61 (q, *J* = 1.2 Hz, 1H), 5.42 (d, *J* = 1.6 Hz, 1H), 4.44 (dt, *J* = 6.0, 2.9 Hz, 1H), 4.36 (tdd, *J* = 7.4, 5.9, 4.7 Hz, 1H), 3.97 (td, *J* = 3.7, 2.4 Hz, 1H), 3.38 (dd, *J* = 11.0, 3.8 Hz, 1H), 3.19 (dd, *J* = 11.0, 3.6 Hz, 1H), 2.88 (ddd, *J* = 14.5, 7.3, 1.1 Hz, 1H), 2.62 (ddd, *J* = 14.5, 5.9, 1.1 Hz, 1H), 2.08 (ddd, *J* = 13.4, 7.5, 6.2 Hz, 1H), 1.69 (ddd, *J* = 13.0, 4.8, 3.2 Hz, 1H), 0.99 (s, 9H), 0.84 (s, 9H) ppm; ^13^C{^1^H} NMR (101 MHz, CDCl_3_): δ 135.8 (CH), 135.8 (CH), 135.6 (CH), 135.6 (CH), 133.7 (C), 133.6 (C), 133.3 (C), 133.2 (C), 131.1 (C), 129.8 (CH), 129.6 (CH), 129.6 (CH), 127.7 (CH), 127.6 (CH), 118.5 (CH_2_), 87.2 (CH), 77.4 (CH), 77.1 (CH), 77.0 (CH), 76.7 (CH), 75.0 (CH), 64.5 (CH_2_), 48.3 (CH_2_), 39.9 (CH_2_), 27.0 (CH_3_), 26.8 (CH_3_), 19.1 (C), 19.1 (C) ppm; IR (film, cm^–1^): 3067, 2931, 2861, 1459, 1257, 1119, 1008, 931; HRMS (ESI) *m*/*z*: [M + H]^+^ calcd. for C_40_H_50_O_3_Si_2_Br, 713.2476; found, 713.2478; [α]_D_^20^ + 32.2, *c* = 0.68, CH_2_Cl_2_.

##### *tert*-butyl(((2*R*,3*S*,5*R*)-3-((*tert*-butyldiphenylsilyl)oxy)-5-(2-methylallyl)tetrahydrofuran-2-yl)methoxy)diphenylsilane (**15**-Procedure A)

Purification with hexane/MTBE (9:1) gave 112.7 mg, yield 87%, as colorless oil; ^1^H NMR (300 MHz, CDCl_3_): δ 7.78–7.23 (m, 20H), 4.90–4.73 (m, 2H), 4.53 (ddd, *J* = 6.8, 4.2, 3.0 Hz, 1H), 4.27 (p, *J* = 6.7 Hz, 1H), 4.07 (q, *J* = 3.5 Hz, 1H), 3.52 (dd, *J* = 11.0, 3.6 Hz, 1H), 3.43–3.23 (m, 1H), 2.54 (dd, *J* = 14.0, 7.2 Hz, 1H), 2.34 (dd, *J* = 14.1, 6.4 Hz, 1H), 2.11 (dt, *J* = 13.2, 6.8 Hz, 1H), 1.84–1.71 (m, 4H), 1.17–1.02 (s, 9H), 0.94 (s, 9H) ppm; ^13^C{^1^H} NMR (75 MHz, CDCl_3_): δ 143.1 (C), 135.7 (CH), 135.6 (CH), 135.5 (CH), 135.4 (CH), 133.8 (C), 133.7 (C), 133.3 (C), 133.2 (C), 129.6 (CH), 129.4 (CH), 129.4 (CH), 127.6 (CH), 127.5 (CH), 127.4 (CH), 111.9 (CH_2_), 86.5 (CH), 77.5 (CH), 74.8 (CH), 64.4 (CH_2_), 44.7 (CH_2_), 40.7 (CH_2_), 26.8 (CH_3_), 26.6 (CH_3_), 22.7 (C), 19.0 (C) ppm; IR (film, cm^–1^): 3070, 2933, 2857, 1479, 1429, 1261, 1115, 1002, 935, 702, 614; HRMS (ESI) *m*/*z*: [M + H]^+^ calcd. for C_41_H_53_O_3_Si_2_, 649.3528; found, 649.3529; [α]_D_^20^ + 37.4, *c* = 0.5, CH_2_Cl_2_; NOESY correlations (see the Supporting Information).

##### *tert*-butyl(((2*R*,3*S*,5*S*)-3-((*tert*-butyldiphenylsilyl)oxy)-5-((*S*)-pent-3-yn-2-yl)tetrahydrofuran-2-yl)methoxy)diphenylsilane (**16**-Procedure A)

Purification with hexane/MTBE (99:1) gave 108.2 mg, yield 82%, as colorless oil; ^1^H NMR (400 MHz, CDCl_3_): δ 7.55–7.30 (m, 8H), 7.29–7.05 (m, 12H), 4.29 (ddd, *J* = 6.1, 4.7, 3.3 Hz, 1H), 3.85 (q, *J* = 3.6 Hz, 1H), 3.64 (dt, *J* = 8.5, 6.3 Hz, 1H), 3.32 (dd, *J* = 11.0, 3.6 Hz, 1H), 3.22–3.14 (m, 1H), 2.59 (ddq, *J* = 9.0, 6.8, 2.3 Hz, 1H), 1.97–1.87 (m, 2H), 1.62 (d, *J* = 2.4 Hz, 3H), 1.03 (s, 9H), 0.89 (s, 9H) ppm; ^13^C{^1^H} NMR (101 MHz, CDCl_3_): δ 135.9 (CH), 135.8 (CH), 135.8 (CH), 135.7 (CH), 135.6 (CH), 135.6 (CH), 135.5 (CH), 133.9 (C), 133.8 (C), 133.5 (C), 133.4 (C), 129.8 (CH), 129.7 (CH), 129.5 (CH), 129.5 (CH), 129.4 (CH), 127.7 (CH), 127.6 (CH), 127.6 (CH), 127.6 (CH), 86.9 (CH), 82.9 (C), 81.3 (C), 74.5 (CH), 64.4 (CH_2_), 39.4 (CH), 32.3 (CH_3_), 26.8 (CH_3_), 26.6 (CH_3_), 19.2 (C), 19.1 (C), 3.7 (CH_3_) ppm; IR (film, cm^–1^): 3071, 2923, 2857, 1447, 1477, 1425, 1263, 1112, 1003, 937, 706; HRMS (ESI) *m*/*z*: [M + H]^+^ calcd. for C_42_H_53_O_3_Si_2_, 661.3528; found, 661.3526.

##### (((2*R*,3*S*,5*S*)-5-Azido-2-(((*tert*-butyldiphenylsilyl)oxy)methyl)tetrahydrofuran-3-yl)oxy)(*tert*-butyl)diphenylsilane (**17**-Procedure A)

Purification with hexane/MTBE (7:3) gave 114.3 mg, yield 90%, as pale-yellow oil; ^1^H NMR (300 MHz, CDCl_3_): δ 7.82–7.22 (m, 20H), 5.49 (dd, *J* = 6.4, 1.7 Hz, 1H), 4.47 (ddt, *J* = 6.9, 4.7, 2.8 Hz, 1H), 4.29 (q, *J* = 3.0 Hz, 1H), 3.63–3.41 (m, 1H), 3.26 (dd, *J* = 11.3, 3.3 Hz, 1H), 2.32–1.94 (m, 2H), 1.10 (s, 9H), 0.98 (s, 9H) ppm; ^13^C{^1^H} NMR (75 MHz, CDCl_3_): δ 135.7 (CH), 135.7 (CH), 135.6 (CH), 135.4 (CH), 135.4 (CH), 133.42 (C), 133.4 (C), 133.0 (C), 132.9 (C), 129.8 (CH), 129.7 (CH), 129.6 (CH), 127.7 (CH), 127.6 (CH), 91.9 (CH), 89.0 (CH), 73.4 (CH), 63.7 (CH_2_), 26.8 (CH_3_), 26.6 (CH_3_), 19.0 (C), 18.9 (C) ppm; IR (film, cm^–1^): 3071, 2937, 2859, 2137, 1487, 1427, 1261, 1111, 1009, 931, 703; HRMS (ESI) *m*/*z*: [M + H]^+^ calcd. for C_37_H_46_O_3_N_3_Si_2_, 636.3072; found, 636.3075.

##### *tert*-butyl(((2*R*,3*S*)-2-(((*tert*-butyldiphenylsilyl)oxy)methyl)tetrahydrofuran-3-yl)oxy)diphenylsilane (**18**-Procedure A)

Purification with hexane/MTBE (99:1) gave 114.1 mg, yield 96%, as colorless oil; ^1^H NMR (300 MHz, CDCl_3_): δ 7.89–7.53 (m, 8H), 7.49–7.22 (m, 12H), 4.47 (td, *J* = 3.9, 2.0 Hz, 1H), 4.14–3.92 (m, 3H), 3.42 (qd, *J* = 10.9, 4.3 Hz, 2H), 1.87 (dtd, *J* = 8.7, 4.6, 1.4 Hz, 2H), 1.11 (s, 9H), 0.99 (s, 9H) ppm; ^13^C{^1^H} NMR (75 MHz, CDCl_3_): δ 135.7 (CH), 135.5 (CH), 135.4 (CH), 133.9 (C), 133.7 (C), 133.3 (C), 133.3 (C), 129.6 (CH), 129.5 (CH), 129.4 (CH), 127.6 (CH), 127.6 (CH), 127.5 (CH), 127.5 (CH), 87.1 (CH), 75 (CH), 67.5 (CH_2_), 64.5 (CH_2_), 35.6 (CH_2_), 26.9 (CH_3_), 26.7 (CH_3_), 19.1 (C), 19.0 (C) ppm; IR (film, cm^–1^): 3067, 2939, 2861, 1485, 1421, 1264, 1112, 1011, 927, 705; HRMS (ESI) *m*/*z*: [M + H]^+^ calcd. for C_37_H_47_O_3_Si_2_, 595.3058; found, 595.3059; [α]_D_^20^ + 23.7, *c* = 1.7, CH_2_Cl_2_.

##### *tert*-butyl(((2*R*,3*S*,5*R*)-3-((*tert*-butyldiphenylsilyl)oxy)-5-(phenylthio)tetrahydrofuran-2-yl)methoxy)diphenylsilane (**19**-Procedure A)

Purification with hexane/AcOEt (99:1) gave 134.8 mg, yield 96%, as colorless oil; ^1^H NMR (300 MHz, CDCl_3_): δ 7.80–7.15 (m, 25H), 5.68 (dd, *J* = 7.5, 4.1 Hz, 1H), 4.47 (dt, *J* = 8.0, 4.0 Hz, 1H), 4.40–4.27 (m, 1H), 3.70 (dd, *J* = 11.3, 2.7 Hz, 1H), 3.54–3.41 (m, 1H), 2.49 (dt, *J* = 14.1, 7.2 Hz, 1H), 2.12 (dt, *J* = 13.6, 4.0 Hz, 1H), 1.15 (s, 9H), 0.98 (s, 9H) ppm; ^13^C{^1^H} NMR (75 MHz, CDCl_3_): δ 137.0 (C), 135.8 (CH), 135.7 (CH), 135.6 (CH), 135.6 (CH), 135.5 (CH), 135.5 (CH), 135.5 (CH), 135.1 (C), 133.6* (C), 133.6* (C), 133.5* (C), 133.4* (C), 133.3* (C), 133.2* (C), 133.1* (C), 133.1* (C), 131.2* (CH), 130.4 (CH), 129.7 (CH), 129.7* (CH), 129.5* (C), 129.5 (CH), 128.7* (CH), 128.7 (CH), 127.7 (CH), 127.6 (CH), 127.5* (CH), 127.5* (CH), 127.5* (CH), 126.8* (CH), 126.4 (CH), 88.8* (CH), 87.2 (CH), 86.2 (CH), 85.8* (CH), 74.3* (CH), 72.8 (CH), 64.1* (CH_2_), 63.4 (CH_2_), 42.5 (CH_2_), 41.3* (CH_2_), 29.6 (CH_2_), 26.8 (CH_3_), 26.7* (CH_3_), 26.6 (CH_3_), 19.1 (C), 19.1 (C) ppm; IR (film, cm^–1^): 3134, 3071, 3050, 3015, 2957, 2931, 2893, 2857, 2773, 2739, 2710, 2316, 1960, 1891, 1825, 1775, 1731, 1660, 1587, 1472, 1427, 1390, 1256, 1112, 702, 612, 505, 436, 423, 413; HRMS (ESI) *m*/*z*: [M + H]^+^ calcd. for C_43_H_51_O_3_SSi_2_, 703.3092; found, 703.3094.

##### Methyl 2-((2*R*,4*S*,5*R*)-4-((*tert*-butyldiphenylsilyl)oxy)-5-(((*tert*-butyldiphenylsilyl)oxy)methyl)tetrahydrofuran-2-yl)-2-methylpropanoate (**20**-Procedure A)

Purification with hexane/MTBE (85:15) gave 102.7 mg, yield 74%, as colorless oil; ^1^H NMR (400 MHz, CDCl_3_): δ 7.60–7.42 (m, 8H), 7.38–7.16 (m, 12H), 4.44 (ddd, *J* = 7.1, 6.2, 4.6 Hz, 1H), 4.16 (dd, *J* = 9.3, 6.8 Hz, 1H), 3.85–3.78 (m, 1H), 3.56 (s, 3H), 3.54–3.48 (m, 1H), 3.31–3.22 (m, 1H), 1.92 (dt, *J* = 12.6, 7.0 Hz, 1H), 1.72 (ddd, *J* = 12.6, 9.3, 6.2 Hz, 1H), 1.19 (s, 3H), 1.08 (s, 3H), 0.98 (d, *J* = 9.4 Hz, 9H), 0.84 (d, *J* = 5.1 Hz, 9H) ppm; ^13^C{^1^H} NMR (101 MHz, CDCl_3_): δ 177.0 (C), 135.8 (CH), 135.8 (CH), 135.8 (CH), 135.7 (CH), 135.6 (CH), 135.6 (CH), 133.8 (C), 133.7 (C), 133.6 (C), 133.4 (C), 129.7 (CH), 129.5 (CH), 129.5 (CH), 127.7 (CH), 127.7 (CH), 127.7 (CH), 127.6 (CH), 127.59, 127.6 (CH), 86.4 (CH), 83.0 (CH), 73.8 (CH), 64.2 (CH_2_), 51.8 (CH_3_), 46.0 (C), 36.9 (CH_2_), 26.9 (CH_3_), 26.7 (CH_3_), 21.1 (CH_3_), 20.5 (CH_3_), 19.1 (C), 19.0 (C) ppm; IR (film, cm^–1^): 2935, 1748, 1653, 1561, 1475, 1429, 1113, 824, 738; HRMS (ESI) *m*/*z*: [M + H]^+^ calcd. for C_42_H_55_O_5_Si_2_, 695.3583; found, 695.3585.

##### 2-((2*S*,4*S*,5*R*)-4-((*tert*-butyldiphenylsilyl)oxy)-5-(((*tert*-butyldiphenylsilyl)oxy)methyl)tetrahydrofuran-2-yl)-1-phenylethan-1-one (**21**-Procedure A)

Purification with hexane/AcOEt (9:1) gave 95.4 mg, yield 67%, as colorless oil; ^1^H NMR (400 MHz, CDCl_3_): δ 7.96–7.87 (m, 2H), 7.61–7.11 (m, 23H), 4.70–4.59 (m, 1H), 4.48–4.41 (m, 1H), 4.04 (td, *J* = 4.0, 2.2 Hz, 1H), 3.54 (dd, *J* = 16.4, 6.2 Hz, 1H), 3.33 (dd, *J* = 11.0, 4.2 Hz, 1H), 3.27 (d, *J* = 7.1 Hz, 1H), 3.25–3.18 (m, 1H), 2.21 (ddd, *J* = 13.4, 7.6, 6.1 Hz, 1H), 1.75 (ddd, *J* = 13.1, 4.4, 2.9 Hz, 1H), 0.98 (s, 9H), 0.83 (s, 9H) ppm; ^13^C{^1^H} NMR (101 MHz, CDCl_3_): δ 198.9 (C), 198.4* (C), 137.3 (C), 137.2* (C), 135.8* (CH), 135.8 (CH), 135.6 (CH), 135.6 (CH), 135.6 (CH), 133.9* (C), 133.9* (C), 133.60 (C), 133.4 (C), 133.3 (C), 133.1 (CH), 129.8 (CH), 129.7 (CH), 129.6* (CH), 129.6 (CH), 128.6 (CH), 128.3 (CH), 128.3 (CH), 127.8 (CH), 127.8 (CH), 127.7 (CH), 127.6 (CH), 87.9* (CH), 87.2 (CH), 75.9 (CH), 75.6* (CH), 75.4 (CH), 75.2* (CH), 64.5 (CH_2_), 64.3* (CH_2_), 45.7 (CH_2_), 44.5* (CH_2_), 42.0* (CH_2_), 40.8 (CH_2_), 27.0 (CH_3_), 26.8 (CH_3_), 26.8 (CH_3_), 19.2* (C), 19.1 (C), 19.1 (C) ppm; IR (film, cm^–1^): 3070, 3050, 2998, 2957, 2931, 2893, 2857, 1961, 1897, 1823, 1684, 1597, 1472, 1448, 1428, 1389, 1361, 1306, 1263, 1208, 1112, 1039, 999, 938, 822, 741, 702, 612, 506, 446, 413; HRMS (ESI) *m*/*z*: [M + H]^+^ calcd. for C_45_H_53_O_4_Si_2_, 713.3477; found, 713.3474.

##### 1-((2*S*,4*S*,5*R*)-4-((*tert*-butyldiphenylsilyl)oxy)-5-(((*tert*-butyldiphenylsilyl)oxy)methyl)tetrahydrofuran-2-yl)propan-2-one (**22**-Procedure A)

Purification with hexane/AcOEt (9:1) gave 101.5 mg, yield 78%, as colorless oil; ^1^H NMR (400 MHz, CDCl_3_): δ 7.61–7.14 (m, 20H), 4.47–4.33 (m, 2H), 3.97 (q, *J* = 3.7 Hz, 1H), 3.35 (dd, *J* = 11.0, 3.9 Hz, 1H), 3.20 (dd, *J* = 11.0, 3.9 Hz, 1H), 2.93 (dd, *J* = 16.0, 7.3 Hz, 1H), 2.66 (dd, *J* = 16.0, 6.0 Hz, 1H), 2.17–2.04 (m, 4H), 1.63 (ddd, *J* = 13.0, 5.0, 3.5 Hz, 1H), 0.99 (s, 9H), 0.84 (s, 9H) ppm; ^13^C{^1^H} NMR (101 MHz, CDCl_3_): δ 207.8 (C), 135.8 (CH), 135.8 (CH), 135.6 (CH), 135.6 (CH), 135.5 (CH), 133.6 (C), 133.5 (C), 133.3 (C), 133.2 (C), 129.8 (CH), 129.8 (CH), 129.6 (CH), 127.8 (CH), 127.7 (CH), 127.6 (CH), 87.0 (CH), 75.3 (CH), 75.0 (CH), 64.4 (CH_2_), 50.6 (CH_2_), 40.8 (CH_2_), 30.8 (CH_3_), 27.0 (CH_3_), 26.7 (CH_3_), 19.1 (C), 19.1 (C) ppm; IR (film, cm^–1^): 3134, 3071, 3049, 2998, 2958, 2931, 2893, 2858, 2709, 1961, 1892, 1825, 1715, 1589, 1472, 1428, 1360, 1242, 1189, 1111, 1007, 936, 823, 741, 703, 612, 506, 443; HRMS (ESI) *m*/*z*: [M + H]^+^ calcd. for C_40_H_51_O_4_Si_2_, 651.3320; found, 651.3324.

##### (*S*)-2-((2*R*,4*S*,5*R*)-4-((*tert*-butyldiphenylsilyl)oxy)-5-(((*tert*-butyldiphenylsilyl)oxy)methyl)tetrahydrofuran-2-yl)-2-((trimethylsilyl)oxy)cyclobutan-1-one (**23**-Procedure A)

Purification with hexane/MTBE (94:6) gave 132.0 mg, yield 88%, as colorless oil; ^1^H NMR (400 MHz, CD_2_Cl_2_): δ 7.56–7.13 (m, 20H), 5.21–5.14 (m, 1H), 4.36 (ddd, *J* = 7.1, 5.7, 4.2 Hz, 1H), 3.92–3.84 (m, 1H), 3.40 (dd, *J* = 11.2, 2.8 Hz, 1H), 3.19 (dd, *J* = 11.2, 3.9 Hz, 1H), 2.78–2.45 (m, 3H), 2.03–1.91 (m, 1H), 1.90–1.79 (m, 2H), 0.94 (s, 9H), 0.79 (s, 9H), 0.00 (s, 9H) ppm; ^13^C{^1^H} NMR (101 MHz, CD_2_Cl_2_): δ 210.8 (C), 209.8* (C), 135.5* (CH), 135.5 (CH), 135.4 (CH), 135.4* (CH), 135.3* (CH), 135.2 (CH), 135.2 (CH), 135.2* (CH), 135.1* (CH), 133.4* (C), 133.3 (C), 133.3 (C), 133.2* (C), 133.0 (C), 132.9 (C), 129.5 (CH), 129.4* (CH), 129.3 (CH), 129.2 (CH), 129.2 (CH), 129.2 (CH), 127.4 (CH), 127.4 (CH), 127.3* (CH), 127.3* (CH), 127.3 (CH), 127.3 (CH), 127.2* (CH), 125.1* (CH), 93.8 (C), 93.2* (C), 87.8* (CH), 87.7* (CH), 87.0 (CH), 85.6* (CH), 80.9* (CH), 80.3* (CH), 79.6 (CH), 79.0* (CH), 74.4* (CH), 73.3 (CH), 72.6* (CH), 64.1 (CH_2_), 63.6* (CH_2_), 40.2 (CH_2_), 40.1* (CH_2_), 36.6 (CH_2_), 36.1* (CH_2_), 33.8* (CH), 29.8 (CH), 29.4* (CH), 26.4 (CH_3_), 26.4* (CH_3_), 26.2* (CH_3_), 26.2 (CH_3_), 23.8 (CH_2_), 23.3* (CH_2_), 20.5* (CH_3_), 18.7 (C), 18.6 (C), 0.9 (CH_3_) ppm; IR (film, cm^–1^): 3640, 3135, 3071, 3050, 2999, 2957, 2931, 2894, 2858, 2710, 1960, 1891, 1789, 1661, 1589, 1472, 1428, 1389, 1362, 1308, 1252, 1180, 1111, 1008, 980, 937, 844, 740, 702, 612, 504, 421; HRMS (ESI) *m*/*z*: [M + H]^+^ calcd. for C_44_H_59_O_5_Si_3_, 751.3665; found, 751.3668; NOESY correlations (see the Supporting Information).

##### (*R*)-2-((2*R*,4*S*,5*R*)-4-((*tert*-butyldiphenylsilyl)oxy)-5-(((*tert*-butyldiphenylsilyl)oxy)methyl)tetrahydrofuran-2-yl)cyclohexan-1-one (**24**-Procedure A)

Purification with hexane/AcOEt (9:1) gave 114.5 mg, yield 83%, as colorless oil; ^1^H NMR (400 MHz, CDCl_3_): δ 7.56–7.40 (m, 8H), 7.35–7.18 (m, 12H), 4.37 (dt, *J* = 6.4, 3.3 Hz, 1H), 4.10 (ddd, *J* = 9.1, 7.2, 5.2 Hz, 1H), 3.96–3.83 (m, 1H), 3.33 (dd, *J* = 10.9, 4.0 Hz, 1H), 3.21 (dd, *J* = 11.0, 4.2 Hz, 1H), 2.74 (ddd, *J* = 11.8, 9.2, 5.4 Hz, 1H), 2.43 (dt, *J* = 13.4, 3.9 Hz, 1H), 2.31–2.20 (m, 3H), 2.00 (d, *J* = 9.2 Hz, 1H), 1.81 (dp, *J* = 18.9, 6.4, 5.2 Hz, 2H), 1.73–1.53 (m, 2H), 1.41 (dtd, *J* = 22.6, 11.2, 10.2, 4.5 Hz, 1H), 0.97 (s, 9H), 0.84 (s, 9H) ppm; ^13^C{^1^H} NMR (101 MHz, CDCl_3_): δ 212.8 (C), 135.9* (CH), 135.8 (CH), 135.8 (CH), 135.8* (CH), 135.7* (CH), 135.6 (CH), 133.7 (C), 133.6 (C), 133.4 (C), 133.4 (C), 129.8 (CH), 129.8 (CH), 129.5 (CH), 129.5 (CH), 127.7 (CH), 127.7* (CH), 127.6* (CH), 127.6 (CH), 127.6 (CH), 86.4 (CH), 78.0 (CH), 74.9 (CH), 64.4 (CH_2_), 56.8 (CH), 42.8 (CH_2_), 42.0 (CH_2_), 40.5 (CH_2_), 31.4 (CH_2_), 28.4 (CH_2_), 27.0 (CH_2_), 27.0 (CH_3_), 26.7 (CH_3_), 25.0* (CH_2_), 24.9 (CH_2_), 19.1 (C), 19.1 (C) ppm; IR (film, cm^–1^): 3071, 3049, 2956, 2931, 2893, 2858, 2739, 2710, 1960, 1891, 1825, 1709, 1589, 1472, 1428, 1390, 1264, 1112, 999, 970, 823, 740, 703, 612, 506; HRMS (ESI) *m*/*z*: [M + H]^+^ calcd. for C_43_H_55_O_4_Si_2_, 691.3633; found, 691.3630.

##### (*R*)-5-((2*R*,4*S*,5*R*)-4-((*tert*-butyldiphenylsilyl)oxy)-5-(((*tert*-butyldiphenylsilyl)oxy)methyl)tetrahydrofuran-2-yl)furan-2(5*H*)-one (**25**-Procedure B)

Purification with hexane/AcOEt (9:1) gave 74.3 mg, yield 55%, as colorless oil; ^1^H NMR (400 MHz, CDCl_3_): δ 7.68 (dd, *J* = 5.7, 1.5 Hz, 1H), 7.60–7.17 (m, 20H), 6.10 (dd, *J* = 5.7, 1.9 Hz, 1H), 5.17 (dt, *J* = 8.5, 1.7 Hz, 1H), 4.39 (td, *J* = 4.1, 1.8 Hz, 1H), 4.03 (tt, *J* = 3.8, 2.1 Hz, 1H), 3.78 (dt, *J* = 8.6, 5.6 Hz, 1H), 3.31 (dd, *J* = 11.1, 4.0 Hz, 1H), 3.13 (dd, *J* = 11.1, 3.7 Hz, 1H), 2.20–2.10 (m, 2H), 0.99 (s, 9H), 0.83 (s, 9H) ppm; ^13^C{^1^H} NMR (101 MHz, CDCl_3_): δ 173.2 (C), 156.2 (CH), 135.8 (CH), 135.7 (CH), 135.5 (CH), 135.5 (CH), 135.5 (CH), 133.4 (C), 133.1 (C), 133.1 (C), 133.0 (CH), 130.0 (CH), 129.9 (CH), 129.7 (CH), 127.9 (CH), 127.8 (CH), 127.7 (CH), 127.6 (CH), 121.7 (CH), 88.4 (CH), 85.0 (CH), 80.6 (CH), 75.0 (CH), 64.4 (CH_2_), 38.4 (CH_2_), 27.0 (CH_3_), 26.7 (CH_3_), 19.1 (C), 19.0 (C) ppm; IR (film, cm^–1^): 3071, 3049, 2956, 2931, 2894, 2858, 1787, 1760, 1589, 1472, 1428, 1390, 1362, 1155, 1113, 1046, 1007, 950, 886, 822, 703, 613, 506, 442, 410; HRMS (ESI) *m*/*z*: [M + H]^+^ calcd. for C_41_H_49_O_5_Si_2_, 677.3113; found 677.3117; NOESY correlations (see the Supporting Information).

##### *tert*-butyl(((2*R*,3*S*,5*R*)-3-((*tert*-butyldiphenylsilyl)oxy)-5-(4-methoxyphenyl)tetrahydrofuran-2-yl)methoxy)diphenylsilane (**26**-Procedure B)

Purification with hexane/MTBE (93:7) gave 99.4 mg, yield 71%, as colorless oil; ^1^H NMR (300 MHz, CDCl_3_): δ 7.79–7.20 (m, 22H), 6.88–6.79 (m, 2H), 5.25 (dd, *J* = 11.0, 4.7 Hz, 1H), 4.61 (d, *J* = 5.2 Hz, 1H), 4.16 (td, *J* = 3.9, 1.5 Hz, 1H), 3.81 (s, 3H), 3.60 (dd, *J* = 11.0, 4.0 Hz, 1H), 3.51–3.33 (m, 1H), 2.22–2.10 (m, 1H), 1.85 (ddd, *J* = 12.7, 11.0, 5.2 Hz, 1H), 1.14 (s, 9H), 0.99 (d, *J* = 5.1 Hz, 9H) ppm; ^13^C{^1^H} NMR (75 MHz, CDCl_3_): δ 158.9 (C), 135.7 (CH), 135.5 (CH), 135.5 (CH), 133.9 (C), 133.8 (C), 133.7 (C), 133.2 (C), 133.1 (C), 129.6 (CH), 129.5 (CH), 129.4 (CH), 127.6 (CH), 127.6 (CH), 127.5 (CH), 127.5 (CH), 127.5 (CH), 113.5 (CH), 88.0 (CH), 80.0 (CH), 75.72 (CH), 64.4 (CH_2_), 55.2 (CH_3_), 44.6 (CH_2_), 26.9 (CH_3_), 26.67 (CH_3_), 19.1 (C) ppm; IR (film, cm^–1^): 3067, 2929, 2861, 1491, 1247, 1117, 1019, 931, 827, 734; HRMS (ESI) *m*/*z*: [M + H]^+^ calcd. for C_44_H_53_O_4_Si_2_, 701.3477; found, 701.3479; NOESY correlations (see the Supporting Information).

##### (((2*R*,3*S*,5*R*)-5-(benzo[*d*][1,3]dioxol-5-yl)-2-(((*tert*-butyldiphenylsilyl)oxy)methyl)tetrahydrofuran-3-yl)oxy)(*tert*-butyl)diphenylsilane (**27**-Procedure B)

Purification with hexane/MTBE (92:8) gave 100.0 mg, yield 70%, as colorless oil; ^1^H NMR (300 MHz, CDCl_3_): δ 7.76–7.53 (m, 8H), 7.52–7.26 (m, 12H), 7.08–6.71 (m, 3H), 5.96 (dd, *J* = 10.3, 1.3 Hz, 2H), 5.28–4.97 (m, 1H), 4.74–4.49 (m, 1H), 4.31–4.11 (m, 1H), 3.72–3.54 (m, 1H), 3.52–3.38 (m, 1H), 2.51–1.72 (m, 2H), 1.10 (dd, *J* = 23.2, 1.3 Hz, 8H), 0.99 (dd, *J* = 4.4, 1.3 Hz, 9H) ppm; ^13^C{^1^H} NMR (75 MHz, CDCl_3_): δ 147.5 (C), 146.7 (C), 135.8 (CH), 135.7 (CH), 135.6 (CH), 135.5 (CH), 135.4 (CH), 133.8 (C), 133.7 (C), 133.1 (CH), 133.0 (CH), 129.6 (CH), 129.5 (CH), 129.5 (CH), 129.4 (CH), 127.6 (CH), 127.6 (CH), 127.5 (CH), 119.5 (CH), 107.8 (CH), 106.7 (CH), 100.8 (CH_2_), 88.1 (CH), 80.2 (CH), 75.7 (CH), 64.4 (CH_2_), 44.7 (CH_2_), 26.9 (CH_3_), 26.7 (CH_3_), 19.0 (C) ppm; IR (film, cm^–1^): 3071, 2921, 2857, 1502, 1481, 1463, 1233, 1115, 1092, 1041, 939, 739; HRMS (ESI) *m*/*z*: [M + H]^+^ calcd. for C_44_H_51_O_5_Si_2_, 715.3270; found, 715.3269; NOESY correlations (see the Supporting Information).

##### *tert*-Butyl(((2*R*,3*S*,5*R*)-3-((*tert*-butyldiphenylsilyl)oxy)-5-(furan-2-yl)tetrahydrofuran-2-yl)methoxy)diphenylsilane (**28**-Procedure B)

Purification with hexane/MTBE (9:1) gave 75.4 mg, yield 57%, as colorless oil; ^1^H NMR (400 MHz, (CD_3_)_2_CO): δ 7.78–7.29 (m, 21H), 6.39–6.30 (m, 2H), 5.25 (dd, *J* = 10.4, 5.4 Hz, 1H), 4.74–4.63 (m, 1H), 4.12 (td, *J* = 4.3, 1.7 Hz, 1H), 3.54–3.46 (m, 1H), 3.41 (dd, *J* = 10.9, 4.1 Hz, 1H), 2.25–2.09 (m, 2H), 1.13 (s, 9H), 0.95 (s, 9H) ppm; ^13^C{^1^H} NMR (101 MHz, (CD_3_)_2_CO): δ 154.1 (C), 142.4 (CH), 135.7 (CH), 135.7 (CH), 135.7 (CH), 135.5 (CH), 135.5 (CH), 135.4 (CH), 133.6 (C), 133.5 (C), 133.1 (C), 133.1 (C), 130.0 (CH), 129.9 (CH), 129.7 (CH), 129.6 (CH), 127.9 (CH), 127.7 (CH), 127.6 (CH), 110.1 (CH), 107.3 (CH), 87.8 (CH), 75.3 (CH), 73.2 (CH), 64.3 (CH_2_), 40.0 (CH_2_), 26.5 (CH_3_), 26.4 (CH_3_), 18.76 (C) ppm; IR (film, cm^–1^): 3067, 2939, 2861, 1693, 1483, 1421, 1382, 1167, 1061, 992, 745; HRMS (ESI) *m*/*z*: [M + H]^+^ calcd. for C_41_H_49_O_4_Si_2_, 661.3164; found, 661.3166.

##### (2*R*,3*R*,4*S*,5*S*)-2-(Acetoxymethyl)-5-(2-methoxyphenyl)tetrahydrofuran-3,4-diyl diacetate (**35**-Procedure B)

Purification with hexane/AcOEt (8:2) gave 57.8 mg, yield 79%, as colorless oil; ^1^H NMR (300 MHz, CDCl_3_): δ 7.52 (ddd, *J* = 7.6, 1.8, 0.8 Hz, 1H), 7.32–7.20 (m, 1H), 6.97 (td, *J* = 7.5, 1.1 Hz, 1H), 6.81 (dd, *J* = 8.2, 1.0 Hz, 1H), 5.83 (dd, *J* = 4.4, 3.1 Hz, 1H), 5.56 (d, *J* = 3.1 Hz, 1H), 5.45 (dd, *J* = 8.1, 4.5 Hz, 1H), 4.51–4.37 (m, 2H), 4.29–4.17 (m, 1H), 3.80 (s, 3H), 2.23–1.99 (m, 6H), 1.77 (s, 3H) ppm; ^13^C{^1^H} NMR (75 MHz, CDCl_3_): δ 170.7 (C), 169.7 (C), 169.3 (C), 155.7 (C), 128.6 (CH), 127.2 (CH), 124.1 (C), 119.9 (CH), 109.5 (CH), 76.8 (CH), 76.5 (CH), 72.6 (CH), 72.0 (CH), 63.6 (CH_2_), 55.1 (CH_3_), 20.8 (CH_3_), 20.4 (CH_3_), 20.2 (CH_3_) ppm; IR (film, cm^–1^): 2961, 1743, 1497, 1446, 1367, 1245, 1043, 1047, 891, 701; HRMS (ESI) *m*/*z*: [M + H]^+^ calcd. for C_18_H_23_O_8_, 367.1387; found, 367.1384; NOESY correlations (see the Supporting Information). The spectroscopic data are in agreement with those reported in the literature^26b^ but refer to a single anomeric compound.

### Allylation Scope on Different Heterocyclic Cores

The procedure herein adopted is the same as described for the previous allylation reactions.

#### 1-Allyl-1,3-dihydroisobenzofuran (**37**)

Purification with hexane/MTBE (99:1) gave 27.6 mg, yield 86%, as colorless oil; the spectroscopic data correspond with those reported in the literature (See the Supporting Information for ^1^H NMR spectra).^34^

#### Methyl 2-allylpyrrolidine-1-carboxylate (**39**)

Purification with hexane/MTBE (78:22) gave 30.4 mg, yield 90%, as colorless oil; the spectroscopic data correspond with those reported in the literature (See the Supporting Information for ^1^H NMR spectra).^35^

#### (2*S*,4*S*)-2-Allyl-4-benzyl-3-tosyloxazolidine (**41**)^36^

Purification with hexane/MTBE (7:3) gave 45.0 mg, yield 63%, as colorless oil; ^1^H NMR (300 MHz, CDCl_3_): δ 7.83–7.70 (m, 2H), 7.42–7.14 (m, 7H), 5.89 (ddt, *J* = 17.2, 10.1, 7.0 Hz, 1H), 5.30–5.13 (m, 2H), 5.00 (dd, *J* = 7.5, 3.2 Hz, 1H), 3.84 (ddq, *J* = 10.1, 7.1, 3.6 Hz, 1H), 3.74 (dd, *J* = 9.1, 3.1 Hz, 1H), 3.28–3.09 (m, 2H), 2.91–2.65 (m, 2H), 2.57–2.36 (m, 4H) ppm; ^13^C{^1^H} NMR (75 MHz, CDCl_3_): δ 144.1 (C), 137.3 (C), 134.1 (C), 132.3 (CH), 129.8 (CH), 129.7 (CH), 129.4 (CH), 129.2 (CH), 128.6 (CH), 128.6 (CH), 128.5 (CH), 127.7 (CH), 126.7 (CH), 118.4 (CH_2_), 91.81, 68.8 (CH_2_), 60.50, 40.9 (CH_2_), 40.46 (CH_2_), 21.43 (CH_3_) ppm; IR (film, cm^–1^): 2985, 2923, 2885, 2254, 1598, 1456, 1337, 1232, 1157, 1093, 904, 725; HRMS (ESI) *m*/*z*: [M + H]^+^ calcd. for C_20_H_24_NO_3_S, 358.1471; found, 358.1475; [α]_D_^20^ – 65.0, *c* = 0.55 in CH_2_Cl_2_; NOESY correlations (see the Supporting Information).

### Large-Scale Synthesis

The allylation reaction of **11** to give **12** was tested on a larger scale to prove the scale up of the synthesis. Specifically, an identical procedure for the synthesis of **12** was followed, using 750 mg of the starting acetal **11** (1.2 mmol) and the corresponding percentages of Re catalyst **8** (15 mol %, 0.18 mmol, 117 mg) and Cu(OTf)_2_ (5 mol %, 0.06 mmol, 22 mg). Purification with hexane/MTBE (98:2) gave 700 mg of product **12** (1.1 mmol) with an isolated yield 92%, as colorless oil. NMR spectra correspond with those obtained for the sub-millimolar scale.

#### Computational Methods

All structures studied during the DFT investigation of the anisole reaction mechanism were optimized with the Gaussian 09 program package.^37^ Previously reported studies on the allylation reaction of five-membered ring oxocarbenium ions, by Woerpel and co-workers, showed that the use of B3LYP could results in difficulties in locating the transitions states when allyltrimethylsilane was involved in the reaction, thus suggesting the use of M06-2X.^38^ Considering the different types of reactants involved, we decided to use the more parametrized B3LYP functional anyway, by specifying a differentiated basis set.^39^ This resulted in the absence of any problem in locating the corresponding TSs. Specifically, C and H atoms were described using the 6-31G(d,p) basis set, whereas O and Si atoms were described with the 6-31+G(d,p) basis set.^40^ Vibrational frequencies were computed at the same level of theory to verify that the optimized structures were minima or TSs. For TS, IRC calculations were computed at the same level of theory in order to confirm the structures at the ends. Solvent effects were then evaluated by running single-point calculations at the same level of theory, the polarizable continuum solvent model (PCM) was used for CH_2_Cl_2_. NPA charges on C1 (furanoside numbering system) are calculated in CH_2_Cl_2_ at the same level of theory. TMS was used to model the presence of a TBDPS-protecting group.^41^

## Supporting Information Available

1H, 13C{1H} NMR spectra for all new compounds, NOESY correlations and spectra, computational details, summary tables of geometrical features, Cartesian coordinates of all computed structures (PDF).
