# Supplementary material for: Highly Stereoselective Glycosylation Reactions of Furanoside Derivatives via Rhenium (V) Catalysis
Source: J Org Chem. 2021 May 25;86(11):7672–86. doi: 10.1021/acs.joc.1c00706 (PMC8279489; doi:10.1021/acs.joc.1c00706)
Supplement: Supplementary file 1 — jo1c00706_si_001.pdf [file jo1c00706_si_001.pdf]

**Supporting information**

# Highly Stereoselective Glycosylation Reactions of Furanoside Derivatives via Rhenium (V) Catalysis

*Emanuele Casali,* [†] *Othman T. Sirwan,* [‡] *Ahmed A. Dezaye,* [§] *Debora Chiodi,* [†] *Alessio Porta* [†] *and Giuseppe Zanoni* [†*]

[†] Department of Chemistry, University of Pavia, Viale Taramelli, 12 - 27100 - Pavia (Italy)
e-mail: gz@unipv.it

[‡] Department of Chemistry, College of Science, Salahaddin University-Erbil, 44002, Erbil (Iraq)

[§] International University of Erbil, Newroz Str., 44001, Erbil-Kurdistan- (Iraq)

**$^{1}H$-$^{13}C\{^{1}H\}$ NMR spectra** page S3

**NOESY correlations and spectra** page S75

**Computational extra material** page S84

**Cartesian coordinates, energies and frequencies** page S88

**$^{1}$H-$^{13}$C{$^{1}$H} NMR Spectra (Compound 9, 300 MHz, $CD_2Cl_2$)**

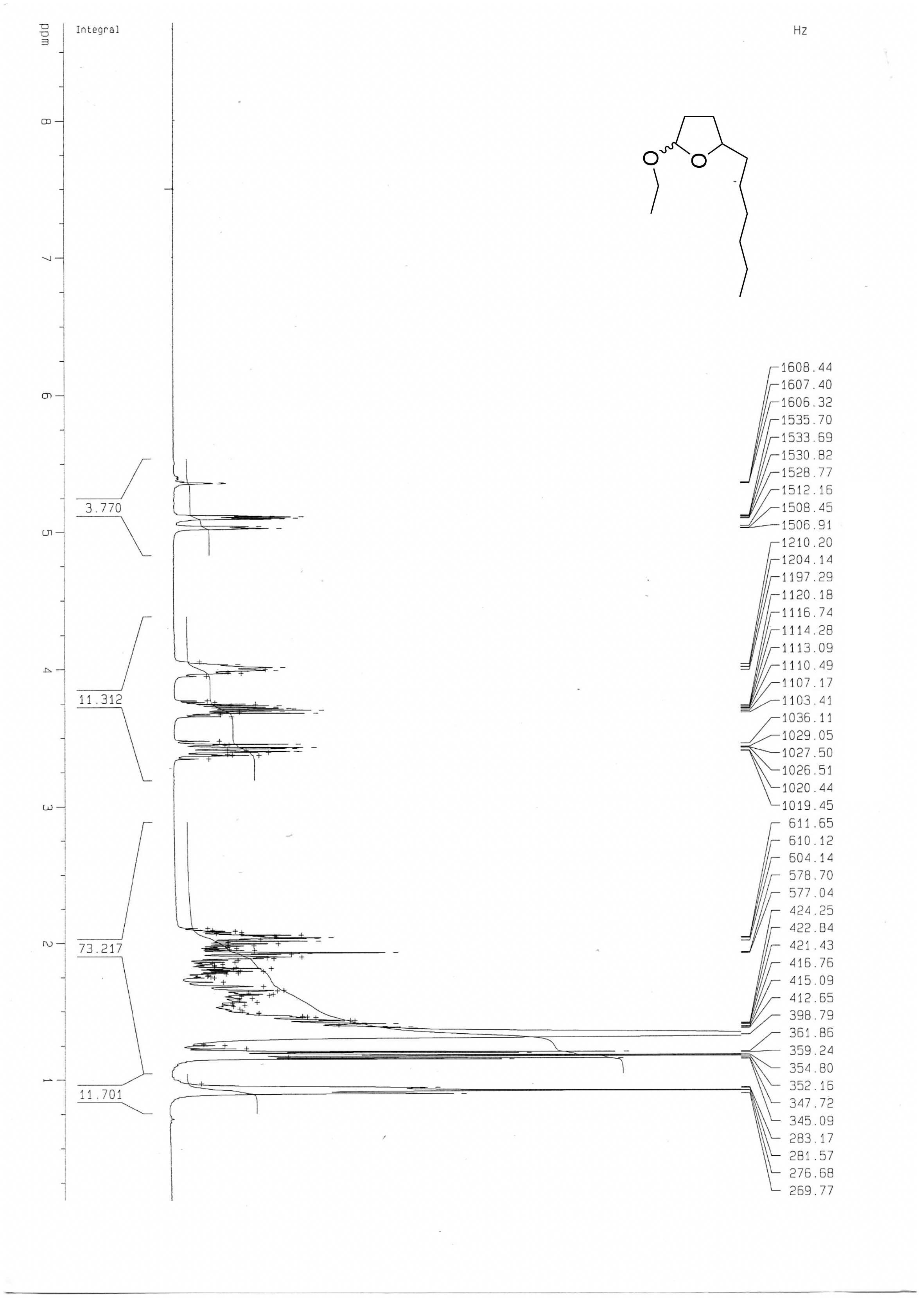

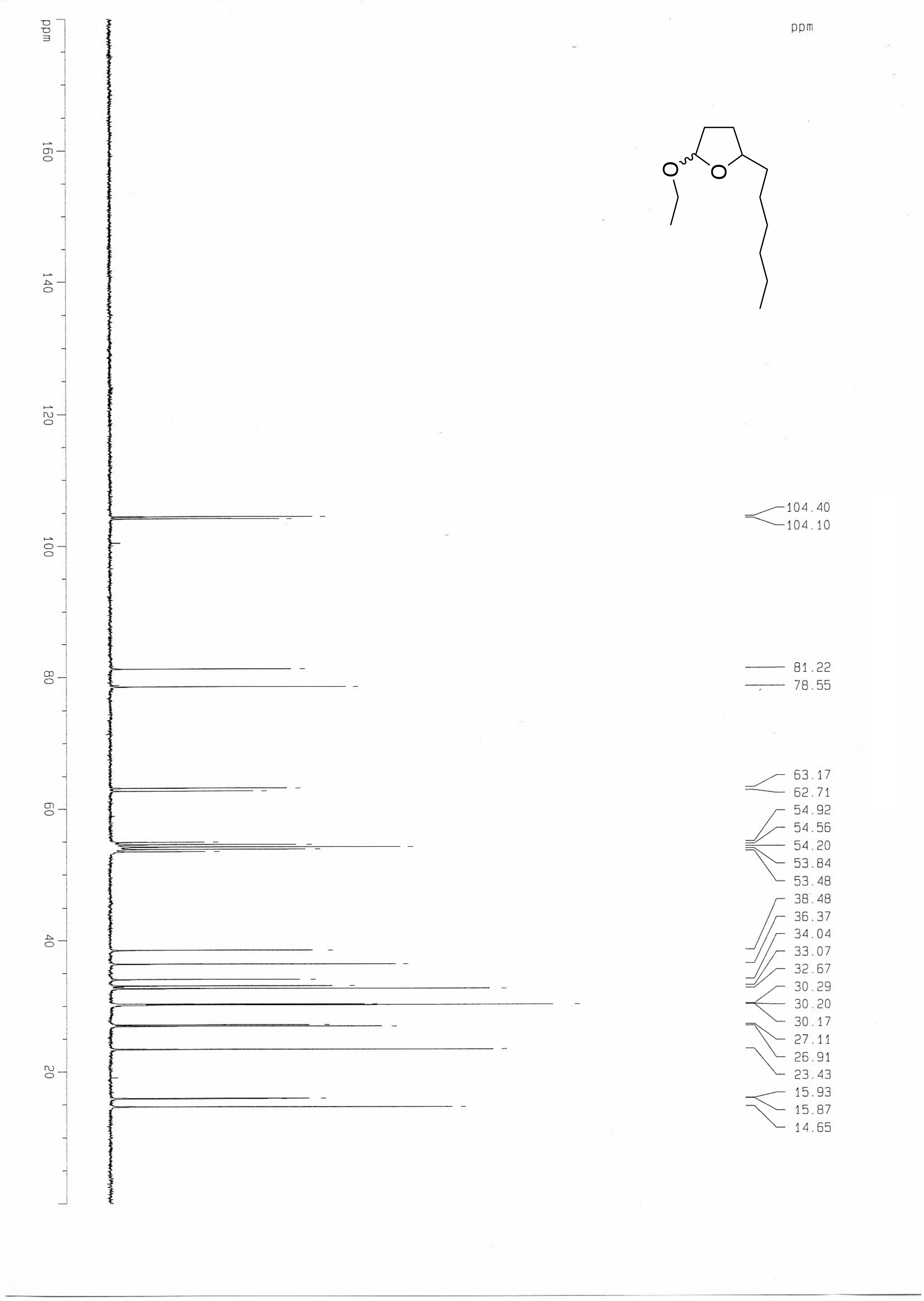
ppm
160
140
120
100
80
60
40
20
104.40
104.10
81.22
78.55
63.17
62.71
54.92
54.56
54.20
53.84
53.48
38.48
36.37
34.04
33.07
32.67
30.29
30.20
30.17
27.11
26.91
23.43
15.93
15.87
14.65

**$^{1}H$-$^{13}C\{^{1}H\}$ NMR Spectra (Compound 11, 400 MHz, $CDCl_3$)**

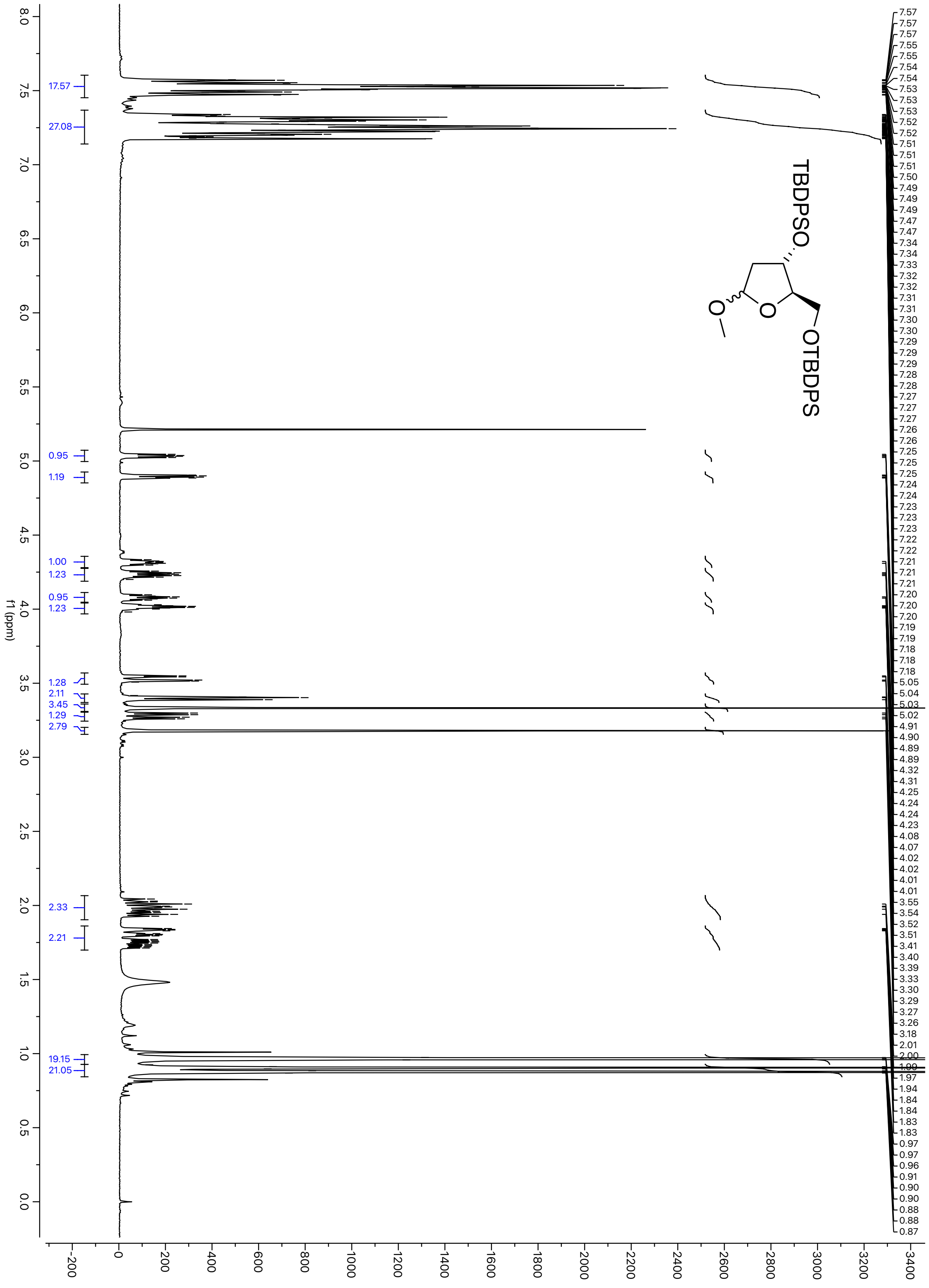

# $^{1}$H-$^{13}$C{$^{1}$H} NMR Spectra (Compound Ba, 300 MHz, $CD_2Cl_2$)

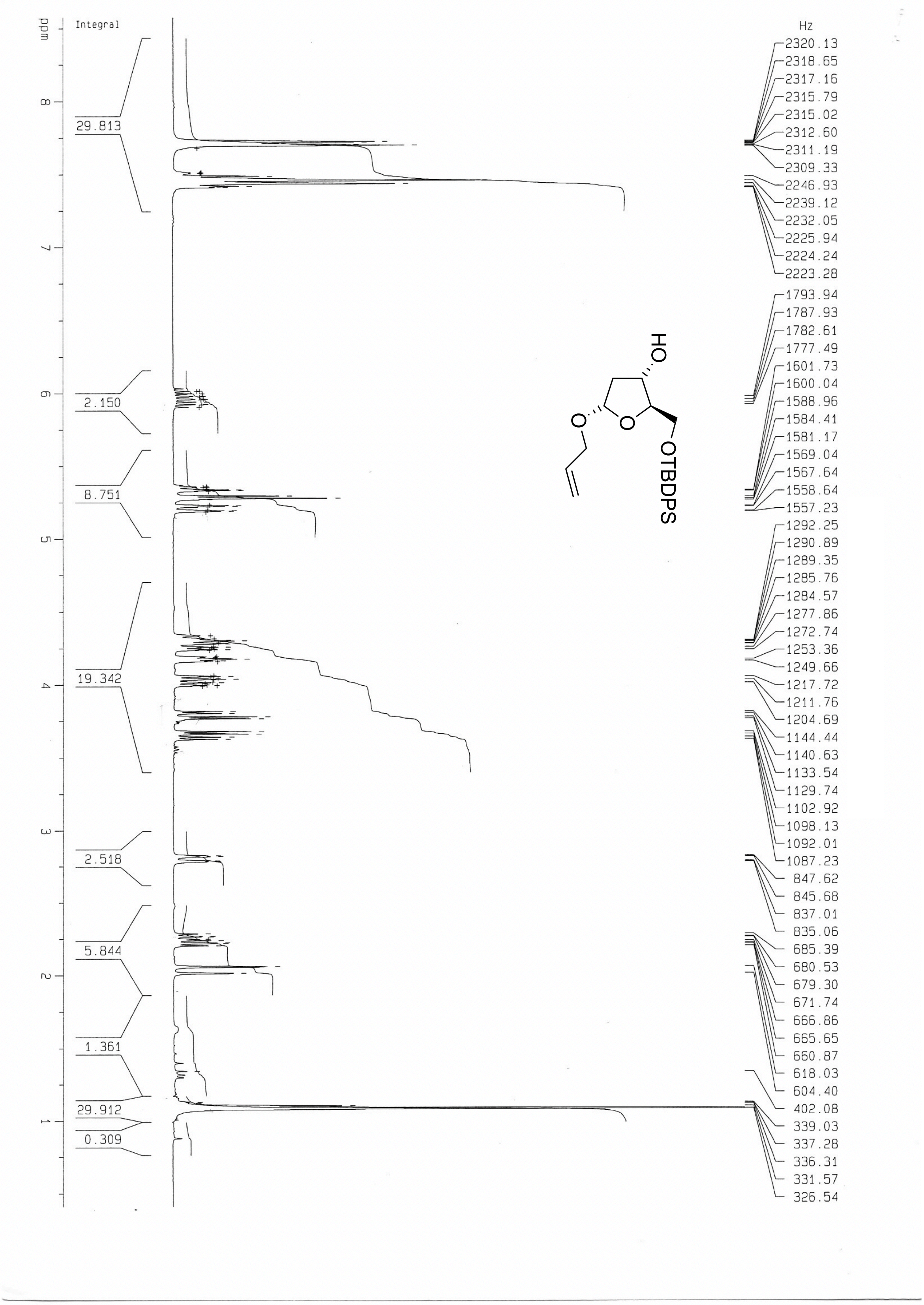

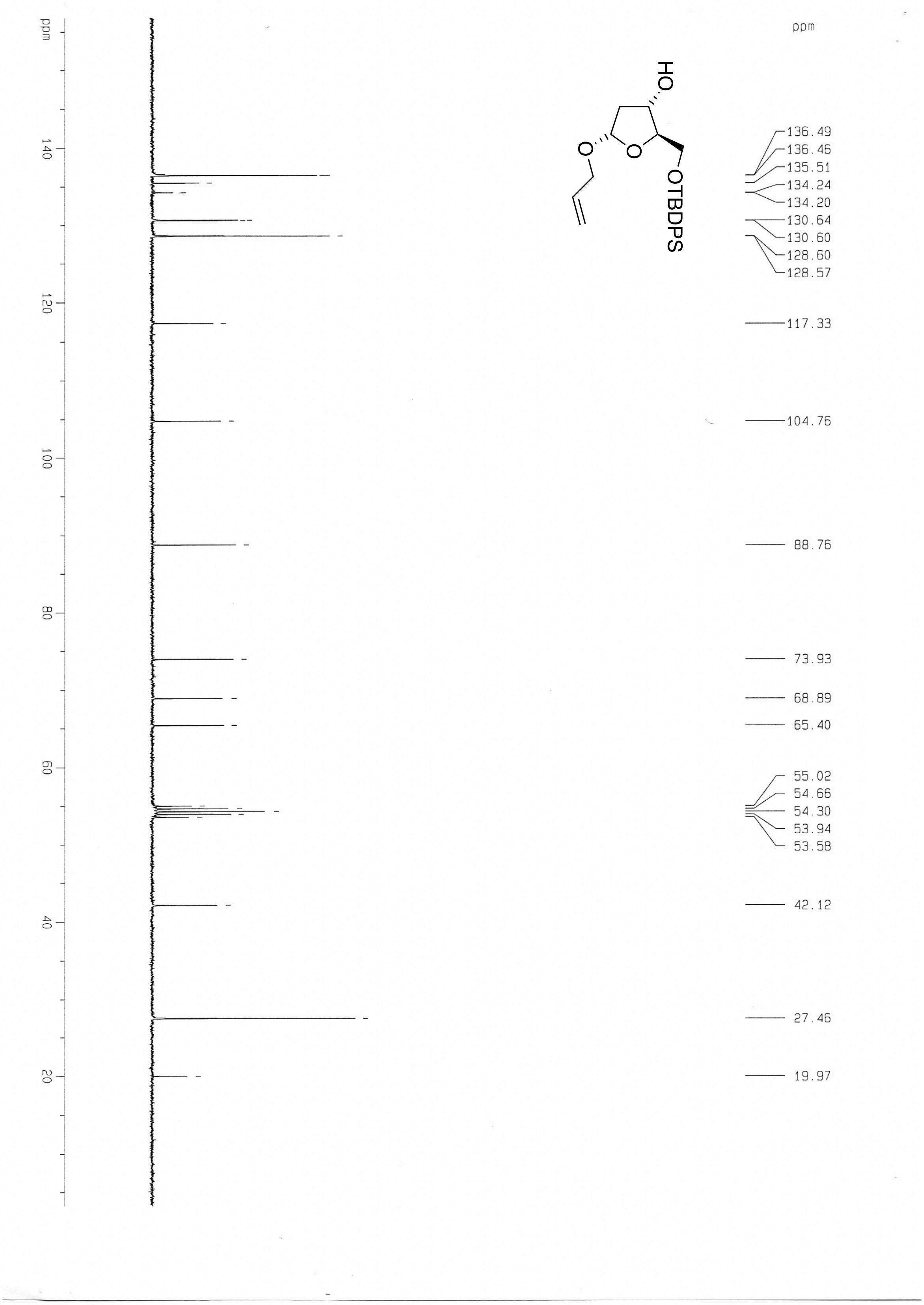
ppm
140
120
100
80
60
40
20
HO
O
OTBDPS
136.49
136.46
135.51
134.24
134.20
130.64
130.60
128.60
128.57
117.33
104.76
88.76
73.93
68.89
65.40
55.02
54.66
54.30
53.94
53.58
42.12
27.46
19.97

## $^{1}H$-$^{13}C\{^{1}H\}$ NMR Spectra (Compound Bb, 300 MHz, $CD_2Cl_2$)

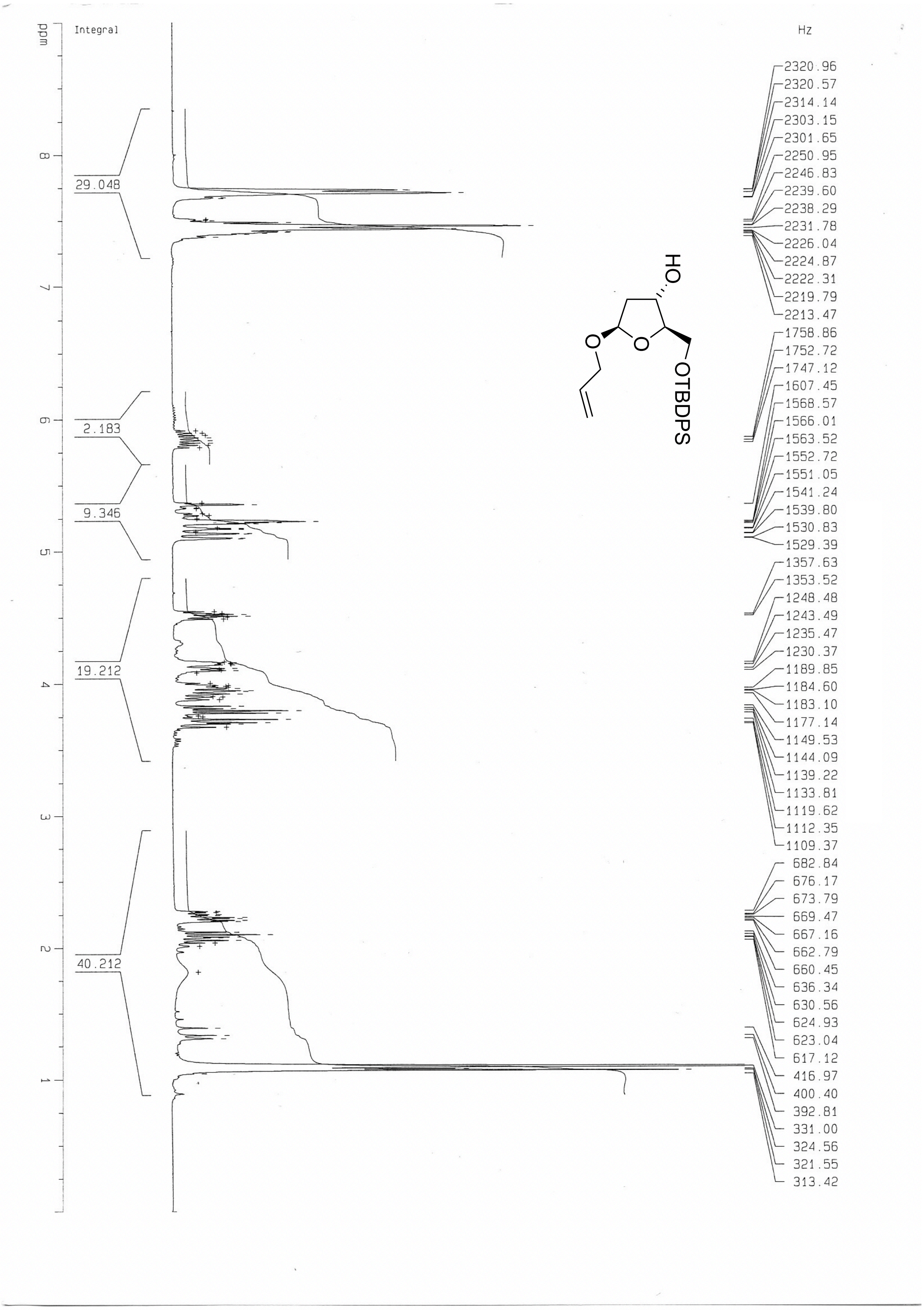

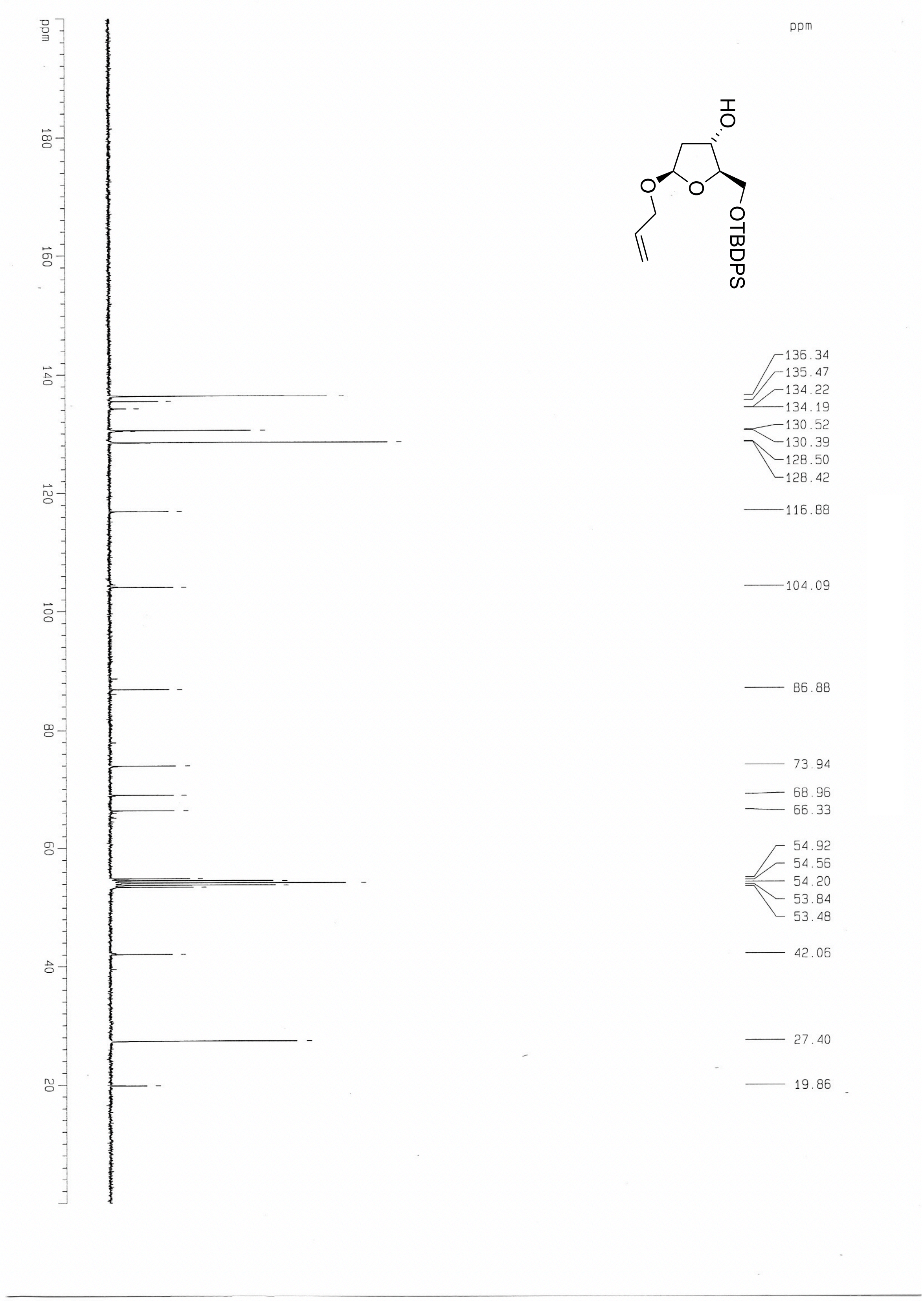
ppm
HO
O
O
OTBDPS
136.34
135.47
134.22
134.19
130.52
130.39
128.50
128.42
116.88
104.09
86.88
73.94
68.96
66.33
54.92
54.56
54.20
53.84
53.48
42.06
27.40
19.86
180
160
140
120
100
80
60
40
20
ppm

**$^{1}$H-$^{13}$C{$^{1}$H} NMR Spectra (Compound S30, 400 MHz, $CDCl_3$)**

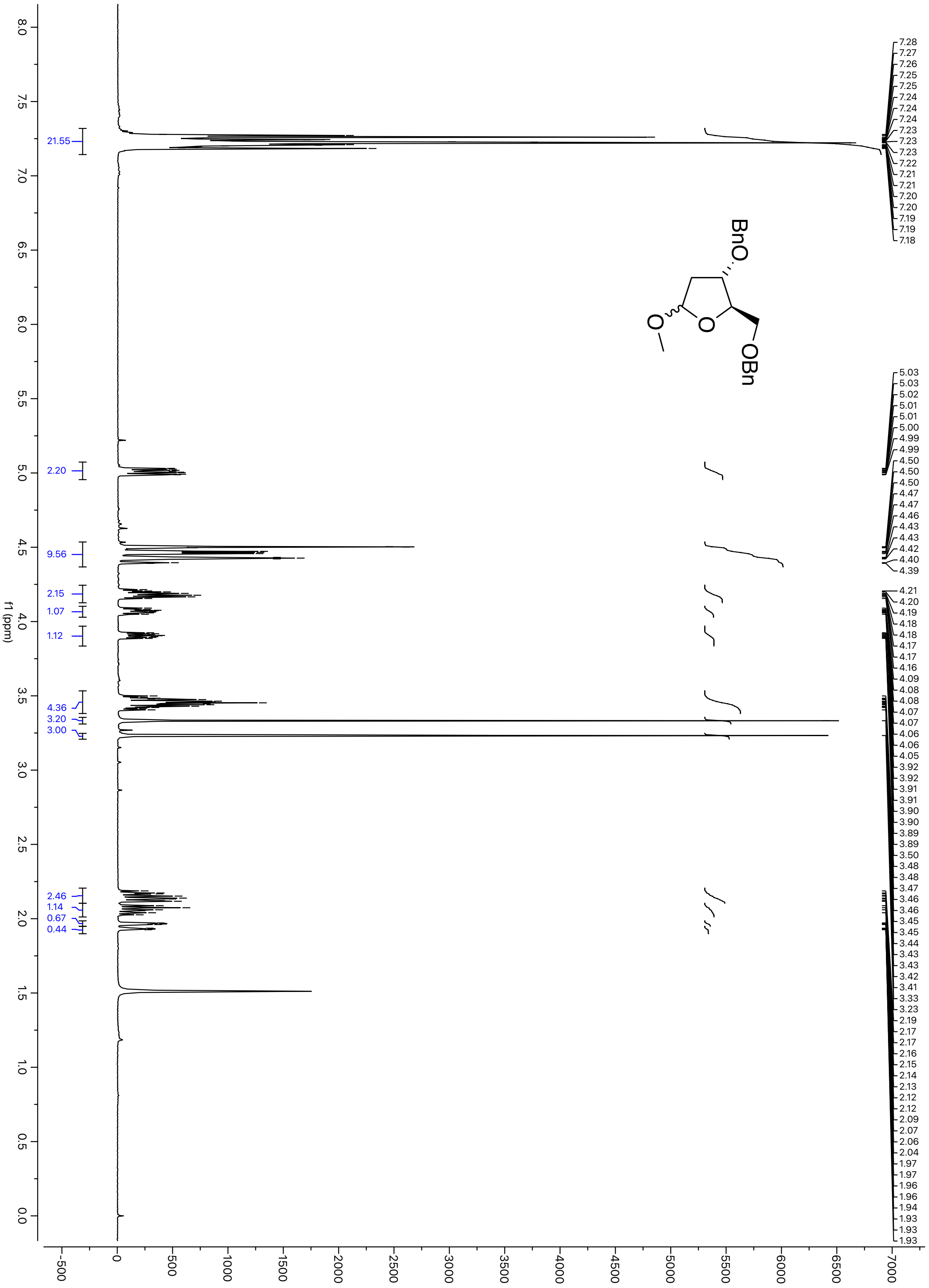

**$^1$H-$^{13}$C{$^1$H} NMR Spectra (Compound S31, 300 MHz, $CD_3CN$)**

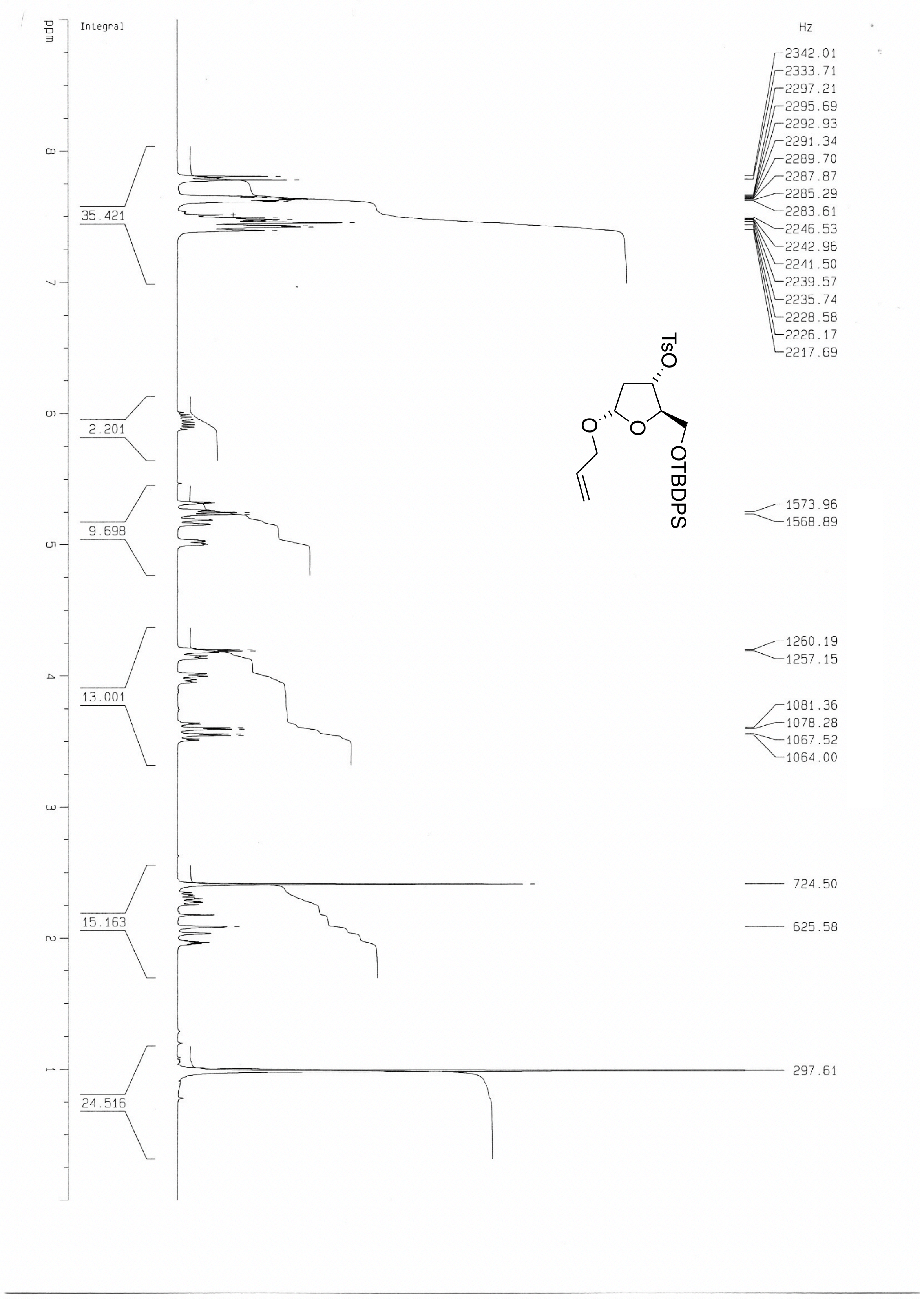

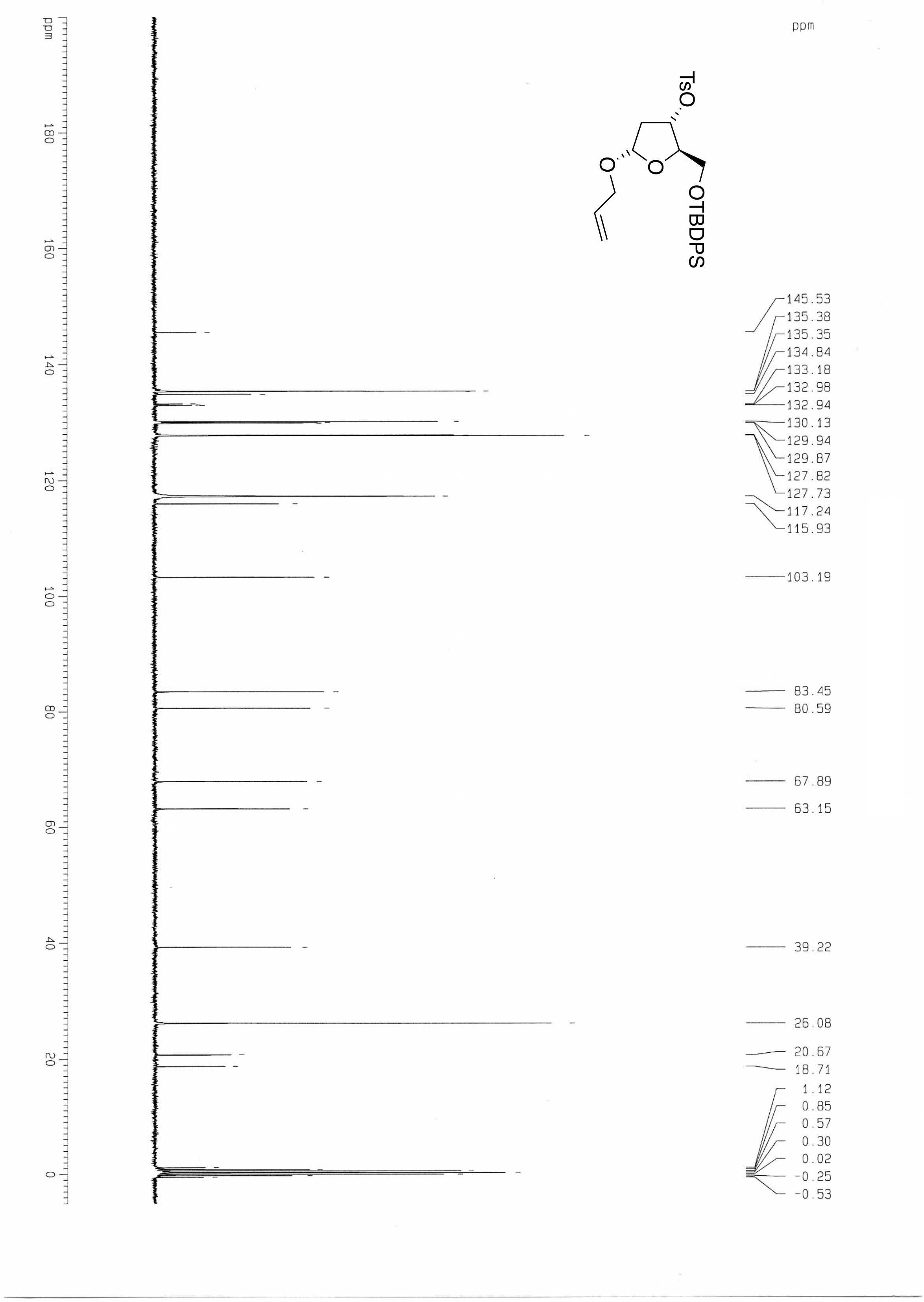
TsO
O
O
OTBDPS
ppm
145.53
135.38
135.35
134.84
133.18
132.98
132.94
130.13
129.94
129.87
127.82
127.73
117.24
115.93
103.19
83.45
80.59
67.89
63.15
39.22
26.08
20.67
18.71
1.12
0.85
0.57
0.30
0.02
-0.25
-0.53
ppm
180
160
140
120
100
80
60
40
20
0

**$^{1}$H-$^{13}$C{$^{1}$H} NMR Spectra (Compound C, 300 MHz, $CDCl_3$)**

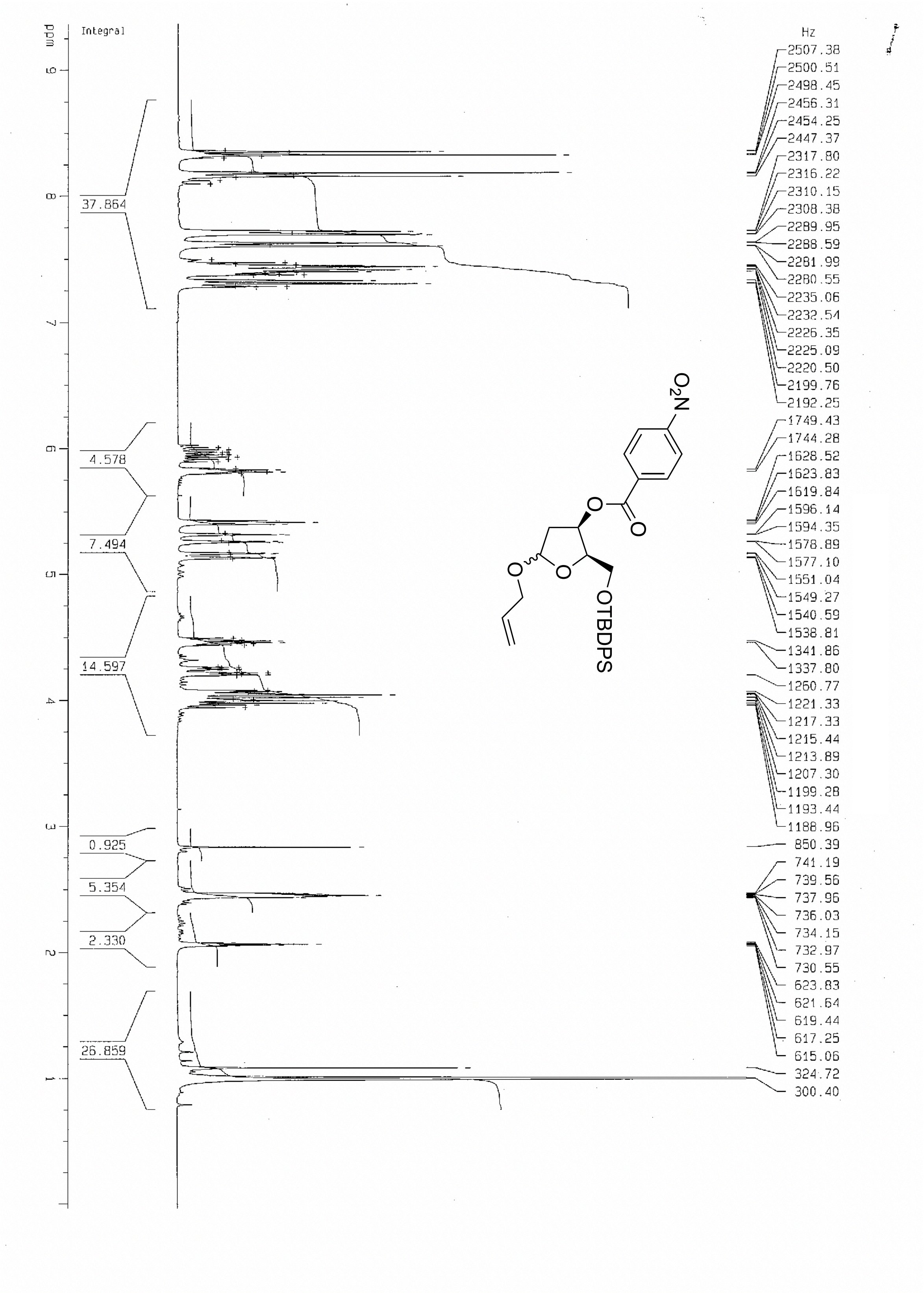

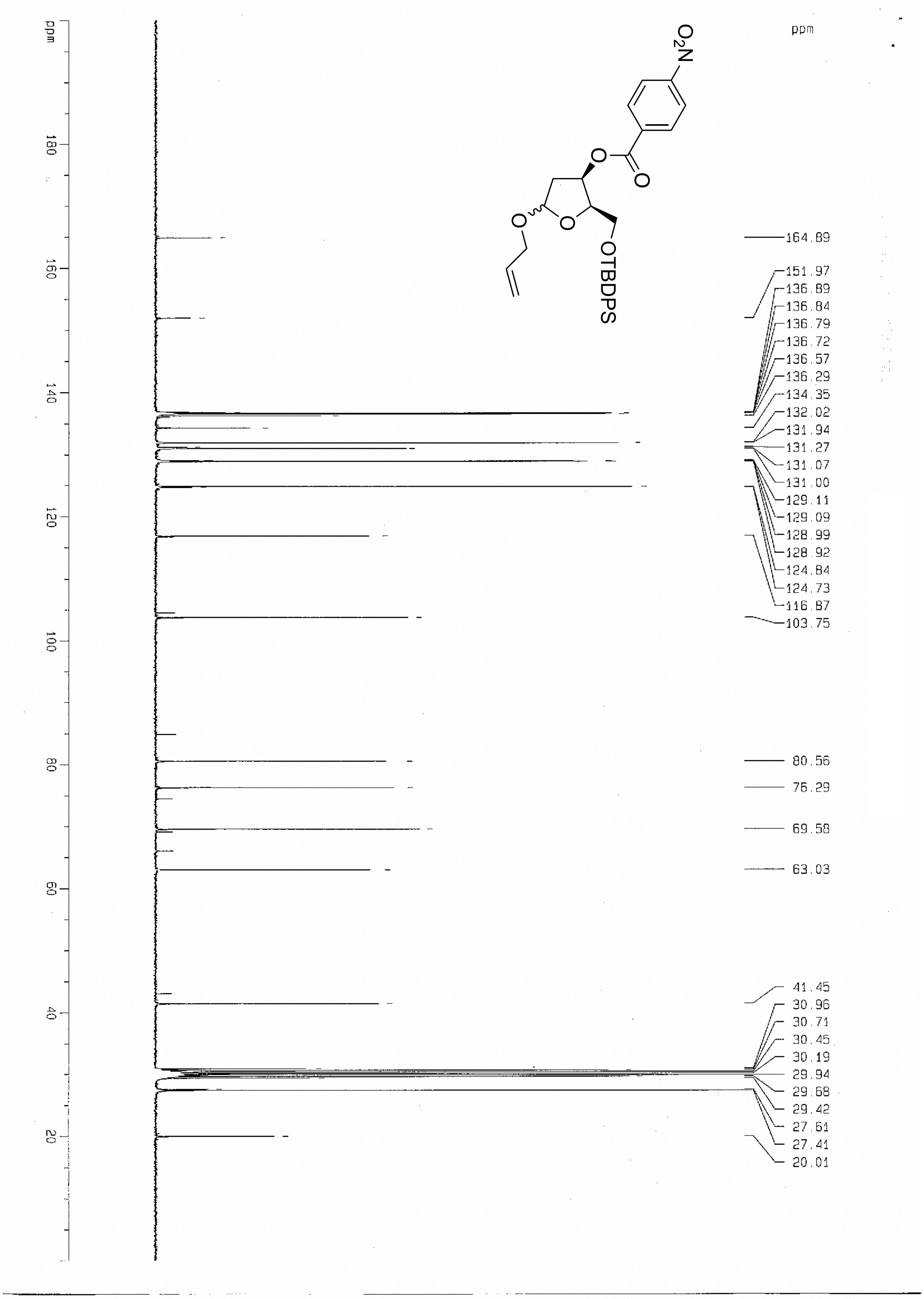

O2N
OTBDPS
ppm
164.89
151.97
136.89
136.84
136.79
136.72
136.57
136.29
134.35
132.02
131.94
131.27
131.07
131.00
129.11
129.09
128.99
128.92
124.84
124.73
116.87
103.75
80.56
76.29
69.58
63.03
41.45
30.96
30.71
30.45
30.19
29.94
29.68
29.42
27.61
27.41
20.01
180
160
140
120
100
80
60
40
20

## $^{1}$H-$^{13}$C{$^{1}$H} NMR Spectra (Compound D, 300 MHz, $(CD_3)_2CO$)

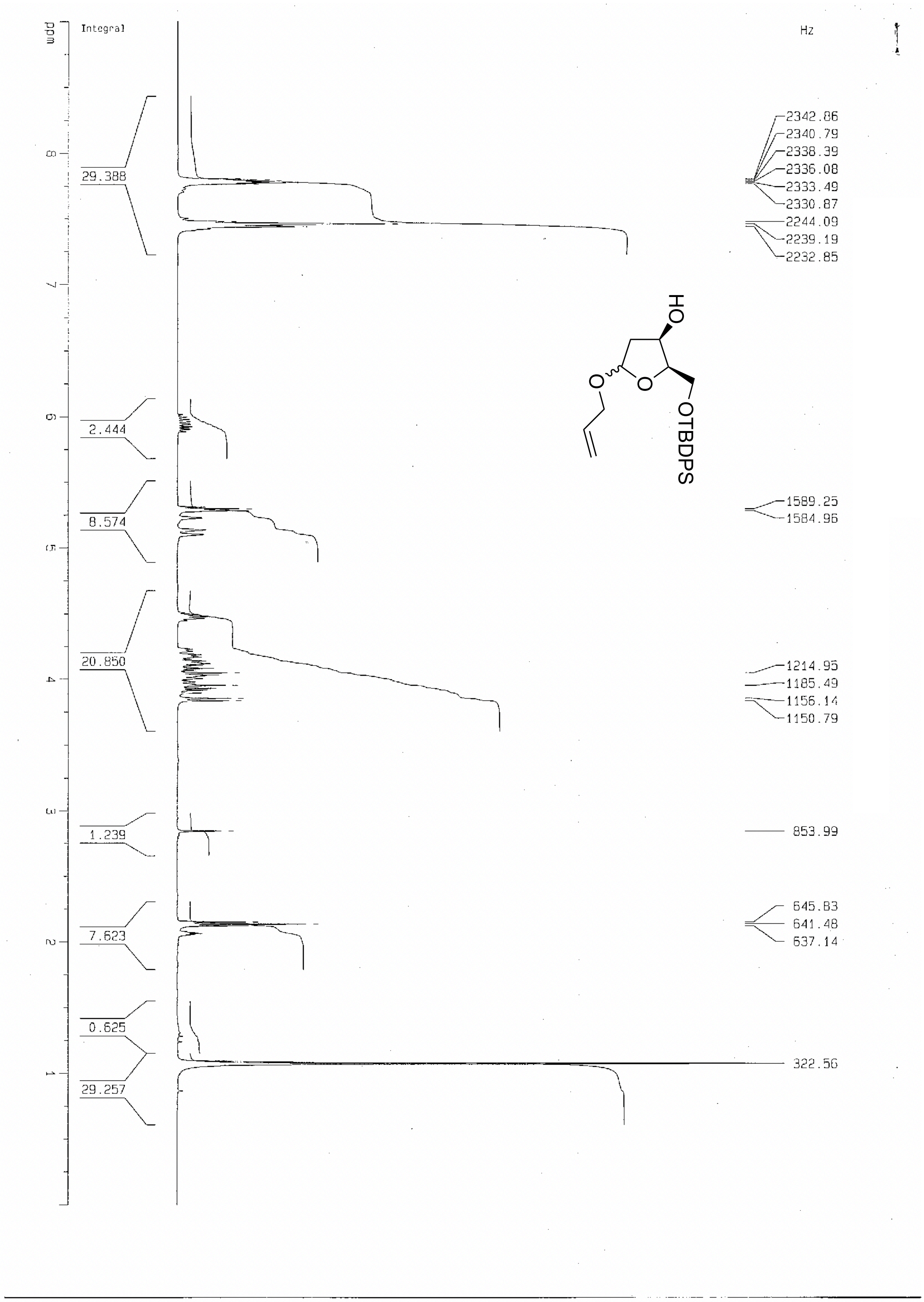

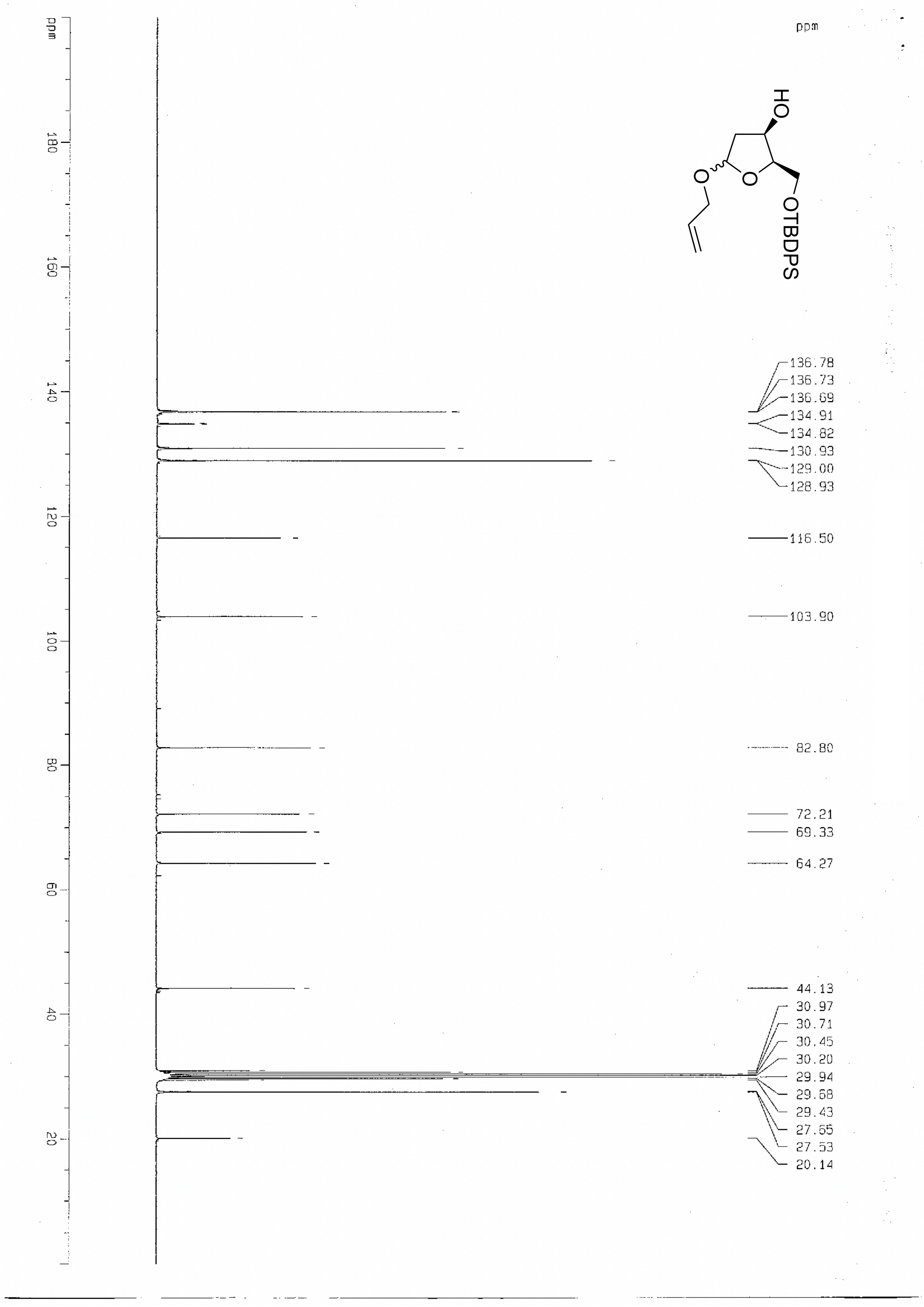
ppm
HO
O
O
OTBDPS
136.78
136.73
136.69
134.91
134.82
130.93
129.00
128.93
116.50
103.90
82.80
72.21
69.33
64.27
44.13
30.97
30.71
30.45
30.20
29.94
29.68
29.43
27.65
27.53
20.14

**$^{1}$H-$^{13}$C{$^{1}$H} NMR Spectra (Compound S32, 300 MHz, $(CD_3)_2CO$)**

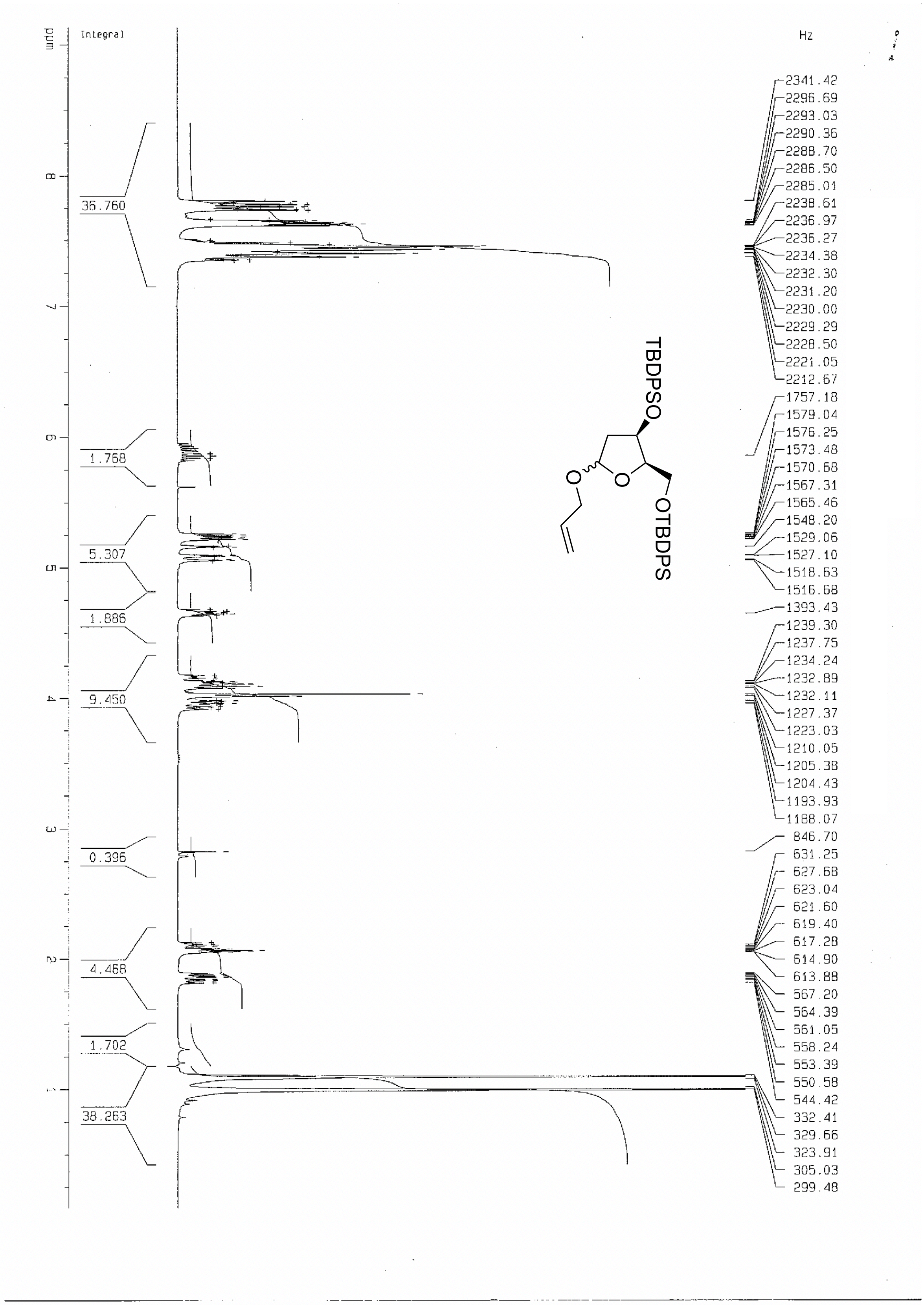

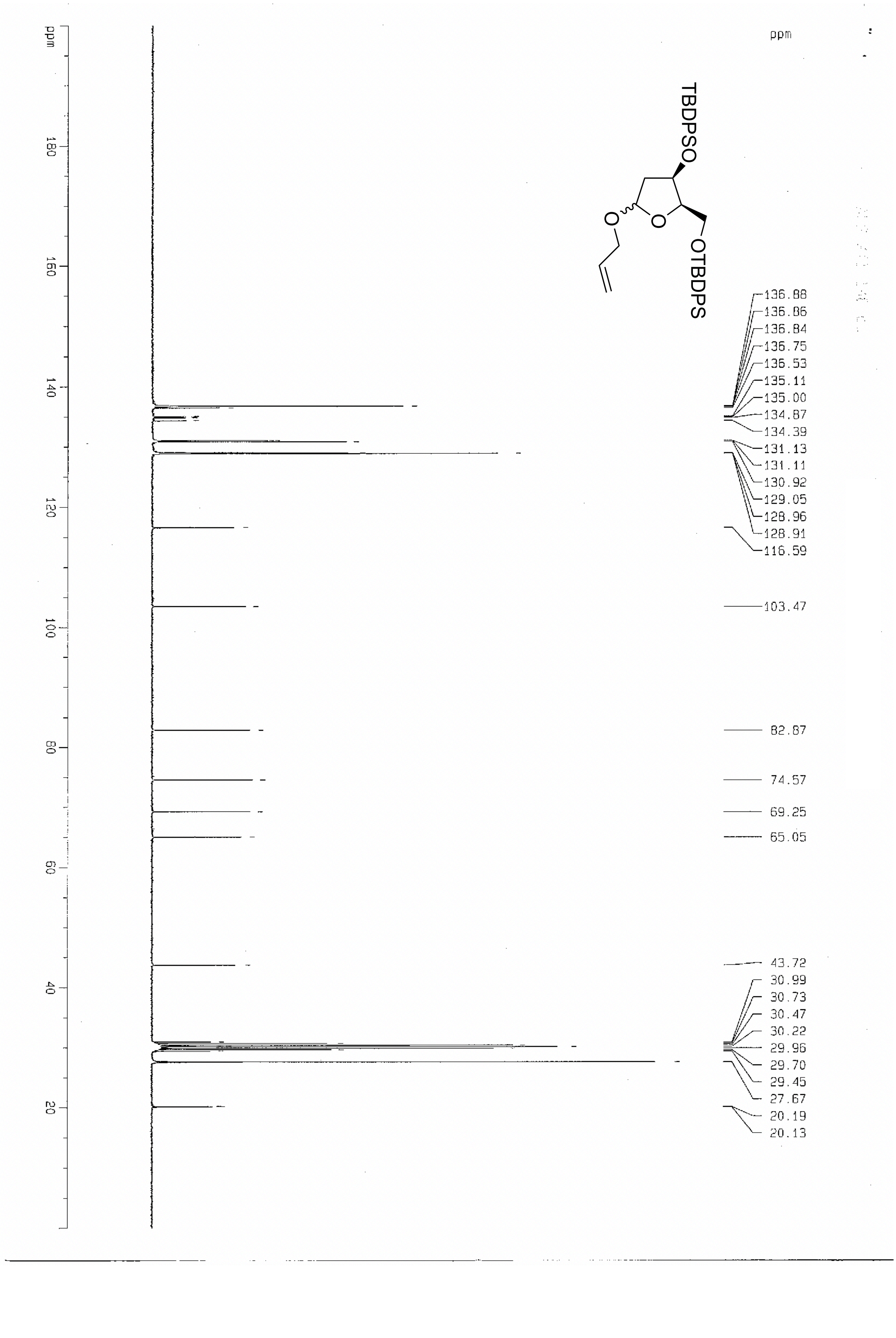
TBDPSO
OTBDPS
ppm
136.88
136.86
136.84
136.75
136.53
135.11
135.00
134.87
134.39
131.13
131.11
130.92
129.05
128.96
128.91
116.59
103.47
82.87
74.57
69.25
65.05
43.72
30.99
30.73
30.47
30.22
29.96
29.70
29.45
27.67
20.19
20.13

**$^{1}$H-$^{13}$C{$^{1}$H} NMR Spectra (Compound S33, 300 MHz, $CD_3CN$)**

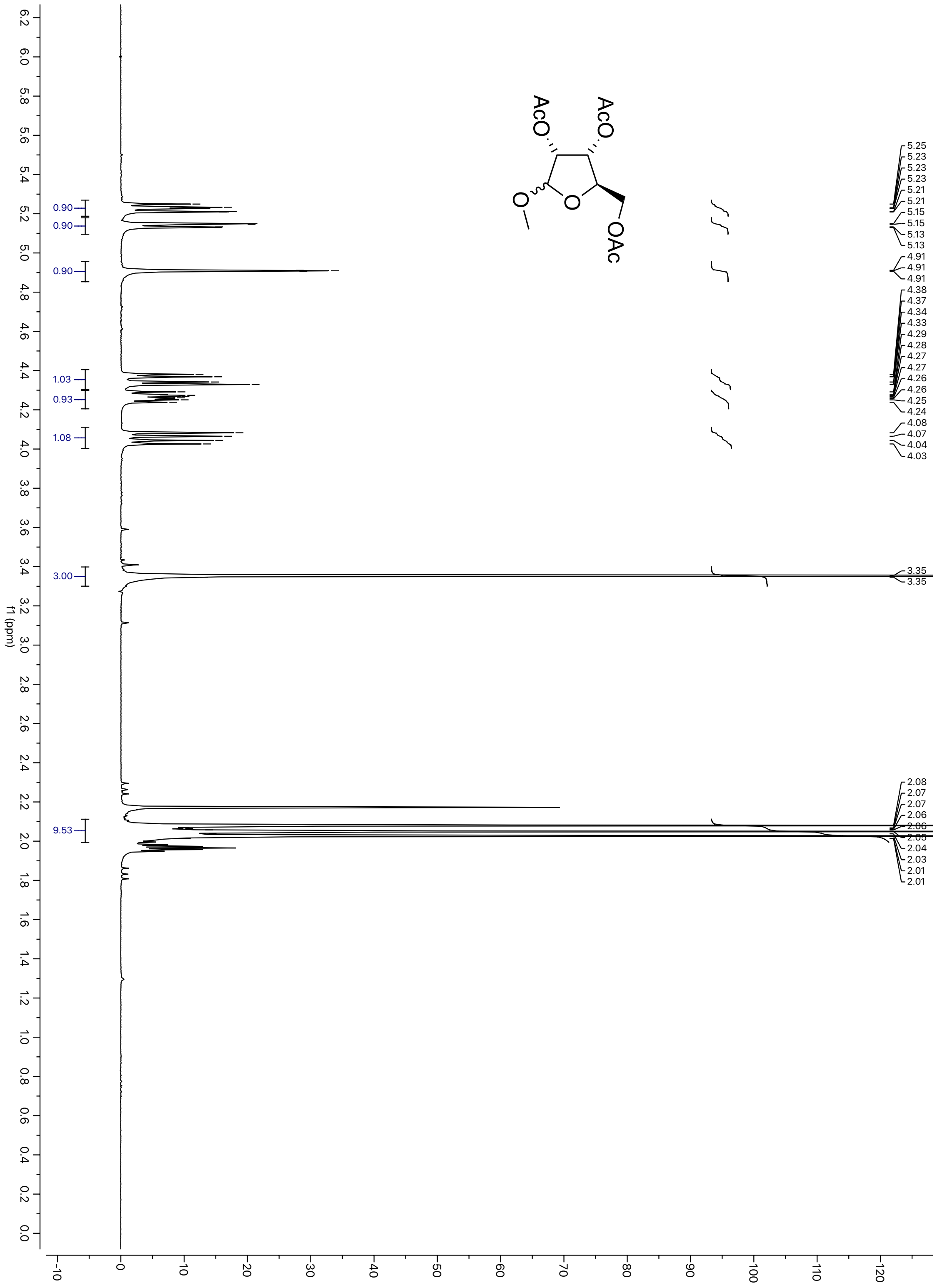

## $^{1}H$-$^{13}C\{^{1}H\}$ NMR Spectra (Compound S34, 400 MHz, $CDCl_3$)

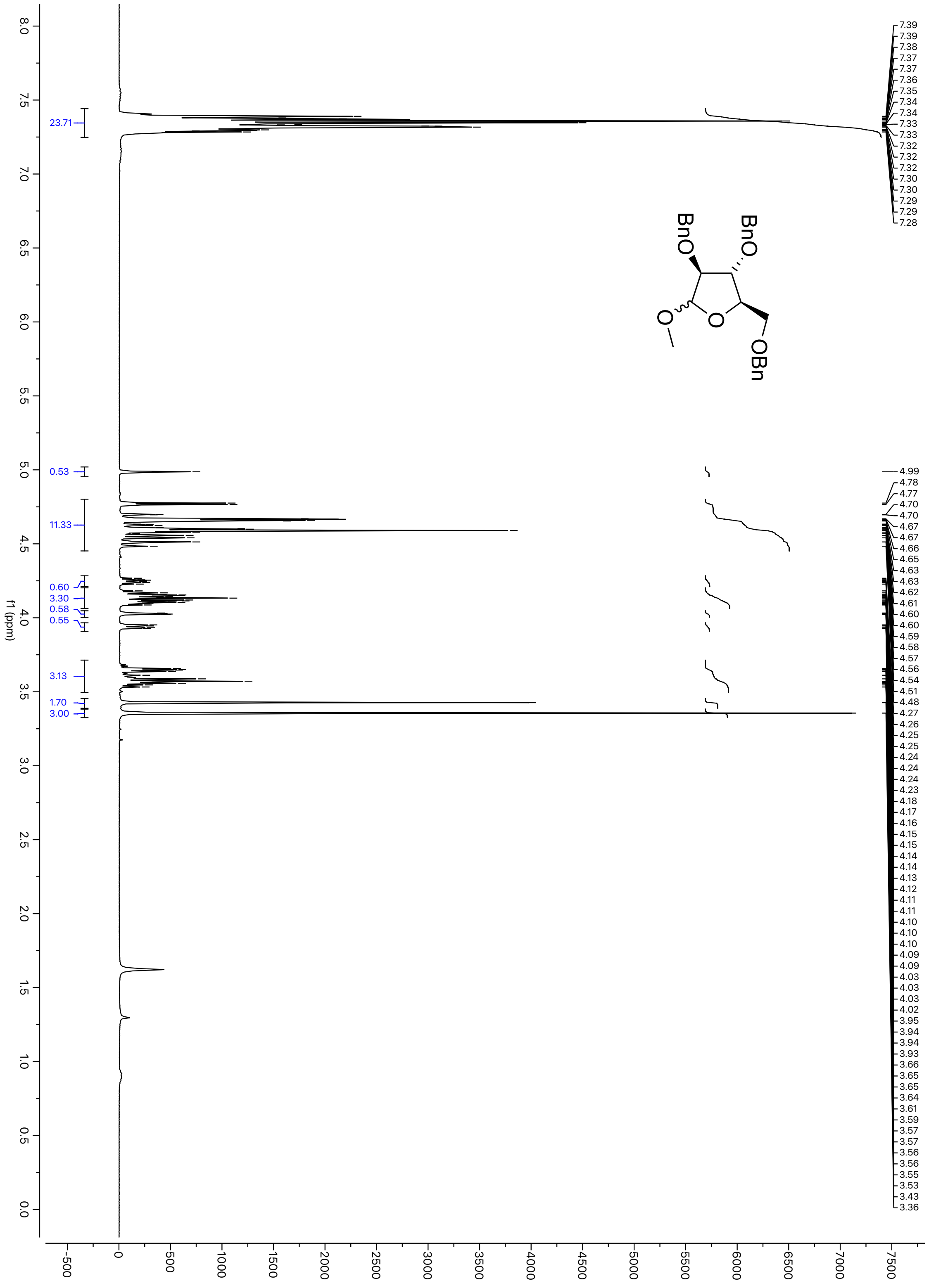

**$^{1}$H-$^{13}$C{$^{1}$H} NMR Spectra (Compound 10a and 10b, 300 MHz, $CDCl_3$)**

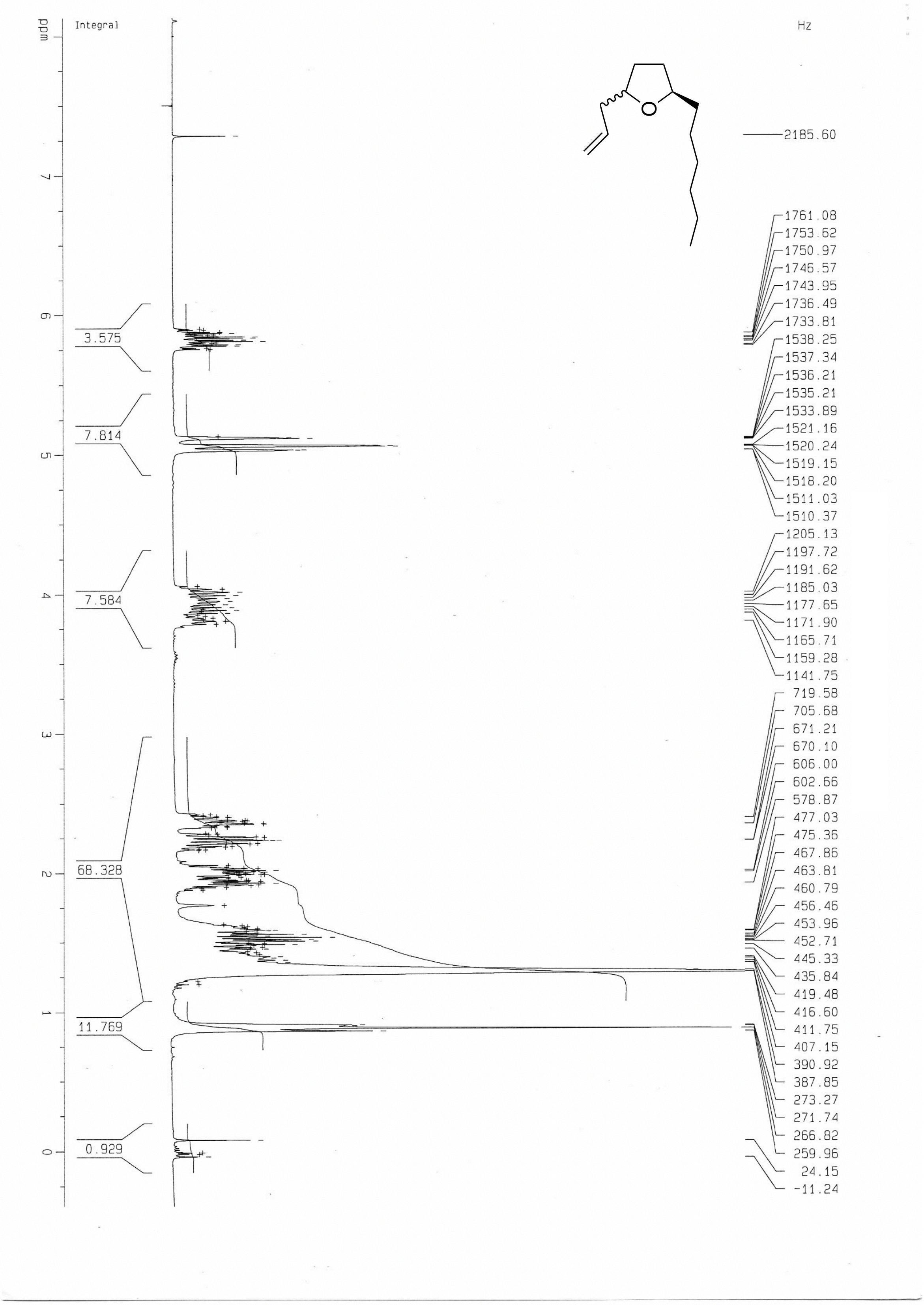

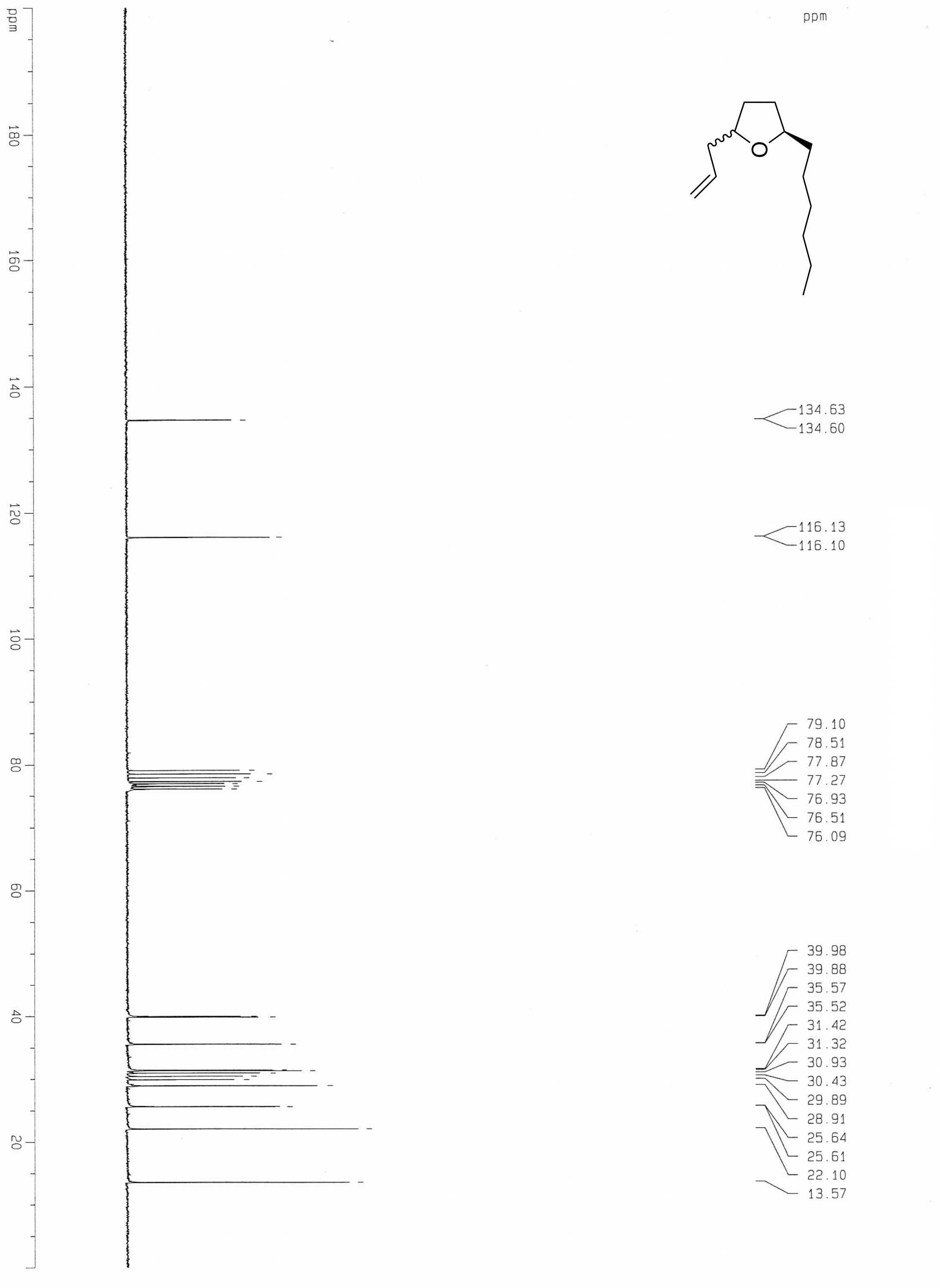
ppm
134.63
134.60
116.13
116.10
79.10
78.51
77.87
77.27
76.93
76.51
76.09
39.98
39.88
35.57
35.52
31.42
31.32
30.93
30.43
29.89
28.91
25.64
25.61
22.10
13.57
O

**$^{1}$H-$^{13}$C{$^{1}$H} NMR Spectra (Compound 12, 300 MHz, $CDCl_3$)**

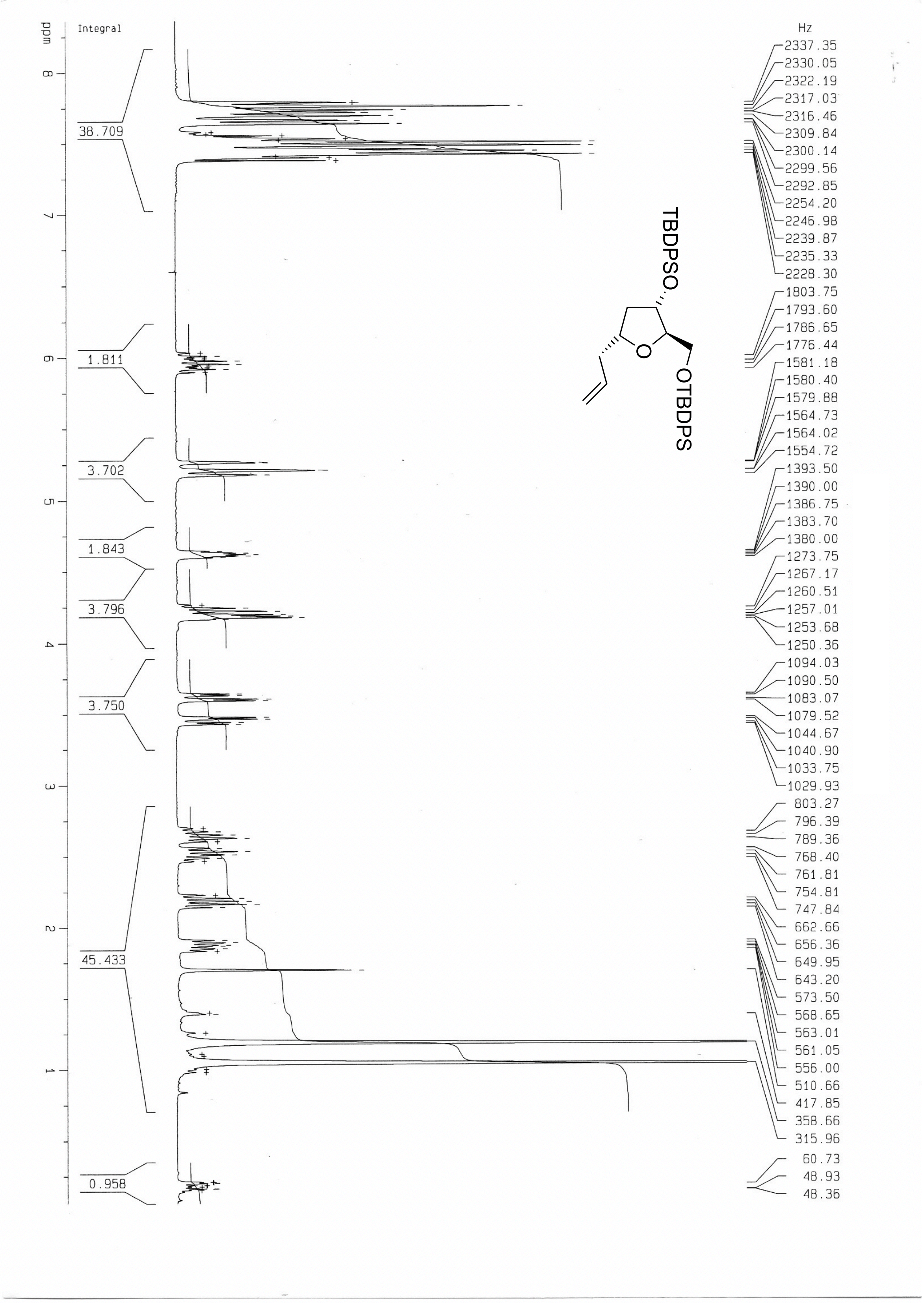

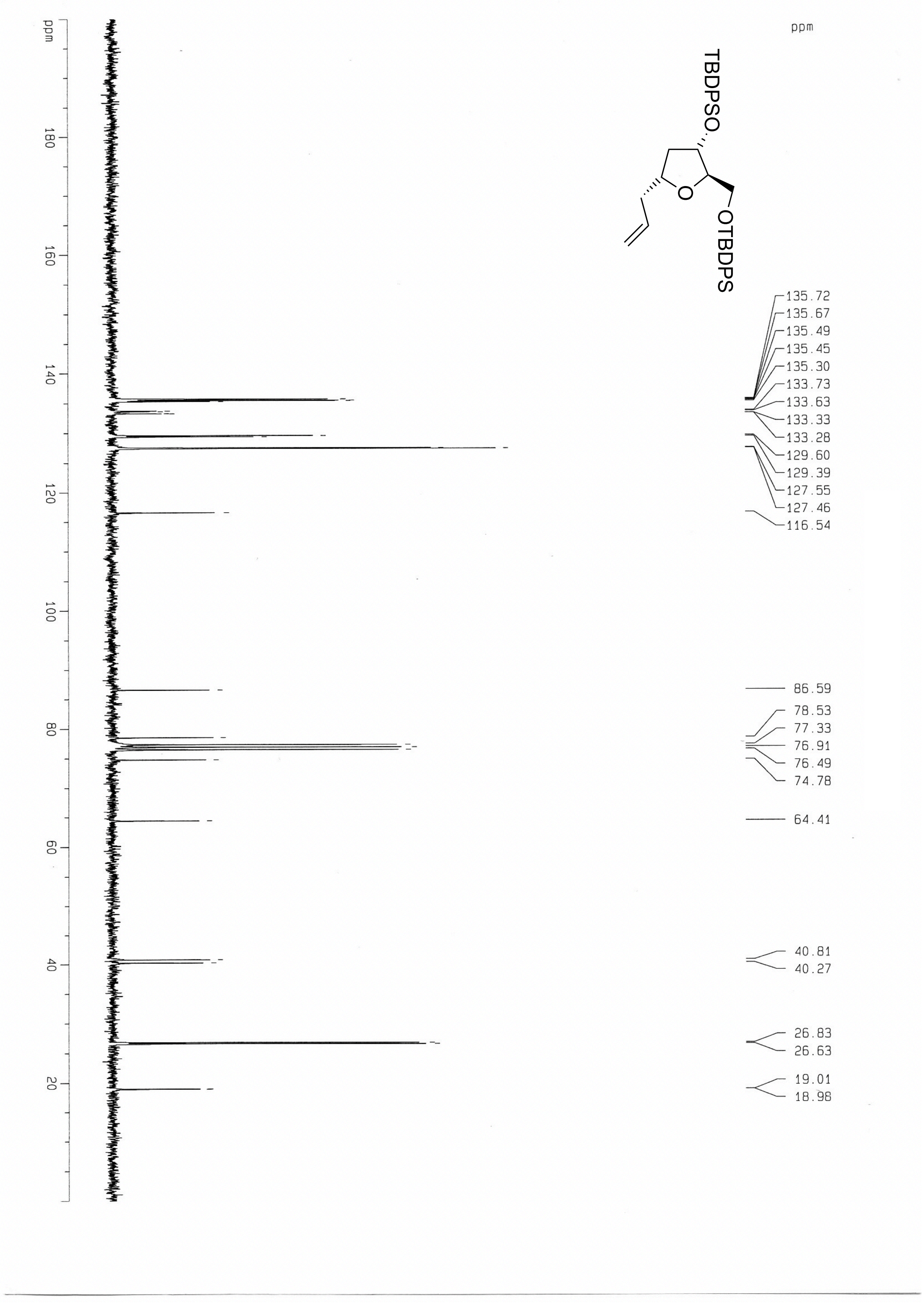
TBDPSO
OTBDPS
O
ppm
135.72
135.67
135.49
135.45
135.30
133.73
133.63
133.33
133.28
129.60
129.39
127.55
127.46
116.54
86.59
78.53
77.33
76.91
76.49
74.78
64.41
40.81
40.27
26.83
26.63
19.01
18.98
180
160
140
120
100
80
60
40
20

**$^{1}H$-$^{13}C\{^{1}H\}$ NMR Spectra (Compound 29, 300 MHz, $CDCl_3$)**

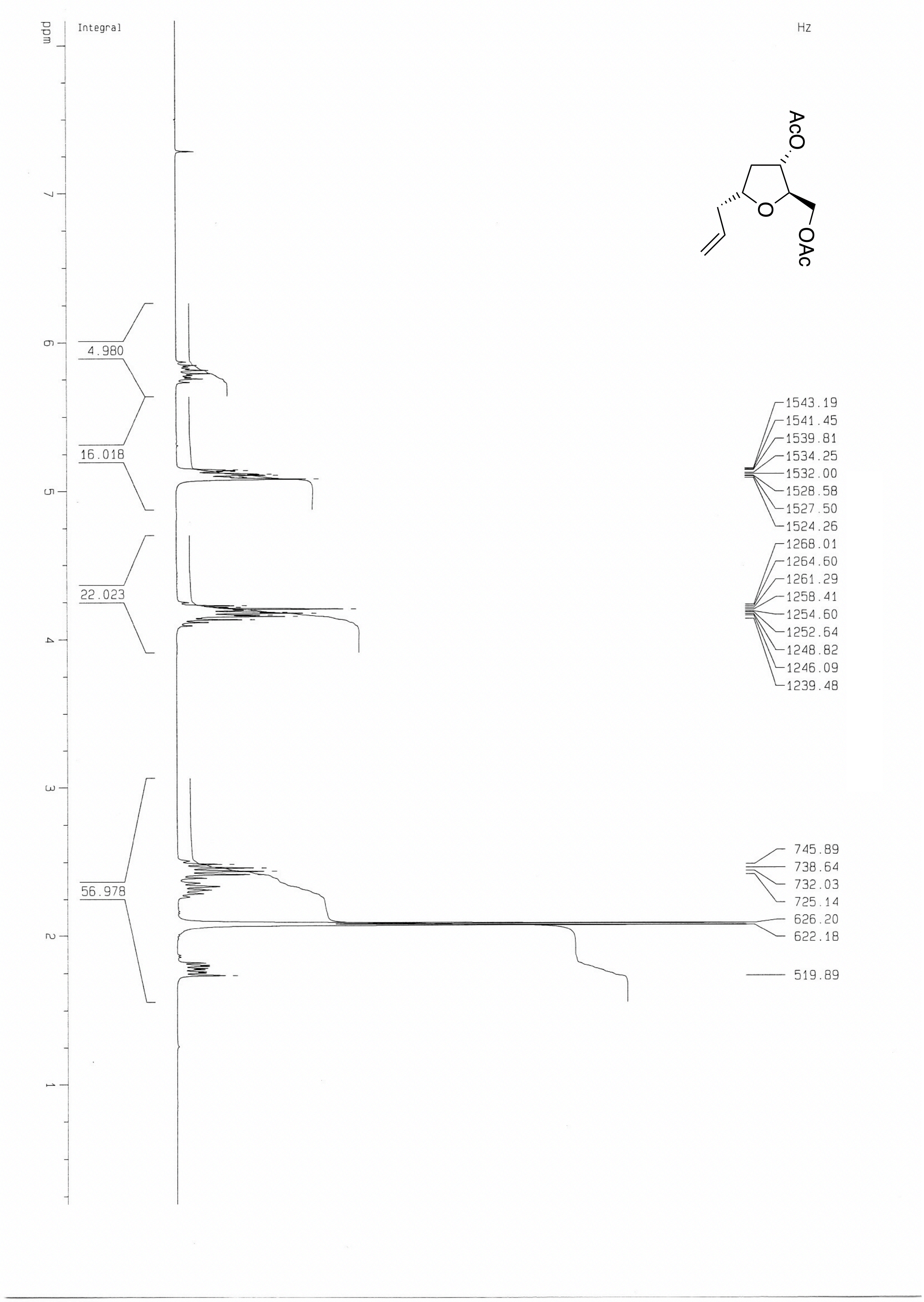

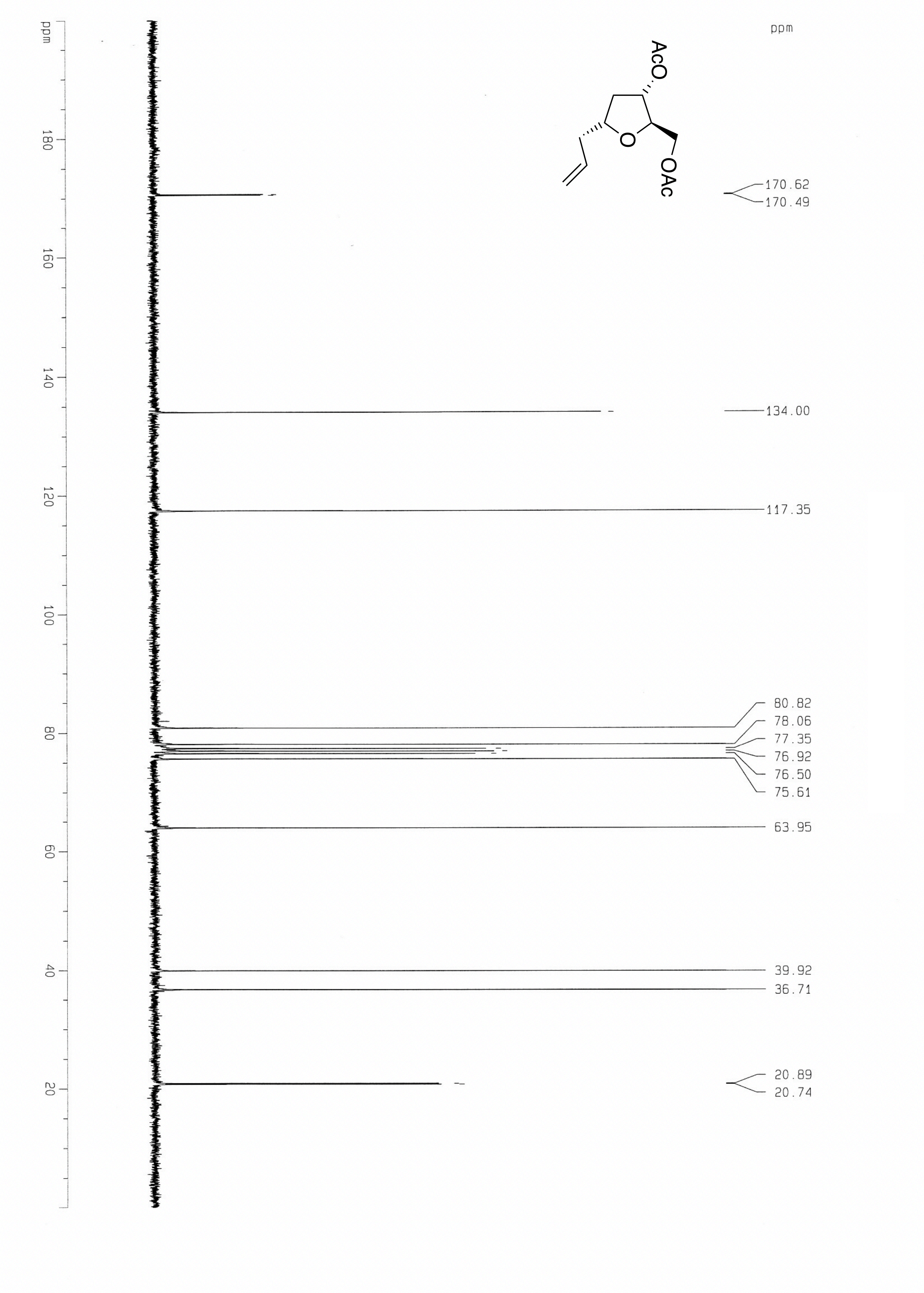
AcO
O
OAc
ppm
170.62
170.49
134.00
117.35
80.82
78.06
77.35
76.92
76.50
75.61
63.95
39.92
36.71
20.89
20.74
ppm
180
160
140
120
100
80
60
40
20

## $^{1}$H-$^{13}$C{$^{1}$H} NMR Spectra (Compound 30, 300 MHz, $CDCl_3$)

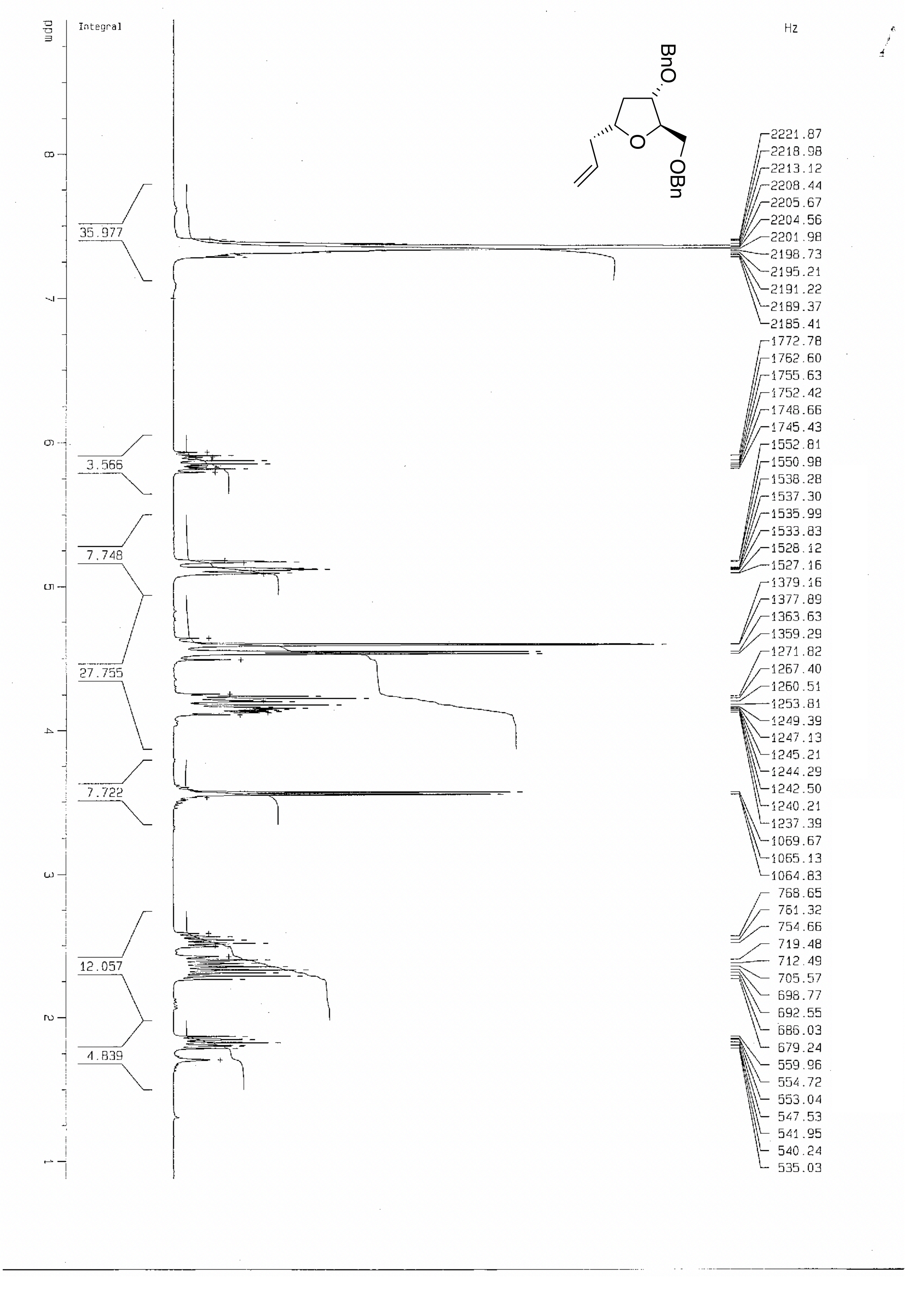

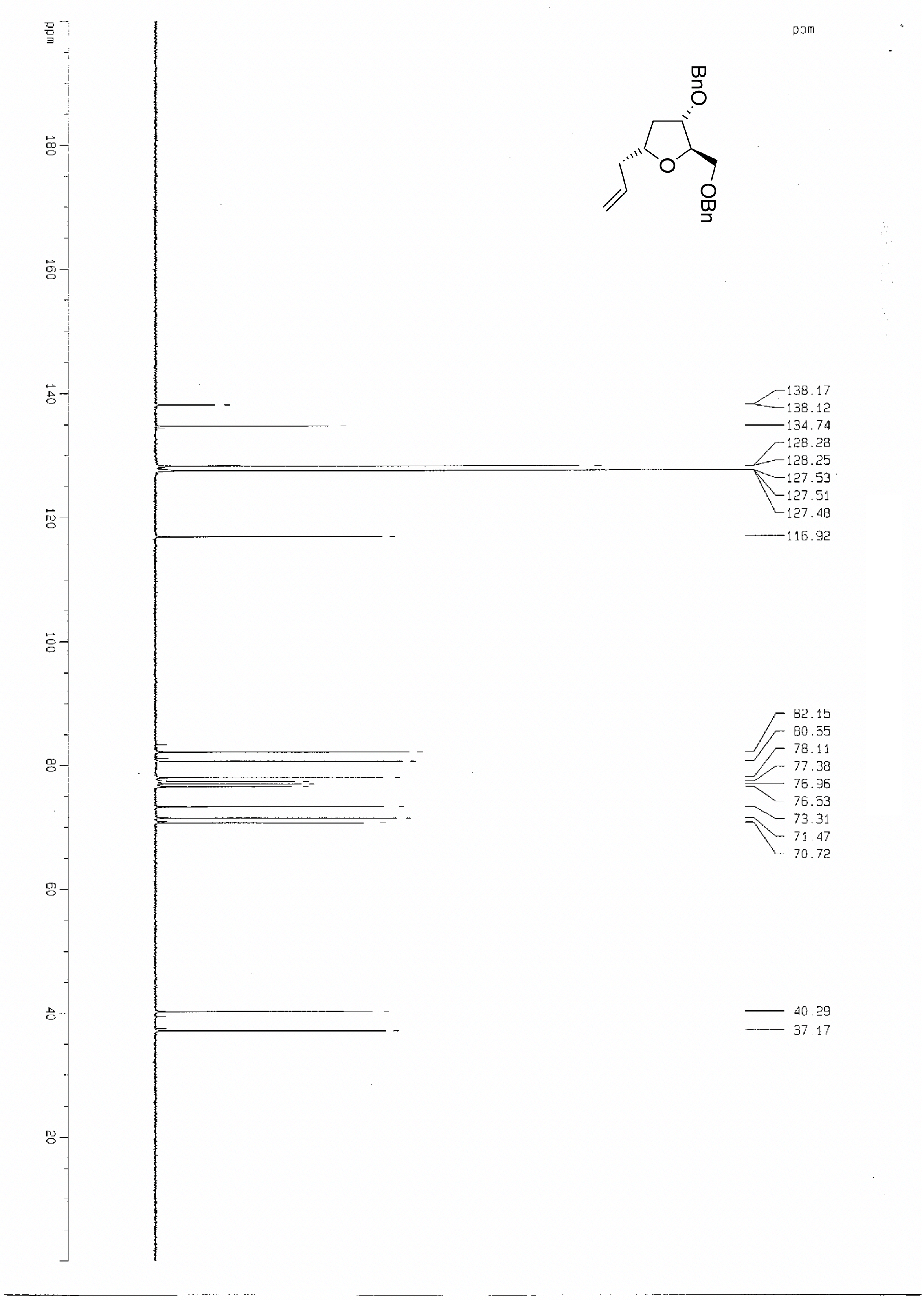
ppm
BnO
O
OBn
138.17
138.12
134.74
128.28
128.25
127.53
127.51
127.48
116.92
82.15
80.65
78.11
77.38
76.96
76.53
73.31
71.47
70.72
40.29
37.17
180
160
140
120
100
80
60
40
20
ppm

**$^{1}H$-$^{13}C\{^{1}H\}$ NMR Spectra (Compound 31, 300 MHz, $CDCl_3$)**

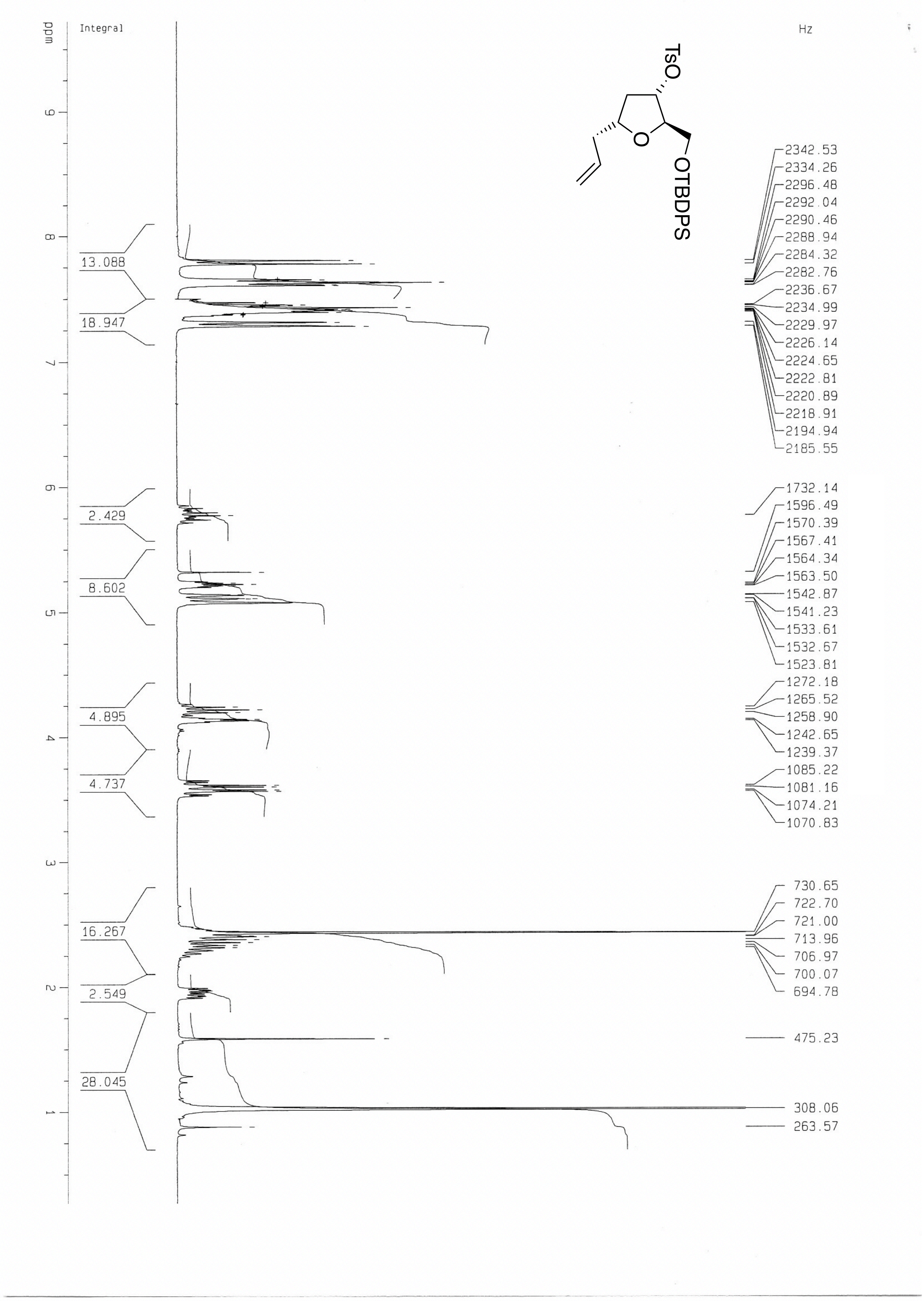

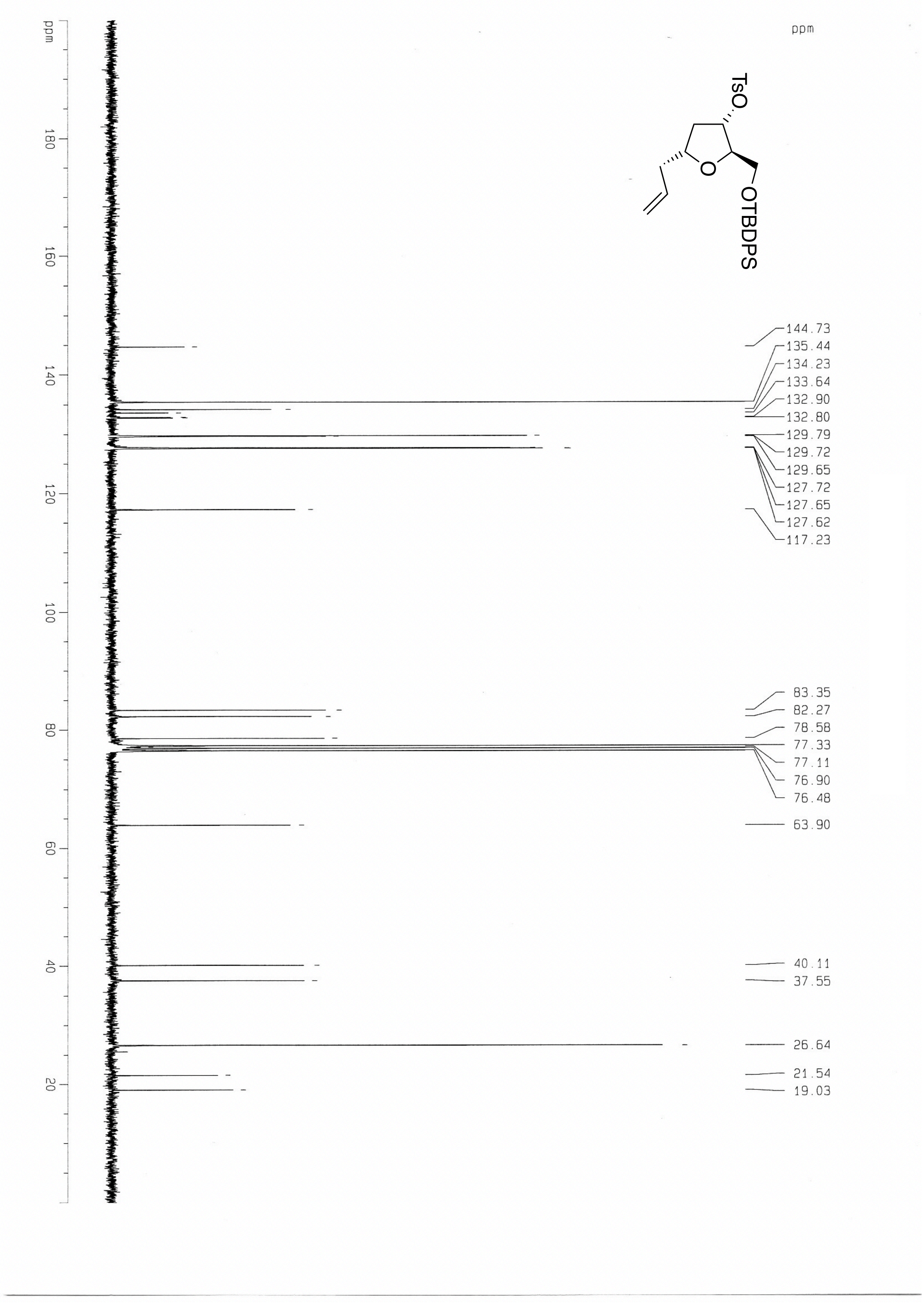
ppm
TsO
OTBDPS
144.73
135.44
134.23
133.64
132.90
132.80
129.79
129.72
129.65
127.72
127.65
127.62
117.23
83.35
82.27
78.58
77.33
77.11
76.90
76.48
63.90
40.11
37.55
26.64
21.54
19.03
ppm
180
160
140
120
100
80
60
40
20

**$^{1}$H-$^{13}$C{$^{1}$H} NMR Spectra (Compound 32, 300 MHz, $CD_2Cl_2$)**

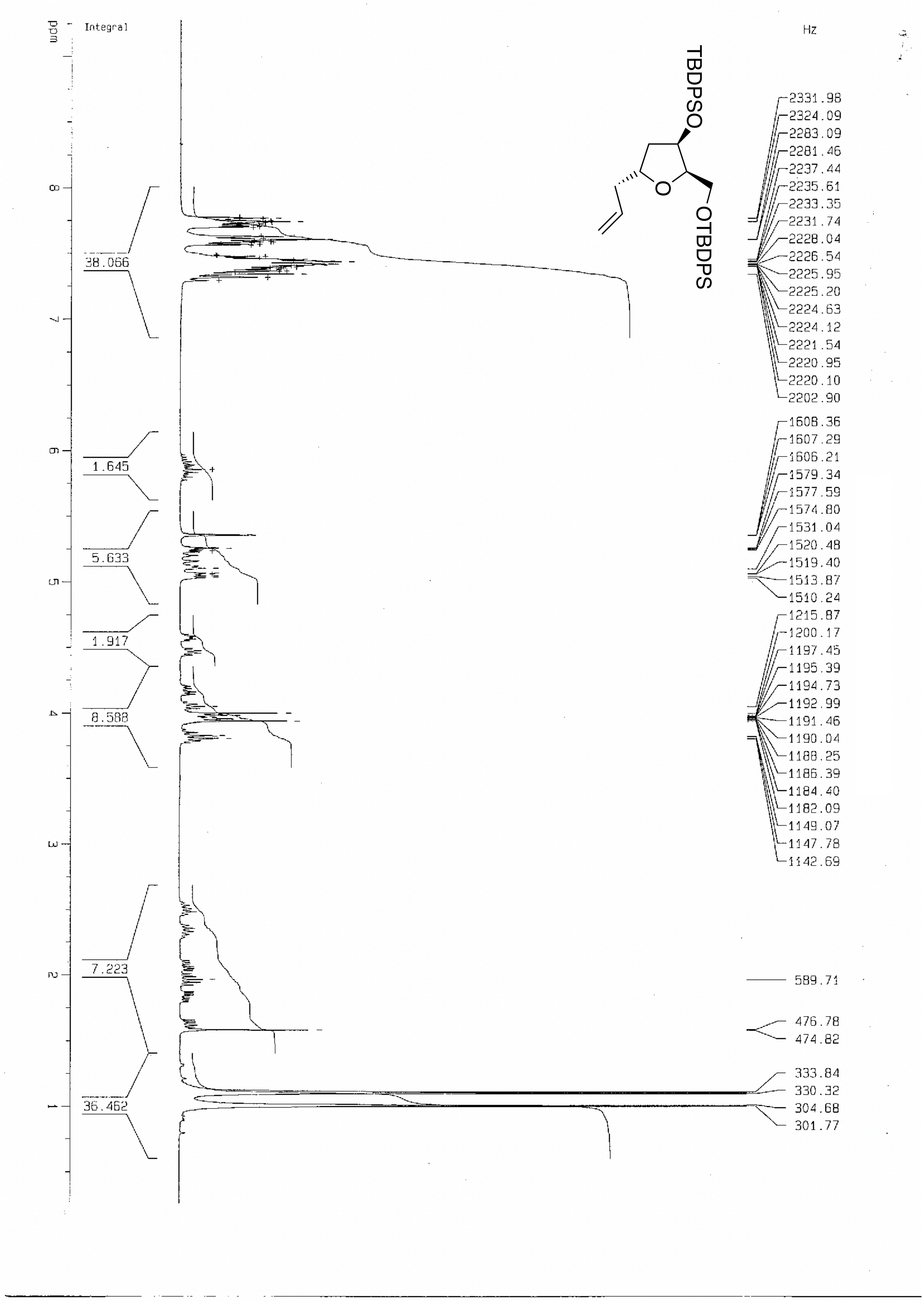

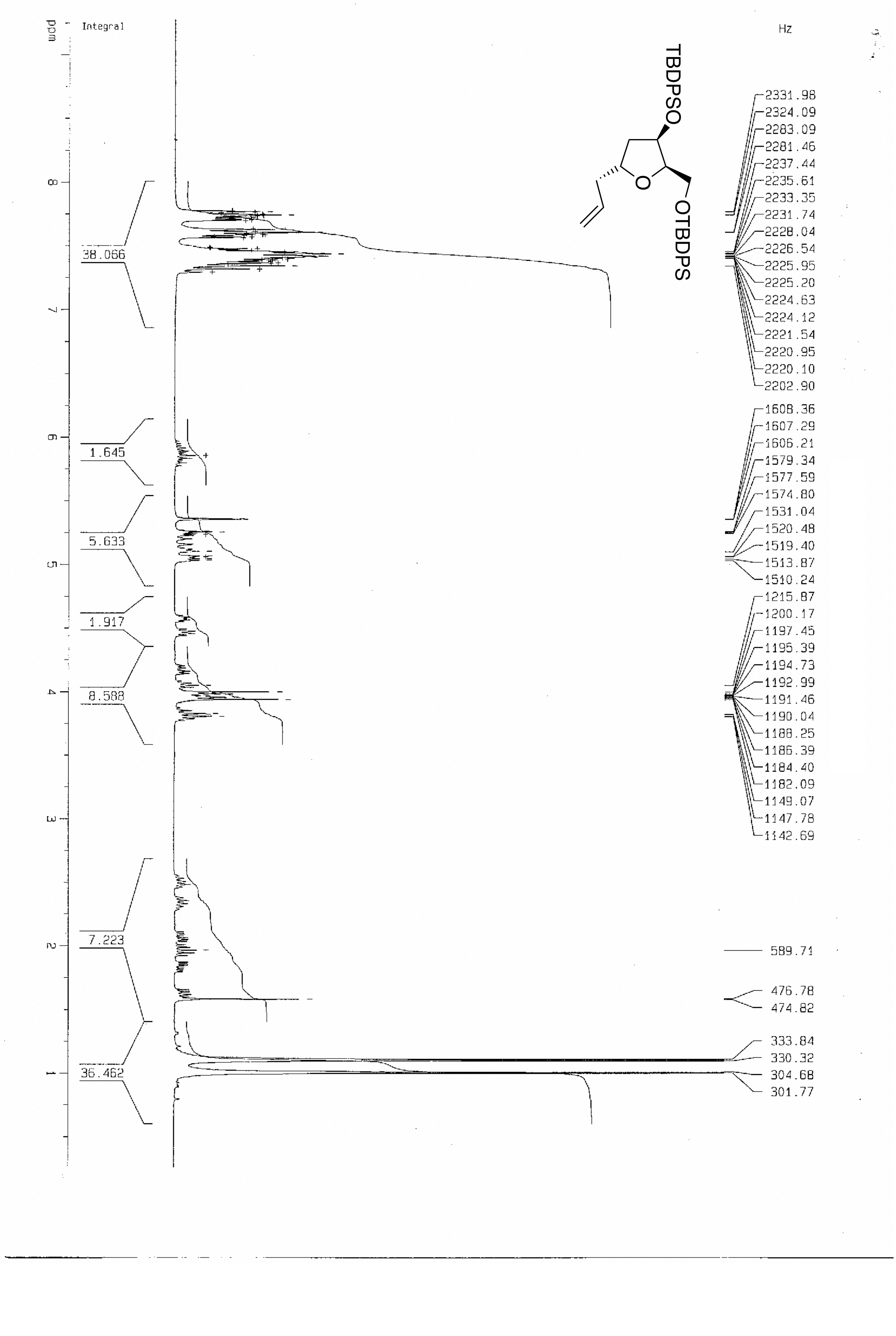

ppm
Integral
Hz
TBDPSO
OTBDPS
O
38.066
1.645
5.633
1.917
8.588
7.223
36.462
2331.98
2324.09
2283.09
2281.46
2237.44
2235.61
2233.35
2231.74
2228.04
2226.54
2225.95
2225.20
2224.63
2224.12
2221.54
2220.95
2220.10
2202.90
1608.36
1607.29
1606.21
1579.34
1577.59
1574.80
1531.04
1520.48
1519.40
1513.87
1510.24
1215.87
1200.17
1197.45
1195.39
1194.73
1192.99
1191.46
1190.04
1188.25
1186.39
1184.40
1182.09
1149.07
1147.78
1142.69
589.71
476.78
474.82
333.84
330.32
304.68
301.77

**$^{1}$H-$^{13}$C{$^{1}$H} NMR Spectra (Compound 33, 300 MHz, CDCl$_3$)**

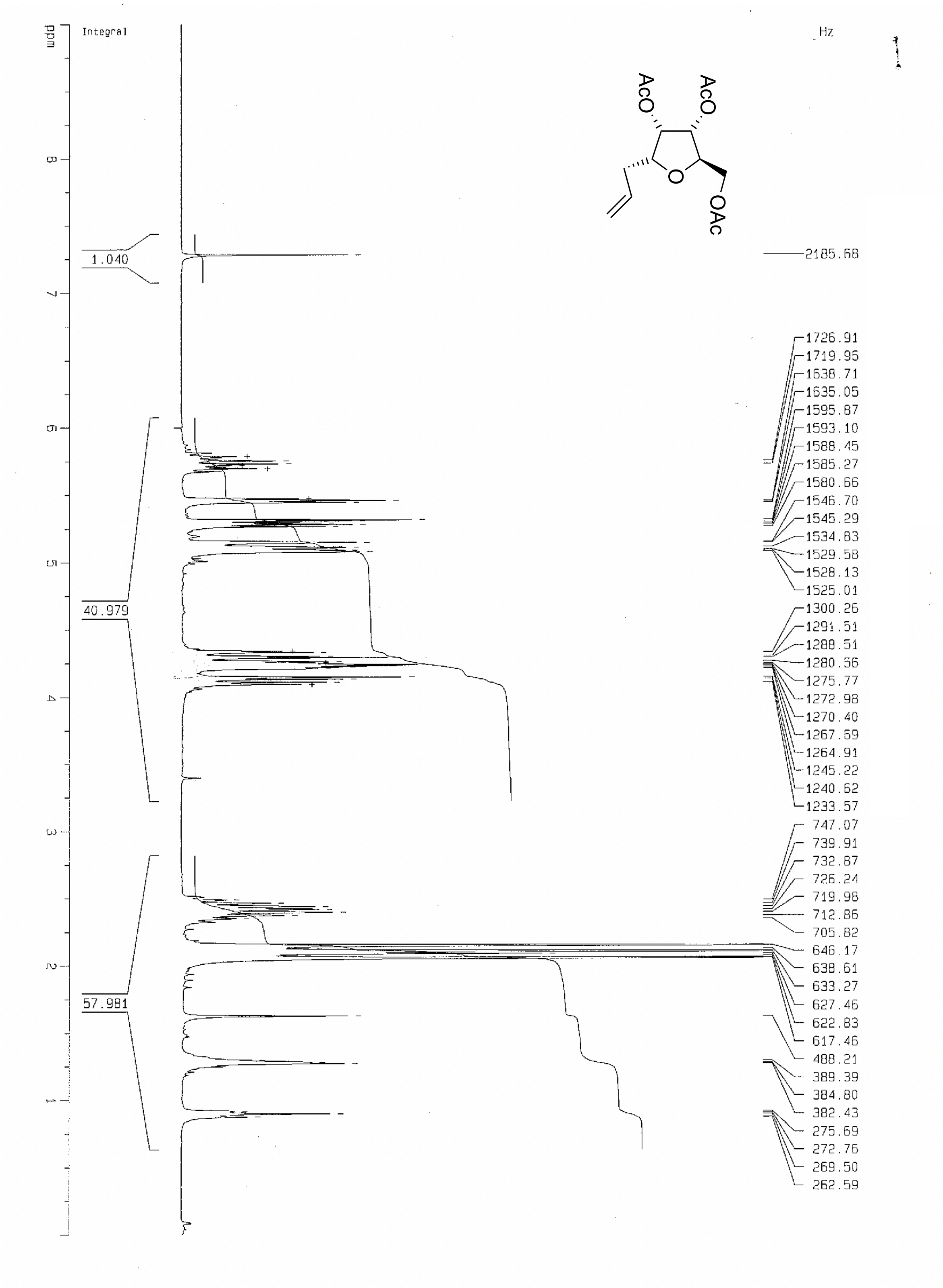

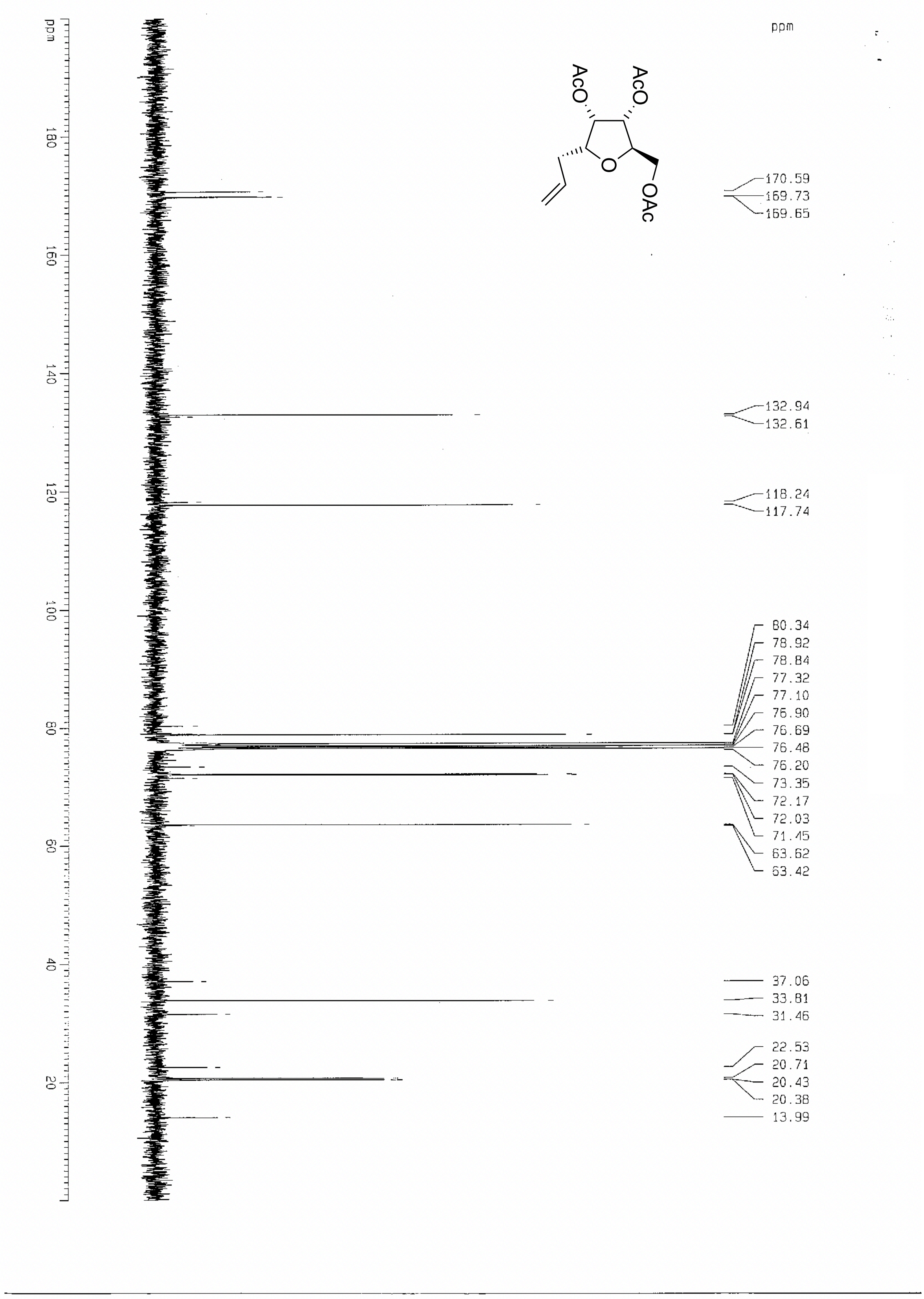
ppm
AcO
AcO
O
OAc
170.59
169.73
169.65
132.94
132.61
118.24
117.74
80.34
78.92
78.84
77.32
77.10
76.90
76.69
76.48
76.20
73.35
72.17
72.03
71.45
63.62
63.42
37.06
33.81
31.46
22.53
20.71
20.43
20.38
13.99
180
160
140
120
100
80
60
40
20

**$^{1}H$-$^{13}C\{^{1}H\}$ NMR Spectra (Compound 34, 300 MHz, $CDCl_3$)**

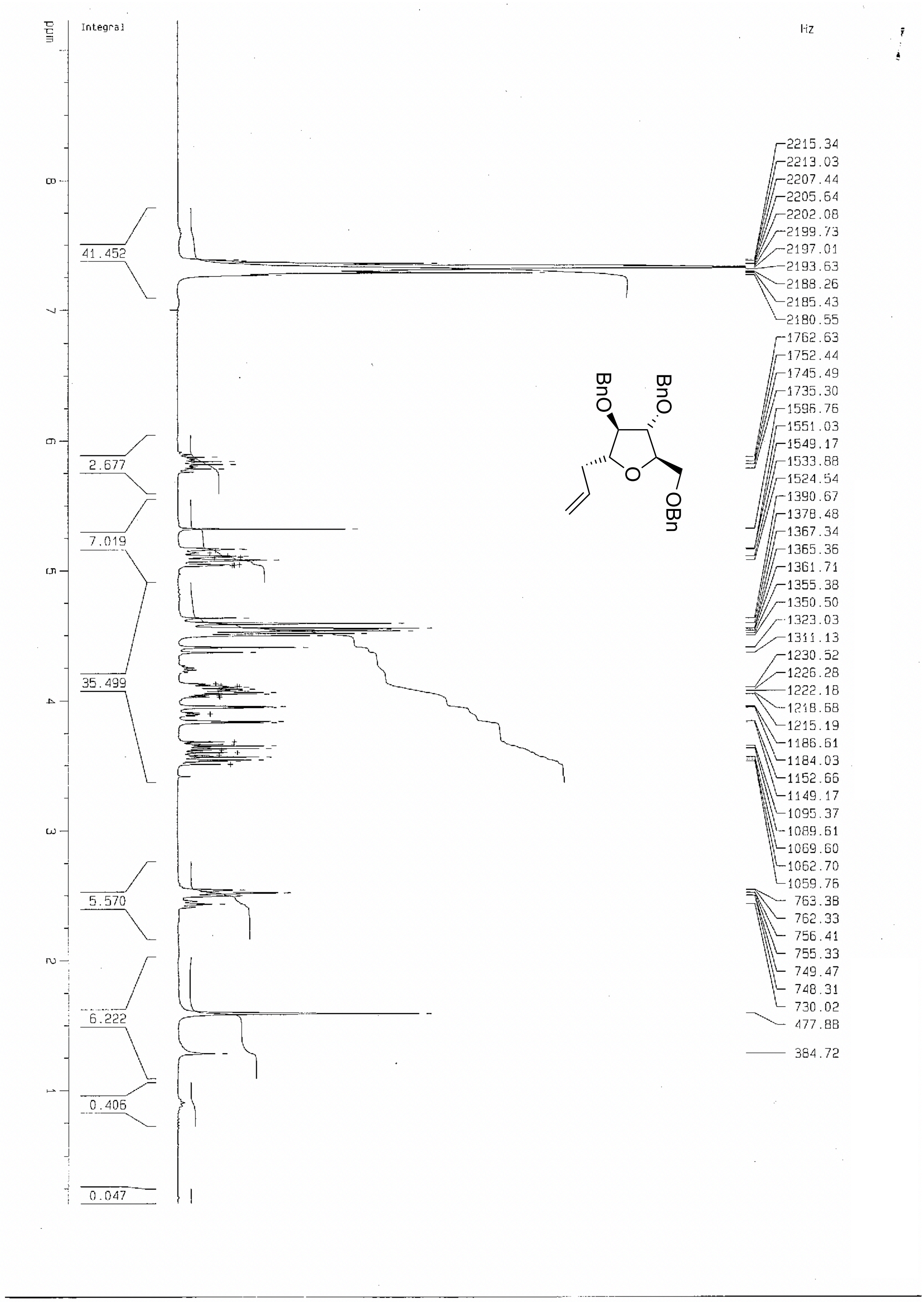

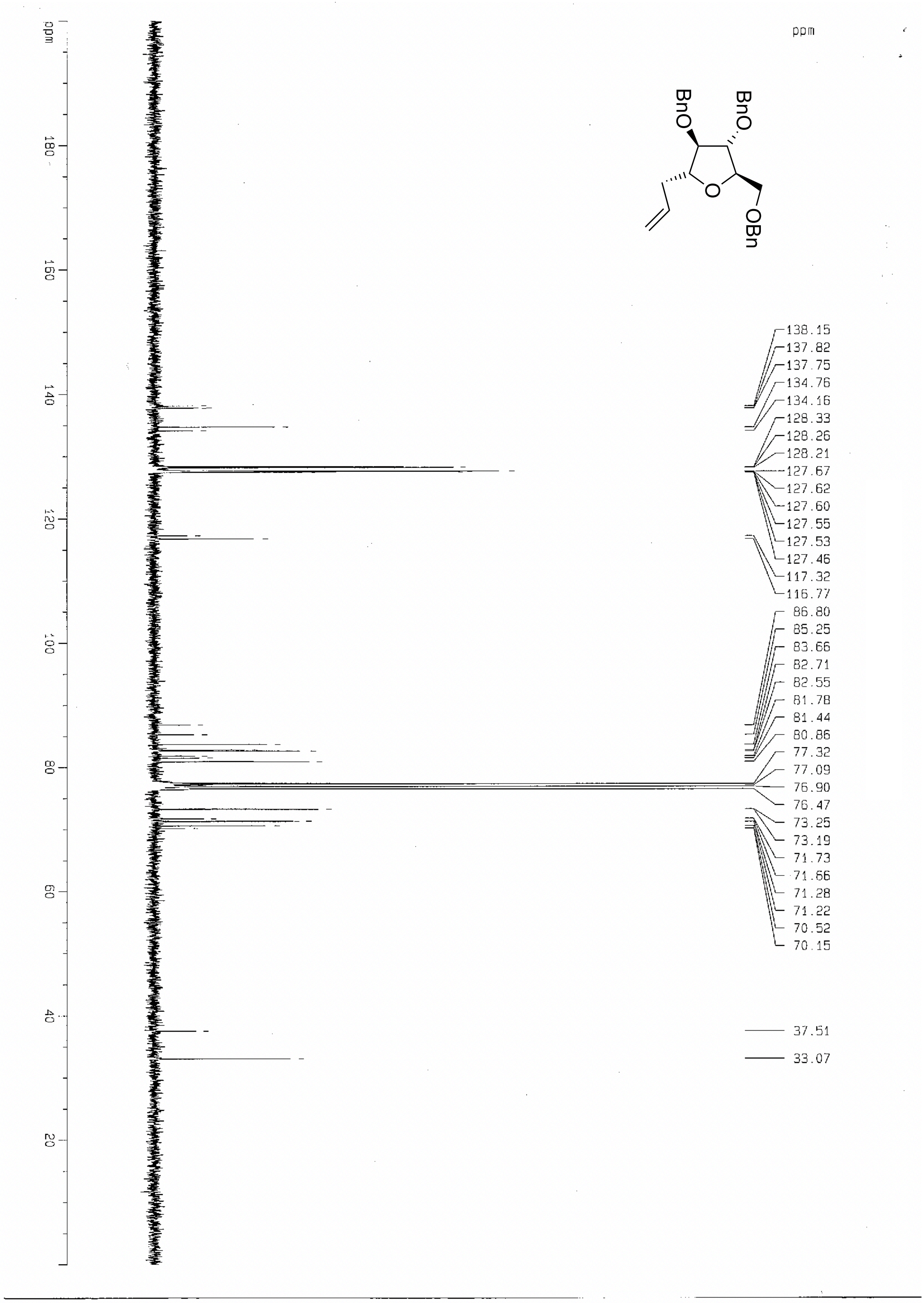
ppm
BnO
BnO
OBn
138.15
137.82
137.75
134.76
134.16
128.33
128.26
128.21
127.67
127.62
127.60
127.55
127.53
127.46
117.32
116.77
86.80
85.25
83.66
82.71
82.55
81.78
81.44
80.86
77.32
77.09
76.90
76.47
73.25
73.19
71.73
71.66
71.28
71.22
70.52
70.15
37.51
33.07
180
160
140
120
100
80
60
40
20

**$^{1}$H-$^{13}$C{$^{1}$H} NMR Spectra (Compound 13, 400 MHz, $(CD_3)_2CO$)**

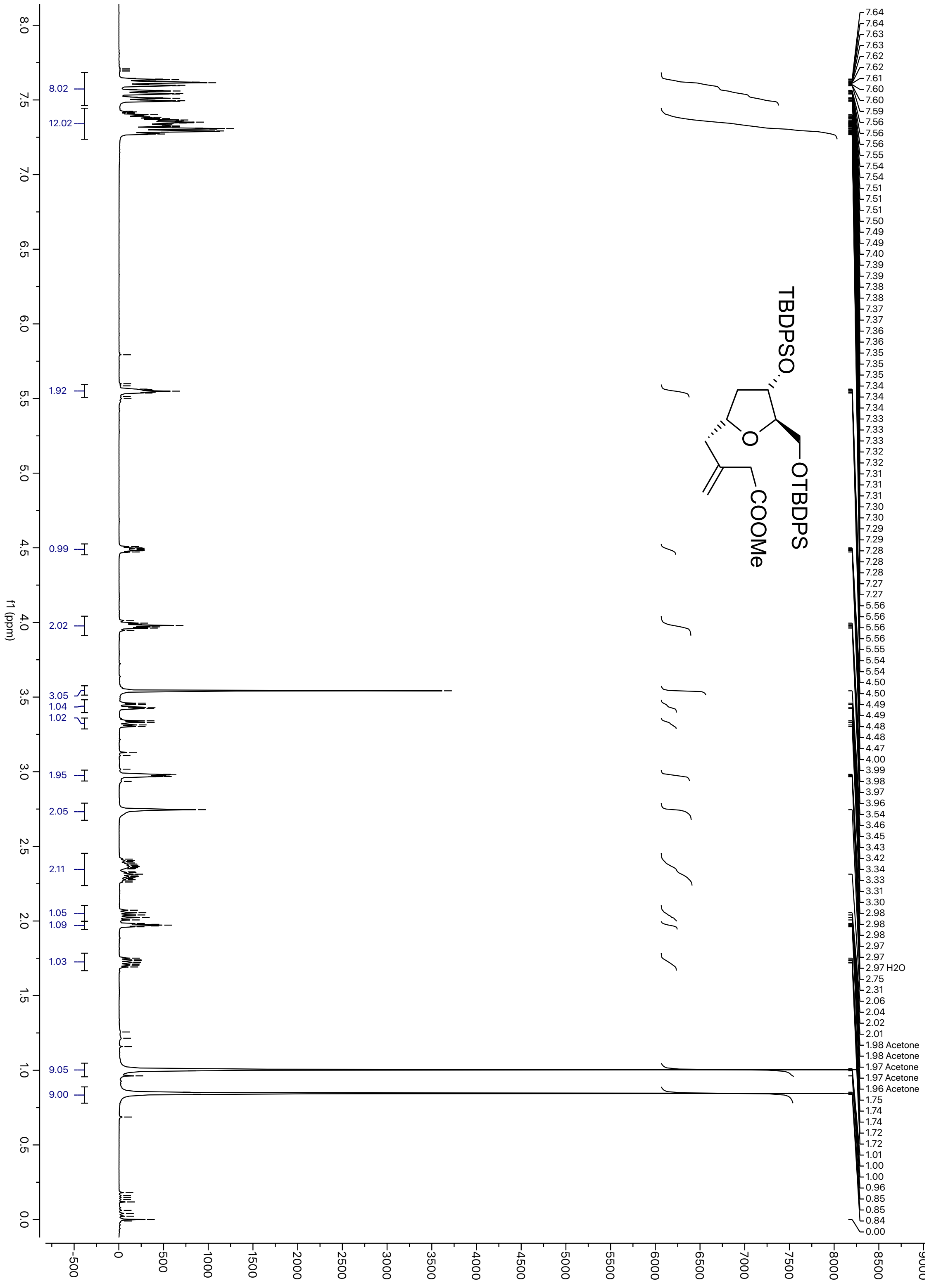

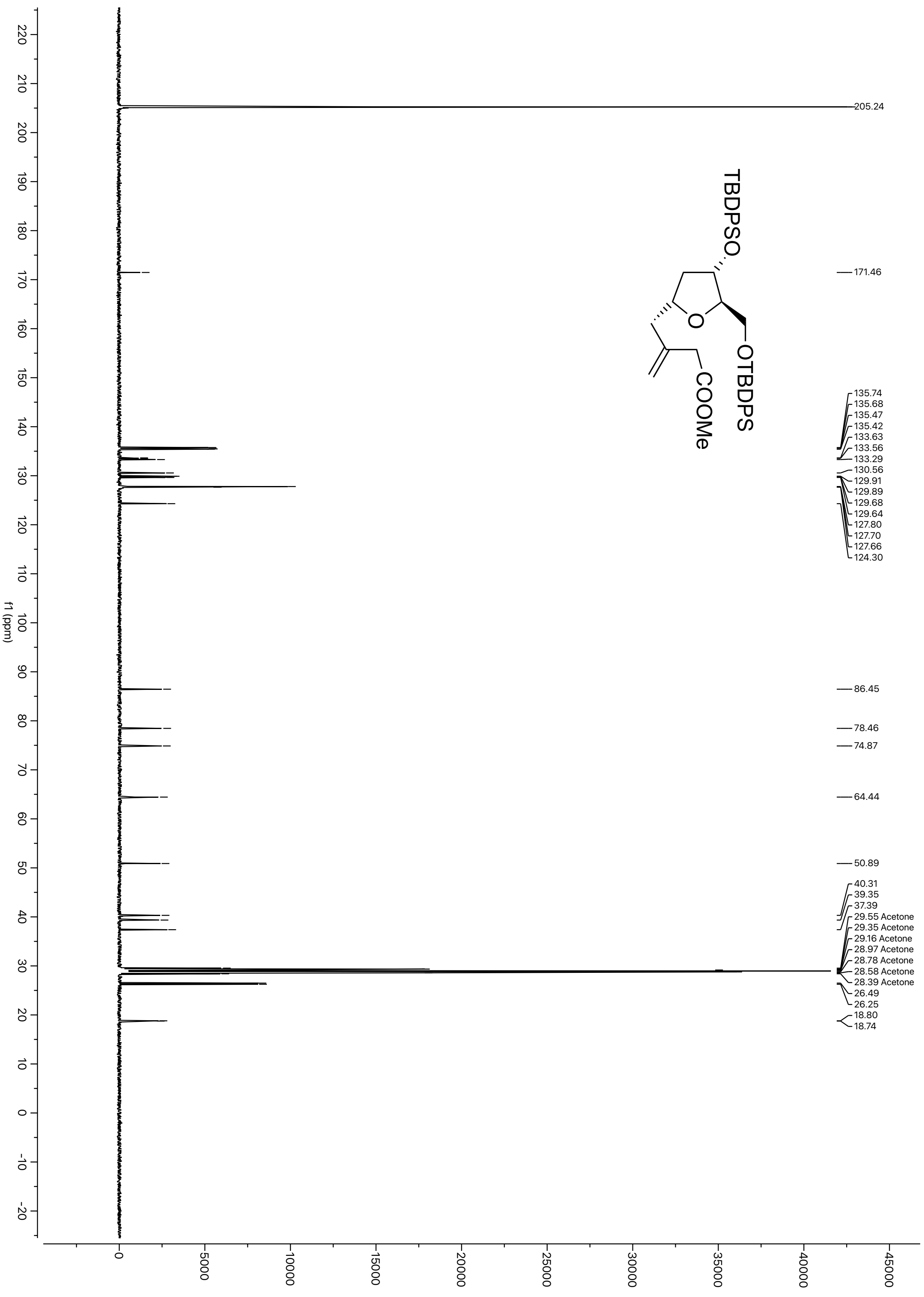
TBDPSO
OTBDPS
O
COOMe
205.24
171.46
135.74
135.68
135.47
135.42
133.63
133.56
133.29
130.56
129.91
129.89
129.68
129.64
127.80
127.70
127.66
124.30
86.45
78.46
74.87
64.44
50.89
40.31
39.35
37.39
29.55 Acetone
29.35 Acetone
29.16 Acetone
28.97 Acetone
28.78 Acetone
28.58 Acetone
28.39 Acetone
26.49
26.25
18.80
18.74
f1 (ppm)

**$^1$H-$^{13}$C{$^1$H} NMR Spectra (Compound 14, 400 MHz, $CDCl_3$)**

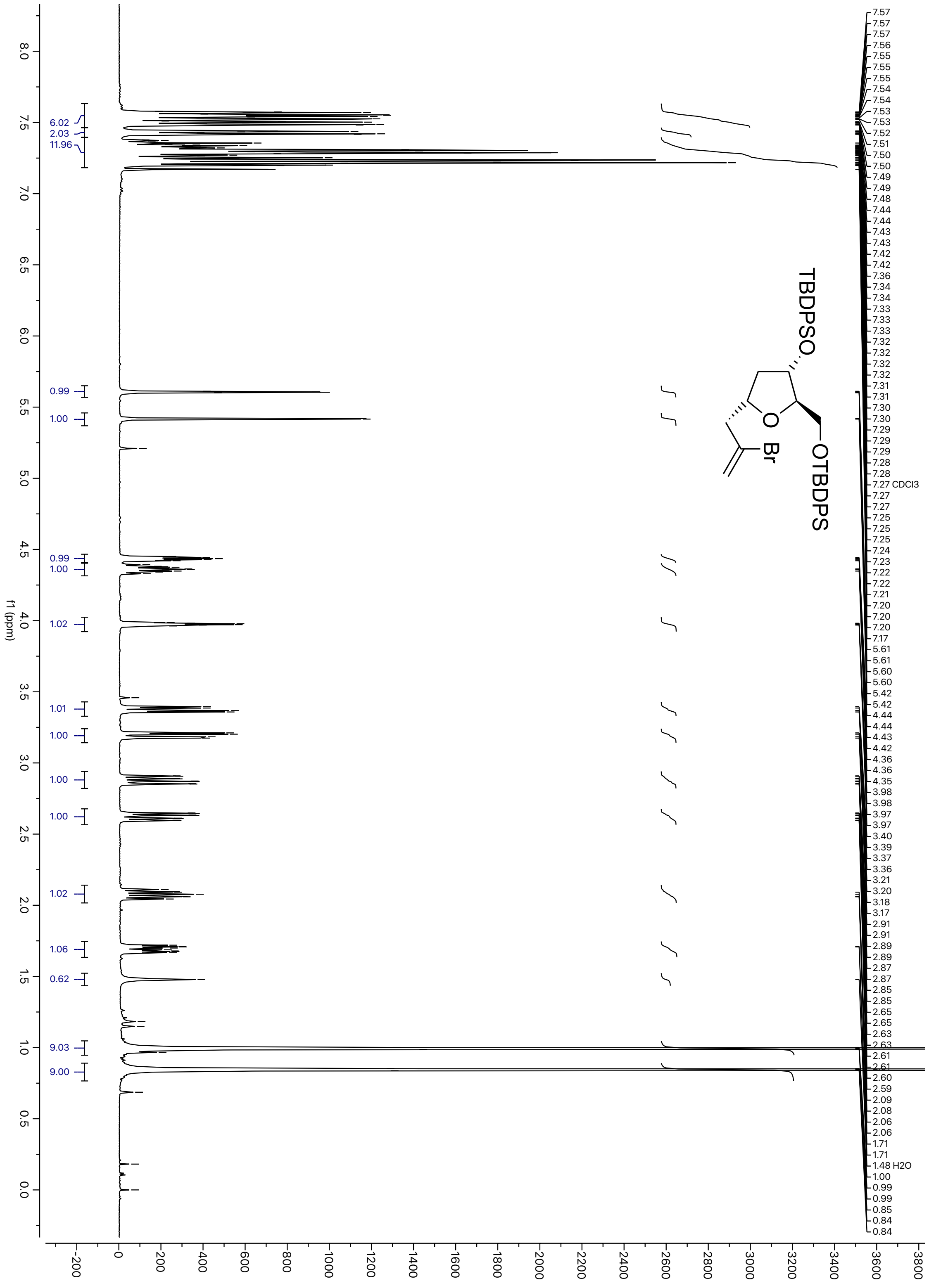

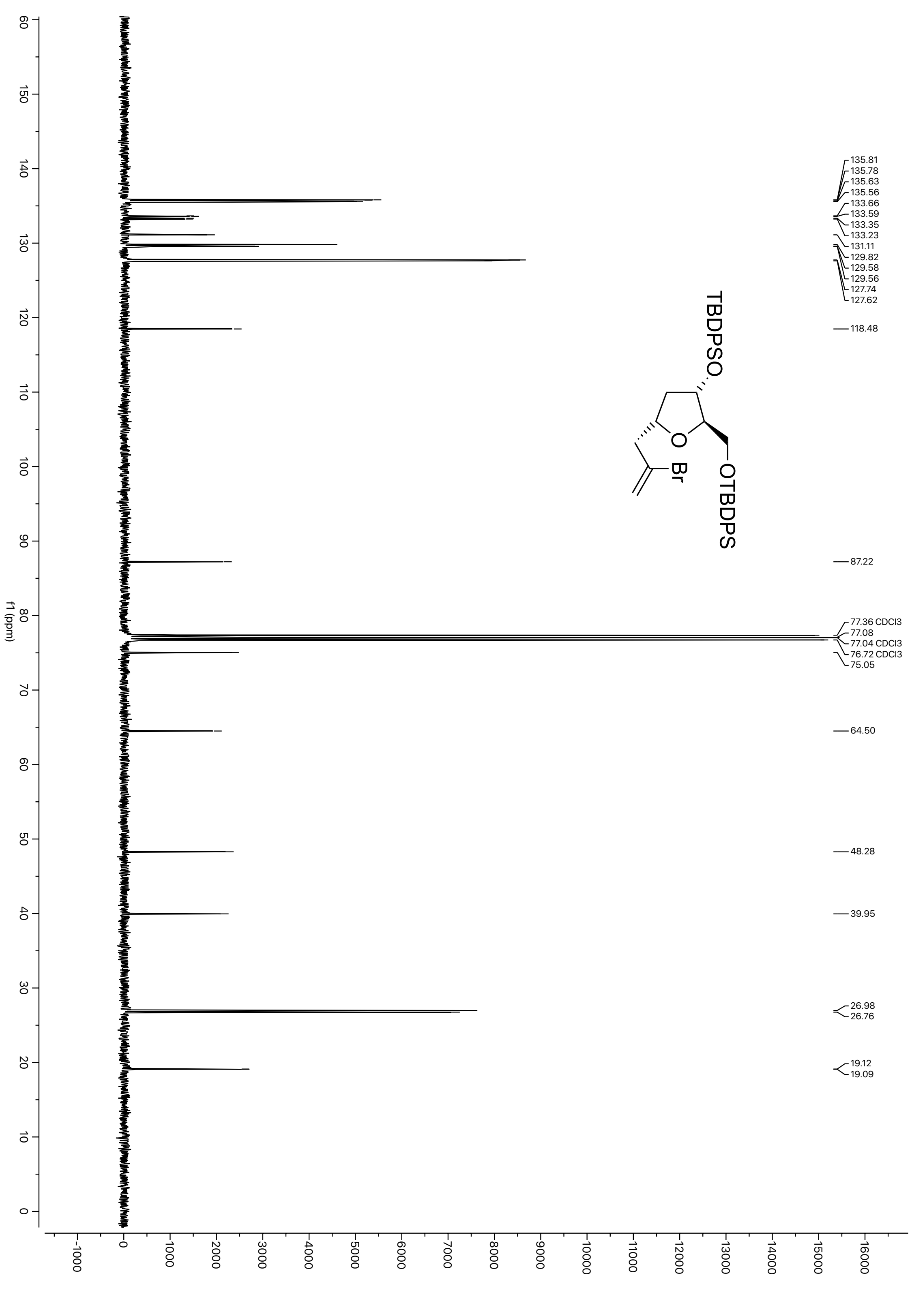
135.81
135.78
135.63
135.56
133.66
133.59
133.35
133.23
131.11
129.82
129.58
129.56
127.74
127.62
118.48
87.22
77.36 CDCl3
77.08
77.04 CDCl3
76.72 CDCl3
75.05
64.50
48.28
39.95
26.98
26.76
19.12
19.09
TBDPSO
OTBDPS
O
Br
f1 (ppm)

## $^{1}$H-$^{13}$C{$^{1}$H} NMR Spectra (Compound 15, 300 MHz, $CDCl_3$)

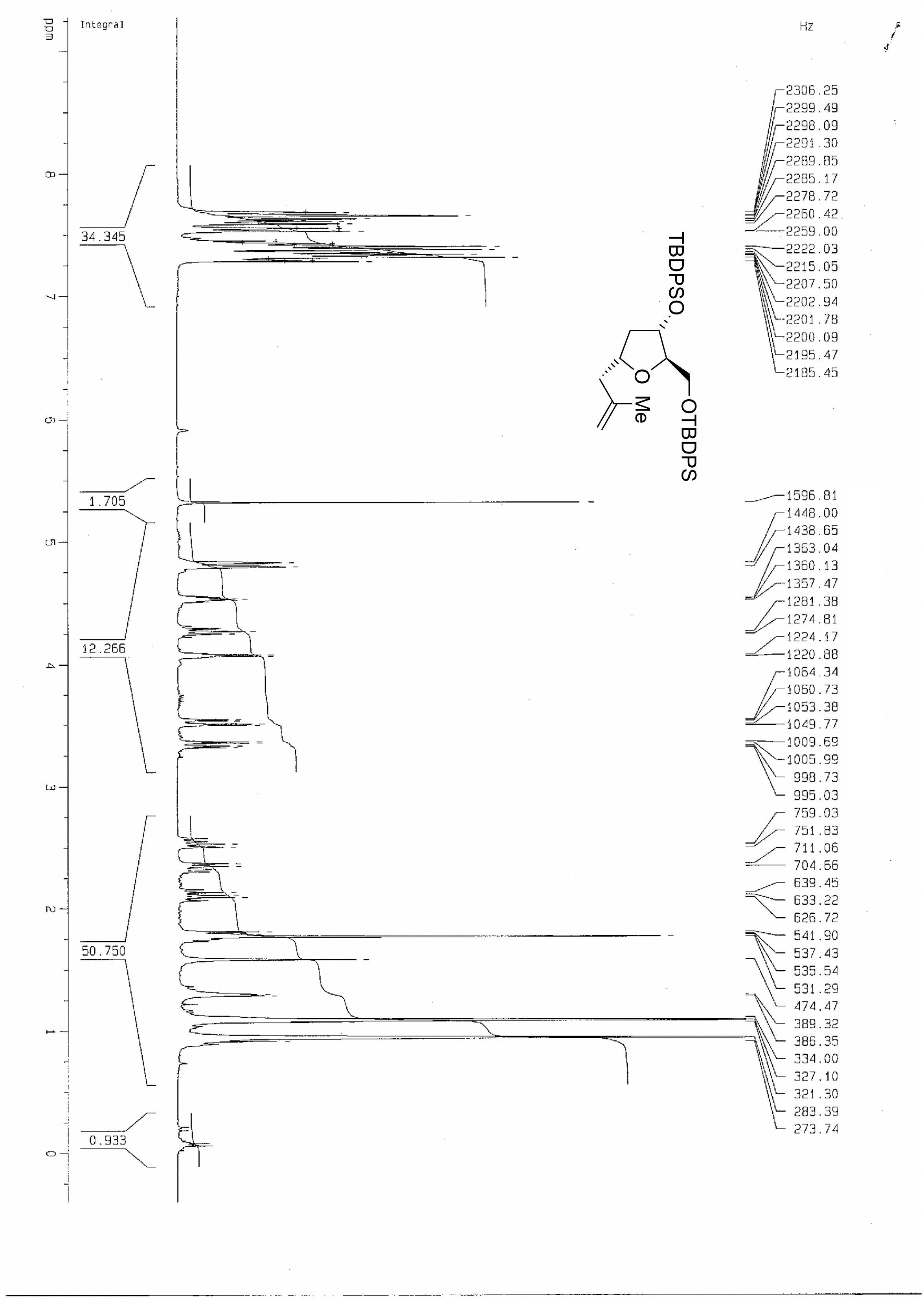

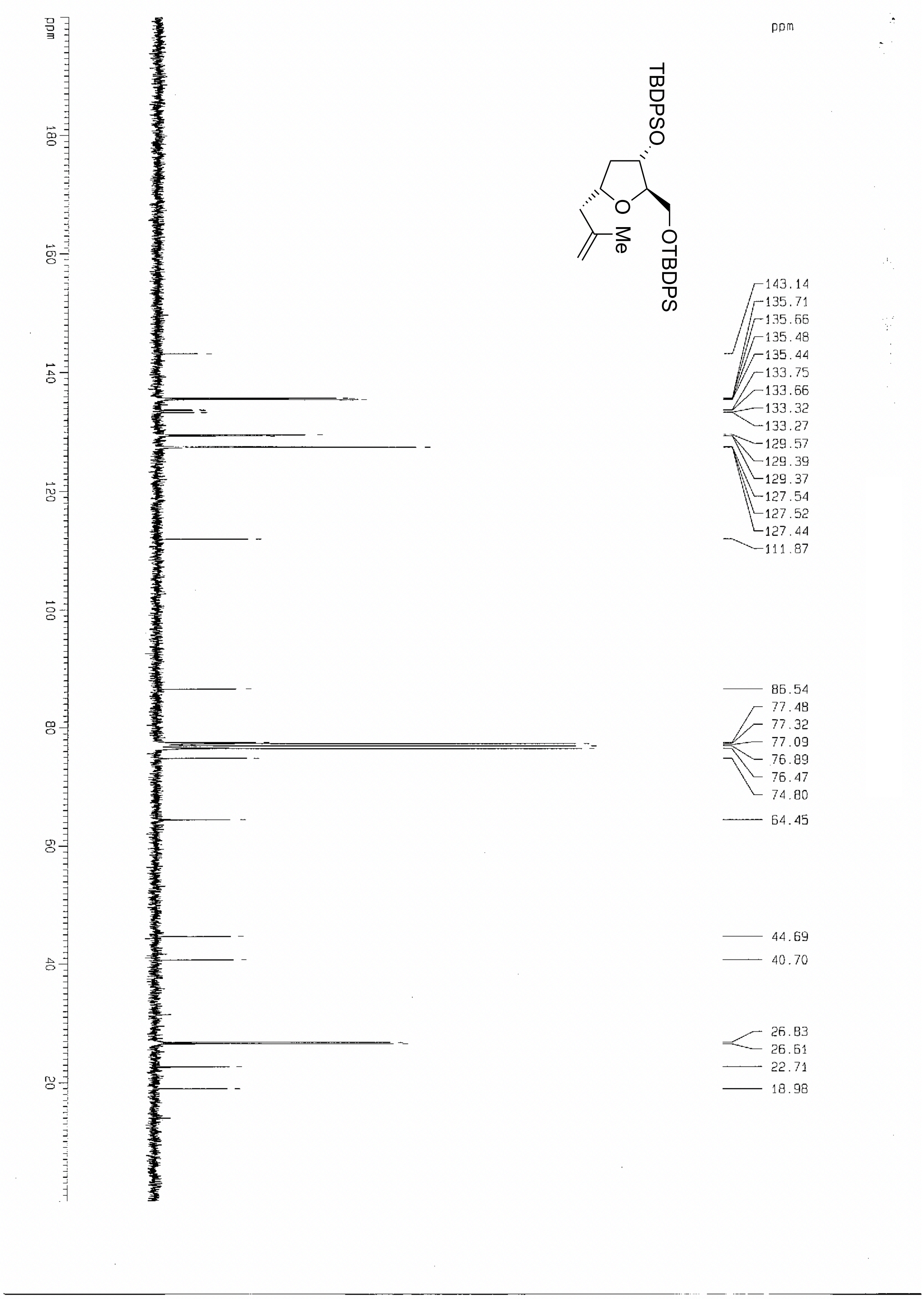
ppm
TBDPSO
OTBDPS
O
Me
143.14
135.71
135.66
135.48
135.44
133.75
133.66
133.32
133.27
129.57
129.39
129.37
127.54
127.52
127.44
111.87
86.54
77.48
77.32
77.09
76.89
76.47
74.80
64.45
44.69
40.70
26.83
26.61
22.71
18.98
180
160
140
120
100
80
60
40
20

# $^{1}H$-$^{13}C\{^{1}H\}$ NMR Spectra (Compound 16, 400 MHz, $CDCl_3$)

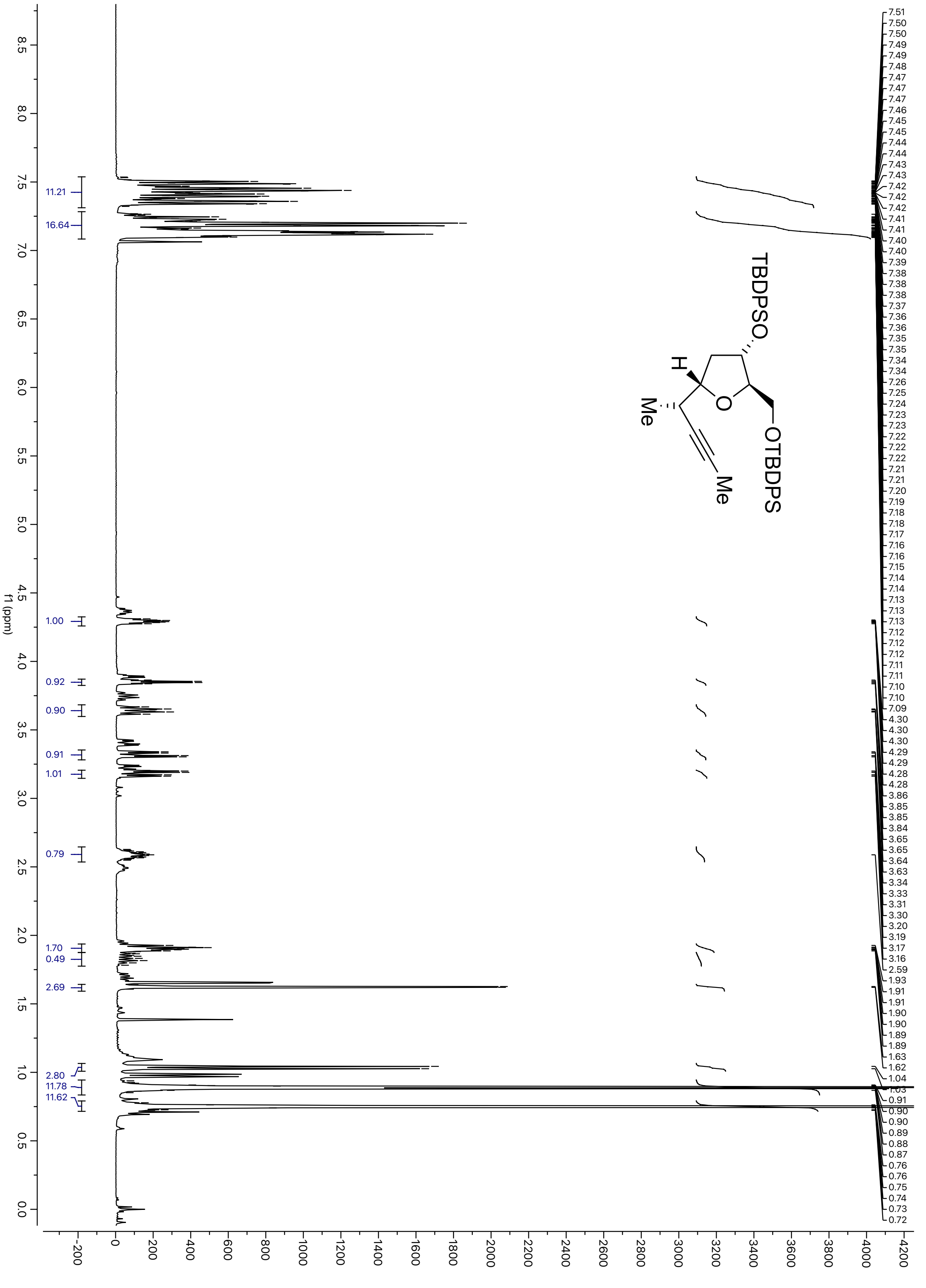

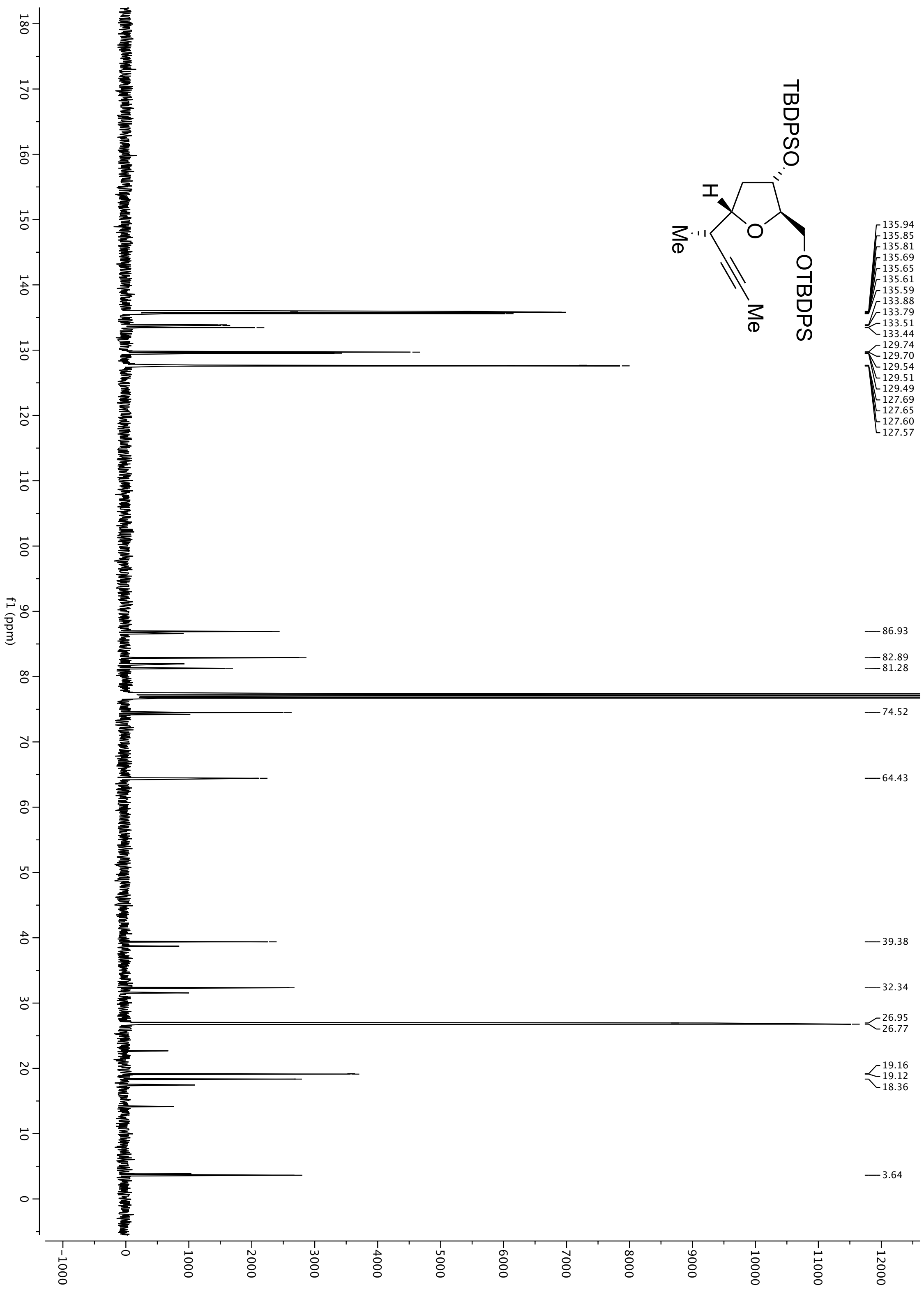

TBDPSO
H
O
OTBDPS
Me
Me
135.94
135.85
135.81
135.69
135.65
135.61
135.59
133.88
133.79
133.51
133.44
129.74
129.70
129.54
129.51
129.49
127.69
127.65
127.60
127.57
86.93
82.89
81.28
74.52
64.43
39.38
32.34
26.95
26.77
19.16
19.12
18.36
3.64
f1 (ppm)

## $^{1}$H-$^{13}$C{$^{1}$H} NMR Spectra (Compound 17, 300 MHz, $CDCl_3$)

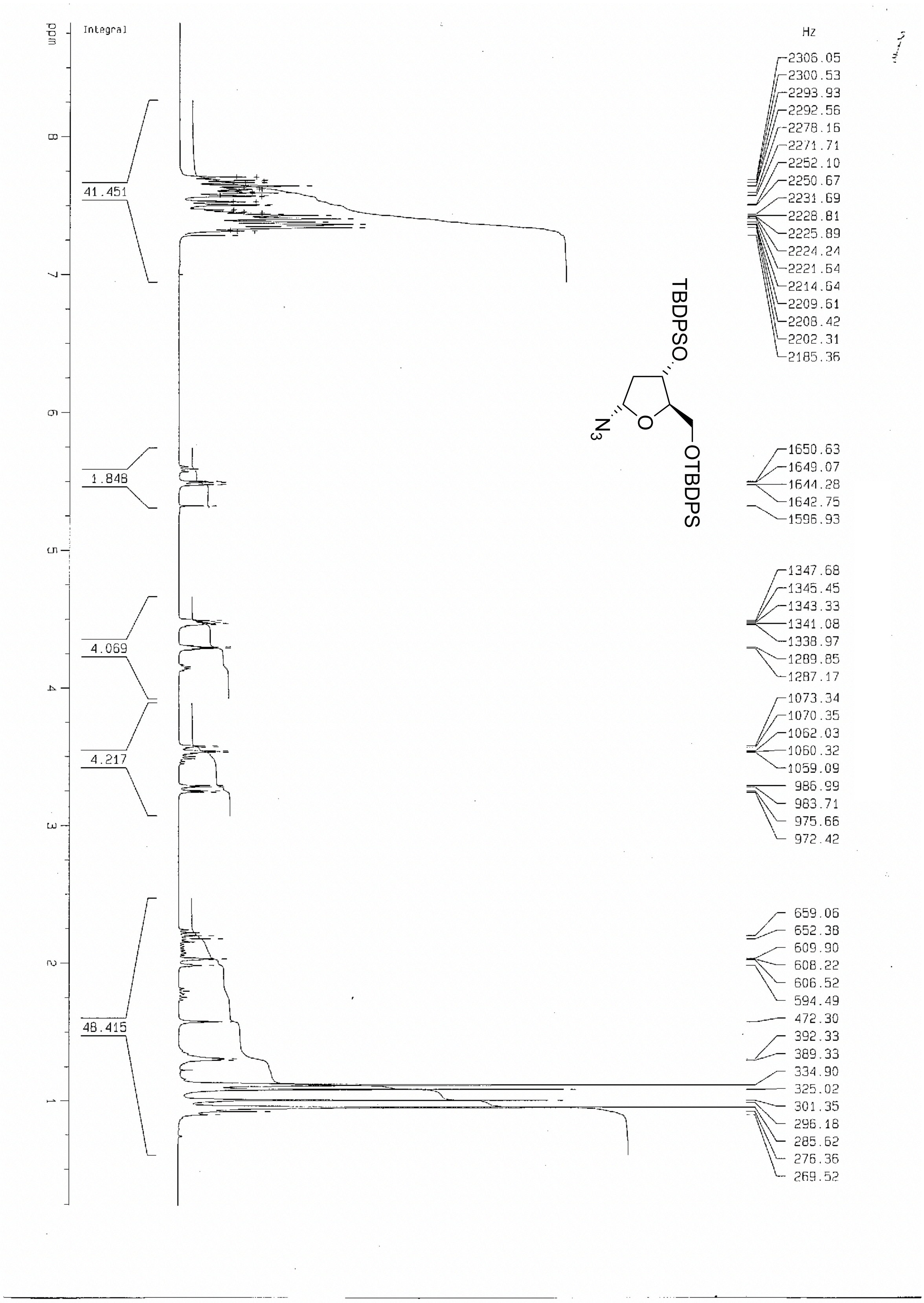

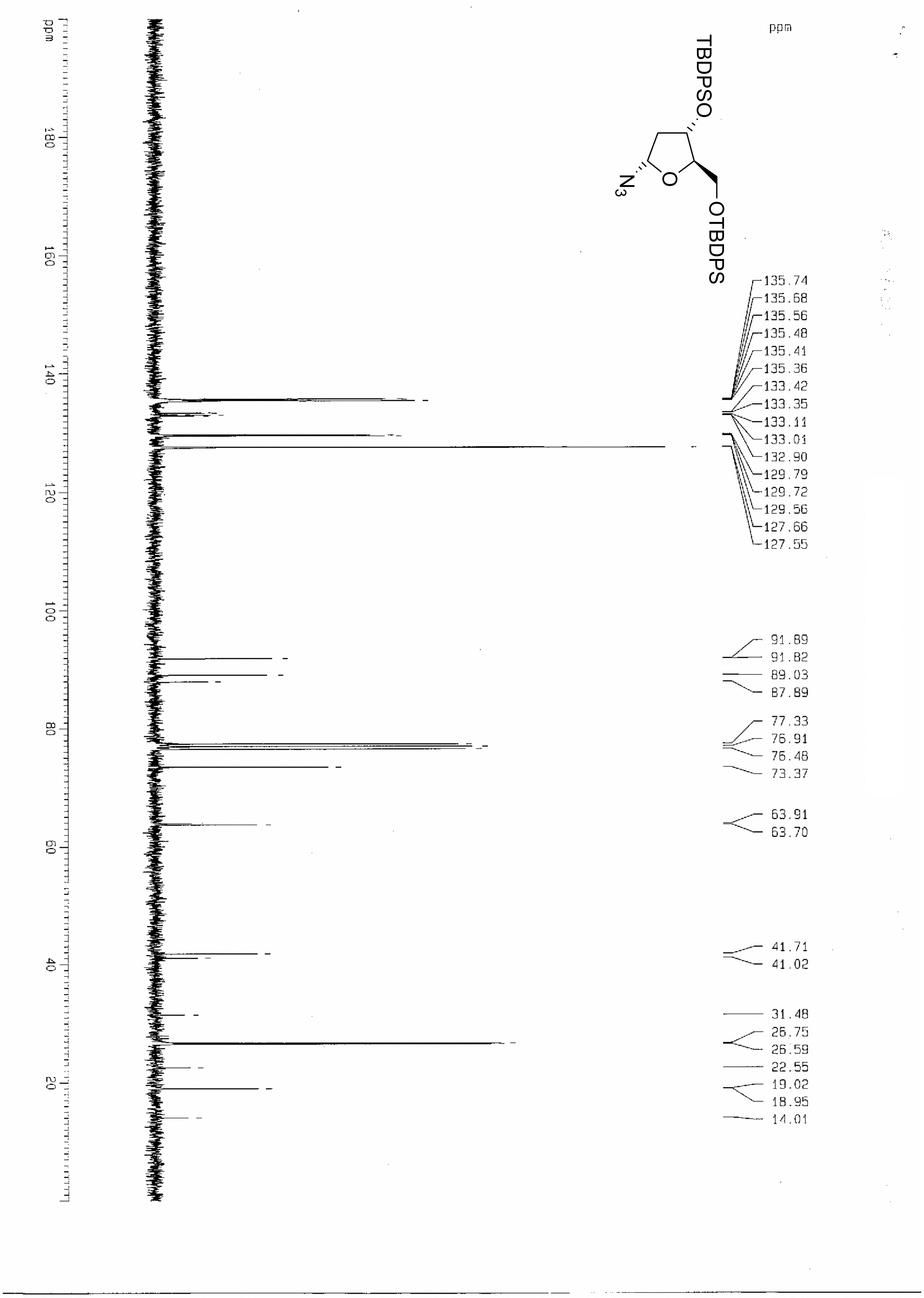
TBDPSO
OTBDPS
O
N3
ppm
135.74
135.68
135.56
135.48
135.41
135.36
133.42
133.35
133.11
133.01
132.90
129.79
129.72
129.56
127.66
127.55
91.89
91.82
89.03
87.89
77.33
76.91
76.48
73.37
63.91
63.70
41.71
41.02
31.48
26.75
26.59
22.55
19.02
18.95
14.01
ppm
180
160
140
120
100
80
60
40
20

**$^{1}$H-$^{13}$C{$^{1}$H} NMR Spectra (Compound 18, 300 MHz, $CDCl_3$)**

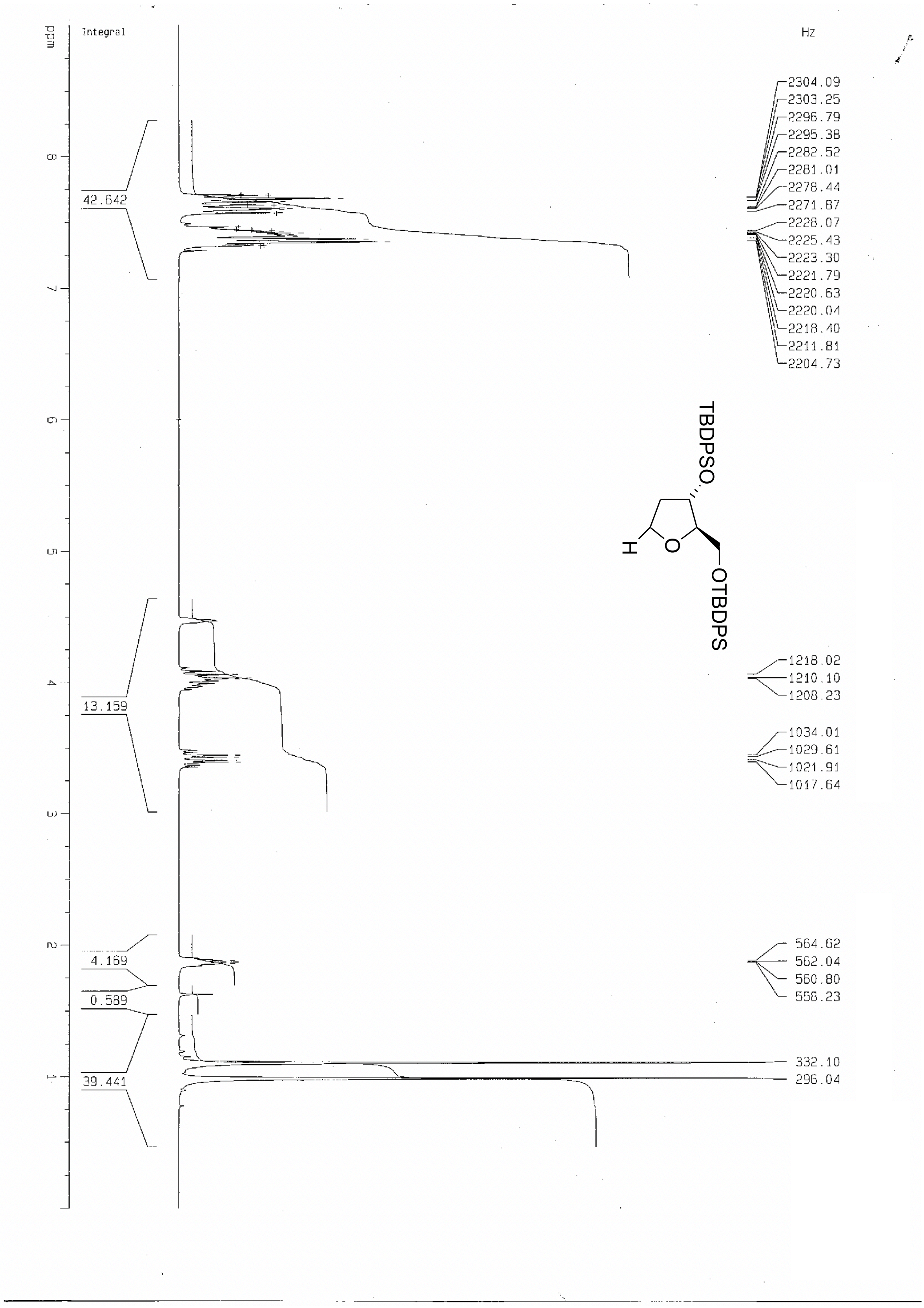

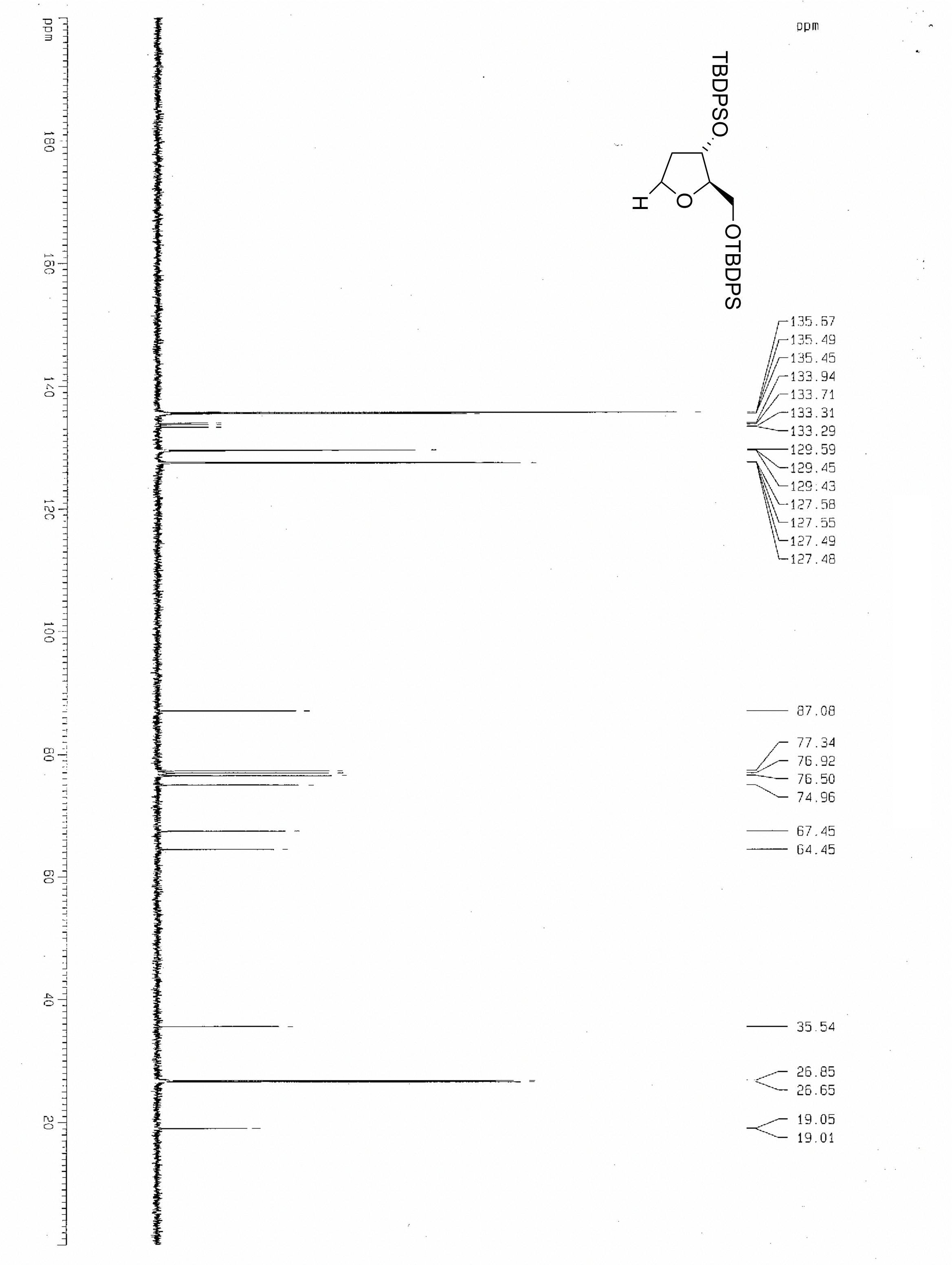
TBDPSO
OTBDPS
O
H
ppm
135.67
135.49
135.45
133.94
133.71
133.31
133.29
129.59
129.45
129.43
127.58
127.55
127.49
127.48
87.08
77.34
76.92
76.50
74.96
67.45
64.45
35.54
26.85
26.65
19.05
19.01
180
160
140
120
100
80
60
40
20

## $^{1}H$-$^{13}C\{^{1}H\}$ NMR Spectra (Compound 19, 300 MHz, $CDCl_3$)

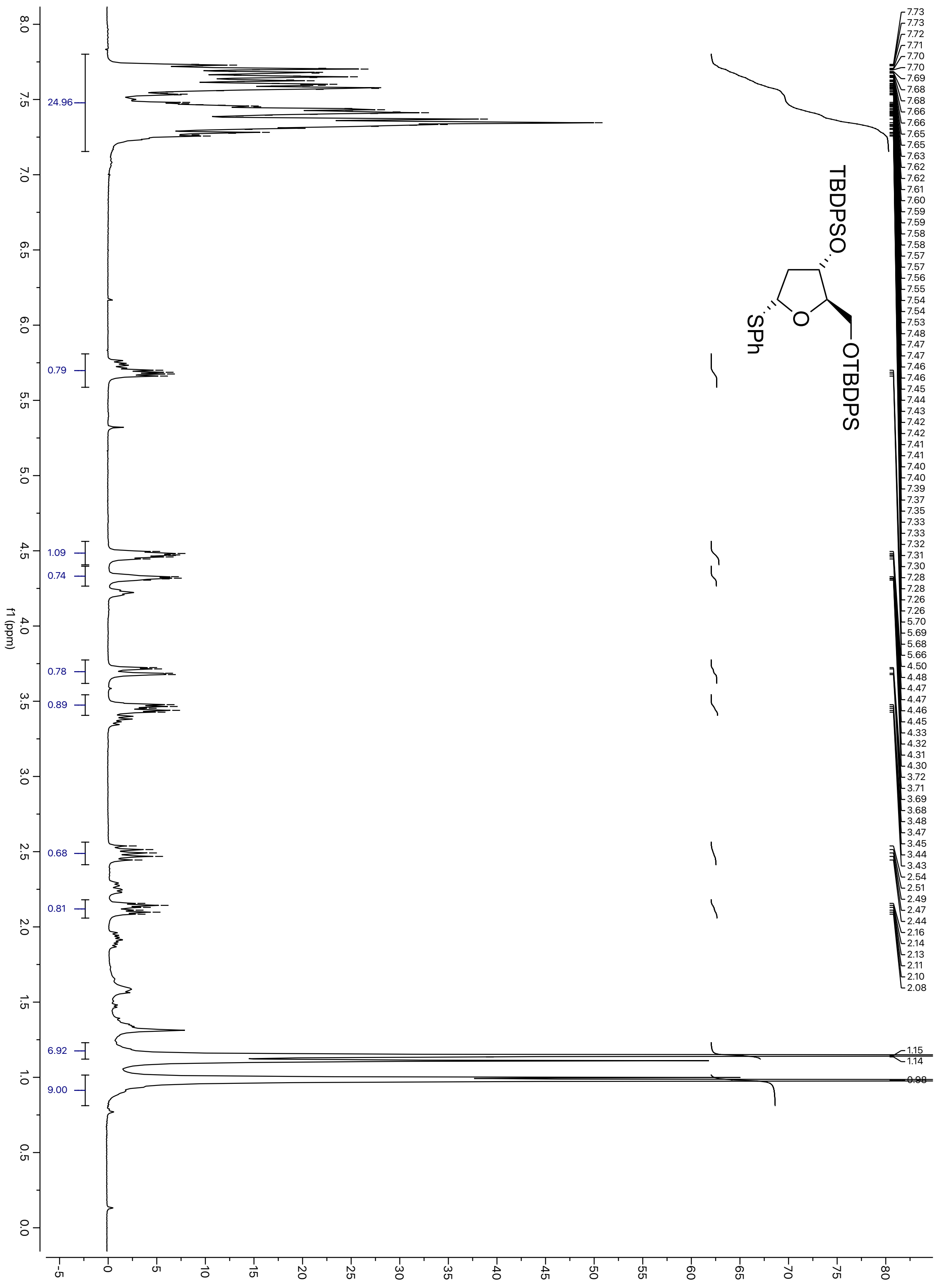

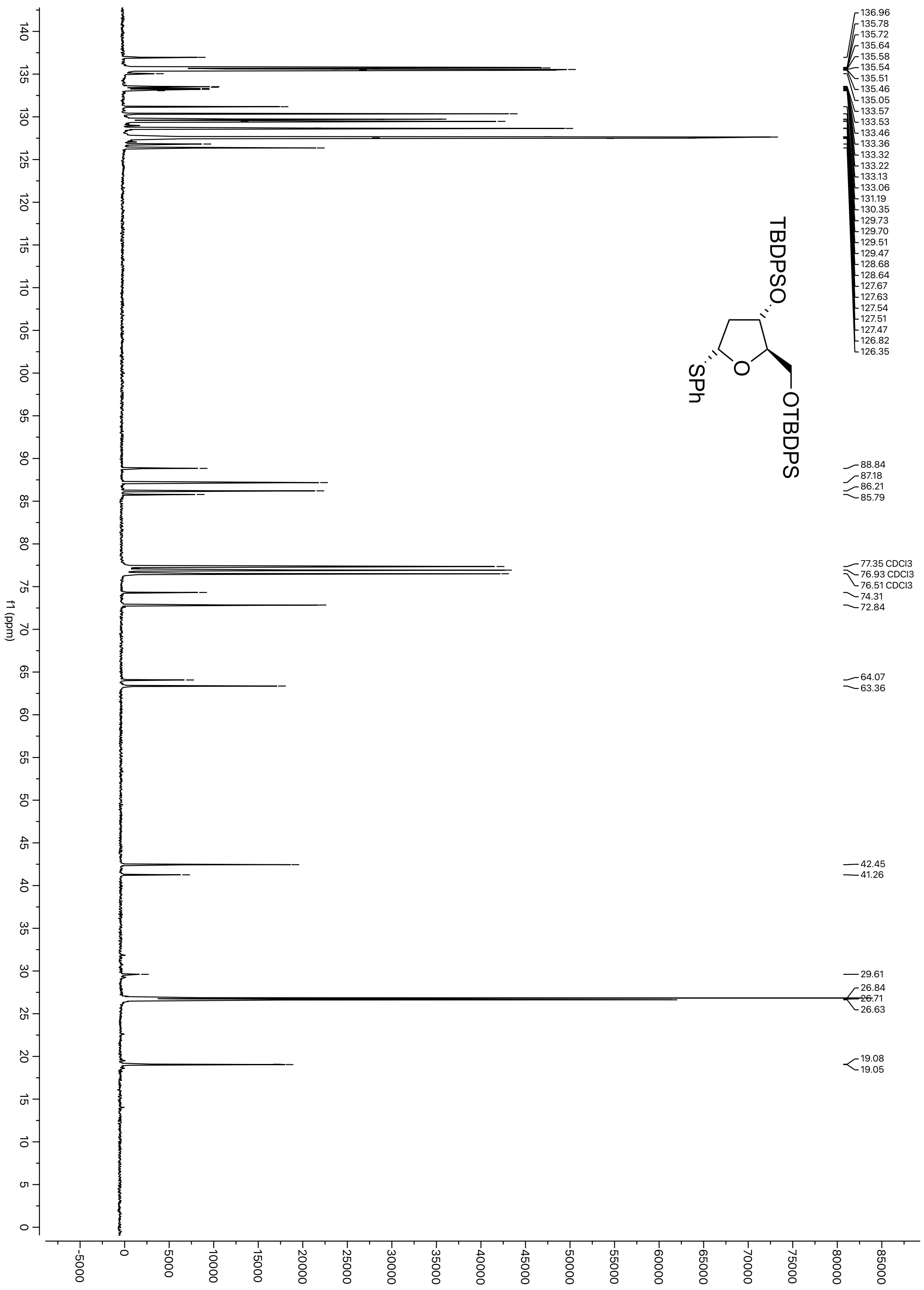

TBDPSO
O
SPh
OTBDPS
136.96
135.78
135.72
135.64
135.58
135.54
135.51
135.46
135.05
133.57
133.53
133.46
133.36
133.32
133.22
133.13
133.06
131.19
130.35
129.73
129.70
129.51
129.47
128.68
128.64
127.67
127.63
127.54
127.51
127.47
126.82
126.35
88.84
87.18
86.21
85.79
77.35 CDCl3
76.93 CDCl3
76.51 CDCl3
74.31
72.84
64.07
63.36
42.45
41.26
29.61
26.84
26.71
26.63
19.08
19.05
f1 (ppm)

## $^{1}H$-$^{13}C\{^{1}H\}$ NMR Spectra (Compound 20, 400 MHz, $CDCl_3$)

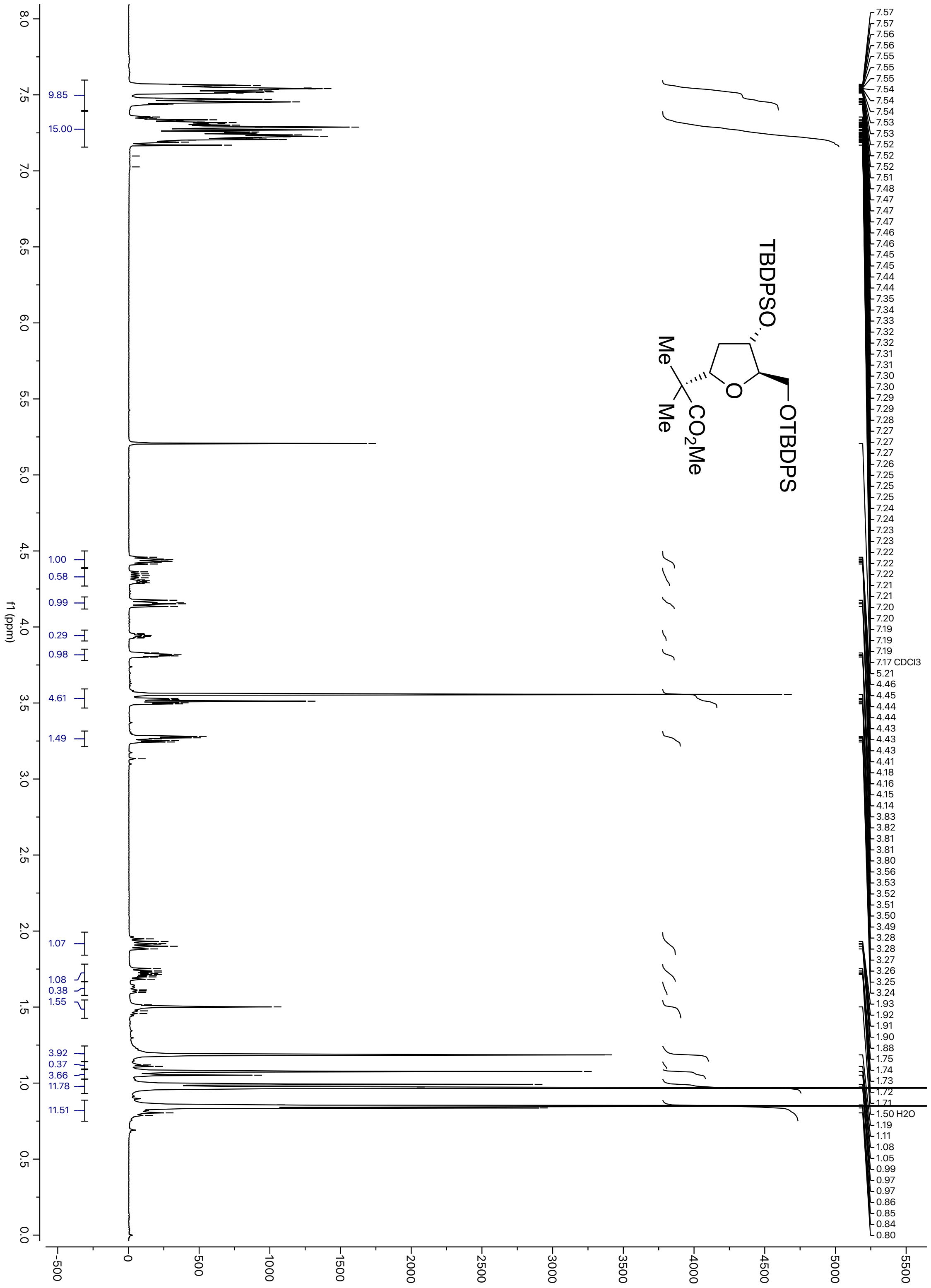

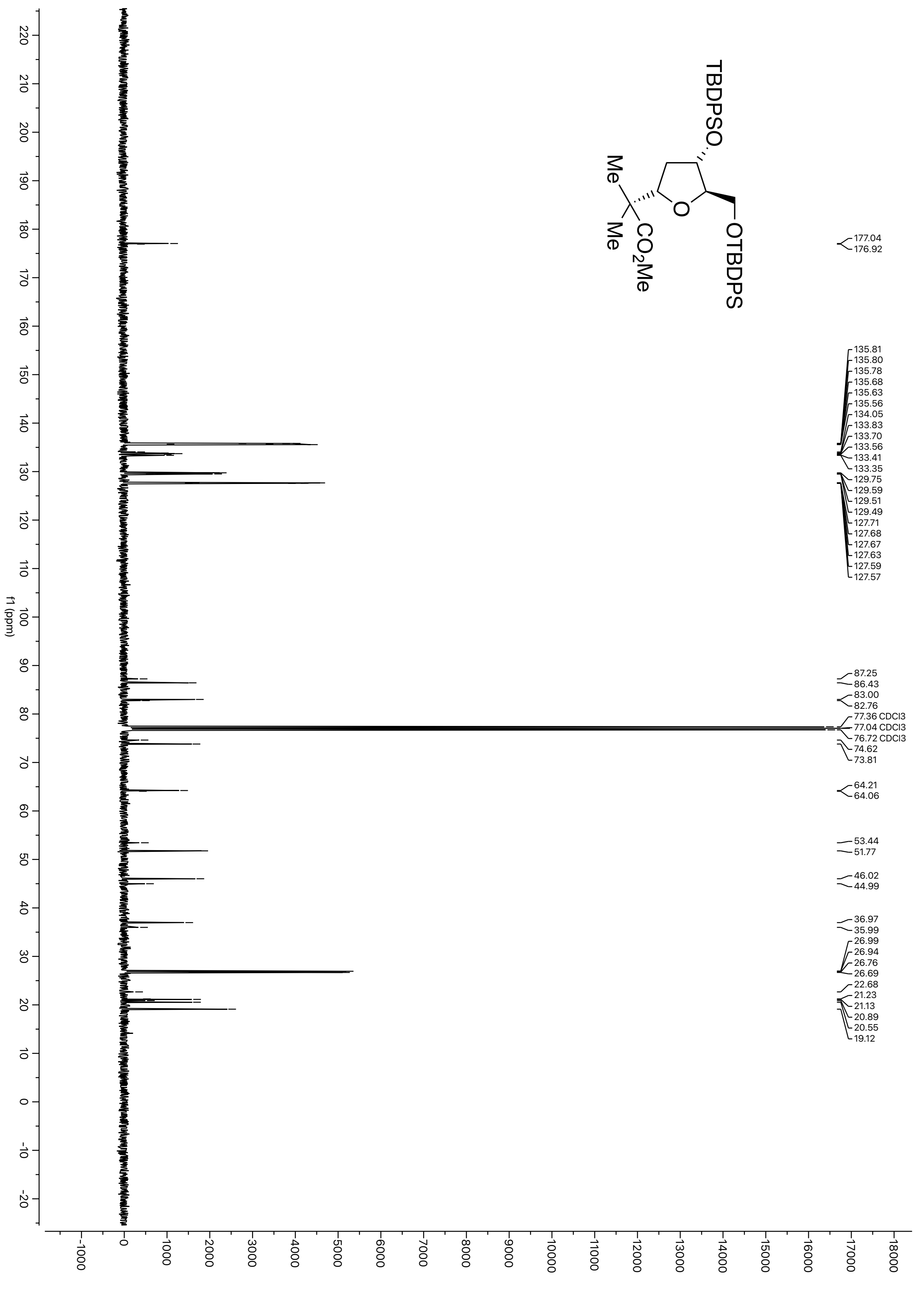
TBDPSO
OTBDPS
O
Me
Me
CO2Me
177.04
176.92
135.81
135.80
135.78
135.68
135.63
135.56
134.05
133.83
133.70
133.56
133.41
133.35
129.75
129.59
129.51
129.49
127.71
127.68
127.67
127.63
127.59
127.57
87.25
86.43
83.00
82.76
77.36 CDCl3
77.04 CDCl3
76.72 CDCl3
74.62
73.81
64.21
64.06
53.44
51.77
46.02
44.99
36.97
35.99
26.99
26.94
26.76
26.69
22.68
21.23
21.13
20.89
20.55
19.12
f1 (ppm)

**$^{1}$H-$^{13}$C{$^{1}$H} NMR Spectra (Compound 21, 400 MHz, $CDCl_3$)**

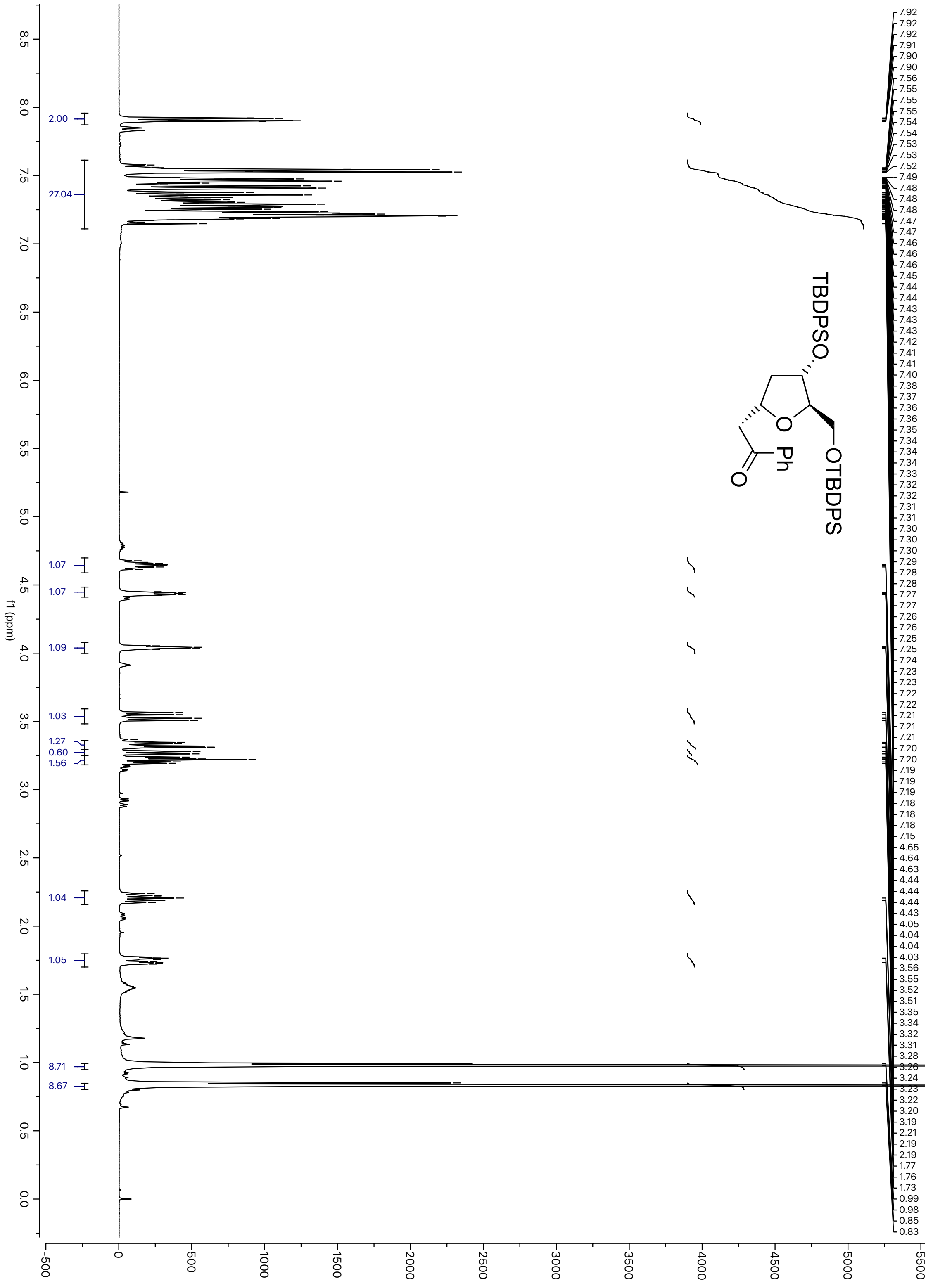

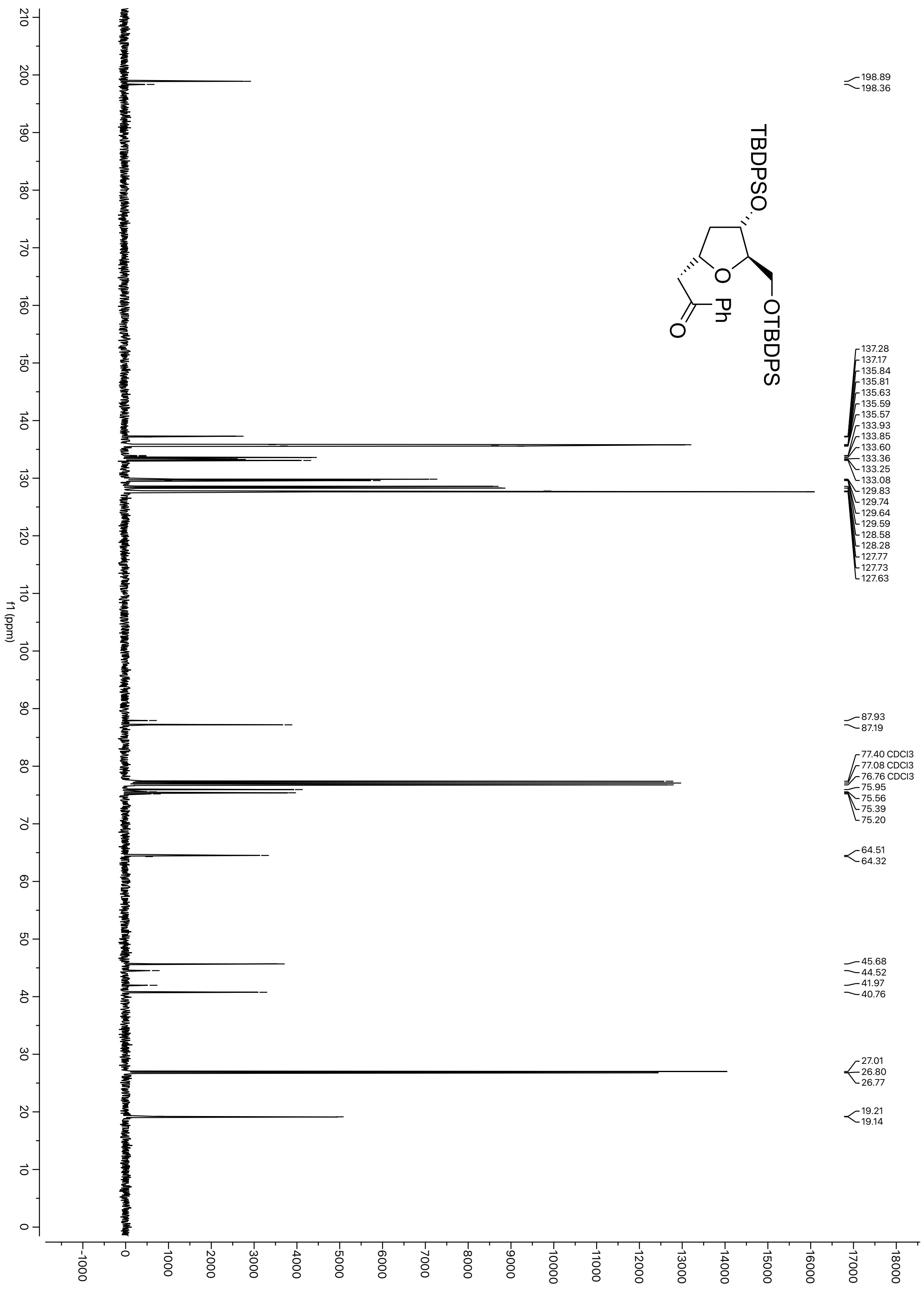
TBDPSO
OTBDPS
O
Ph
O
198.89
198.36
137.28
137.17
135.84
135.81
135.63
135.59
135.57
133.93
133.85
133.60
133.36
133.25
133.08
129.83
129.74
129.64
129.59
128.58
128.28
127.77
127.73
127.63
87.93
87.19
77.40 CDCl3
77.08 CDCl3
76.76 CDCl3
75.95
75.56
75.39
75.20
64.51
64.32
45.68
44.52
41.97
40.76
27.01
26.80
26.77
19.21
19.14
f1 (ppm)

**$^{1}H$-$^{13}C\{^{1}H\}$ NMR Spectra (Compound 22, 400 MHz, $CDCl_3$)**

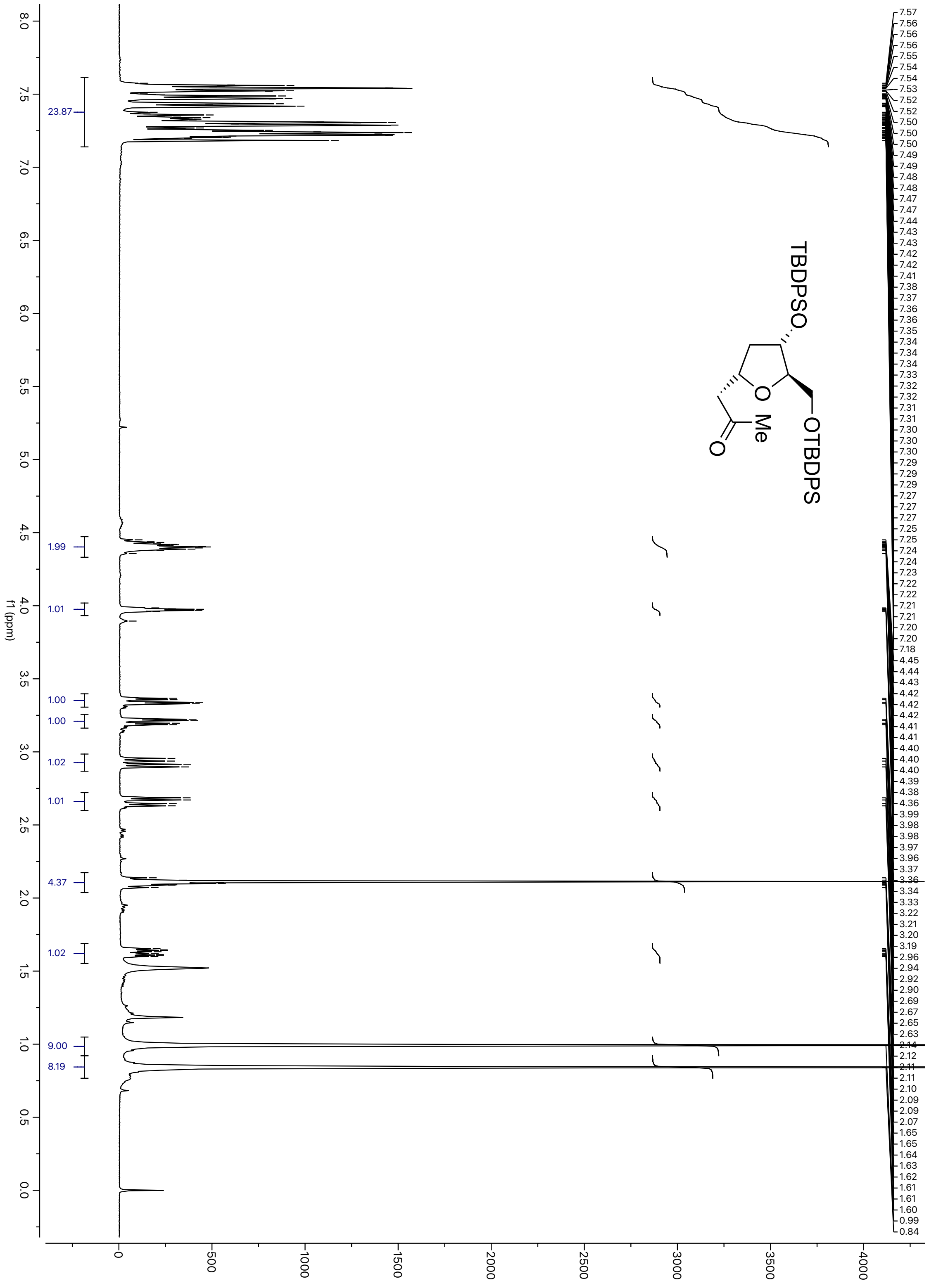

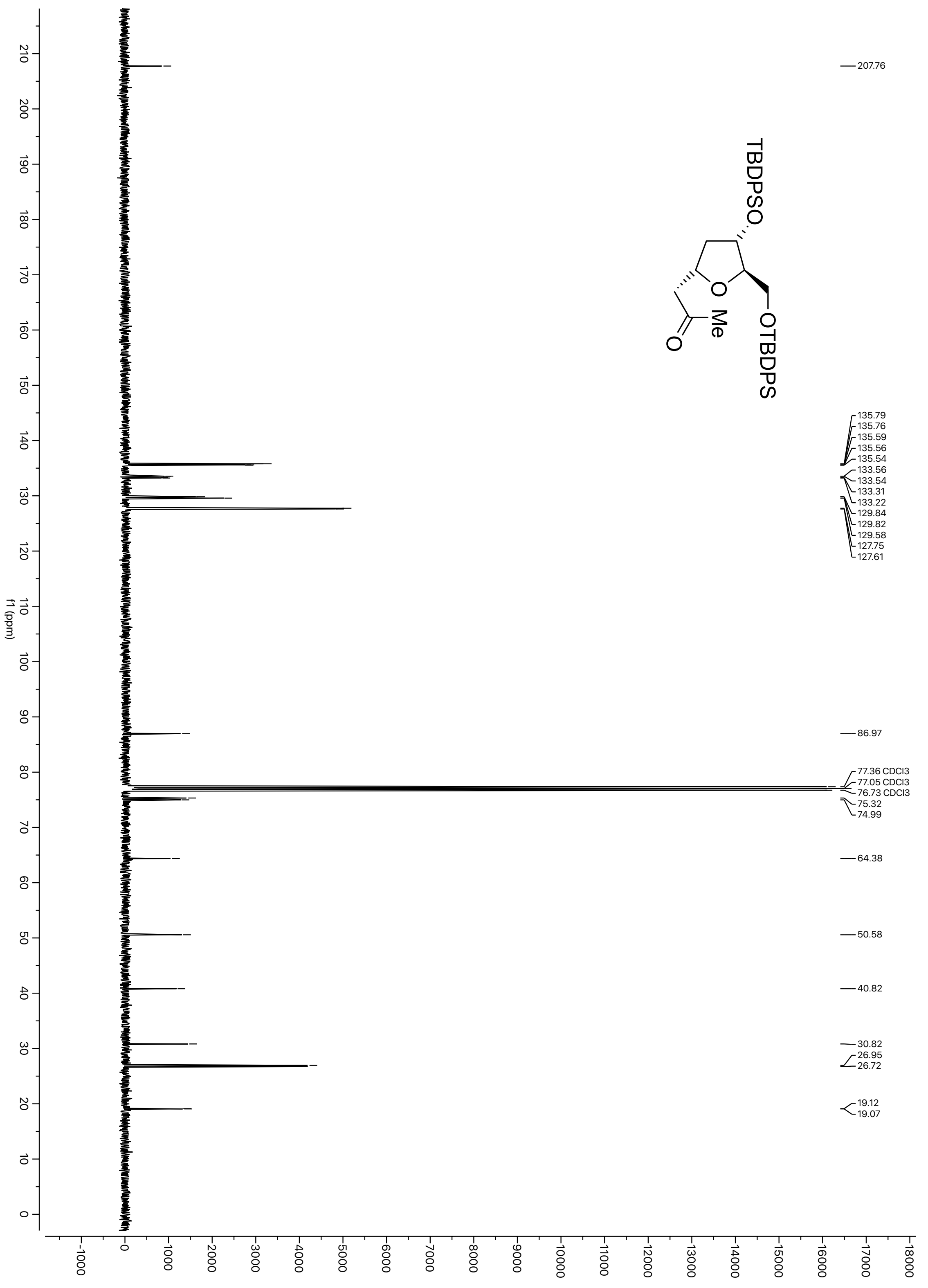

TBDPSO
OTBDPS
O
Me
O
207.76
135.79
135.76
135.59
135.56
135.54
133.56
133.54
133.31
133.22
129.84
129.82
129.58
127.75
127.61
86.97
77.36 CDCl3
77.05 CDCl3
76.73 CDCl3
75.32
74.99
64.38
50.58
40.82
30.82
26.95
26.72
19.12
19.07
f1 (ppm)

**$^{1}$H-$^{13}$C{$^{1}$H} NMR Spectra (Compound 23, 400 MHz, $CD_2Cl_2$)**

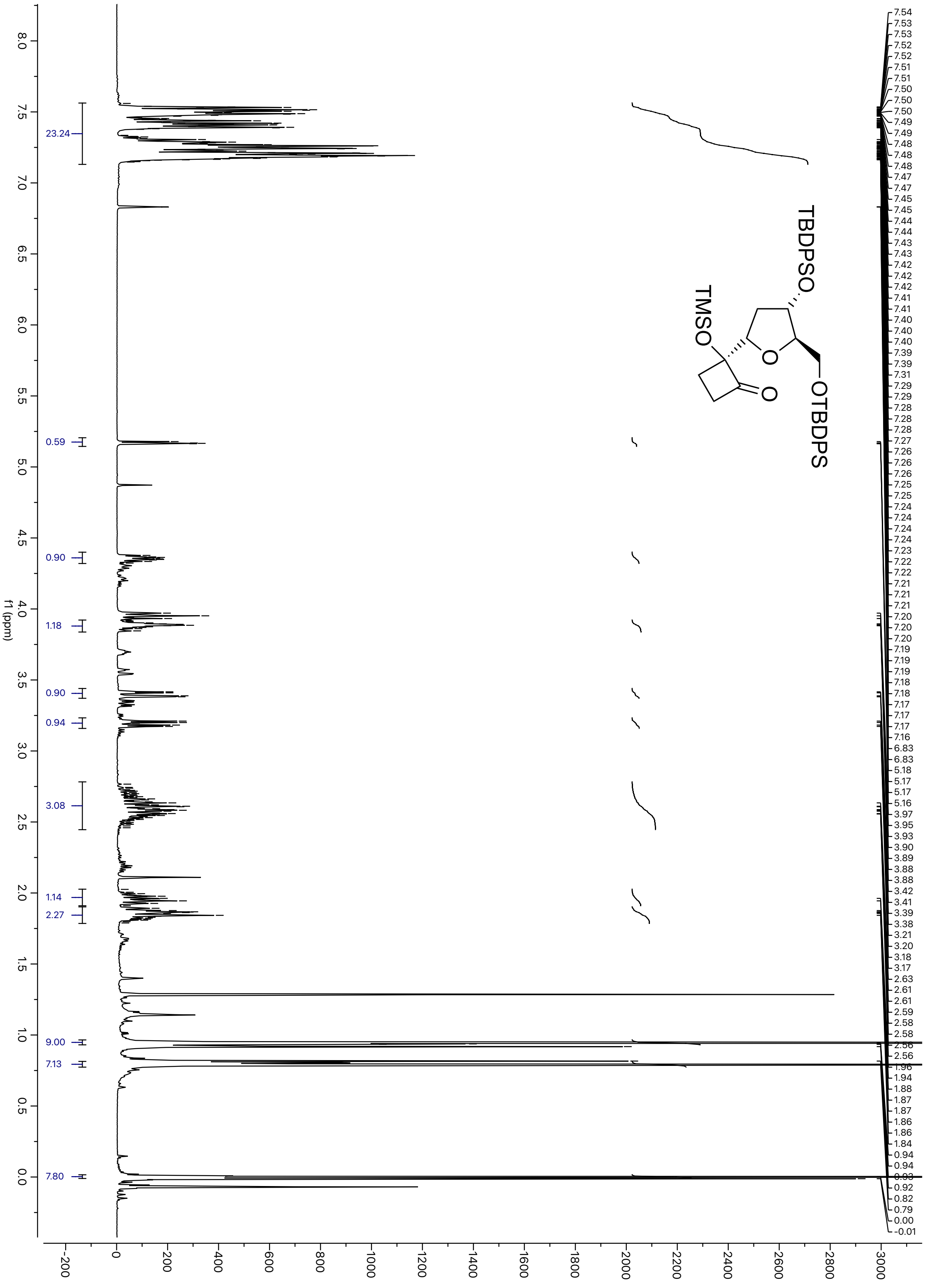

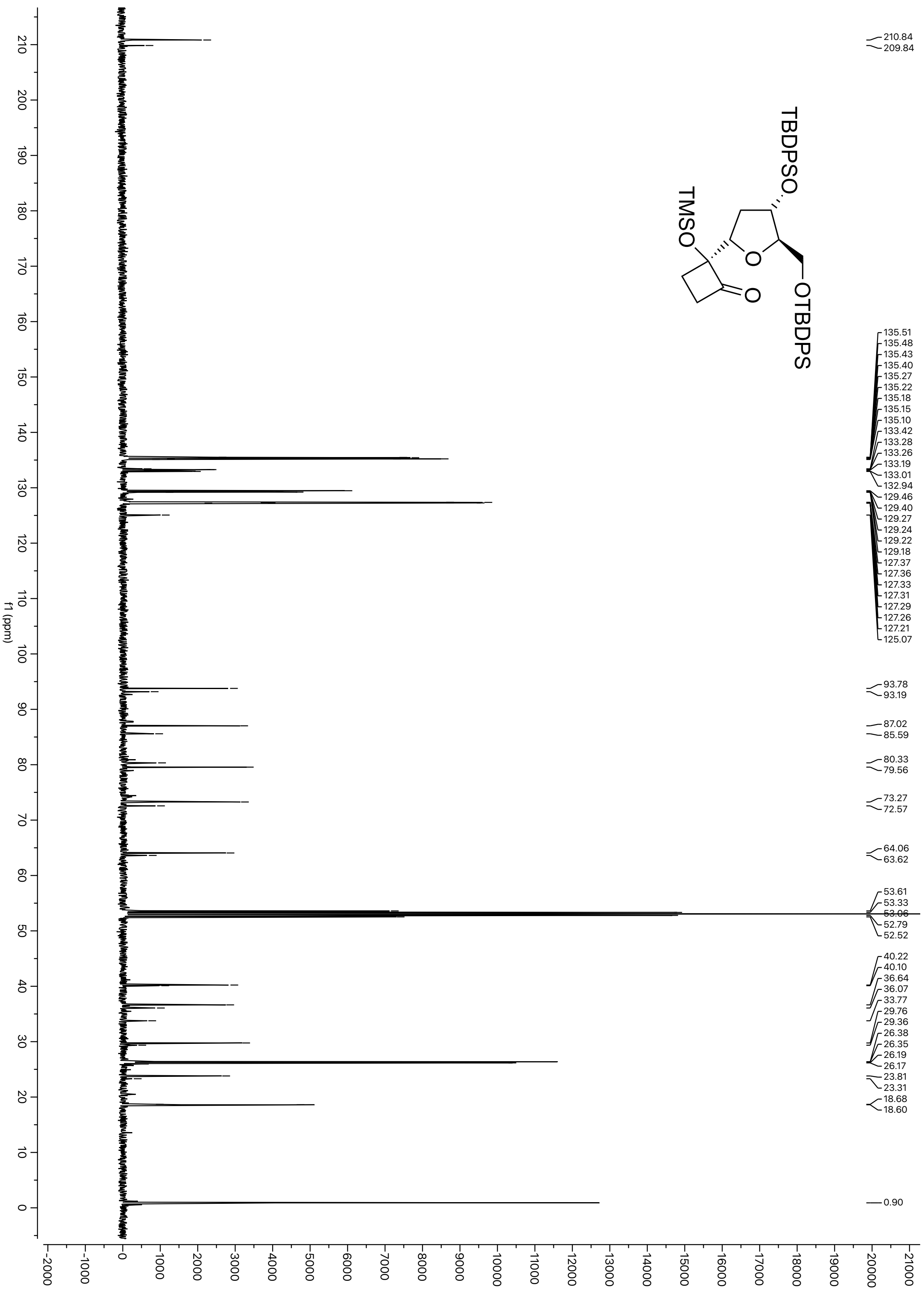

TBDPSO
TMSO
O
O
OTBDPS
210.84
209.84
135.51
135.48
135.43
135.40
135.27
135.22
135.18
135.15
135.10
133.42
133.28
133.26
133.19
133.01
132.94
129.46
129.40
129.27
129.24
129.22
129.18
127.37
127.36
127.33
127.31
127.29
127.26
127.21
125.07
93.78
93.19
87.02
85.59
80.33
79.56
73.27
72.57
64.06
63.62
53.61
53.33
53.06
52.79
52.52
40.22
40.10
36.64
36.07
33.77
29.76
29.36
26.38
26.35
26.19
26.17
23.81
23.31
18.68
18.60
0.90
f1 (ppm)

## $^{1}$H-$^{13}$C{$^{1}$H} NMR Spectra (Compound 24, 400 MHz, $CDCl_3$)

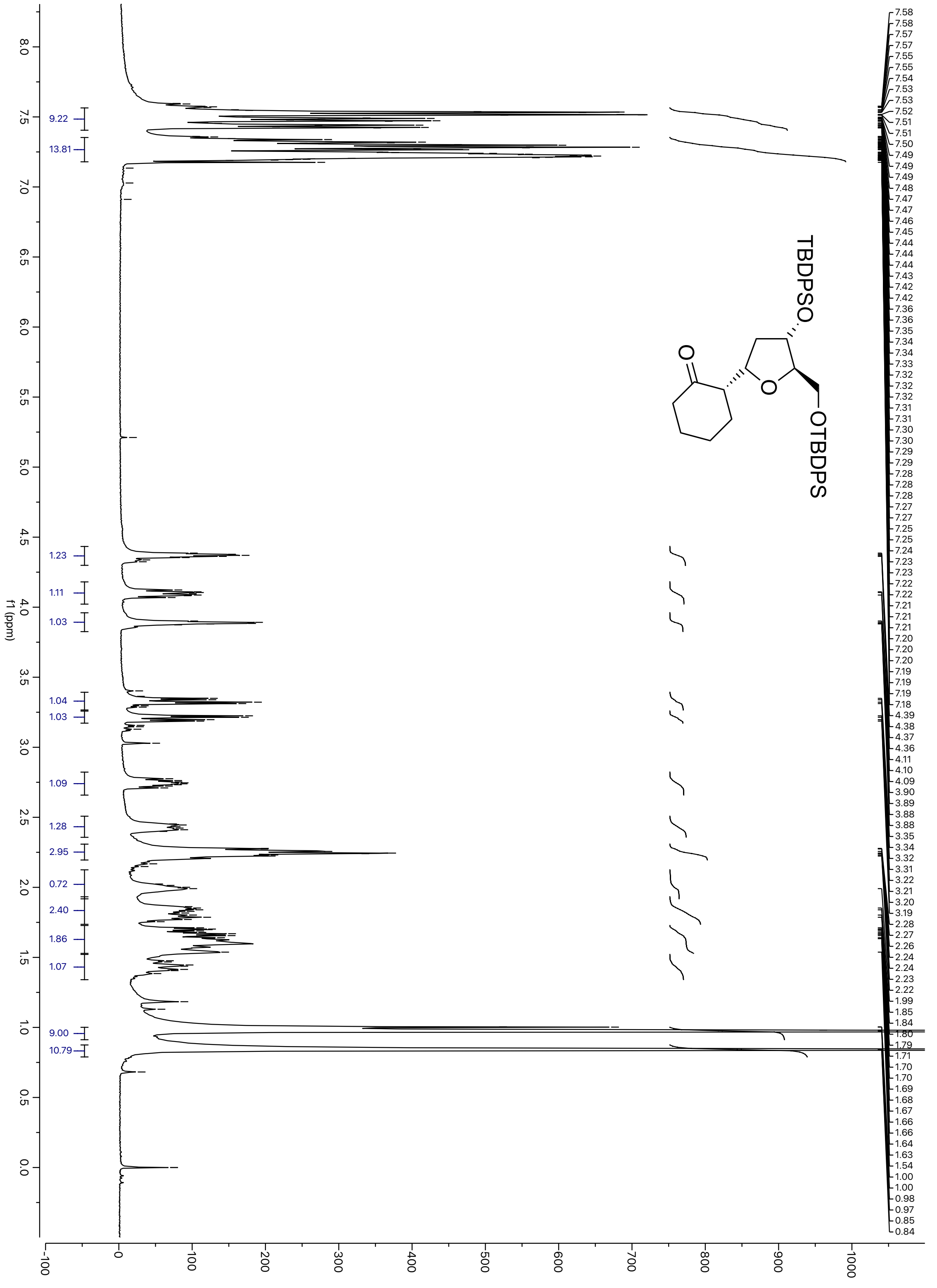

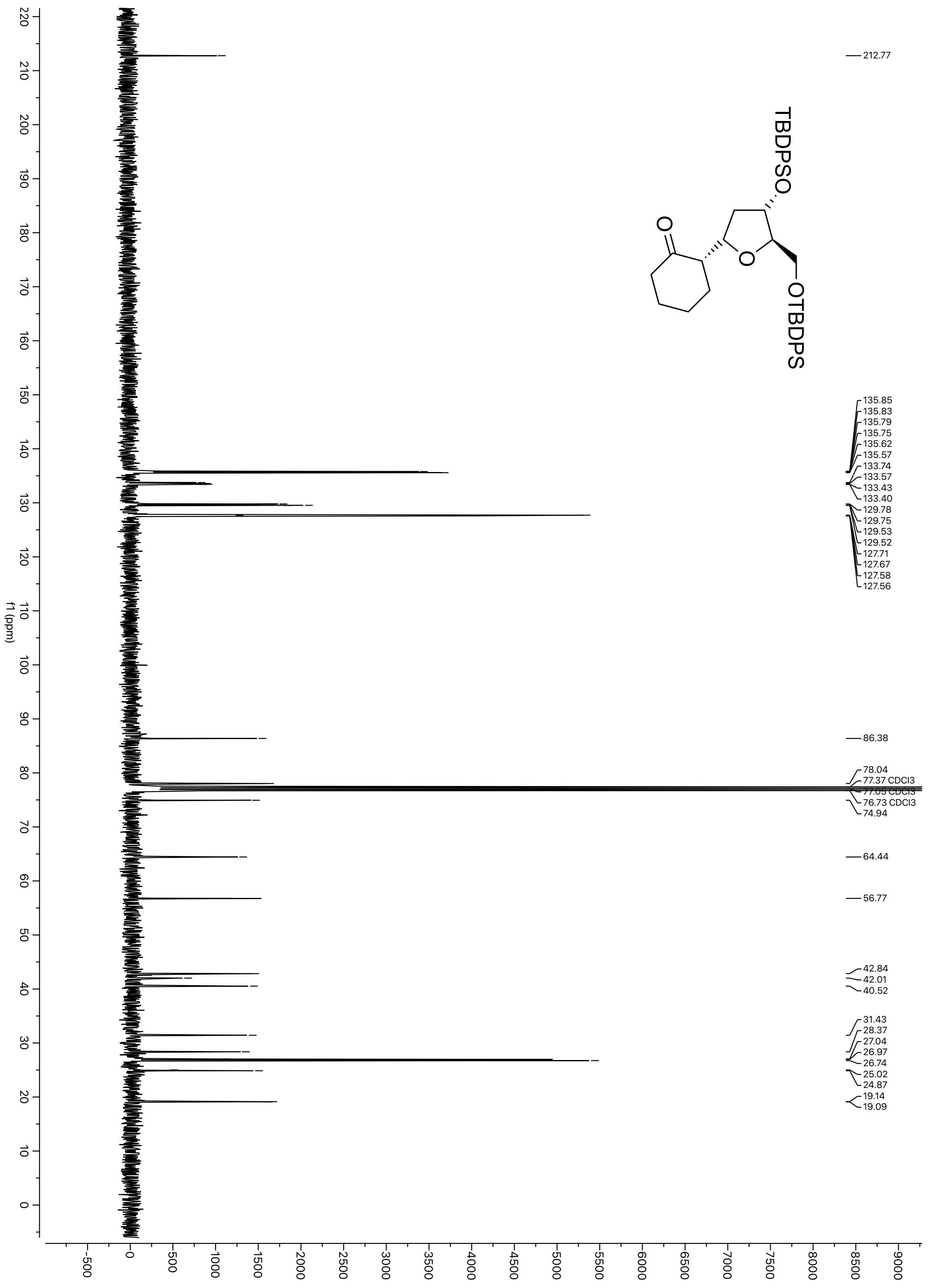
TBDPSO
OTBDPS
O
O
212.77
135.85
135.83
135.79
135.75
135.62
135.57
133.74
133.57
133.43
133.40
129.78
129.75
129.53
129.52
127.71
127.67
127.58
127.56
86.38
78.04
77.37 CDCl3
77.05 CDCl3
76.73 CDCl3
74.94
64.44
56.77
42.84
42.01
40.52
31.43
28.37
27.04
26.97
26.74
25.02
24.87
19.14
19.09
f1 (ppm)

**$^{1}$H-$^{13}$C{$^{1}$H} NMR Spectra (Compound 25, 400 MHz, $CDCl_3$)**

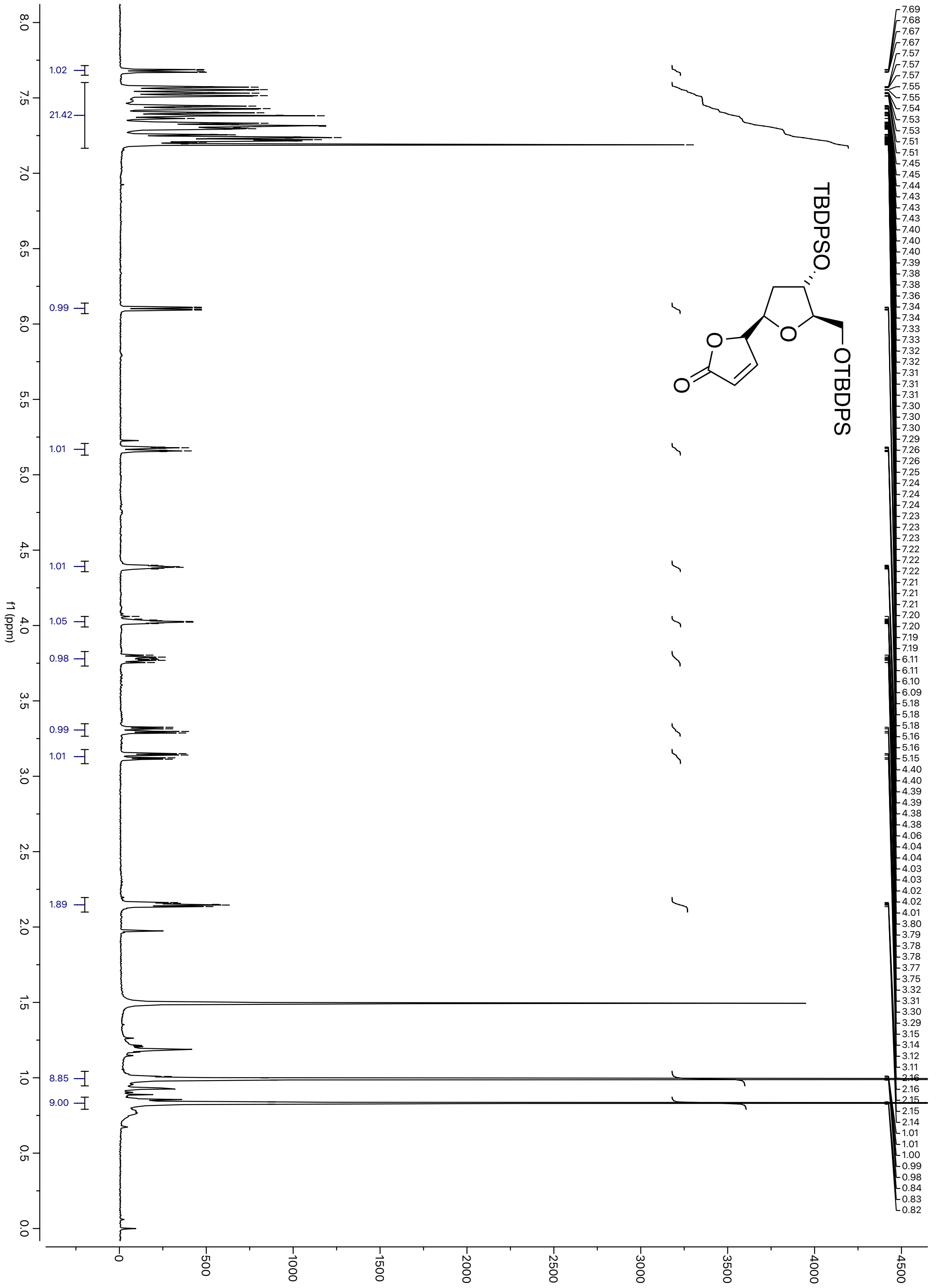

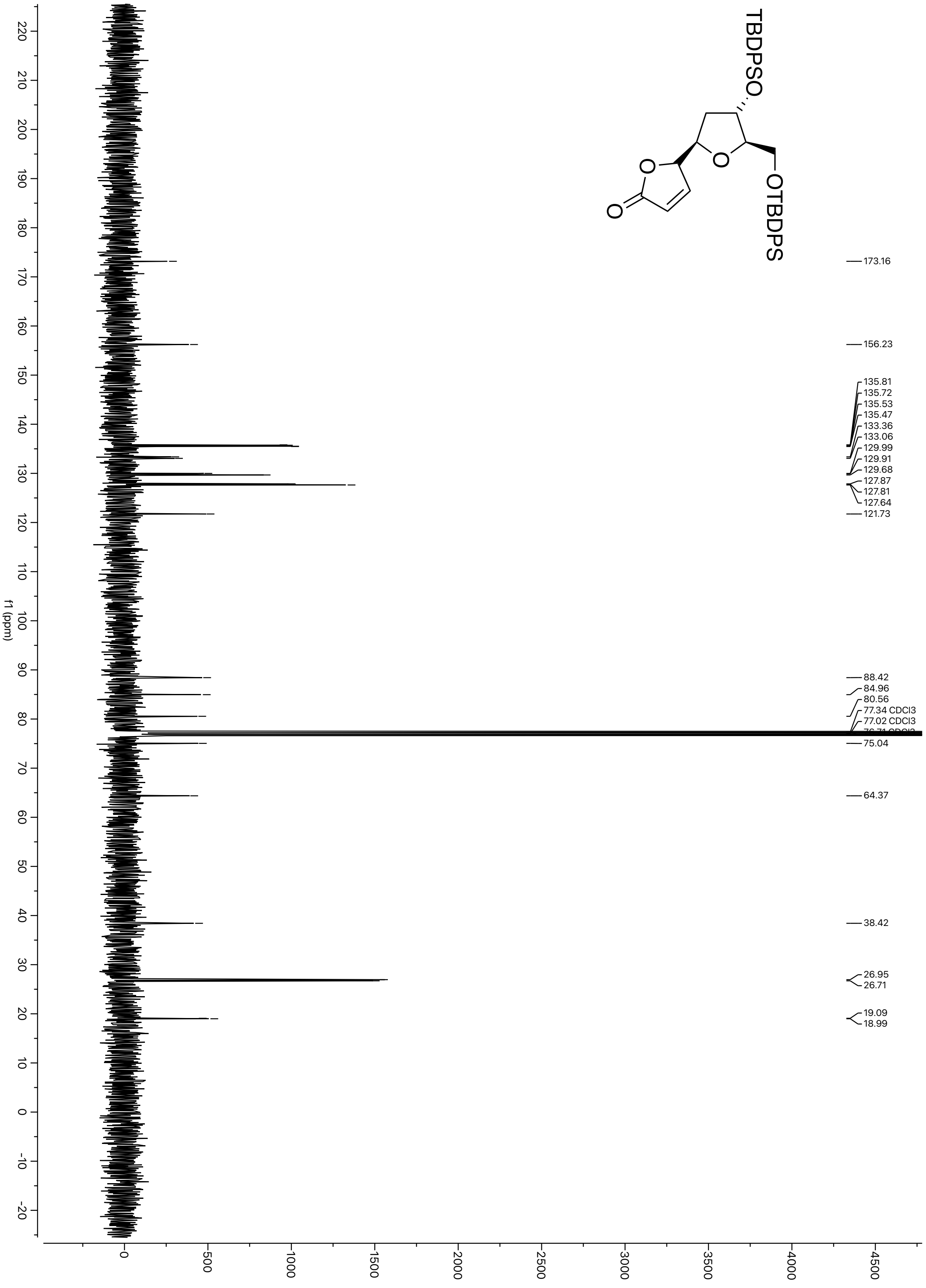
TBDPSO
OTBDPS
O
O
O
173.16
156.23
135.81
135.72
135.53
135.47
133.36
133.06
129.99
129.91
129.68
127.87
127.81
127.64
121.73
88.42
84.96
80.56
77.34 CDCl3
77.02 CDCl3
75.04
64.37
38.42
26.95
26.71
19.09
18.99
f1 (ppm)

## $^{1}H$-$^{13}C\{^{1}H\}$ NMR Spectra (Compound 26, 300 MHz, $CDCl_3$)

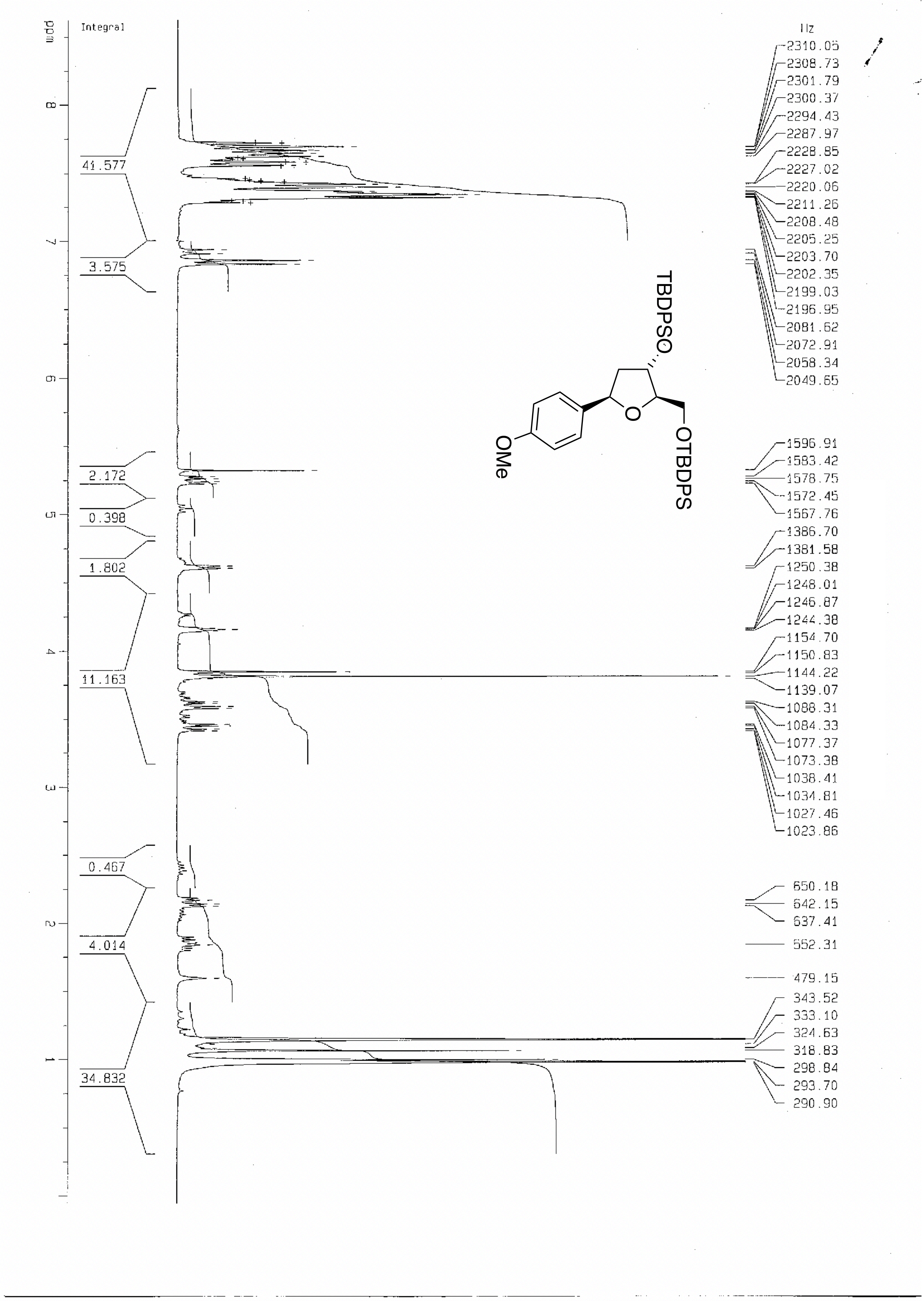

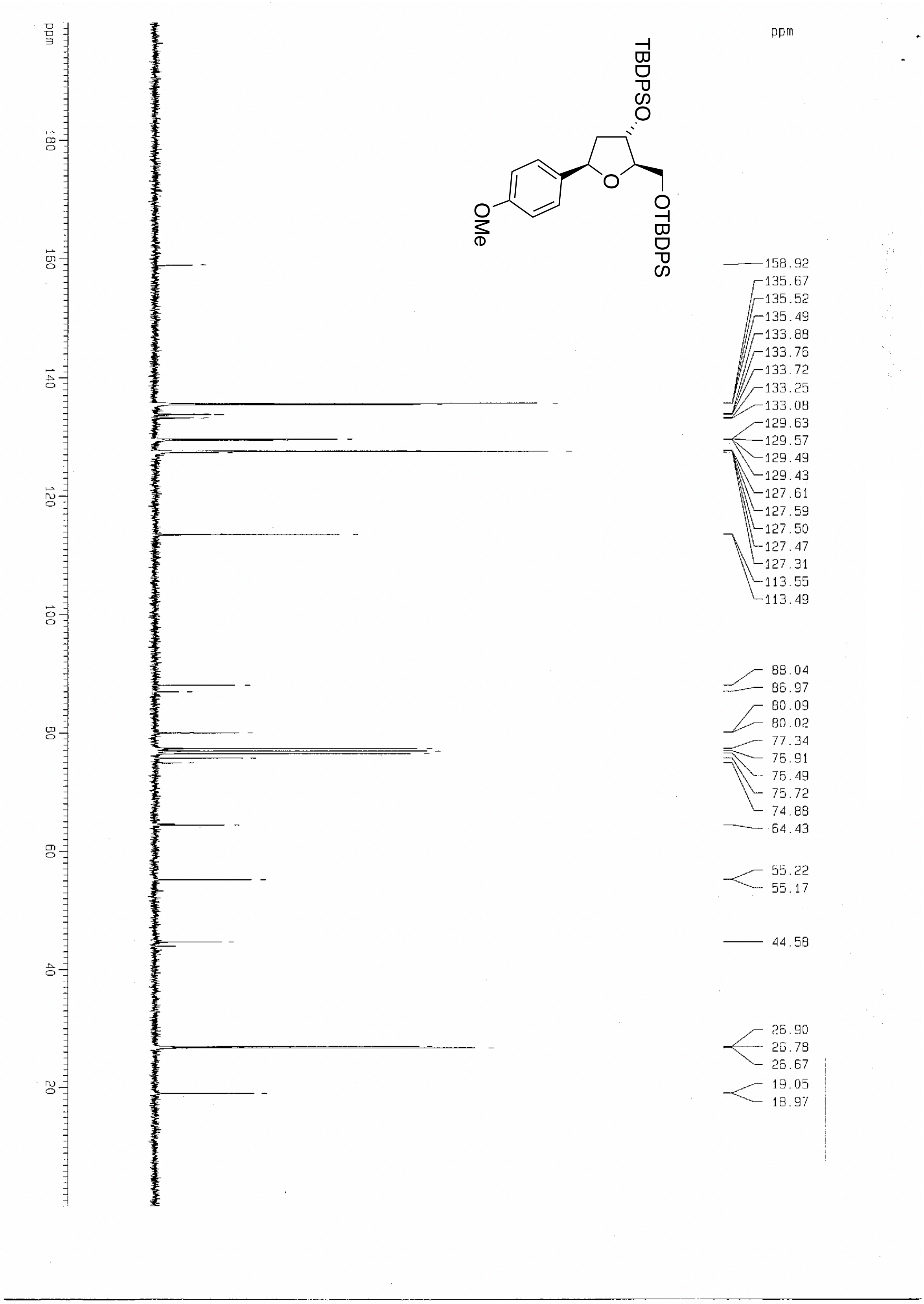
TBDPSO
OTBDPS
O
OMe
ppm
158.92
135.67
135.52
135.49
133.88
133.76
133.72
133.25
133.08
129.63
129.57
129.49
129.43
127.61
127.59
127.50
127.47
127.31
113.55
113.49
88.04
86.97
80.09
80.02
77.34
76.91
76.49
75.72
74.88
64.43
55.22
55.17
44.58
26.90
26.78
26.67
19.05
18.97

**$^{1}$H-$^{13}$C{$^{1}$H} NMR Spectra (Compound 27, 300 MHz, $CDCl_3$)**

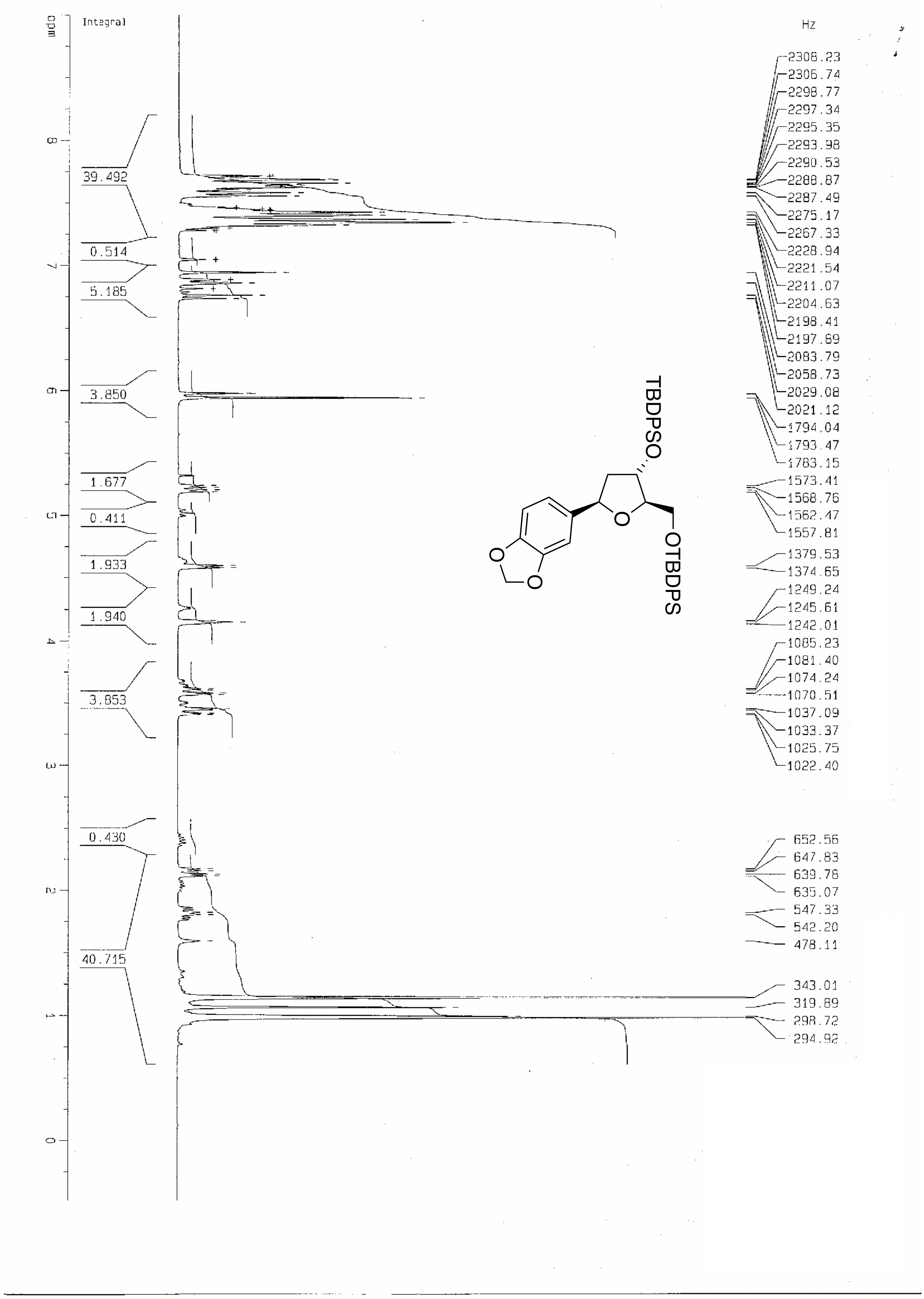

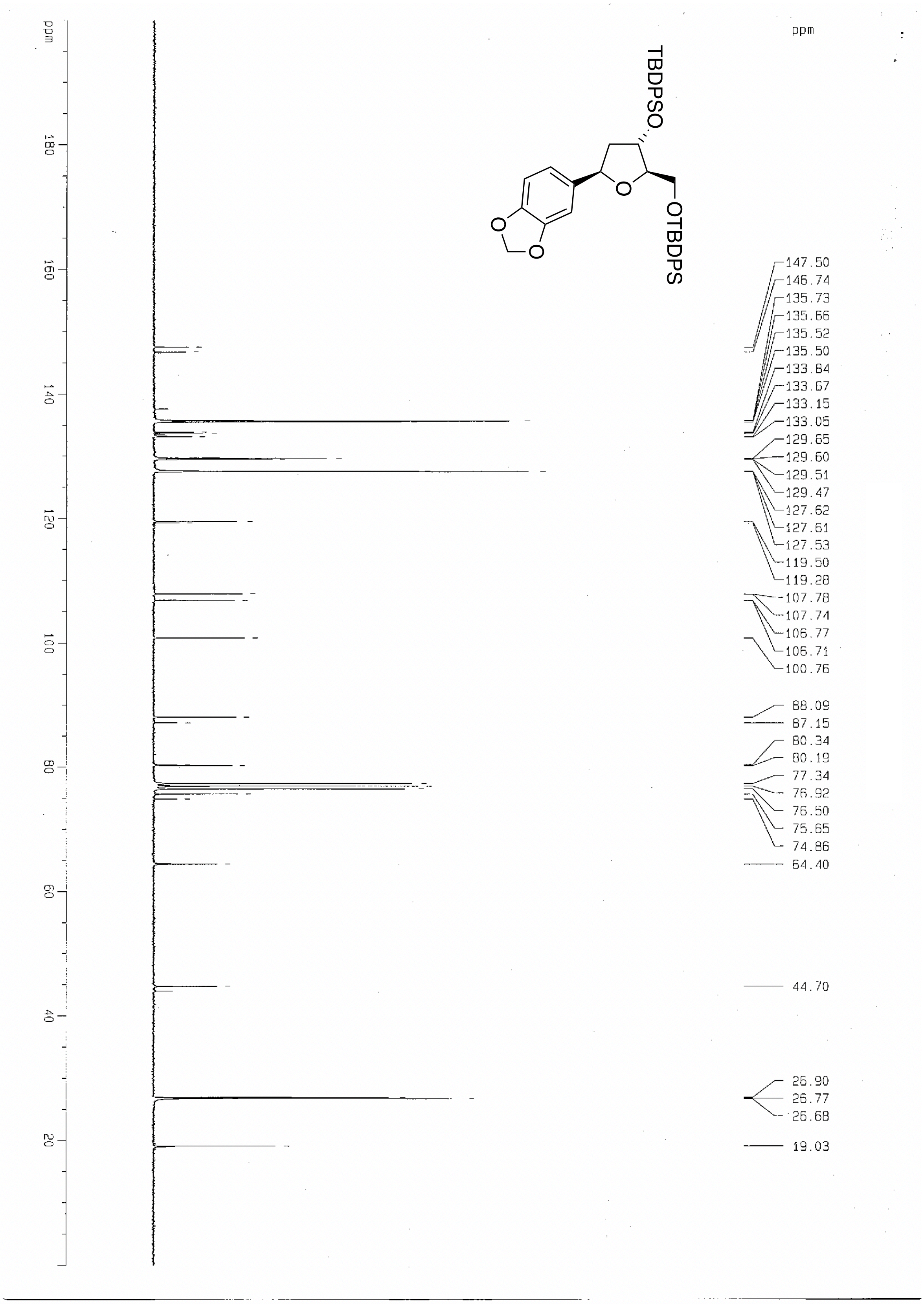
ppm
TBDPSO
OTBDPS
147.50
146.74
135.73
135.66
135.52
135.50
133.84
133.67
133.15
133.05
129.65
129.60
129.51
129.47
127.62
127.61
127.53
119.50
119.28
107.78
107.74
106.77
106.71
100.76
88.09
87.15
80.34
80.19
77.34
76.92
76.50
75.65
74.86
64.40
44.70
26.90
26.77
26.68
19.03

**$^{1}$H-$^{13}$C{$^{1}$H} NMR Spectra (Compound 28, 400 MHz, $(CD_3)_2CO$)**

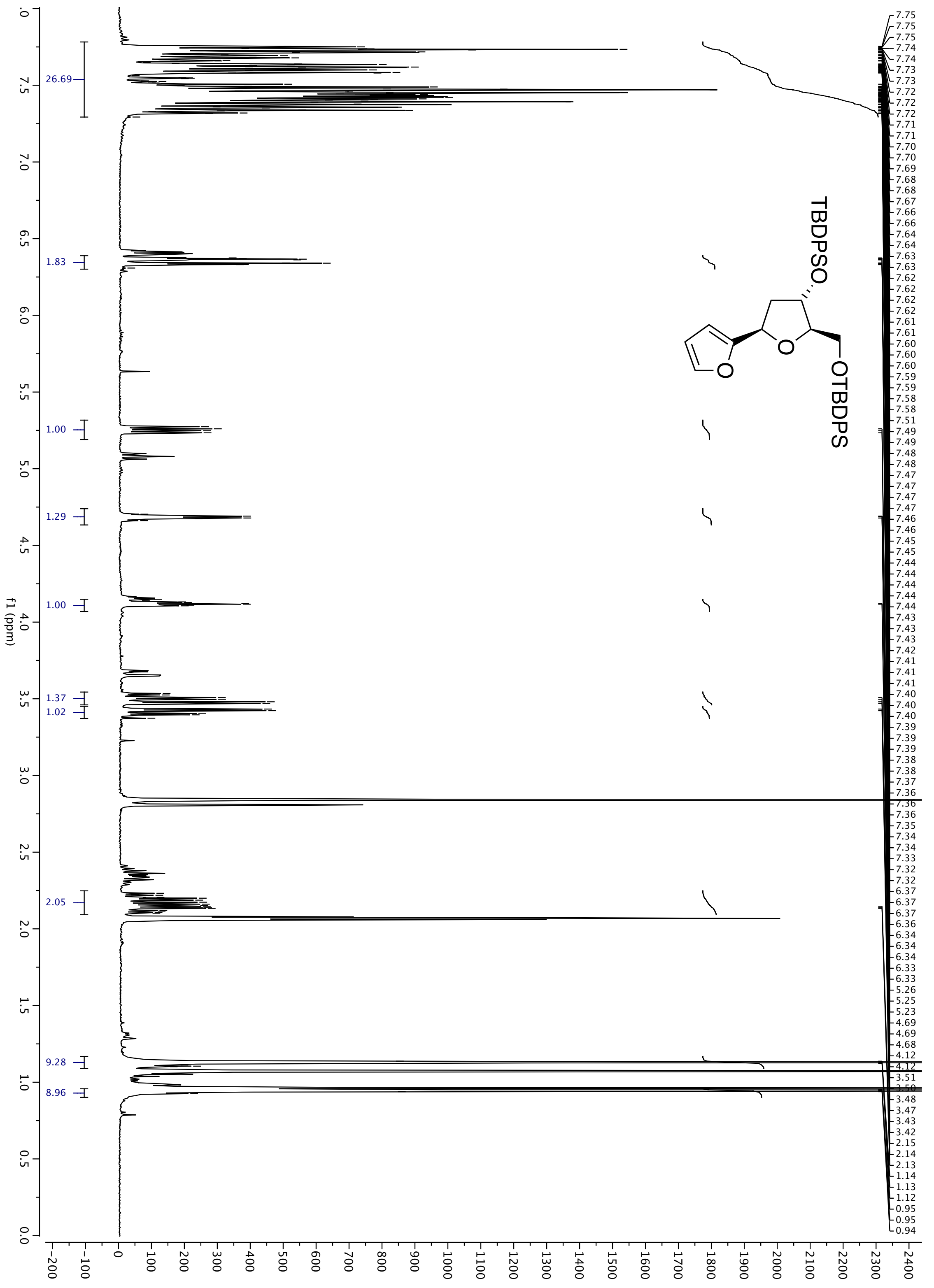

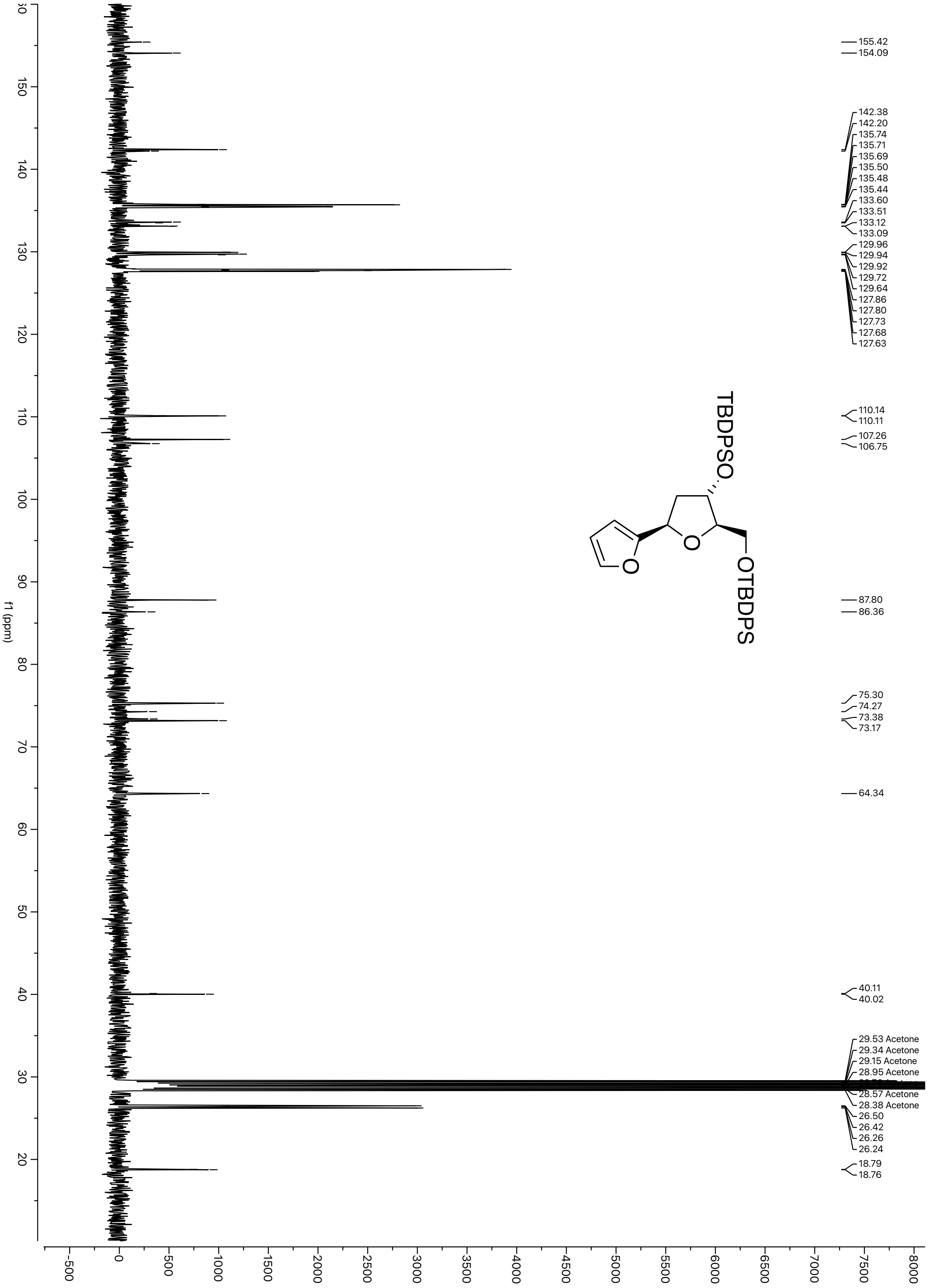
155.42
154.09
142.38
142.20
135.74
135.71
135.69
135.50
135.48
135.44
133.60
133.51
133.12
133.09
129.96
129.94
129.92
129.72
129.64
127.86
127.80
127.73
127.68
127.63
110.14
110.11
107.26
106.75
87.80
86.36
75.30
74.27
73.38
73.17
64.34
40.11
40.02
29.53 Acetone
29.34 Acetone
29.15 Acetone
28.95 Acetone
28.57 Acetone
28.38 Acetone
26.50
26.42
26.26
26.24
18.79
18.76
TBDPSO
OTBDPS
f1 (ppm)

$^{1}$H-$^{13}$C{$^{1}$H} NMR Spectra (Compound 35, 300 MHz, CDCl3)

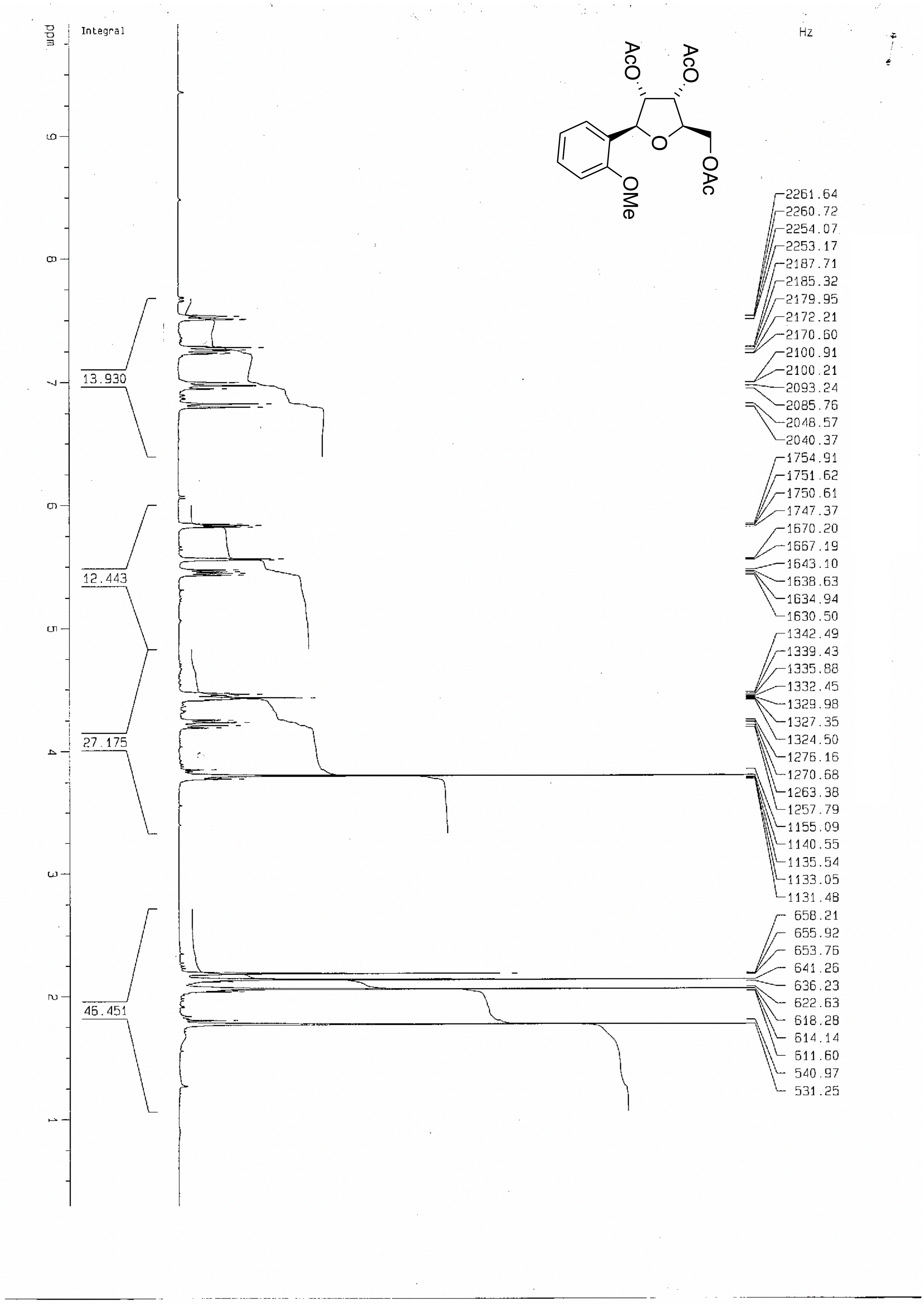

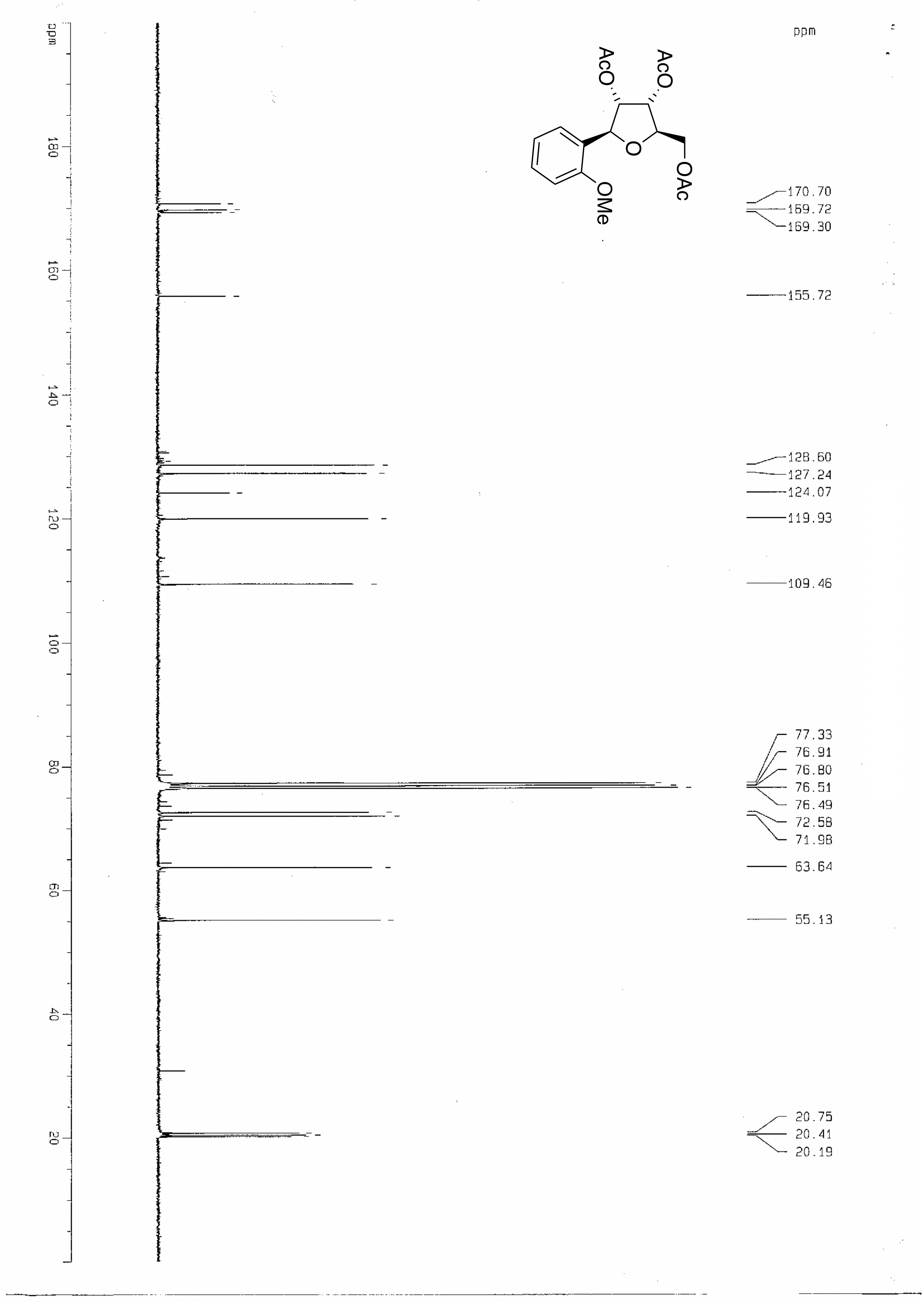

ppm
AcO
AcO
O
OAc
OMe
170.70
169.72
169.30
155.72
128.60
127.24
124.07
119.93
109.46
77.33
76.91
76.80
76.51
76.49
72.58
71.98
63.64
55.13
20.75
20.41
20.19
ppm
180
160
140
120
100
80
60
40
20

## $^{1}$H-$^{13}$C{$^{1}$H} NMR Spectra (Compound 37, 300 MHz, $CDCl_3$)

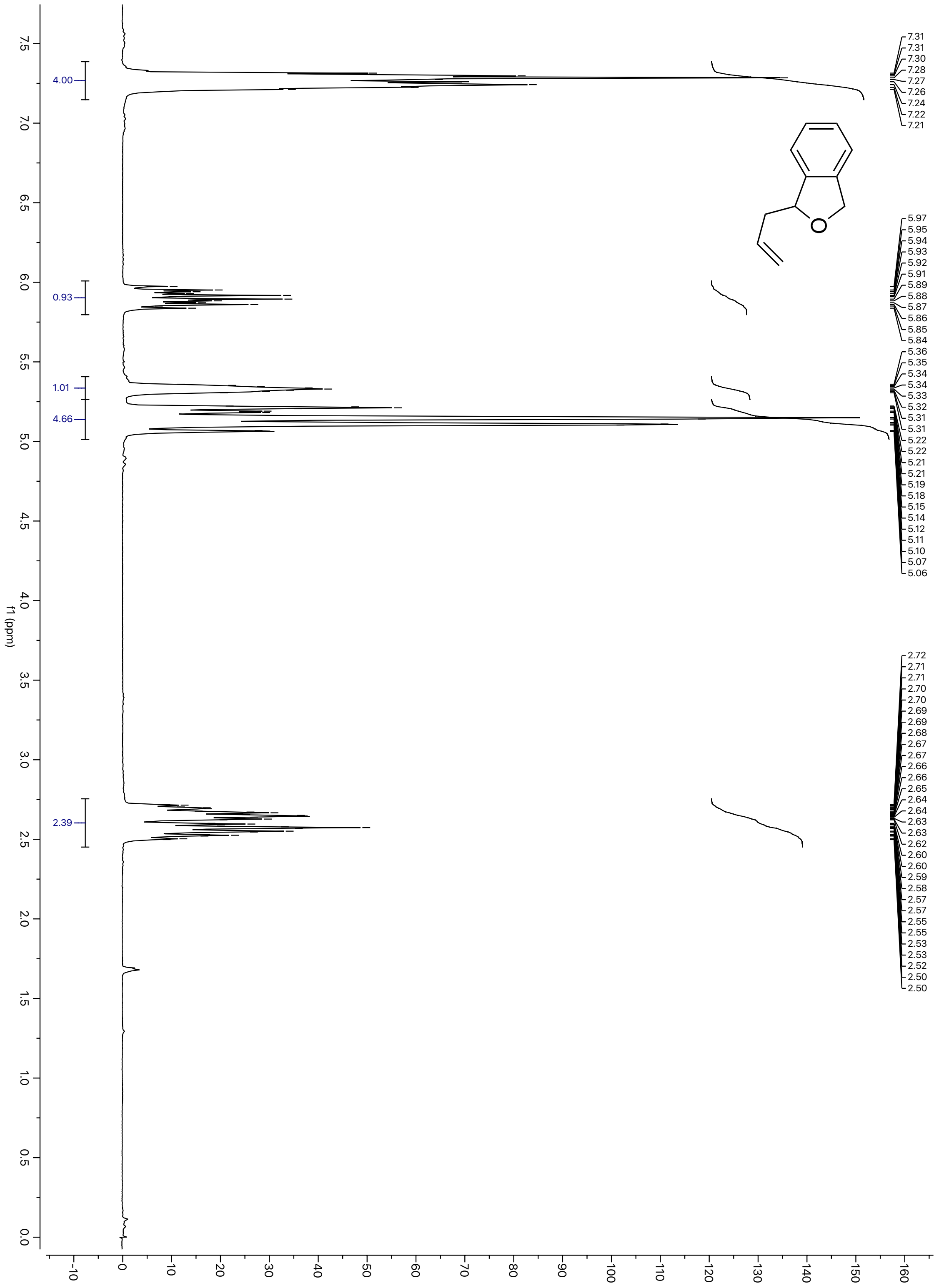

**$^{1}$H-$^{13}$C{$^{1}$H} NMR Spectra (Compound 39, 300 MHz, $CDCl_3$)**

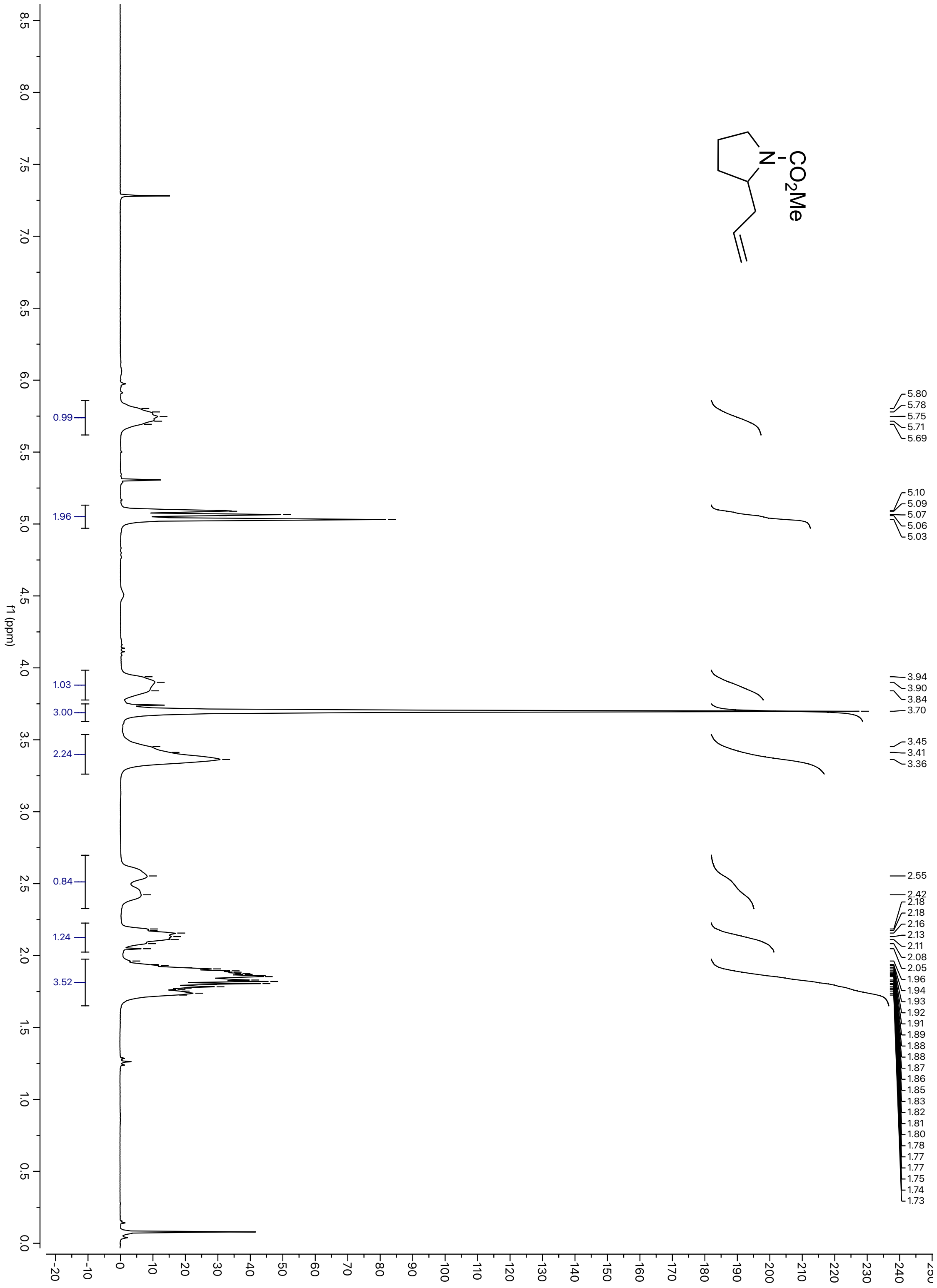

**$^{1}$H-$^{13}$C{$^{1}$H} NMR Spectra (Compound 41, 300 MHz, $CDCl_3$)**

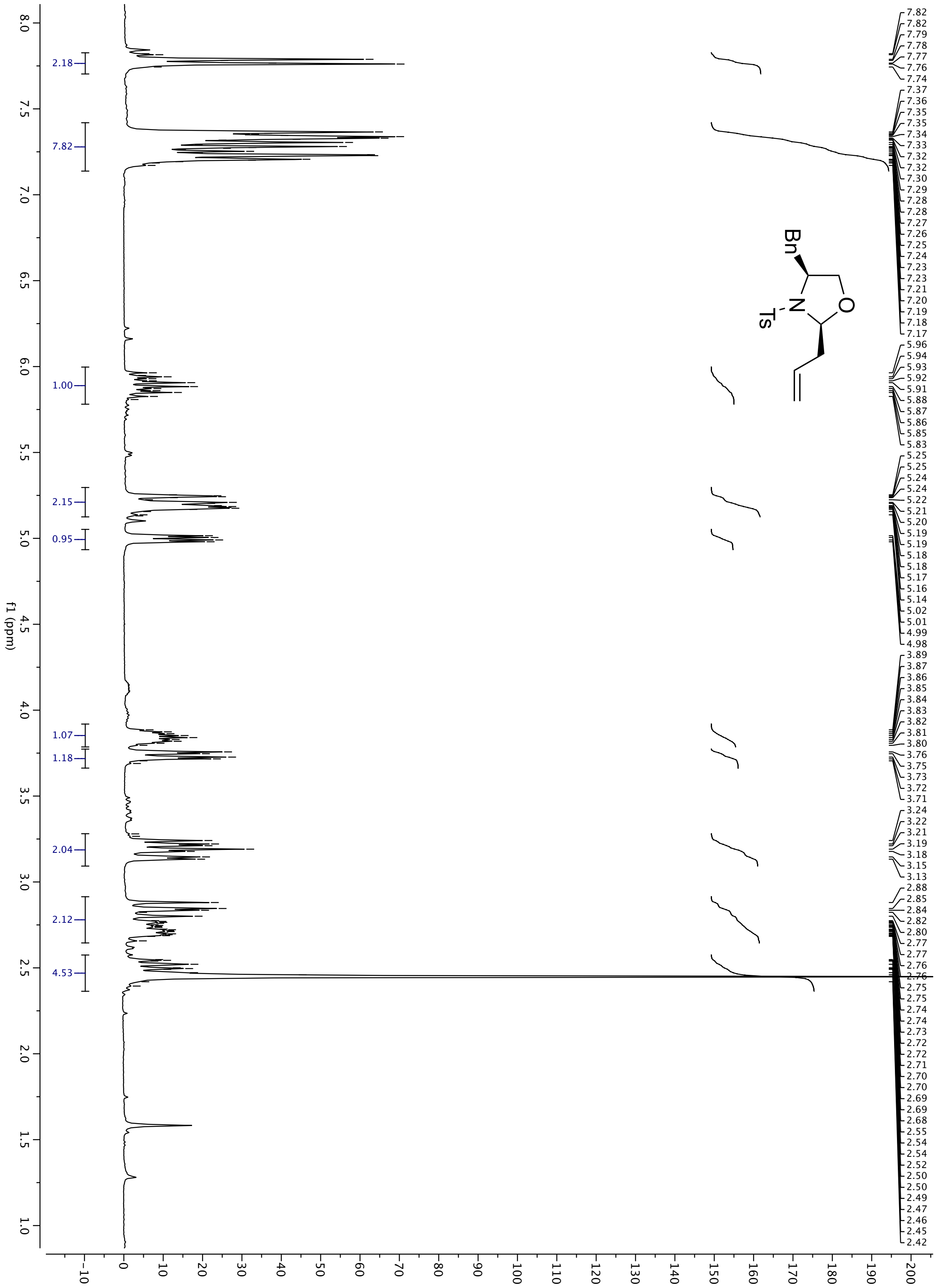

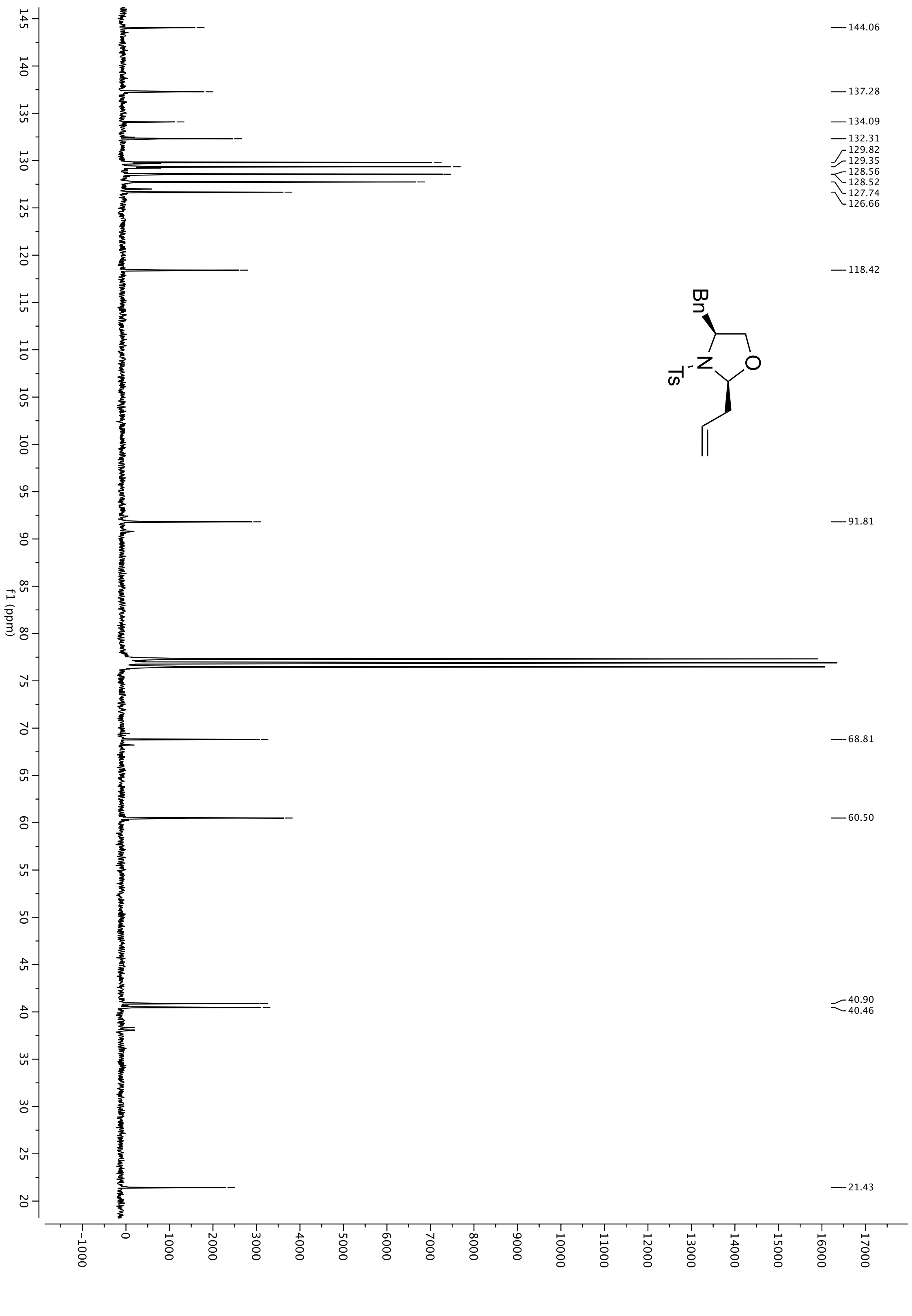
144.06
137.28
134.09
132.31
129.82
129.35
128.56
128.52
127.74
126.66
118.42
91.81
68.81
60.50
40.90
40.46
21.43
Bn
N
O
Ts
f1 (ppm)

**NOESY NMR Spectra and correlations (Compound 15, 200 MHz, $CDCl_3$)**

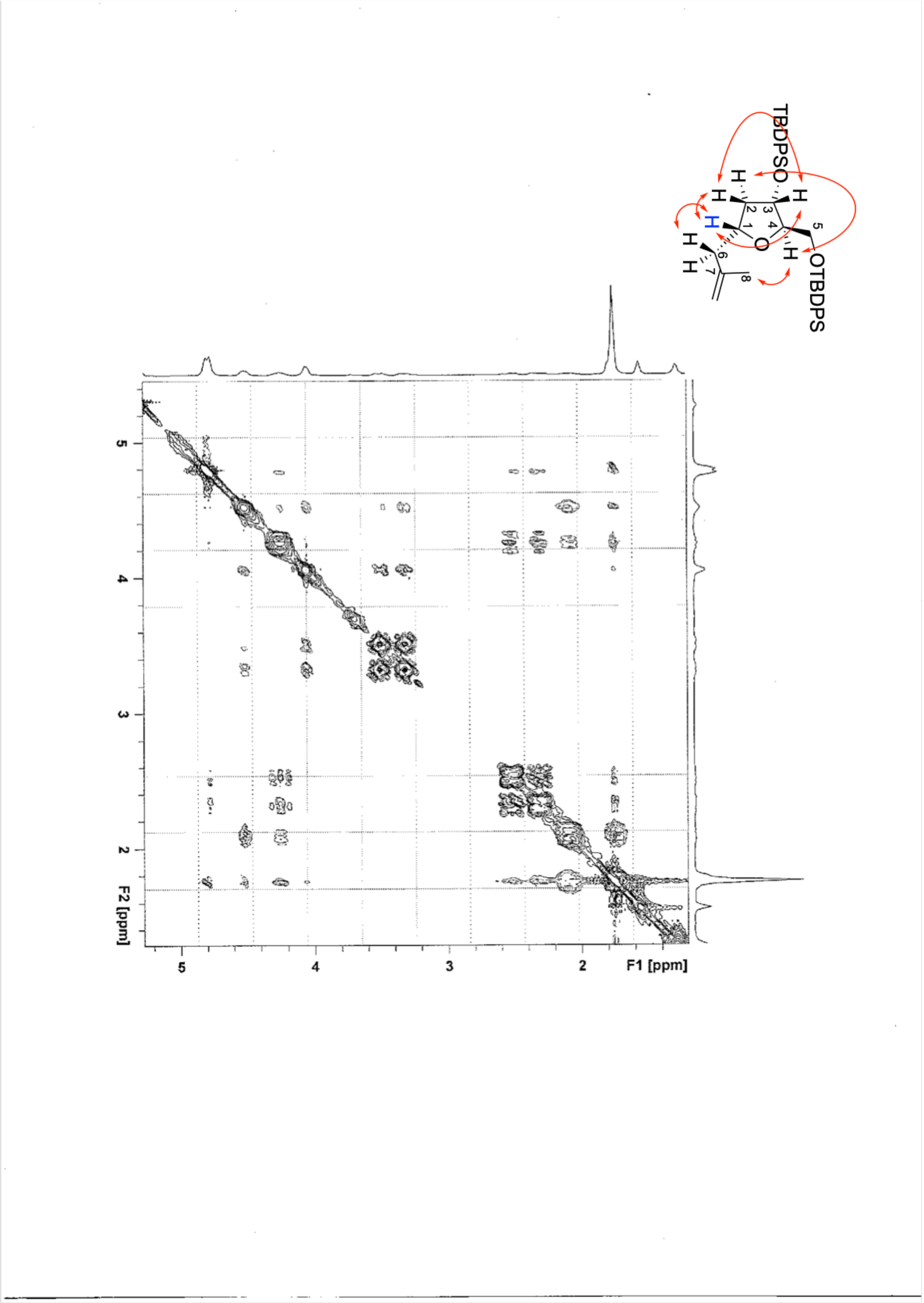

**NOESY NMR Spectra and correlations (Compound 23, 200 MHz, $CD_2Cl_2$)**

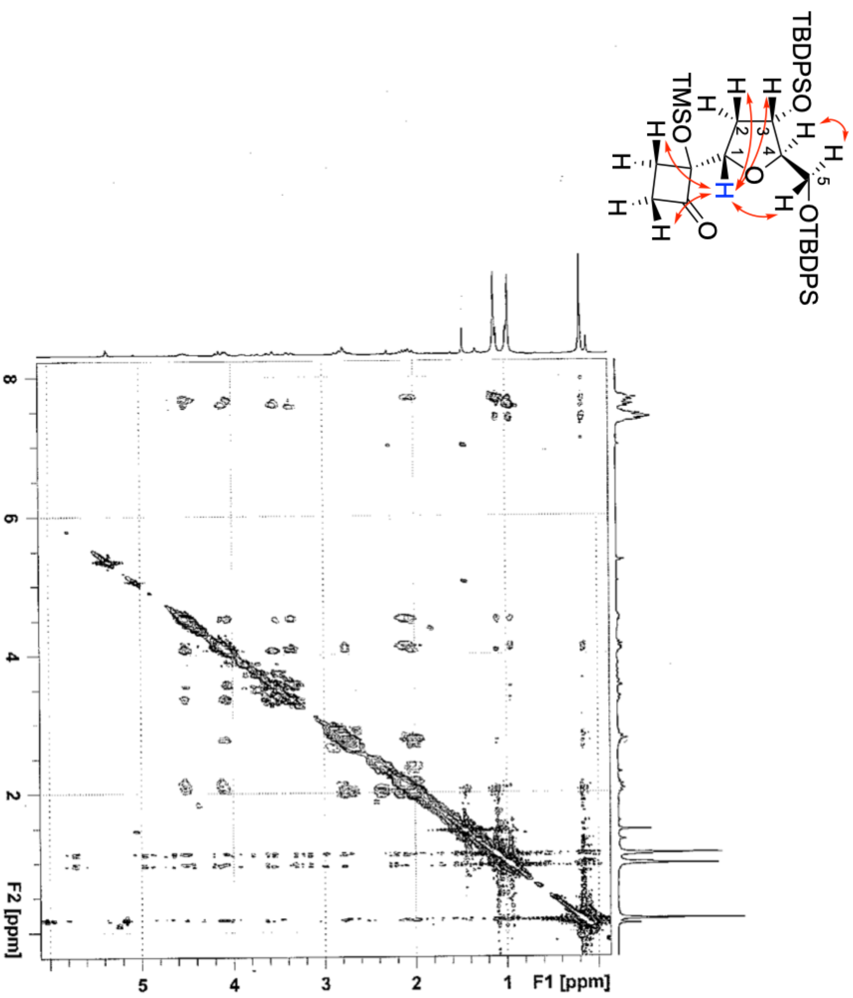

**NOESY NMR Spectra and correlations (Compound 25, 200 MHz, $CDCl_3$)**

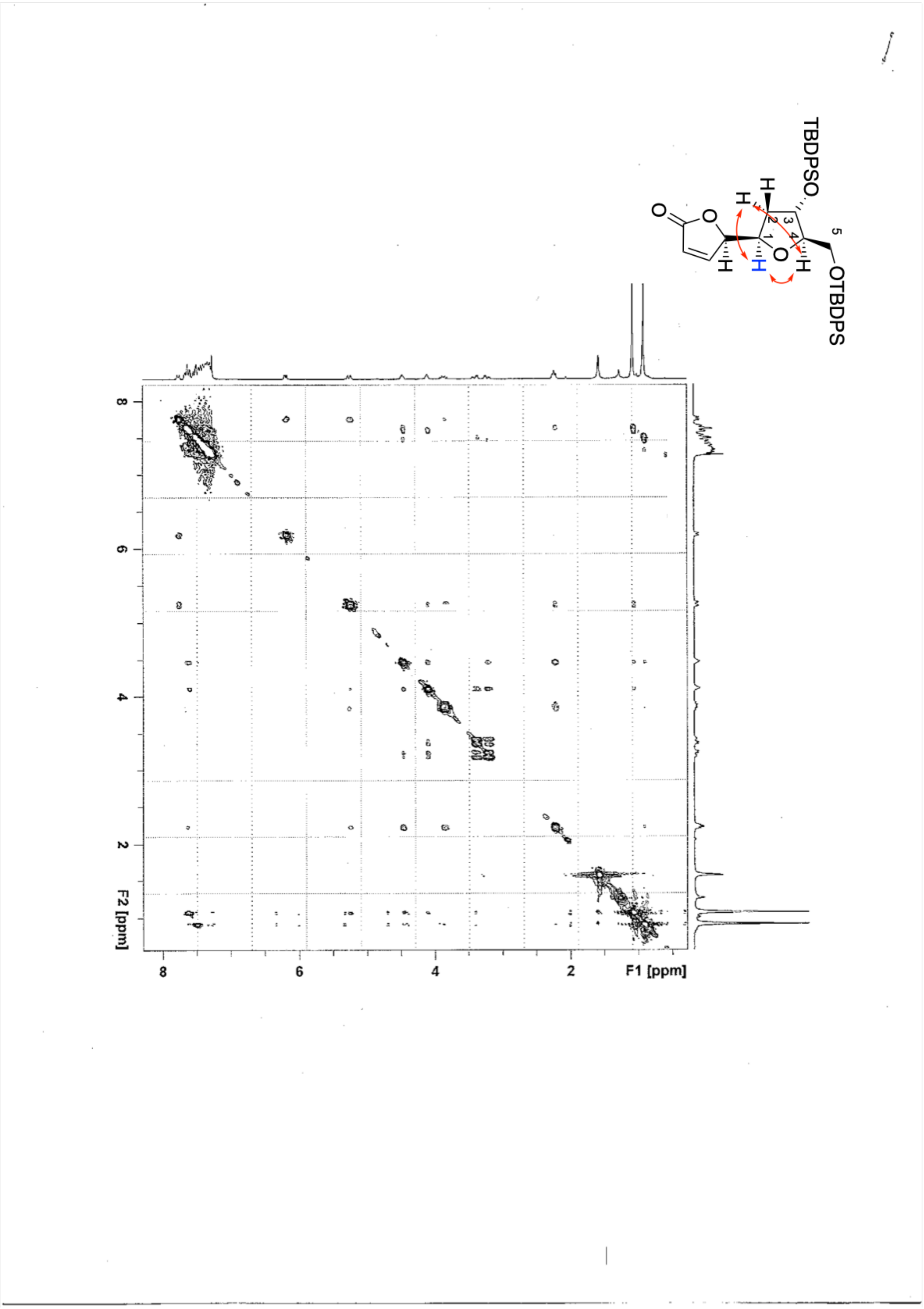

**NOESY NMR Spectra and correlations (Compound 26, 200 MHz, $CDCl_3$)**

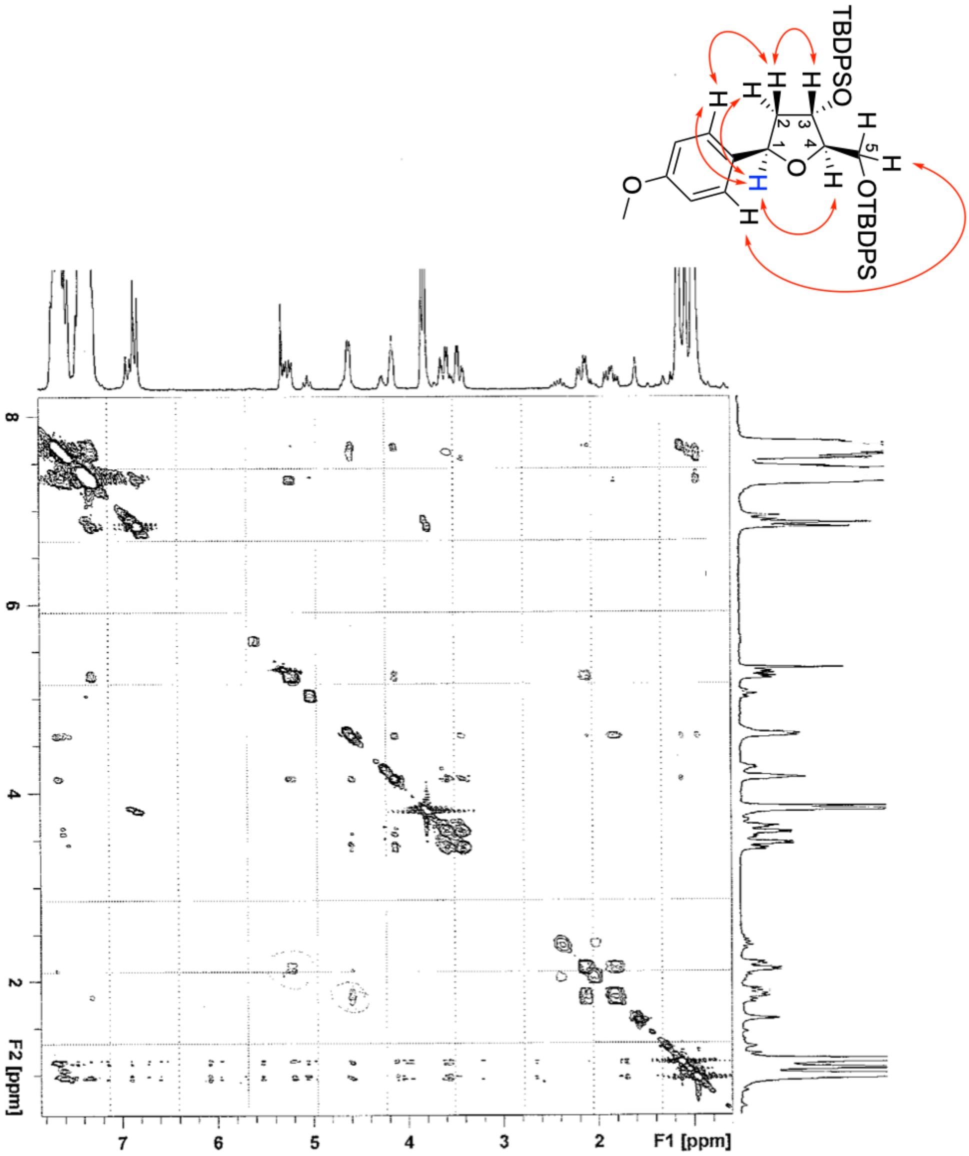

**NOESY NMR Spectra and correlations (Compound 27, 200 MHz, $CDCl_3$)**

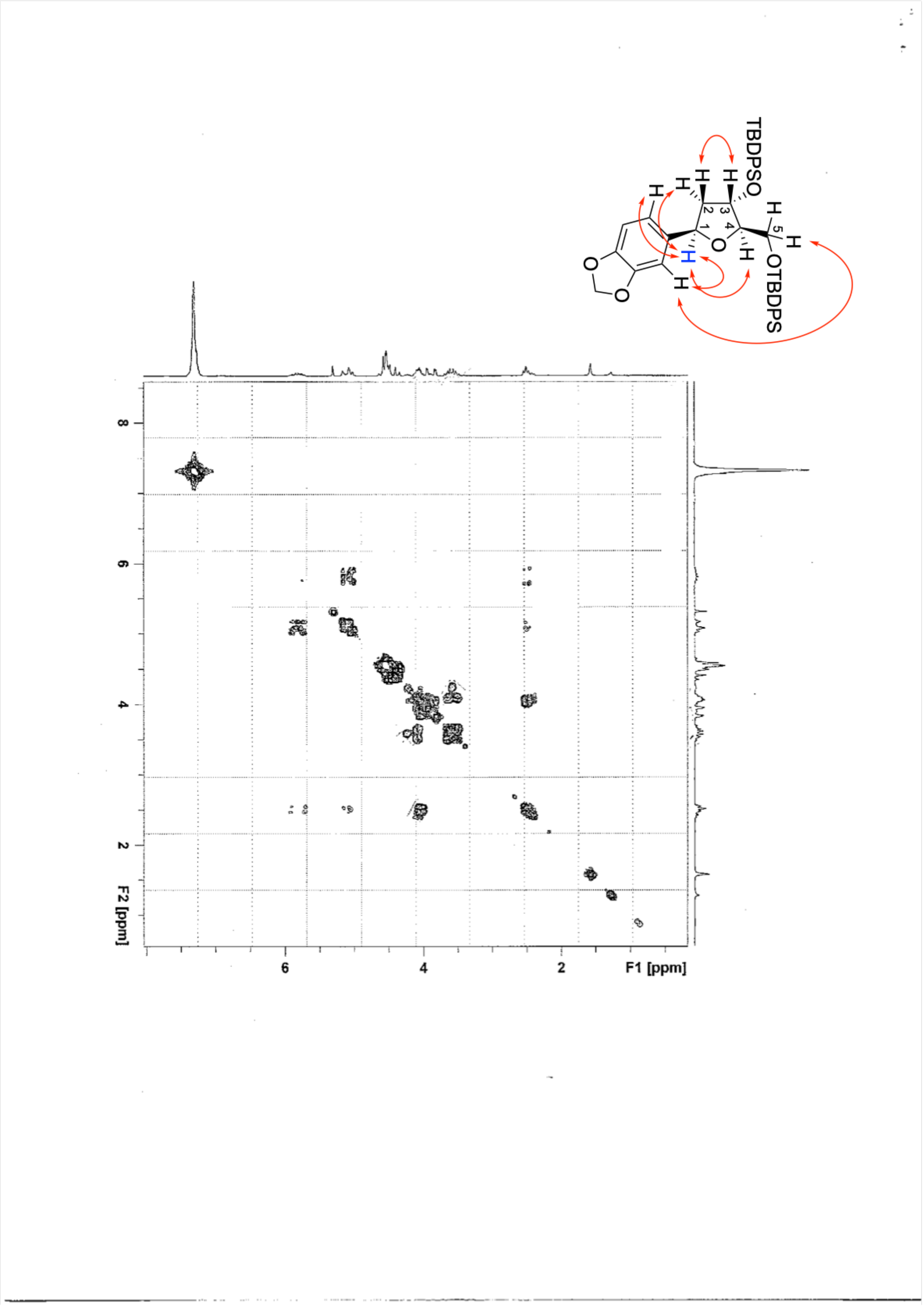

**NOESY NMR Spectra and correlations (Compound 33, 200 MHz, $CDCl_3$)**

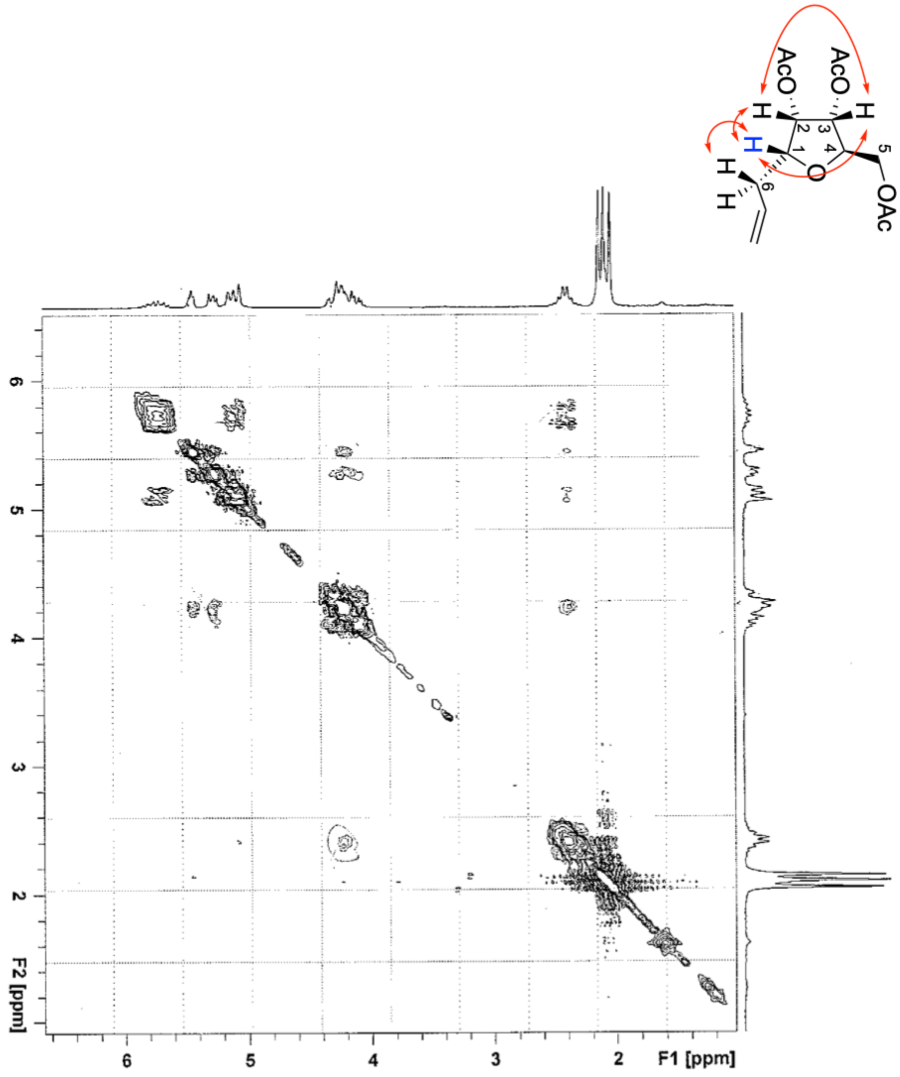

**NOESY NMR Spectra and correlations (Compound 34, 200 MHz, $CDCl_3$)**

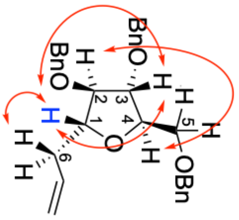

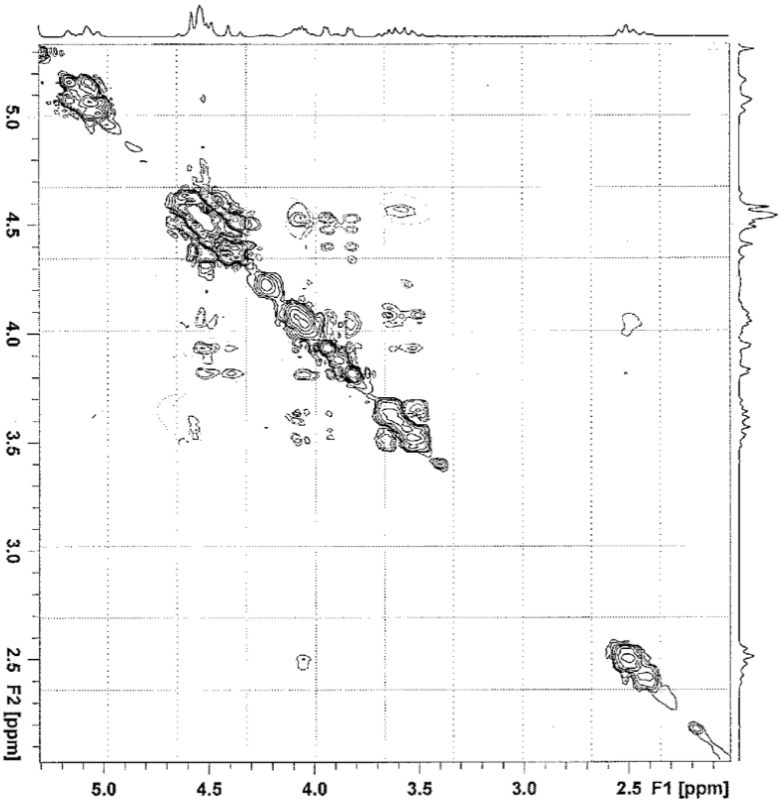

**NOESY NMR Spectra and correlations (Compound 35, 200 MHz, $CDCl_3$)**

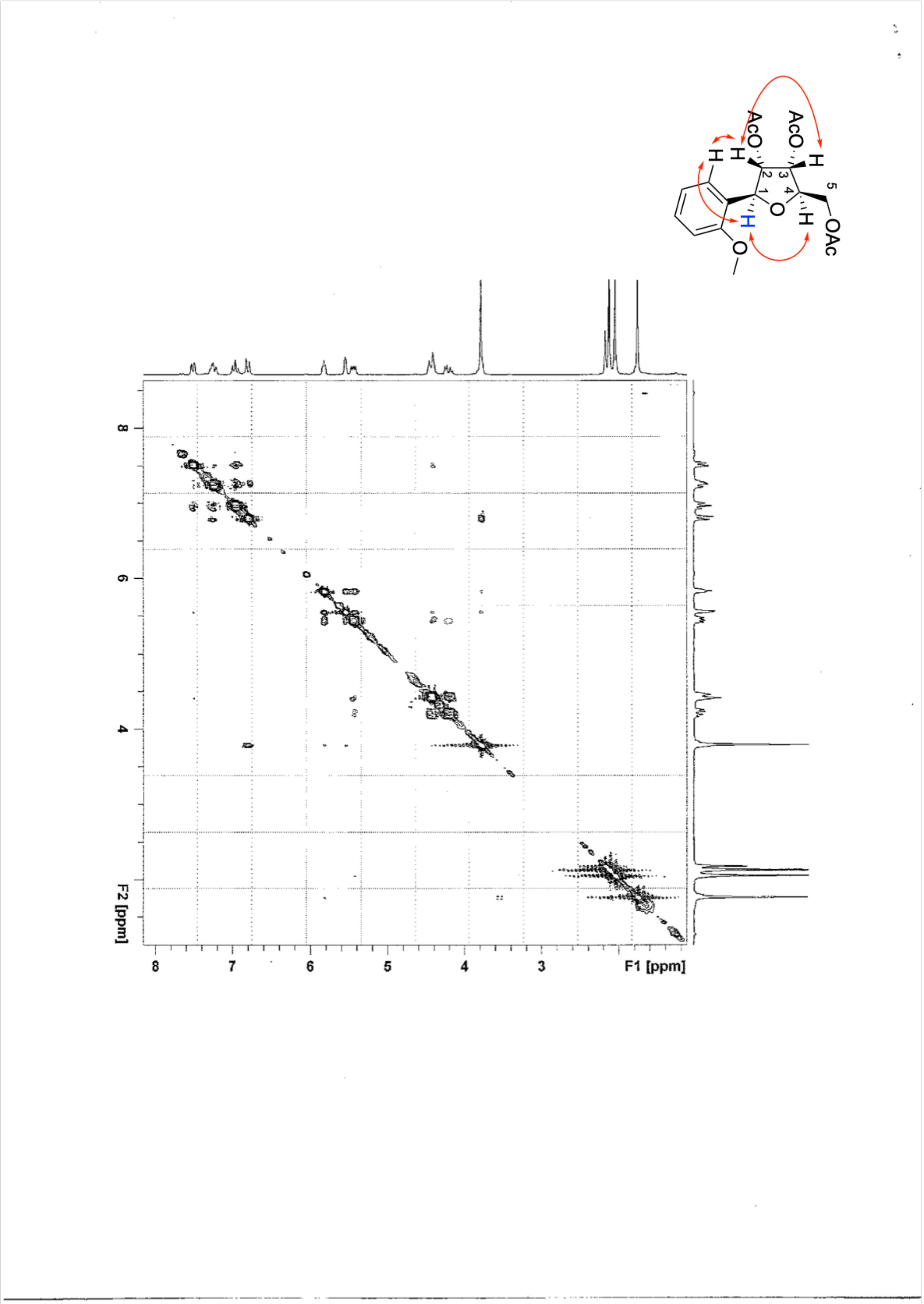

**NOESY NMR Spectra and correlations (Compound 41, 200 MHz, $CDCl_3$)**

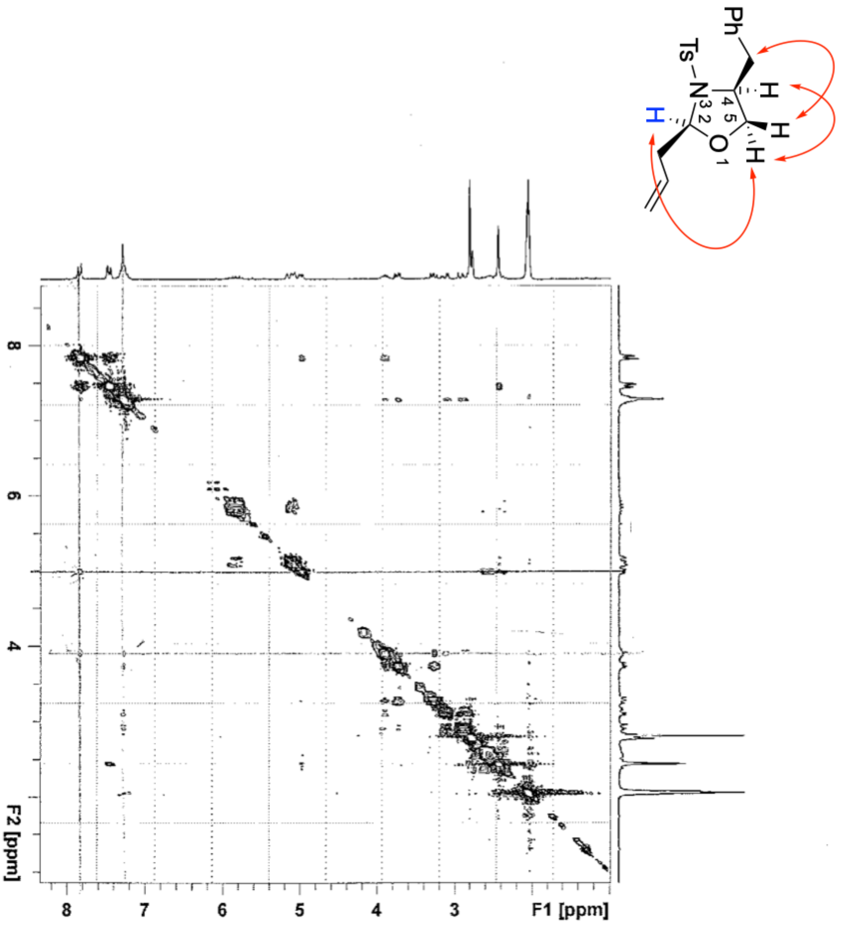

## Computational extra material

**Table S1.** Geometrical feature of the species involved in the β-approach mechnaism to the oxocabenium ion **5** with anisole as nucleophile [B3LYP/6-31G(d,p)&6-31+G(d,p)/DCM(PCM)//B3LYP/6-31G(d,p)&6-31+G(d,p)]

| Specie | $E_{rel}$ (kcal/mol) | $d_{O\text{-}C1}$ (Å) | $d_{C1\text{-}C(anisole)}$ (Å) | $d_{O\text{-}H}$ (Å) | $\rho_{C1}$ |
|---|---|---|---|---|---|
| **5 + anisole** | 0.00 | 1.26 | — | — | 0.538 |
| **Adduct-β** | -3.43 | 1.27 | 3.11 | 4.03 | 0.525 |
| **TS1-β** | 1.90 | 1.33 | 2.06 | 2.83 | 0.301 |
| **INT1-β** | -5.96 | 1.41 | 1.59 | 2.58 | 0.131 |
| **TS2-β** | 19.23 | 1.51 | 1.53 | 1.36 | 0.107 |
| **INT2-β** | -17.28 | 2.64 | 1.39 | 0.97 | 0.082 |

**Table S2.** Geometrical feature of the species involved in the α-approach mechnaism to the oxocabenium ion **5** with anisole as nucleophile [B3LYP/6-31G(d,p)&6-31+G(d,p)/DCM(PCM)//B3LYP/6-31G(d,p)&6-31+G(d,p)]

| Specie | $E_{rel}$ (kcal/mol) | $d_{O\text{-}C1}$ (Å) | $d_{C1\text{-}C(anisole)}$ (Å) | $d_{O\text{-}H}$ (Å) | $\rho_{C1}$ |
|---|---|---|---|---|---|
| **5 + anisole** | 0.00 | 1.26 | — | — | 0.538 |
| **Adduct-α** | -3.34 | 1.28 | 2.71 | 3.44 | 0.474 |
| **TS1-α** | -1.32 | 1.31 | 2.18 | 2.86 | 0.337 |
| **INT1-α** | -7.29 | 1.41 | 1.62 | 2.64 | 0.141 |
| **TS2-α** | 17.71 | 1.51 | 1.53 | 1.36 | 0.105 |
| **INT2-α** | -16.59 | 2.61 | 1.39 | 0.97 | 0.078 |

**Table S3.** Geometrical feature of the species involved in the equilibration step between **INT2-α** and **INT2-β** [B3LYP/6-31G(d,p)&6-31+G(d,p)/DCM(PCM)//B3LYP/6-31G(d,p)&6-31+G(d,p)]

| Specie | $E_{rel}$ (kcal/mol) | $d_{O\text{-}C1}$ (Å) | $d_{C1\text{-}C(anisole)}$ (Å) | $d_{O\text{-}H}$ (Å) | $\theta_{C(OTMS)\text{-}C\text{-}C1\text{-}C(anisole)}$ (°) | $\rho_{C1}$ |
|---|---|---|---|---|---|---|
| **INT2-α** | -16.59 | 2.61 | 1.39 | 0.97 | -92.7 | 0.078 |
| **TS3-α-β** | -13.88 | 3.05 | 1.39 | 0.97 | -168.5 | 0.093 |
| **INT2-β** | -17.28 | 2.64 | 1.39 | 0.97 | 138.8 | 0.082 |

**Chart S1.** Reaction mechanism and energetic pathway for the reaction of oxocabenium **5** with anisole [B3LYP/6-31G(d,p)&6-31+G(d,p)/DCM(PCM)//B3LYP/6-31G(d,p)&6-31+G(d,p)]

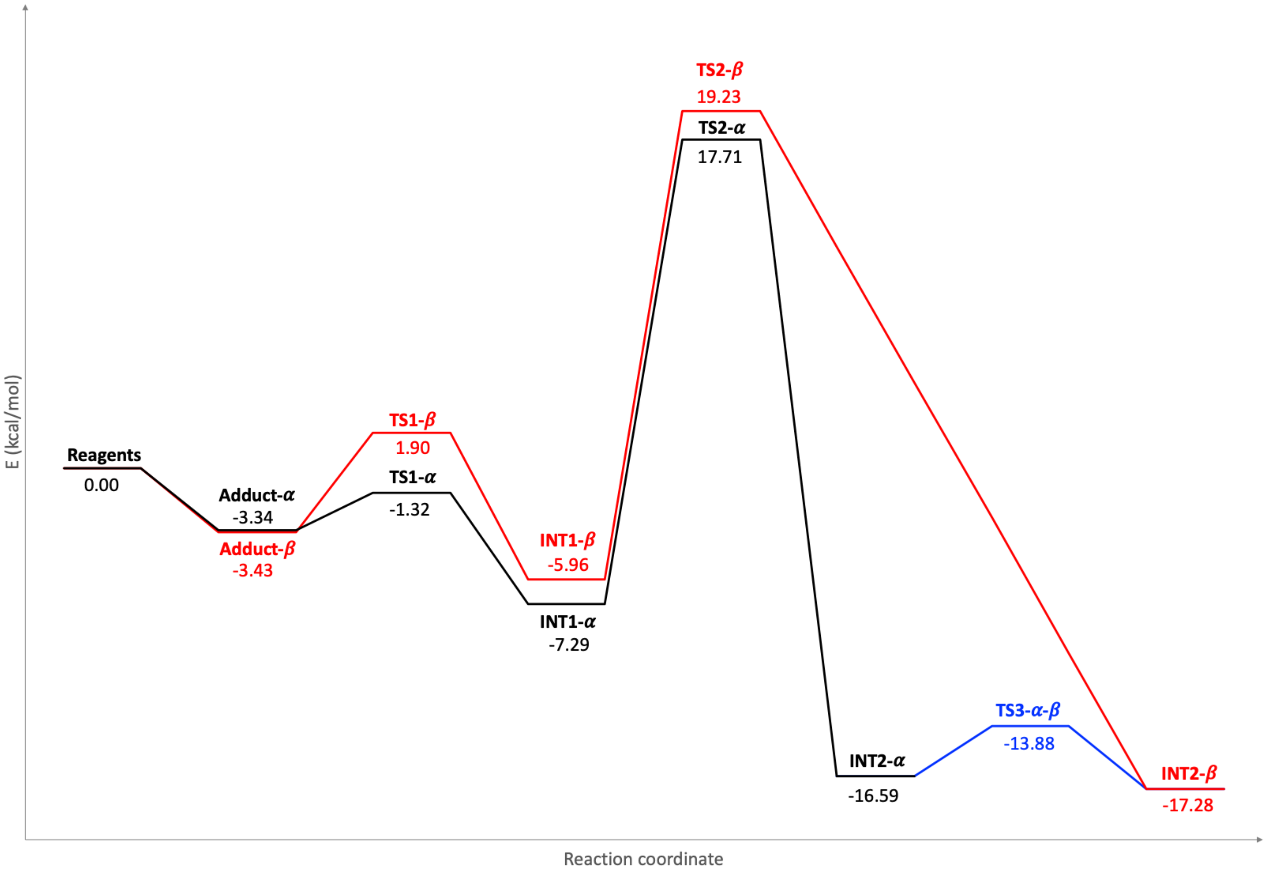


The final equilibration step between **INT2-α** and **INT2-β** is the key step for all the mechanism, because the ratio in energy of these two intermediates is preserved also in the final deprotonated species **26-TMS-β** and **26-TMS-α** (see Figure S1). By evaluating the Boltzmann population analysis for the activation energy of the forward (**INT2-α** to **INT2-β**) and the reverse (**INT2-β** to **INT2-α**) reactions we were able to calculate the observed diastereomeric ratio for the final products (see Table S4) which resulted totally in agreement with the experimental observed one.

**Table S4.** Boltzmann population analysis of the forward and reverse activation energy across **TS3-α-β** equilibration reaction step.

| Activation Energy | Energy (kcal/mol) | Population ratio | Calculated | Exerimental |
|---|---|---|---|---|
| **INT2-*α* to INT2-*β*** | 2.71 | 1.00 | **76.22** | **78** |
| **INT2-*β* to INT2-*α*** | 3.40 | 0.31 | **23.78** | **22** |
| | **Sum** | 1.31 | 100.00 | 100 |

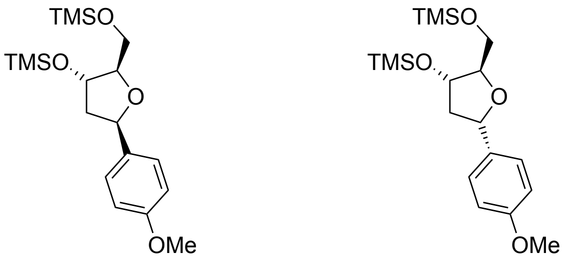


**26-TMS-*β***, 0.00 kcal/mol **26-TMS-*α***, 1.54 kcal/mol

**Figure S1.** Relative energy between products **26-TMS-β** and **26-TMS-α** [B3LYP/6-31G(d,p)&6-31+G(d,p)/DCM(PCM)//B3LYP/6-31G(d,p)&6-31+G(d,p)]

## Cartesian Coordinates, energies and frequencies

**5**

b3lyp/6-31g(d,p)&6-31+g(d,p),

el. energy = -1238.795360 a.u.

```
C 1.22149300 3.31983700 0.02650500
O 0.52997500 2.83817400 -0.91030700
C 0.17524300 1.38521600 -0.61646800
C 1.16414400 1.00791400 0.50699300
O 2.33541600 0.45887200 -0.00863600
Si 2.74187300 -1.22575600 -0.06235500
C 1.63013700 -2.06018600 -1.32724000
H 0.57040900 -1.97462100 -1.06150200
H 1.77177000 -1.63721900 -2.32810900
H 1.86535700 -3.12909100 -1.38929400
C 4.53856100 -1.20860100 -0.57615400
H 5.16265000 -0.70549200 0.16954400
H 4.67070300 -0.69390700 -1.53362300
H 4.91521100 -2.23083000 -0.69352800
C 2.47028300 -1.93747000 1.65642400
H 1.41922000 -1.91289100 1.96565000
H 3.06553600 -1.41302700 2.41177300
H 2.77894600 -2.98927700 1.67004700
C 1.51952100 2.38976200 1.12396600
H 0.89499700 2.66822300 1.98834100
H 2.56338500 2.45571600 1.45068900
H 0.65961300 0.36091100 1.23262100
C -1.31531200 1.35247500 -0.26419400
O -1.60897900 0.00650200 -0.02291500
Si -3.20711500 -0.64070000 0.06241800
C -4.07479200 -0.28702100 -1.56743500
H -3.51567000 -0.69401700 -2.41702700
H -5.06505000 -0.75730600 -1.57163500
H -4.23182700 0.78344700 -1.74119200
C -4.09327700 0.18562700 1.50045000
H -5.10502700 -0.22232500 1.60703300
H -4.19873700 1.26728900 1.35794900
H -3.56935700 0.01451200 2.44724900
C -2.91913400 -2.46713200 0.34616400
H -2.36167800 -2.91630800 -0.48232600
H -2.35963000 -2.64318200 1.27080500
H -3.87438200 -2.99729700 0.43011400
H -1.88843000 1.77523300 -1.10131800
H -1.50513600 1.98055200 0.62272300
H 0.38216000 0.86485300 -1.55115500
H 1.51586100 4.36553100 -0.07392600
```

**Anisole**

b3lyp/6-31g(d,p)&6-31+g(d,p),

el. energy = -346.780749 a.u.

```
C -2.28363000 0.33324700 -0.00006700
C -1.85440600 -0.99892700 0.00009300
C -0.49822500 -1.30493900 0.00001500
C 0.45127600 -0.27313400 -0.00025200
O 1.76324800 -0.67353300 -0.00043900
C 2.77295500 0.32610600 0.00099200
H 3.72341200 -0.20857400 0.00181800
H 2.71347600 0.95857400 -0.89361500
H 2.71150800 0.95794200 0.89590500
C 0.03370400 1.06230000 -0.00043900
C -1.33503000 1.35284000 -0.00032900
H -1.65271500 2.39189800 -0.00047100
H 0.75298000 1.87264600 -0.00070300

H -0.14896400 -2.33240700 0.00017000
H -2.58222400 -1.80549200 0.00031300
H -3.34332500 0.56872100 0.00001500
```

**Adduct-β**

b3lyp/6-31g(d,p)&6-31+g(d,p),

el. energy = -1585.593952 a.u.

```
C -1.57008500 0.20462100 -0.01308700
C -3.31880800 -2.11676800 1.10130500
C -3.83823700 -1.09914100 1.91980900
C -4.75612800 -0.18496700 1.41577800
C -5.19093900 -0.28517500 0.07870600
C -4.68177900 -1.30485700 -0.74558800
C -3.75756600 -2.21590500 -0.22312000
H -3.38702200 -3.01366900 -0.86009800
H -5.01438700 -1.40766300 -1.77132200
O -6.09675100 0.64127300 -0.31459000
C -6.65900700 0.55495000 -1.62819200
H -7.37909800 1.36948300 -1.69159300
H -5.88909200 0.68652700 -2.39694600
H -7.17218800 -0.40145800 -1.77299000
H -5.17896200 0.59673600 2.03826300
H -3.53805600 -1.03231400 2.96185700
C -0.80464700 0.78867100 1.10508300
C 0.60867800 0.98356800 0.49578300
O 0.69744800 2.29433900 0.02019000
Si 2.11155900 3.28369500 0.00400400
C 1.45460900 4.95816500 -0.50757900
H 0.73559100 5.34317700 0.22289000
H 0.95546300 4.90683400 -1.48086800
H 2.27118200 5.68421600 -0.58946300
C 2.84673000 3.28728800 1.73559400
H 2.12701400 3.64382100 2.48051200
H 3.20316400 2.29855600 2.04580900
H 3.71144500 3.96039000 1.76703700
C 3.33107700 2.57348600 -1.23907900
H 3.62819400 1.55104000 -0.97990500
H 4.24236100 3.18236200 -1.26459300
H 2.91489600 2.56585300 -2.25269900
C 0.61474400 -0.03324700 -0.66392500
C 1.19116700 -1.41821100 -0.35957700
O 2.55258000 -1.21817200 -0.09451600
Si 3.73424400 -2.46884300 -0.10298700
C 3.23849100 -3.75898000 1.17415700
H 2.28654300 -4.24309000 0.92731500
```

```
H 3.15032500 -3.32166800 2.17479100
H 3.99536000 -4.55003200 1.22696400
C 5.31647000 -1.57968200 0.35205400
H 5.55399700 -0.79500100 -0.37378300
H 6.15961200 -2.27914000 0.37381200
H 5.24135400 -1.11776800 1.34200500
C 3.79586800 -3.21779300 -1.82742800
H 2.85374800 -3.69989000 -2.11230600
H 4.57450600 -3.98797500 -1.87472000
H 4.03440200 -2.46223700 -2.58385600
H 1.01557800 -2.07252800 -1.22507300
H 0.66970400 -1.86102600 0.50502100
O -0.85401300 -0.21619500 -0.97056600
H 1.03587400 0.38405100 -1.57812400
H 1.39756100 0.72959600 1.21162200
H -0.83087300 0.08761900 1.95020000
H -1.24735500 1.72839500 1.45132800
H -2.64919700 0.14464400 -0.14097000
H -2.63080500 -2.85188300 1.50734400
```

**Adduct-α**

b3lyp/6-31g(d,p)&6-31+g(d,p),

el. energy = -1585.594923 a.u.

```
C -1.43217700 -0.69036200 0.58700500
H -2.41359000 -1.13911000 0.70890200
C -3.22552400 1.09408400 -0.38335600
C -3.92973300 1.10849700 0.83793100
C -4.91448900 0.16882000 1.09726400
C -5.24877900 -0.79225800 0.11825400
O -6.22597300 -1.65633800 0.46250300
C -6.68967100 -2.61559300 -0.49572300
H -7.48962000 -3.16090500 0.00265200
H -7.08321300 -2.11733100 -1.38756000
H -5.89021500 -3.31044500 -0.77500200
C -4.57660500 -0.79728100 -1.11820300
C -3.58240200 0.15039100 -1.35964700
H -3.07402300 0.14873100 -2.31935700
H -4.83324700 -1.51577400 -1.88683200
H -5.46644400 0.16511700 2.03123700
H -3.70363500 1.86413000 1.58509500
O -0.72729500 -1.09721600 -0.39839500
C 0.65558000 -0.50868600 -0.35087400
C 0.54365000 0.60446400 0.71099700
O 0.22930800 1.83982800 0.12982100
Si 1.35561300 3.10823600 -0.18055900
C 2.55960500 2.53424900 -1.50618200
H 3.10742700 1.63759800 -1.19572200
H 2.04466700 2.31409600 -2.44816500
H 3.29924900 3.31644100 -1.71317100
C 0.27090000 4.51830500 -0.76178500
H -0.44741900 4.81551700 0.00938600
H -0.28808800 4.24529800 -1.66312500
H 0.88104800 5.39556700 -1.00448200
C 2.25759200 3.50326200 1.42233200
H 2.87052200 2.66807600 1.77952200
H 1.56072600 3.78399600 2.21956800
H 2.93596300 4.35008100 1.26583700
C -0.66569200 0.13106900 1.55367500
H -0.36225900 -0.54227300 2.37171400
H -1.23670500 0.94839700 1.99276000
H 1.46168200 0.63637200 1.30687500
```

```
C 1.62168300 -1.65275100 -0.03137600
O 2.90486600 -1.08701400 -0.01555200
Si 4.36377000 -1.99604700 -0.00168300
C 4.42125800 -3.06069000 -1.55121800
H 4.34681500 -2.45193300 -2.45888600
H 5.37150300 -3.60530400 -1.59719000
H 3.62263900 -3.81071700 -1.57651800
C 4.38594000 -3.06279300 1.54833700
H 5.32495600 -3.62509700 1.60790500
H 3.57229600 -3.79719100 1.55890000
H 4.30955000 -2.45505600 2.45672300
C 5.69767200 -0.68259500 0.01564600
H 5.64231300 -0.04834300 -0.87522100
H 5.61049000 -0.04042700 0.89832800
H 6.69198500 -1.14253800 0.03557000
H 1.51099200 -2.43331000 -0.79725200
H 1.36244900 -2.10134400 0.94175400
H 0.82973700 -0.11052700 -1.35036300
H -2.48001000 1.85267500 -0.59795700
```

**TS1-β**

b3lyp/6-31g(d,p)&6-31+g(d,p),

el. energy = -1585.586486 a.u.

im. frequency = -213.89

```
C -1.57205600 0.41463400 0.00262300
C -2.69537400 -1.16502900 0.69341800
C -3.47824900 -0.52033300 1.70702900
C -4.74927500 -0.07901200 1.44080700
C -5.34119800 -0.35067700 0.17767600
C -4.63386600 -1.09255400 -0.80192400
C -3.35503800 -1.52314500 -0.52326700
H -2.80982700 -2.09076700 -1.27064300
H -5.09356100 -1.33155700 -1.75258500
O -6.56962300 0.12699400 0.01270800
C -7.29416800 -0.10715100 -1.21271800
H -8.25206200 0.39012500 -1.07468700
H -6.76344400 0.33364500 -2.06111700
H -7.44854500 -1.17854400 -1.36634000
H -5.34000000 0.45362600 2.17797500
H -3.04207900 -0.33564600 2.68399200
C -0.70544500 0.89468400 1.15083800
C 0.68863500 1.03094900 0.49061900
O 0.82172100 2.33408000 -0.01447000
Si 2.27871900 3.23190000 -0.12231400
C 1.70704000 4.92435800 -0.68275200
H 1.03048400 5.37660100 0.04990100
H 1.17829900 4.86373200 -1.63985200
H 2.56020600 5.59928700 -0.81413000
C 3.08540800 3.27271200 1.57870100
H 2.41802100 3.70893400 2.33001100
H 3.38612100 2.27707100 1.92403500
H 3.99252300 3.88736900 1.54699200
C 3.42294300 2.41720300 -1.37536100
H 3.68136200 1.39256400 -1.08529900
H 4.35962700 2.98091400 -1.45775200
H 2.96899700 2.38787800 -2.37226900
C 0.61168500 -0.00391100 -0.64657400
C 1.08832800 -1.41564700 -0.29881100
O 2.46748800 -1.32931800 -0.04313400
Si 3.53707600 -2.66422600 0.00415300
```

```
C 2.97261600 -3.84138800 1.36241200
H 1.97868300 -4.25831000 1.16232100
H 2.94293700 -3.34191800 2.33718200
H 3.66439300 -4.68735900 1.44721200
C 5.20015500 -1.89244900 0.38348900
H 5.49161800 -1.17947400 -0.39480400
H 5.98022700 -2.65963800 0.44272900
H 5.18054200 -1.36130000 1.34088700
C 3.51272700 -3.52225800 -1.67131200
H 2.52724200 -3.93308200 -1.91876000
H 4.21941600 -4.36023200 -1.67686300
H 3.80636200 -2.83762000 -2.47448700
H 0.85833600 -2.08140300 -1.14311100
H 0.54683500 -1.79748600 0.58276600
O -0.82192900 -0.02654500 -1.00052300
H 1.12015800 0.35276700 -1.54276100
H 1.48937200 0.76757100 1.19024400
H -0.67483900 0.17299300 1.97059300
H -1.05572900 1.85095900 1.54545600
H -2.45689800 0.95778000 -0.31825500
H -1.83637600 -1.75452100 0.99966000
```

**TS1-α**

b3lyp/6-31g(d,p)&6-31+g(d,p),

el. energy  = -1585.593900 a.u.

im. frequency = -129.96

```
C -1.44189000 -0.79168600 0.70959500
H -2.25726000 -1.44066700 1.01272500
C -2.83818900 0.56021500 -0.26728400
C -3.69480900 0.91254800 0.81784100
C -4.90144000 0.28003500 1.00493400
C -5.34339000 -0.68621100 0.06562900
O -6.52469000 -1.24558300 0.33396000
C -7.09917300 -2.20400100 -0.57372300
H -8.05319400 -2.48150400 -0.12960500
H -7.26368100 -1.75243200 -1.55619500
H -6.45837100 -3.08657300 -0.65911500
C -4.55136600 -0.99616800 -1.06450200
C -3.33765200 -0.35677100 -1.23260500
H -2.72985900 -0.59502200 -2.10013900
H -4.89196300 -1.71592000 -1.79822800
H -5.55149700 0.51715200 1.84007000
H -3.38090200 1.67961500 1.51935600
O -0.66430400 -1.28434800 -0.22899200
C 0.65330900 -0.60249200 -0.25396200
C 0.51799400 0.55330300 0.75805900
O 0.14969200 1.75702200 0.12263400
Si 1.22611000 3.05020700 -0.23854100
C 2.43036900 2.49021700 -1.56956000
H 3.00434600 1.61408500 -1.24883700
H 1.90983800 2.23723800 -2.50014700
H 3.14504400 3.28900200 -1.79973500
C 0.08631500 4.41048500 -0.83880100
H -0.62539400 4.70918900 -0.06194400
H -0.48188200 4.09496000 -1.72042800
H 0.66474300 5.29791800 -1.11931200
C 2.13897500 3.52620000 1.33654100
H 2.79120400 2.72565900 1.70287500
H 1.44528500 3.79877100 2.13946000
H 2.77895500 4.39551800 1.14557000
```

```
C -0.64802200 0.07225800 1.64744900
H -0.28656100 -0.58866800 2.44893700
H -1.19746300 0.89085000 2.10784000
H 1.44455900 0.65630700 1.33140800
C 1.71398000 -1.65341100 0.08204800
O 2.96166600 -1.00989800 0.00542100
Si 4.46596700 -1.82991200 -0.02834000
C 4.53749500 -2.89871800 -1.57564500
H 4.40506700 -2.29843600 -2.48239400
H 5.51242300 -3.39471400 -1.64748600
H 3.77643300 -3.68723100 -1.57531600
C 4.61071300 -2.88923200 1.52145900
H 5.58221700 -3.39622800 1.54629200
H 3.84096400 -3.66836100 1.56320500
H 4.53266200 -2.28389400 2.43135800
C 5.72702500 -0.44538400 -0.06242300
H 5.59139100 0.19273300 -0.94193800
H 5.64865800 0.18356600 0.83055800
H 6.74500100 -0.84925100 -0.09763600
H 1.62266000 -2.48321300 -0.63278800
H 1.53201100 -2.06108600 1.08932500
H 0.79114000 -0.23082900 -1.27029700
H -2.01604600 1.22265400 -0.52560200
```

**INT1-β**

b3lyp/6-31g(d,p)&6-31+g(d,p),

el. energy  = -1585.594222 a.u.

```
C -1.56833000 0.09005700 0.22918000
C -2.76187700 -0.87822500 0.63658900
C -3.68001700 -0.23923700 1.60852100
C -4.99909200 -0.06295700 1.35508900
C -5.55305300 -0.53406500 0.11798400
C -4.74371000 -1.21013500 -0.84708700
C -3.41907800 -1.38532800 -0.58833200
H -2.78046000 -1.88325500 -1.31125300
H -5.18079500 -1.57526200 -1.76811900
O -6.83031700 -0.30001600 -0.03573700
C -7.55471300 -0.71275200 -1.22740100
H -8.57525900 -0.37724700 -1.05885100
H -7.13292100 -0.22192200 -2.10683100
H -7.52281800 -1.79994200 -1.32344300
H -5.67096500 0.41814000 2.05758200
H -3.26116700 0.10905200 2.54831400
C -0.64966500 0.51679800 1.37417100
C 0.64912900 0.82806100 0.60626000
O 0.54163900 2.12921600 0.06504600
Si 1.83201200 3.20955500 -0.21938200
C 0.97033200 4.80967100 -0.68224600
H 0.34042600 5.17050500 0.13776400
H 0.33435200 4.67314200 -1.56347500
H 1.70060400 5.59279500 -0.91517500
C 2.84176500 3.38688000 1.36132200
H 2.21843200 3.71215400 2.20159000
H 3.33772500 2.45206200 1.64488100
H 3.62785800 4.13821600 1.22239500
C 2.91269200 2.56901900 -1.62106000
H 3.35008300 1.59380700 -1.38097300
H 3.73828400 3.26422500 -1.81425800
H 2.34171800 2.46810800 -2.55086000
C 0.64459400 -0.24913000 -0.49943500
C 1.45036300 -1.50468800 -0.16417700
```

O 2.81347700 -1.14430500 -0.10491800
Si 4.10139200 -2.25393800 -0.00918700
C 3.90473900 -3.29372400 1.55129800
H 2.99262500 -3.90134600 1.53060000
H 3.87368100 -2.66484700 2.44807800
H 4.74947900 -3.98360300 1.66103800
C 5.63229000 -1.17517800 0.05988500
H 5.70803300 -0.54179800 -0.83026100
H 6.53943300 -1.78754800 0.11308600
H 5.61667000 -0.52293200 0.93945900
C 4.08453000 -3.35589200 -1.53726900
H 3.17482800 -3.96374900 -1.60130000
H 4.93437300 -4.04809400 -1.51888900
H 4.16020900 -2.76229600 -2.45494700
H 1.25661800 -2.25923900 -0.93996000
H 1.10742200 -1.92159500 0.79686900
O -0.74989900 -0.63188700 -0.67686300
H 1.00151400 0.17669800 -1.44050800
H 1.53375900 0.72105900 1.24378800
H -0.49161600 -0.31057800 2.07597400
H -1.00193900 1.39398900 1.91963400
H -2.00699900 0.96431500 -0.26758700
H -2.24545700 -1.72077300 1.12562600

**INT1-α**

b3lyp/6-31g(d,p)&6-31+g(d,p),

el. energy = -1585.600545 a.u.

C -1.27590900 -1.07100400 0.88005500
H -1.69212600 -1.96008200 1.36024100
C -2.52429600 -0.22932100 0.27499400
C -3.50131300 0.19146400 1.29406000
C -4.83076900 -0.06353600 1.18274500
C -5.32515800 -0.75376400 0.03124500
O -6.62141400 -0.95837000 0.02145800
C -7.28061100 -1.63653700 -1.07944800
H -8.33401600 -1.64442200 -0.80906000
H -7.13169800 -1.07815700 -2.00621500
H -6.90485400 -2.65825400 -1.16795100
C -4.44215100 -1.17564900 -1.00701400
C -3.10732600 -0.92754900 -0.87946900
H -2.41562600 -1.27193200 -1.64208100
H -4.82601900 -1.69766100 -1.87471400
H -5.54900800 0.24677100 1.93384100
H -3.13574700 0.73290800 2.16137400
O -0.45107500 -1.48131900 -0.18525600
C 0.76098800 -0.67380200 -0.25252800
C 0.57052200 0.43962700 0.78998200
O -0.08286600 1.57284300 0.21993000
Si 0.71714600 3.00719900 -0.27578300
C 1.84706500 2.63054300 -1.73066200
H 2.60641200 1.88559900 -1.46868500
H 1.28309700 2.25370300 -2.59129800
H 2.37050300 3.53891400 -2.05142700
C -0.69530000 4.14553800 -0.75636400
H -1.36340100 4.33014900 0.09171600
H -1.28794600 3.72277400 -1.57511900
H -0.31155200 5.11467600 -1.09419000
C 1.70416300 3.68307800 1.17720000
H 2.51430700 3.00947500 1.47720300
H 1.06746200 3.86238300 2.05043000
H 2.16700600 4.63828200 0.90313200
C -0.38495500 -0.22412400 1.79378500

H 0.16530200 -0.89092300 2.46462900
H -0.90953900 0.51313900 2.40339500
H 1.52619400 0.71595200 1.24461100
C 1.96363000 -1.58363400 0.00951100
O 3.13304900 -0.81023100 -0.15732400
Si 4.71626500 -1.44085500 -0.11764200
C 4.89522300 -2.75439600 -1.45523600
H 4.65757200 -2.35033500 -2.44544100
H 5.92615800 -3.12559400 -1.48762400
H 4.24670300 -3.61959800 -1.27716000
C 5.03950100 -2.18433700 1.58352300
H 6.07098700 -2.54881800 1.65261100
H 4.38190500 -3.03562500 1.79366400
H 4.89826900 -1.44195100 2.37694800
C 5.80488000 0.04947000 -0.44709900
H 5.57467200 0.49726300 -1.41962200
H 5.66761400 0.81749200 0.32153600
H 6.86360800 -0.23295500 -0.45115800
H 1.92028800 -2.42424300 -0.69673800
H 1.90057600 -2.00193100 1.02629300
H 0.83577200 -0.25351700 -1.25860700
H -1.98126500 0.67191600 -0.08271700

**TS2-β**

b3lyp/6-31g(d,p)&6-31+g(d,p),

el. energy = -1585.558984 a.u.

im. frequency = -1436.04

C 1.36194100 -1.29771300 0.07328600
C 2.73269300 -0.61292100 0.10927900
C 3.22829900 0.12002400 1.24379700
C 4.52147100 0.57221000 1.28243000
C 5.41941500 0.26369900 0.22657600
C 4.98733400 -0.51392800 -0.87335800
C 3.68644700 -0.96720000 -0.90493700
H 3.35369700 -1.57660600 -1.74097200
H 5.66795700 -0.76819100 -1.67588500
O 6.64790900 0.74734200 0.36772200
C 7.66322000 0.47904700 -0.62183400
H 8.55526700 0.98349300 -0.25627000
H 7.84706500 -0.59600400 -0.69825600
H 7.37118400 0.89296400 -1.59090600
H 4.89619500 1.15179700 2.11882600
H 2.56715000 0.34309000 2.07388500
C 0.38938300 -1.17272900 1.23389600
C -0.96857000 -1.03553400 0.51201300
O -1.36113000 -2.30529100 0.04723800
Si -2.97061700 -2.88818200 -0.02213200
C -2.76889900 -4.68102200 -0.52425900
H -2.19114800 -5.23795500 0.22084900
H -2.25314700 -4.76608500 -1.48661100
H -3.74541300 -5.16799300 -0.62428900
C -3.75119100 -2.70948000 1.68287700
H -3.17968400 -3.25087200 2.44473300
H -3.83714800 -1.66357600 1.99871900
H -4.76580800 -3.12458100 1.67490200
C -3.93513700 -1.89562200 -1.29763900
H -3.96826800 -0.83025000 -1.04334800
H -4.97029800 -2.25175400 -1.35623400
H -3.49587500 -1.99522800 -2.29656900
C -0.64494800 -0.09294500 -0.66630100

C -0.79563800 1.40071200 -0.37147900
O -2.16344500 1.64454900 -0.16414900
Si -2.87304400 3.19433800 -0.03141600
C -2.10924800 4.08183000 1.44477500
H -1.03379300 4.25019600 1.31492700
H -2.25628100 3.51528800 2.37101000
H -2.57379000 5.06498100 1.58272200
C -4.69258600 2.83407300 0.22372900
H -5.10409900 2.27001500 -0.61980700
H -5.26431100 3.76434000 0.31490300
H -4.85698500 2.25011300 1.13538300
C -2.55180600 4.15177000 -1.62015800
H -1.48627900 4.35056100 -1.78172600
H -3.05737400 5.12383200 -1.58530000
H -2.93351500 3.61247900 -2.49397200
H -0.39672300 1.96872500 -1.22531900
H -0.20062300 1.67315800 0.51632600
O 0.76724700 -0.39305700 -0.97772600
H -1.22339100 -0.36097900 -1.55189800
H -1.72255800 -0.57569900 1.16082800
H 0.58086000 -0.27746400 1.83254100
H 0.40500000 -2.04617800 1.88737800
H 1.38891500 -2.30907400 -0.33502600
H 1.93674600 0.20329200 -0.63671800

**TS2-α**

b3lyp/6-31g(d,p)&6-31+g(d,p),

el. energy = -1585.563608 a.u.

im. frequency = -1436.80

C -1.10980400 -1.38751700 1.09694400
H -1.09454500 -2.38117900 1.54685500
C -2.49238800 -1.04937100 0.52895700
C -3.04282100 0.28178100 0.52965900
C -4.34033800 0.50200800 0.14182600
C -5.18258300 -0.58647000 -0.19822300
O -6.42148800 -0.26015800 -0.55212100
C -7.37788800 -1.27944300 -0.90674800
H -8.29098300 -0.73958500 -1.14927400
H -7.03349300 -1.84032400 -1.78010700
H -7.55489200 -1.94966500 -0.06107500
C -4.69164700 -1.91394600 -0.14884800
C -3.38954400 -2.13325000 0.24084600
H -3.01557900 -3.15231900 0.29655600
H -5.33076200 -2.75200100 -0.39612600
H -4.75631600 1.50260700 0.09945600
H -2.39454600 1.11605000 0.77493400
O -0.40482700 -1.49161700 -0.24032300
C 0.79490700 -0.63889600 -0.25758500
C 0.54869100 0.40664500 0.84199500
O -0.18970600 1.50658200 0.33972100
Si 0.50999100 3.00527100 -0.12546700
C 1.64204800 2.73385900 -1.60322800
H 2.45337300 2.03639400 -1.36746900
H 1.09305400 2.34243800 -2.46711600
H 2.10183900 3.68109600 -1.90834900
C -0.97480700 4.06511000 -0.55798200
H -1.63441100 4.20209400 0.30574100
H -1.55898200 3.62175000 -1.37158000
H -0.65156200 5.05895800 -0.88718700
C 1.47587900 3.69943100 1.33262400
H 2.32811800 3.06836300 1.60778500
H 0.84141700 3.82171700 2.21724300
H 1.87920100 4.68615900 1.07661700
C -0.29801000 -0.35774400 1.88526100
H 0.35609900 -0.88405900 2.58554700
H -0.91892600 0.32783500 2.46340200
H 1.50294400 0.72539400 1.27370600
C 2.02382400 -1.52879800 -0.04750300
O 3.15545400 -0.69565400 -0.13587600
Si 4.76582000 -1.25849900 -0.26275100
C 4.90674900 -2.36863500 -1.77688800
H 4.61395400 -1.83874900 -2.68997200
H 5.94318700 -2.70133100 -1.90629200
H 4.28913700 -3.26992100 -1.69332200
C 5.20249900 -2.20793800 1.30327900
H 6.24455700 -2.54598400 1.26765600
H 4.57881600 -3.09975800 1.43399100
H 5.08970800 -1.58045300 2.19430300
C 5.77416800 0.31076500 -0.44055200
H 5.48679300 0.86499200 -1.34021000
H 5.63731400 0.96948600 0.42354600
H 6.84244200 0.07956200 -0.51785600
H 2.01896000 -2.31111600 -0.81882700
H 1.96851900 -2.02830400 0.93299200
H 0.83540300 -0.17529400 -1.24395000
H -1.66025500 -1.06842800 -0.54078500

**INT2-β**

b3lyp/6-31g(d,p)&6-31+g(d,p),

el. energy = -1585.617549 a.u.

C 1.13509800 -1.33285300 0.31829200
C 2.44142700 -0.85009800 0.31331100
C 2.83536000 0.38386100 0.93135500
C 4.13671600 0.80303600 0.89393500
C 5.12016000 0.01582500 0.23149600
C 4.76395200 -1.20806300 -0.38910800
C 3.45378000 -1.62118100 -0.34198700
H 3.17057100 -2.55633800 -0.81624700
H 5.50839000 -1.81034500 -0.89387800
O 6.34746500 0.51521400 0.24963800
C 7.44069100 -0.17747200 -0.39094800
H 8.31120900 0.45326700 -0.22347800
H 7.59340800 -1.15654300 0.07074000
H 7.25104800 -0.27734500 -1.46287700
H 4.45425700 1.73065200 1.35685400
H 2.09659100 0.99375300 1.43876600
C -0.04948800 -0.75914700 0.98478700
C -1.32613100 -0.82891400 0.09826500
O -1.55882800 -2.17906900 -0.26213300
Si -2.96451600 -3.06390300 0.17080300
C -2.66553600 -4.76488900 -0.55441700
H -1.76571600 -5.22680800 -0.13429500
H -2.54926200 -4.72016800 -1.64252400
H -3.51082900 -5.42797500 -0.33868700
C -3.09093700 -3.10524000 2.04975200
H -2.21668800 -3.58587500 2.50295200
H -3.19412900 -2.10315700 2.48169800
H -3.97407900 -3.67749500 2.35658000
C -4.47100100 -2.22224800 -0.57743300
H -4.59845500 -1.19738400 -0.21087400
H -5.38215400 -2.77375700 -0.31795500
H -4.40771600 -2.18638000 -1.67051400

C -1.13855400 -0.02237400 -1.19754600
C -0.84349000 1.46404100 -0.99152900
O -1.78695300 1.99322000 -0.08308400
Si -2.13366100 3.65400200 0.08987300
C -0.57574100 4.54221400 0.67426300
H 0.23992400 4.46086800 -0.05356600
H -0.22311700 4.14027900 1.63096100
H -0.77276900 5.61051500 0.82010900
C -3.48803100 3.70186500 1.38420500
H -4.37002000 3.14448600 1.05161800
H -3.80018000 4.73293700 1.58428700
H -3.14862700 3.26651600 2.33019400
C -2.70643200 4.34674700 -1.56369300
H -1.92745100 4.29461600 -2.33246500
H -2.98273200 5.40209200 -1.45625200
H -3.58641400 3.81091800 -1.93594900
H -0.88239900 1.95941700 -1.97062800
H 0.18288700 1.58149500 -0.61174800
O -0.03407400 -0.56832500 -1.92333400
H -2.06274200 -0.13011100 -1.78019100
H -2.15337400 -0.39814700 0.67208400
H 0.10386800 0.25437000 1.35912800
H -0.25996800 -1.40632100 1.85083000
H 0.96493100 -2.30202700 -0.14498200
H -0.30571400 -1.44038300 -2.24583700

**INT2-α**

b3lyp/6-31g(d,p)&6-31+g(d,p),

el. energy = -1585.616754 a.u.

C 0.93644700 -1.42883000 -0.53454500
H 0.55400100 -2.36368400 -0.13447400
C 2.31396300 -1.24629000 -0.44587200
C 2.99192600 -0.08187000 -0.94089600
C 4.34931300 0.04182000 -0.82004000
C 5.10734100 -0.99250500 -0.20440900
O 6.41394800 -0.77769700 -0.14746100
C 7.30224500 -1.75338300 0.43920500
H 8.29731900 -1.32471600 0.34038500
H 7.06133400 -1.90068200 1.49528100
H 7.24500100 -2.69756500 -0.10867900
C 4.46721000 -2.15623100 0.29497700
C 3.10349200 -2.26954700 0.17216600
H 2.60264300 -3.15350500 0.55633900
H 5.04038900 -2.94459600 0.76596200
H 4.88158800 0.91103200 -1.18994100
H 2.41877300 0.70998100 -1.40666500
O -0.18505900 -0.83741800 1.75138000
C -1.25155500 -0.17610200 1.06891300
C -0.69533700 0.50972900 -0.18607900
O 0.29512800 1.43711300 0.21073000
Si 0.11280900 3.14237600 0.09735400
C -1.39943100 3.67404800 1.08038400
H -2.32200800 3.22324300 0.69779200
H -1.30540300 3.41077800 2.13958700
H -1.52294200 4.76177000 1.02177100
C 1.70332600 3.80807700 0.83172700
H 2.58102700 3.45017800 0.28321100
H 1.81299500 3.50926400 1.87963400
H 1.71363300 4.90315400 0.79609100
C -0.08062600 3.60034300 -1.71863800
H -0.97356300 3.14974900 -2.16652300
H 0.78879000 3.29283000 -2.31043500
H -0.18175500 4.68646300 -1.82629300
C -0.05001400 -0.53096200 -1.17599200
H -0.86832700 -1.12775200 -1.58991300
H 0.39997800 0.04927200 -1.98423400
H -1.51639100 0.98542600 -0.73340800
C -2.33956800 -1.21730500 0.80102600
O -3.31419200 -0.63714500 -0.03941200
Si -4.91616100 -1.20717300 -0.19550800
C -5.76719500 -1.11033600 1.48087900
H -5.76405000 -0.08671400 1.87107400
H -6.81267400 -1.42885300 1.39565400
H -5.29371400 -1.75897800 2.22640800
C -4.87617700 -2.98628400 -0.81388200
H -5.89459100 -3.36369800 -0.96160200
H -4.38403300 -3.65730300 -0.10042100
H -4.35321200 -3.06669400 -1.77345900
C -5.68513200 -0.04337600 -1.44719100
H -5.65959700 0.99174600 -1.09051500
H -5.15557900 -0.08603700 -2.40494500
H -6.73239000 -0.30669600 -1.63271600
H -2.75268600 -1.53613600 1.76720900
H -1.89451400 -2.10846600 0.33130200
H -1.67086800 0.61518100 1.70471600
H 0.44777800 -0.15564400 2.02364900

**TS3-α-β**

b3lyp/6-31g(d,p)&6-31+g(d,p),

el. energy = -1585.610512 a.u.

im. frequency = -39.38

C -1.23657500 0.18655000 -0.45595200
H -1.03489400 0.46401800 -1.48911100
C -2.52975700 -0.20086300 -0.14524000
C -2.94427400 -0.60613400 1.17236100
C -4.23174000 -0.99090200 1.41228000
C -5.18547500 -0.98996800 0.35047600
O -6.40159300 -1.37575100 0.69163300
C -7.47434300 -1.41921500 -0.27804000
H -8.34271300 -1.76315400 0.27957800
H -7.65812800 -0.42083600 -0.68265500
H -7.23455900 -2.12737900 -1.07510000
C -4.81066700 -0.59380300 -0.96051400
C -3.51311000 -0.21089100 -1.19175800
H -3.21084700 0.09163100 -2.19016500
H -5.53329000 -0.59406000 -1.76649500
H -4.56729000 -1.30007600 2.39590900
H -2.22484400 -0.60611600 1.98399600
O 0.98228000 0.53148000 -2.51699900
C 1.79335200 0.31739000 -1.36051800
C 1.16016500 0.96367500 -0.10589200
O 0.78460000 2.29198000 -0.45980600
Si 1.28515400 3.70271200 0.36775600
C 3.16351800 3.80417500 0.34664900
H 3.63087100 2.95287900 0.85441200
H 3.55130800 3.84222000 -0.67709500
H 3.50036300 4.71149700 0.86144500
C 0.48561900 5.09697900 -0.59526700
H -0.60508800 4.99791100 -0.60717100
H 0.83575600 5.11436000 -1.63283000
H 0.72785800 6.06684500 -0.14661700

C 0.65396000 3.62040000 2.14297600
H 1.05951900 2.75692700 2.68359400
H -0.43998000 3.56617200 2.17891800
H 0.95603400 4.51553400 2.69874400
C -0.06883800 0.23164000 0.46063800
H 0.21923300 -0.80019900 0.72253800
H -0.35604400 0.70046300 1.41069100
H 1.92464400 0.95177100 0.67921100
C 1.99645600 -1.19507300 -1.25412600
O 2.55924300 -1.50390700 0.00844300
Si 3.50037400 -2.89096600 0.32451900
C 4.97704100 -2.92266300 -0.84212000
H 5.58055800 -2.01351600 -0.74606400
H 5.62431100 -3.77595300 -0.60825800
H 4.67968600 -3.02328800 -1.89177700
C 2.43172000 -4.42466800 0.08015800
H 3.00347600 -5.33384500 0.29900300
H 2.06989500 -4.50970400 -0.95100000
H 1.56118200 -4.41460100 0.74580200
C 4.02146900 -2.68452600 2.11390700
H 4.60293500 -1.76677700 2.25137900
H 3.15193400 -2.63817900 2.77846600
H 4.64367900 -3.52619400 2.43798100
H 2.63510800 -1.51833600 -2.08542000
H 1.02474400 -1.69766700 -1.38120800
H 2.77038500 0.80077900 -1.49942300
H 0.92944300 1.48940300 -2.64939600

**26-TMS-β**

b3lyp/6-31g(d,p)&6-31+g(d,p),

el. energy = -1585.248003 a.u.

C -1.54769800 0.93817800 -0.42564500
C -2.99700600 0.53586700 -0.29603600
C -3.99799700 1.51885000 -0.29489400
C -5.33433100 1.18593000 -0.11249500
C -5.70338500 -0.15464900 0.06228400
C -4.72088200 -1.15040300 0.05053100
C -3.38022400 -0.79599900 -0.12463400
H -2.62165100 -1.57147000 -0.14618600
H -4.98276400 -2.19502000 0.17092900
O -7.04477400 -0.38843000 0.22936800
C -7.47669800 -1.73081600 0.40223500
H -8.56033300 -1.68447200 0.51628600
H -7.22984700 -2.34780900 -0.47082700
H -7.03655500 -2.18199500 1.30044200
H -6.10983200 1.94495600 -0.11185400
H -3.72606200 2.56156200 -0.44154000
C -0.79093900 1.12383800 0.90007200
C 0.67311500 1.00494200 0.46290700
O 1.13359800 2.27967200 0.02559000
Si 2.72259500 2.84541400 0.07178400
C 2.56872600 4.67552600 -0.32789600
H 1.96657600 5.19267900 0.42678100
H 2.08357300 4.81897700 -1.29945500
H 3.55285700 5.15668100 -0.36747100
C 3.44473000 2.58118300 1.79715900
H 2.82836600 3.06397900 2.56375600
H 3.52588800 1.51741700 2.04699100
H 4.45232800 3.00928800 1.85854600
C 3.79095800 1.96245000 -1.20674600
H 3.80700000 0.88257000 -1.02491700
H 4.82403800 2.32932700 -1.17207000
H 3.40975600 2.12952200 -2.22051700
C 0.58863900 -0.00489000 -0.71105100
C 1.04551700 -1.41792900 -0.35191500
O 2.44961600 -1.41046900 -0.12158400
Si 3.38075400 -2.80487200 0.07353700
C 2.76328600 -3.78786100 1.56230400
H 1.73274500 -4.13361800 1.42244200
H 2.79394900 -3.18378700 2.47605200
H 3.38621200 -4.67491200 1.72753400
C 5.12716900 -2.17308800 0.35467300
H 5.47198500 -1.58194700 -0.50033000
H 5.82928400 -3.00265000 0.49678000
H 5.17350500 -1.53599400 1.24432700
C 3.27694400 -3.86774500 -1.48212500
H 2.25611400 -4.21919600 -1.66983800
H 3.91626600 -4.75370300 -1.38967600
H 3.60606600 -3.30760100 -2.36433500
H 0.77877100 -2.08843200 -1.17979300
H 0.50759700 -1.75998700 0.54491900
O -0.79251600 -0.08790600 -1.09601000
H 1.18563600 0.36587700 -1.55272800
H 1.31535000 0.61791800 1.26271600
H -1.05651000 0.31211100 1.58657300
H -0.99190700 2.07971600 1.38835700
H -1.48224100 1.86917200 -1.00766600

**26-TMS-α**

b3lyp/6-31g(d,p)&6-31+g(d,p),

el. energy = -1585.244822 a.u.

C -1.08828500 -1.50114100 0.56272800
H -0.92044200 -2.52750400 0.91553400
C -2.57148400 -1.32922700 0.32034400
C -3.47108100 -2.33730900 0.68968300
C -4.84073200 -2.19047600 0.49588200
C -5.33888800 -1.01986200 -0.08770600
O -6.70078100 -0.96232000 -0.24464400
C -7.25971300 0.19235600 -0.85523800
H -8.33669500 0.02289500 -0.88623800
H -7.05193800 1.09722600 -0.27049300
H -6.88390700 0.32777000 -1.87708200
C -4.45425900 -0.00329400 -0.47067900
C -3.08379000 -0.16515600 -0.26200300
H -2.39884800 0.62461400 -0.55523000
H -4.81810400 0.90974500 -0.92713600
H -5.53878000 -2.97139100 0.77949900
H -3.09439400 -3.25584500 1.13393400
O -0.36514800 -1.32999300 -0.68096100
C 0.80987400 -0.54453900 -0.48078600
C 0.50139400 0.37129700 0.71467300
O -0.17257200 1.55624800 0.29284700
Si 0.56037400 3.06048300 0.07692400
C 1.85835500 2.98378900 -1.29035800
H 2.63185000 2.24308000 -1.05957700
H 1.40670000 2.71028900 -2.25053800
H 2.35071100 3.95569700 -1.41626700
C -0.85353000 4.20744800 -0.38810900
H -1.60711500 4.24004000 0.40598100
H -1.34568900 3.86837100 -1.30603400
H -0.49252000 5.22867500 -0.55630100
C 1.37637900 3.60818300 1.69006700
H 2.18954600 2.93511700 1.98413400

H 0.64990900 3.63814300 2.50970700
H 1.80569200 4.61159100 1.58443800
C -0.44402100 -0.49650400 1.56164900
H 0.12403200 -1.03511200 2.32660200
H -1.18485600 0.12455900 2.06726000
H 1.41777100 0.62303700 1.26049500
C 2.02238900 -1.45620800 -0.25464500
O 3.19457300 -0.65413700 -0.16320300
Si 4.77015200 -1.25594400 -0.19896800
C 5.09371300 -2.11564300 -1.84691800
H 4.92758200 -1.43050300 -2.68562600
H 6.13027300 -2.46875400 -1.90271700
H 4.44373400 -2.98597200 -1.99130900
C 5.02933200 -2.47165400 1.22209900
H 6.05864400 -2.84923100 1.22834900
H 4.36363300 -3.33854600 1.14179300
H 4.84334500 -1.99362400 2.19043000
C 5.85779500 0.26320600 0.00033300
H 5.68506900 0.97595100 -0.81309900
H 5.64549400 0.77373600 0.94580200
H 6.92019900 -0.00618300 -0.00679800
H 2.07946300 -2.15949700 -1.09599200
H 1.88803600 -2.04475600 0.66551800
H 0.97496500 0.04285600 -1.38882100
